# European Society for Paediatric Gastroenterology, Hepatology and Nutrition/North American Society for Pediatric Gastroenterology, Hepatology and Nutrition guidelines for treatment of functional constipation in children aged 0–18 years

**DOI:** 10.1002/jpn3.70447

**Published:** 2026-05-20

**Authors:** Morris Gordon, Anna de Geus, Mary Boruta, Marcin Banasiuk, Marc Benninga, Osvaldo Borrelli, Anil Darbari, Dawn Dore‐Stites, Michelle Gould, Juliette Hawa, Kirsten Jones, Alexandra Kilgore, Hayat Mousa, Samuel Nurko, Nikhil Thapar, Julie Khlevner, Vasiliki Sinopoulou, Merit Tabbers

**Affiliations:** ^1^ School of Medicine University of Central Lancashire Preston UK; ^2^ Amsterdam UMC, Paediatric Gastroenterology Amsterdam The Netherlands; ^3^ Amsterdam UMC, Amsterdam Gastroenterology Endocrinology Metabolism University of Amsterdam Amsterdam The Netherlands; ^4^ Amsterdam UMC, Amsterdam Reproduction and Development research institute Amsterdam University of Amsterdam Amsterdam The Netherlands; ^5^ Department of Pediatrics, School of Medicine Duke University Durham North Carolina USA; ^6^ Department of Paediatric Gastroenterology and Nutrition Medical University of Warsaw Warszawa Poland; ^7^ Great Ormond Street Hospital for Children London UK; ^8^ Children's National Hospital Washington District of Columbia USA; ^9^ Division of Pediatric Psychology, Department of Pediatrics, Michigan Medicine University of Michigan Ann Arbor Michigan USA; ^10^ Division of Gastroenterology, Hepatology, and Nutrition The Hospital for Sick Children Toronto Canada; ^11^ Department of Physical Therapy Children's Hospital Colorado Aurora Colorado USA; ^12^ Department of Clinical Nutrition and Lactation Nationwide Children's Hospital Columbus Ohio USA; ^13^ Department of Pediatrics, Digestive Health Institute, Children's Hospital Colorado University of Colorado School of Medicine Colorado Aurora USA; ^14^ Division of Gastroenterology, Lustgarten Motility Center The Children's Hospital of Philadelphia Philadelphia Pennsylvania USA; ^15^ Center for Motility and Functional Gastrointestinal Disorders, Boston Children's Hospital Harvard Medical School Boston Massachusetts USA; ^16^ Queensland Children's Hospital South Brisbane Queensland Australia; ^17^ School of Medicine, University of Queensland; Centre for Child Nutrition Research University of Technology Brisbane Queensland Australia; ^18^ Division of Pediatric Gastroenterology, Hepatology and Nutrition Columbia University Vagelos College of Physicians and Surgeons New York New York USA

**Keywords:** disorders of gut brain interaction, gastroenterology, pediatric, therapeutic management

## Abstract

**Objectives:**

Functional constipation (FC) is common in childhood, significantly impacting quality of life. Since the 2014 international guideline, new evidence has been published, and methods on making guidelines have developed. This treatment guideline for FC in children aged 0–18 years is a collaborative effort of the European and North American Societies for Paediatric Gastroenterology, Hepatology and Nutrition (ESPGHAN and NASPGHAN). The aim was to deliver evidence‐based recommendations applicable across all treatment settings worldwide and offer methodological guidance for future research.

**Methods:**

The guideline development followed the “Grading of Recommendations Assessment, Development and Evaluation” (GRADE) approach. The Guideline Development Group (GDG) comprised clinical experts, representing ESPGHAN, NASPGHAN and Cochrane. Prospectively agreed‐upon treatments were formatted into “patient, intervention, comparison, outcome” questions. The GDG also determined decision thresholds for efficacy and safety outcomes through a Delphi process to support grading of the literature. Final recommendations were established by consensus voting, and a treatment algorithm was developed.

**Results:**

A total of 102 original randomized controlled trials (RCTs) assessing treatment of FC in children aged 0–18 years were identified. Consensus was reached for 11 GRADE recommendations. Nineteen good practice statements were formulated, and guidance for future research methodology was proposed.

**Conclusions:**

This guideline is the result of a collaborative effort between ESPGHAN and NASPGHAN on treatment options for FC. Systematic review of the evidence has revealed major evidence gaps for commonly used treatments for FC and highlights the need for large pediatric RCTs, particularly on treatment options with no evidence or only very low certainty evidence.

## Abstract (highlights)

**What is Known:**

- Given the chronic nature of pediatric functional constipation, ongoing guidance and treatment from healthcare professionals is often needed, with each child requiring an individualized management approach.
- The last published guideline on treatment of functional constipation in children dates from 2014 and evidence was limited for the majority of interventions.

**What is New:**

- Predefined thresholds for outcome imprecision were applied, reflecting advances in evidence synthesis for guideline development.
- Recommendations are provided per treatment option, supported by a comprehensive algorithm guiding stepwise treatment.
- Polyethylene glycol (PEG) is the only treatment with a strong recommendation in the current guideline.
- There is moderate certainty evidence that linaclotide (effect size small), abdominal transcutaneous electrical stimulation in combination with pelvic floor muscle exercises (effect size moderate), and percutaneous tibial nerve stimulation in combination with pelvic floor muscle exercises (effect size small) have favorable effects and are therefore suggested as a treatment option. Magnesium (hydr)oxide (effect size small), specific probiotic mixture (effect size small), specific synbiotic preparation (effect size large), cow's milk‐free diet (effect size moderate), and pelvic floor physiotherapy (effect size large) are considered of low certainty evidence and suggested as a treatment option.
- Nineteen good practice statements have been formulated for treatments lacking sufficient evidence.

## Graphical abstract

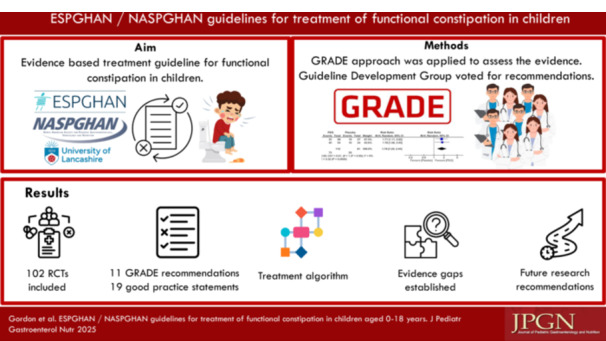

## SUMMARY OF RECOMMENDATIONS

### Pharmacological treatment: Disimpaction

#### Good practice statements

‐ The Guideline Development Group (GDG) suggests that consideration and management of **fecal impaction is vital to ensure long‐term therapeutic success** with any maintenance therapy. The GDG acknowledges that the lack of a clear definition for fecal impaction influences assessment in practice and is a barrier to achieving successful disimpaction.
‐ The GDG **suggests that high‐dose polyethylene glycol or enemas can be used** as treatment options for fecal impaction.
‐ The GDG also **recognizes other treatments that have utility in disimpaction** and may be used under locally agreed standardized protocols.

### Pharmacological maintenance treatment

#### GRADE recommendations

‐ **Polyethylene glycol** with or without electrolytes **is recommended** as a treatment option for functional constipation (FC) in children *(strong recommendation, overall moderate certainty evidence, effect size moderate)*
‐ **Magnesium (hydr)oxide is suggested** as a treatment option for FC in children *(conditional recommendation, overall low certainty evidence, effect size small)*
‐ **Prucalopride is not suggested** as a treatment option for FC in children *(conditional recommendation, overall moderate certainty evidence, no effect)*
‐ **Linaclotide is suggested** as a treatment option for FC in children *(conditional recommendation, overall moderate certainty evidence, effect size small)*
‐ **Enemas are not suggested** as routine add on to maintenance laxative treatment for FC in children *(conditional recommendation, overall low certainty evidence, no effect)* (please also see the GPS)

#### Good practice statements

‐ The GDG suggests that **lactulose has a therapeutic role** for FC in children.
‐ The GDG suggests that **bisacodyl has a therapeutic role** for FC in children.
‐ The GDG suggests that **senna has a therapeutic role** for FC in children.
‐ The GDG suggests that **sodium picosulfate has a therapeutic role** for FC in children.
‐ The GDG suggests that **liquid paraffin has a therapeutic role** for FC in children. However, **routine use is not recommended** due to the potential significant side effects.
‐ The GDG **does not suggest the use of lubiprostone** as a treatment for FC in children.
‐ The GDG **does not suggest the use of domperidone** as a treatment for FC in children.
‐ General statement regarding rectal therapies: The GDG acknowledges the **potential psychosocial impact of all per rectum therapies.** The decision to consider such therapies should be shared with children and their families, taking key factors into account.
‐ The GDG suggests **enemas have a maintenance therapeutic role** for FC in select children.
‐ The GDG suggests **rectal suppositories have a therapeutic role** for FC in select cases, either as an adjunct or as an alternative when other therapies are not effective or appropriate.
‐ The GDG suggests **transanal irrigation has a therapeutic role** for FC in select cases (where other therapies are not effective or appropriate) and under specialized expert care.
‐ The GDG suggests **botulinum toxin A injections have a therapeutic role** for FC in select cases (refractory to conventional treatments) under specialized expert care.

### Nonpharmacological maintenance treatment

#### GRADE recommendations

‐ **A specific probiotic mixture** (containing: *Lactobacillus acidophilus*, *Bifidobacterium longum*, and *Streptococcus thermophilus*) **is suggested** as a treatment option for FC in children (conditional recommendation, overall low certainty evidence, effect size small)
‐ **A specific synbiotic preparation** (multi‐strain probiotics and prebiotics*) **is suggested** as a treatment option for FC in children (conditional recommendation, overall low certainty evidence, effect size large)

*Lactobacillus casei, *Lactobacillus rhamnosus*, Lactobacillus plantarum, and *Bifidobacterium lactis* and prebiotics (fiber, polydextrose, fructo‐oligosaccharides, and galacto‐oligosaccharides)

‐ **Cow's milk‐free diet** trial for 2–4 weeks **is suggested** as an adjunct treatment option for FC in children not responding to conventional therapy alone and when cow's milk allergy is suspected *(conditional recommendation, overall low certainty evidence, effect size moderate)*
‐ **Pelvic floor physiotherapy is suggested** as an adjunct treatment option for FC in children *(conditional recommendation, overall low certainty evidence, effect size large)*
‐ **Abdominal transcutaneous electrical stimulation** in combination with pelvic floor muscle exercises **is suggested** as a treatment option for FC in children *(conditional recommendation, overall moderate certainty evidence, effect size moderate)*
‐ **Percutaneous tibial nerve stimulation** in combination with pelvic floor muscle exercises **is suggested** as a treatment option for FC in children *(conditional recommendation, overall moderate certainty evidence, effect size small)*

#### Good practice statements

‐ The GDG **recommends demystification, explanation, and guidance for toilet training** (if developmentally appropriate) in the treatment of FC in children.
‐ General statement regarding diet and physical activity: The GDG **recommends a healthy, balanced diet with age‐appropriate water and fiber intake and normal physical activity** in children with FC.
‐ The GDG has made a GRADE recommendation about specific preparations of probiotics and synbiotics. **Other pro‐ and synbiotic preparations do not have sufficient evidence** at a strain level to support such recommendations due to issues with the precision of the data and methods of the studies
‐ The GDG suggests that **behavioral interventions should not be used in isolation** but employed in select patients as an adjunct to other pharmacological therapies.
‐ The GDG suggests **not to use biofeedback alone**, but to consider it as part of a wider strategy of pelvic floor physiotherapy.

### Surgical therapy

#### Good practice statements

‐ The GDG **recognizes the role of surgical interventions in a very select group of patients** who are refractory to medical therapy. Prior to surgery, it is vital to ensure that the range of other treatments in this guideline have been carefully considered, a multi‐disciplinary team is involved, risks are weighed, appropriate investigations have been conducted, and that the postsurgical prognosis is discussed with the child and family.

## 1 INTRODUCTION

Functional constipation (FC) is a common problem in childhood, with a worldwide prevalence of 9.5%.^1^ Children with FC present with infrequent, hard, and painful stools often accompanied by fecal incontinence, without an underlying organic cause. FC greatly affects the social, physical, and emotional functioning of children and contributes significantly to the utilization of healthcare services.^2^, ^3^ FC diagnosis is based on the pediatric Rome IV criteria.^4^, ^5^ The etiology is multifactorial and not completely understood. Given the chronic nature of the condition, ongoing guidance and treatment from healthcare professionals is often needed. Children with FC present across primary, secondary, and tertiary care settings, with each child requiring a specific management approach tailored to individual circumstances and disease severity.

In 2014, the European Society for Paediatric Gastroenterology, Hepatology and Nutrition (ESPGHAN) and North American Society for Pediatric Gastroenterology, Hepatology and Nutrition (NASPGHAN) published a joint guideline on the evaluation and treatment of childhood constipation.^6^ Since then, numerous new studies have been published, and the diagnostic criteria have been updated with the release of the latest Rome IV criteria for pediatric FC. Additionally, there have been substantial developments in the methods for synthesis of evidence and guideline decision‐making. Therefore, an updated guideline was indicated.

The present treatment recommendations are established to provide a framework for shared decision‐making between patients, their caregivers and healthcare professionals. Constipation is also a common symptom in children that have other underlying organic conditions, but the present guideline is not geared towards those patients.^6^

This guideline represents the consensus of the Guideline Development Group (GDG) with the available evidence at the time of preparation. This guideline is intended for patients and their caregivers, healthcare professionals in primary, secondary, and tertiary care settings, researchers, and public policy makers. Treatment recommendations may not apply in all situations and should be interpreted in the light of specific clinical situations and resource availability. In addition, the remarks accompanying the guideline recommendations should always be considered, as they may be important in specific cases. Updates to this guideline may be necessary as new trial data emerges. The guideline is meant to serve as an educational resource to provide information to support patient care. The treatment recommendations are not rules and should not be seen as establishing a legal standard of care or promoting, requiring, or discouraging any treatment. Clinical considerations may justify an approach that diverges from the guideline recommendations.

## 2 METHODS

The development process was guided by the grading of recommendations assessment, development and evaluation (GRADE) framework, as outlined in the GRADE handbook, supported by the World Health Organization (WHO).^7^ In line with this guidance and in line with similar guidelines, a complete protocol for the technical review, along with associated operating procedures, was agreed upon in advance and previously published.^8^, ^9^, ^10^ The methodology used in developing this guideline will be described concisely, as the published protocol serves as a comprehensive reference.^10^

The GDG was chaired by a member of each of the societies (For ESPGHAN M.M.T., for NASPGHAN J.K.), as well as a GRADE methodologist pediatrician, and Editor of Biomedical Evidence Synthesis and Translation (BEST) and guideline production unit (M.G.). Wider GDG members were chosen as experts in FC management and to ensure a broad range of clinical expertise. The makeup of the GDG was reviewed and ratified by each of the two societies prospectively. The 15 voting members included pediatric gastroenterologists (M.B., M.A.B., O.B., M.B., A.D., M.J.G., J.K., A.K., H.M., S.N., M.M.T, and N.T.), a clinical psychologist (D.D.), a physical therapist (J.H.), and a clinical dietitian (K.J.). A nonvoting methodological team comprised the GRADE co‐chair (M.G.) and 2 methods members (A.G., V.S.), who were primarily responsible for technical systematic review and GRADE analysis of data and data synthesis summaries. One of the methodological team members (V.S.) is also a registered dietitian. A pediatric surgeon was invited to review and comment on the guideline, but did not vote on the recommendations. All members agreed to co‐author the full guideline and maintain the confidentiality of open discussion and debate within the process. Any relevant conflicts of interest were declared at the start of the process and again before each step of the voting process. In case of any conflicts of interest related to a particular treatment, the GDG member was not allowed to participate in the discussion nor to vote on the recommendation concerning that treatment. Patients and caregivers were involved in the development of a core outcome set for therapeutic studies in FC.^11^ This core outcome set serves as the foundation for the current guideline. Patients and caregivers were not involved in other parts of the guidelines production.

### 2.1 Type of patients

Consensus was reached to consider children with FC, refractory/therapy‐resistant constipation, and fecal impaction in the scope of the guideline. Treatment of fecal impaction was considered separately. Both refractory constipation and fecal impaction are not currently defined by international consensus. Based on current literature and by consensus of the GDG, definitions of refractory constipation and fecal impaction will be included in the guideline (see section *Definitions*).

### 2.2 Types of treatments

All pharmacological, nonpharmacological, and surgical treatment options that were prospectively agreed upon were considered and are listed in full in the previously published protocol.^10^ Pharmacological treatments were divided into two categories: management of fecal impaction and maintenance therapy. Surgical treatment will be discussed briefly, given the absence of randomized controlled trials (RCTs) and limited expertise within the GDG to make specific recommendations for individual surgical options.

We provide a summary table of typical dosing regimens used in research and clinical practice to support understanding of the guideline's recommendations. The table is included at the end of the pharmacological treatment sections. It is intended for contextual reference only and must not be used as a source of prescribing guidance. Actual prescribing should always follow current national formularies, locally approved drug lists, and regulatory authorities’ guidance, which may differ by country due to variations in licensing status, availability of formulations, and evolving safety information.

### 2.3 Outcomes

The selected outcomes were based on the previously published core outcome set for pediatric FC.^11^ Primary (critical) and secondary (important) outcomes were established, with consensus obtained from GDG members (Table 1). This was agreed prior to technical review of the evidence. Tolerability (defined as tolerability, acceptability, or compliance) was added to the core outcome set as the GDG regarded it as an important factor to consider for treatment recommendations.

**Table 1:** Selected outcomes.

Primary (critical) outcomes	
Treatment success (as defined by the authors)	Dichotomous
Defecation frequency	Dichotomous/continuous
Withdrawals due to adverse events	Dichotomous
Secondary (important) outcomes	
Painful defecation	Dichotomous/continuous
Stool consistency	Dichotomous/continuous
Quality of life or change in quality life measured using any validated measurement tool	Dichotomous/continuous
Fecal incontinence (if age appropriate)	Dichotomous/continuous
Abdominal pain (if age appropriate)	Dichotomous/continuous
School attendance (if age appropriate)	Dichotomous/continuous
Serious adverse events	Dichotomous
Total adverse events	Dichotomous
Tolerability, or defined as acceptability or compliance	Dichotomous/continuous

### 2.4 Thresholds for outcomes

The established thresholds for all the selected outcomes were previously published.^10^ The same approach as described in detail in the ESPGHAN/NASPGHAN functional abdominal pain disorders treatment guideline was used to establish the thresholds.^9^ See Figure 2 for the thresholds for the dichotomous outcomes (efficacy and safety) and Table 2 for the continuous outcomes.

**Table 2:** Thresholds for continuous outcomes.

Outcome	Trivial to small	Small to moderate	Moderate to large
Increase in defecation frequency per week	1.2	2.3	3.7
Decrease in painful defecations per week	1.1	2.2	3.5
Decrease of pain during defecation on VAS‐score (0–100)	13	26	41
Change in stool consistency on the Bristol Stool Form Scale (1–7, 1 = very hard stools, 7 = very soft stools)	0.8	1.5	2.3
Improvement in quality of life on PedsQL score (0–100)	13	23	38
Decrease in fecal incontinence frequency per week	1.0	2.4	4.0
Decrease of abdominal pain measured on a 0–4‐point scale (0 = no pain, 4 = a lot of pain)	0.7	1.2	2.0
Number of school days missed per month	3	6	9
Tolerability on 4‐point Likert scale (0 = poor tolerability, 4 = excellent tolerability)	0.6	1.1	1.9

Abbreviations: PedsQL, pediatric quality of life inventory; VAS, visual analog scale.

### 2.5 Search strategy

**Figure 1:**
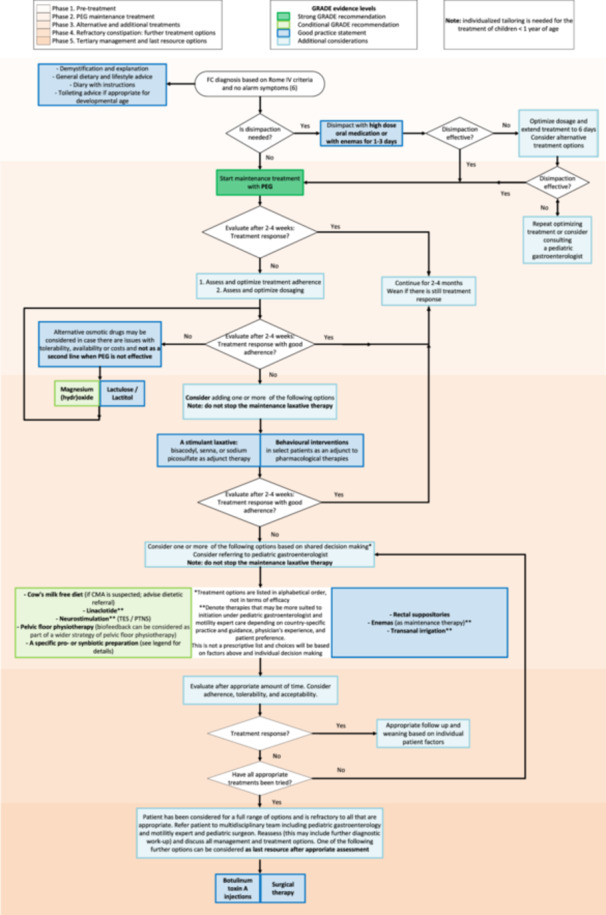
Treatment algorithm for FC: The GRADE data synthesis is the primary source informing the flow of treatments. However, best practice statements and additional considerations have also been included as they may be of additional value. The suggested flow provides a framework for shared decision‐making. The choice of any particular treatment option should strongly reflect the balance of efficacy and safety from an individual patient perspective, local availability and feasibility, and potentially legal frameworks. The consecutive order of treatments as proposed in this algorithm, may as such be modified following local context. As there is currently no international consensus or evidence regarding the definition of treatment success/treatment response for FC in children, a specific definition was not included in this guideline. Whether a patient has achieved treatment response is left to the discretion and clinical expertise of the treating physician. A specific probiotic mixture containing *Lactobacillus acidophilus*, *Bifidobacterium longum*, and *Streptococcus thermophilus*. A specific synbiotic preparation containing multi‐strain probiotics *Lactobacillus casei*, *Lactobacillus rhamnosus*, *Lactobacillus plantarum*, and *Bifidobacterium lactis* and prebiotics (fiber, polydextrose, fructo‐oligosaccharides, and galacto‐oligosaccharides). CMA, cow's milk allergy; FC, functional constipation; GRADE, grading of recommendations assessment, development and evaluation; PEG, polyethylene glycol; PTNS, percutaneous tibial nerve stimulation; TES, transcutaneous electrical stimulation.

An experienced Cochrane information specialist conducted a comprehensive search of the following databases: the Cochrane Central Register of Controlled Trials (CENTRAL) (via Ovid EBMR) (inception to present); Embase (via Ovid) (1974 to present); MEDLINE (via Ovid) (1946 to present); PubMed (excluding MEDLINE) (inception to present); ClinicalTrials. gov; WHO ICTRP. The search for pharmacological interventions for maintenance treatment is up to date as of December 2022, for pharmacological interventions for fecal impaction up to November 2023, for nonpharmacological interventions as of May 2023, and surgical interventions as of May 2024. No language restrictions were applied. Abstract publications were included to reduce publication bias, and authors were contacted for additional information. To identify additional studies, the reference lists of relevant systematic reviews were checked, and experts were contacted. The GDG was presented a final file with all included studies and was given the change to add any missing studies. The preferred reporting items for systematic reviews and meta‐analyses (PRISMA) flowchart shows the results of the entire search (Supporting Information: File S1—Figure 1 and Table 1).

### 2.6 Study selection, data collection, and analysis

Only RCTs enrolling children from the age of 0–18 years, with FC, with or without fecal impaction, or with refractory/therapy‐resistant constipation as defined by the authors, were included. In addition, trials were included when interventions of interest for FC were compared with other active interventions or standard therapy, placebo, or no therapy.

Pairwise meta‐analyses were performed using Review Manager (Version 5.4, the Cochrane Collaboration). Network meta‐analysis for all pharmacological and nonpharmacological treatments was planned as an adjunct to pairwise meta‐analysis. However, we assessed the assumption of transitivity by comparing the distribution of potential effect modifiers across pairwise comparisons and found that it was not sufficiently met. Therefore, we performed a network meta‐analysis for the pharmacological treatments only, which met the transitivity assumption. We employed a frequentist framework using multivariate meta‐analysis.^12^ Heterogeneity was assessed using the I2 statistic for each pairwise comparison, and with the loop‐specific approach for the direct and indirect estimates. Surface under the cumulative ranking curve was used to rank treatments. Statistical analyses were performed using the R statistical software and netmeta package.^13^

### 2.7 Certainty of the evidence

Risk of bias was assessed using the Cochrane risk‐of‐bias tool for RCTs, and certainty of the evidence was assessed using the GRADE approach.^14^ Since the core outcome set was used (Table 1), indirectness of reported outcomes was not considered an issue and rated as “not serious” by default. Publication bias had been addressed through the search strategy, with insufficient study numbers to allow funnel plot use and therefore was also judged as “not serious” by default. Following data extraction, the GDG decided not to GRADE the outcome of tolerability, due to a significant heterogeneity across studies in terms of how tolerability was defined and reported.

### 2.8 Voting

All GDG members completed educational modules on the GRADE approach to enhance their understanding of the evidence summaries prior to the voting process. Prior to attending a 2‐day guideline summit held in Reykjavik, Iceland, in October 2024, all voting GDG members received a comprehensive technical summary of the data synthesis. Evidence‐based recommendations were proposed by the methodologists (M.G., A.G., and V.S.) and were discussed in detail by the GDG, including the various factors in evidence to decision frameworks influencing the strength of final recommendations.^15^

After discussing the exact wording and strength of the recommendation, the GDG proceeded to vote.

At least 75% of the voting members had to agree with the explicit formulation. This threshold was maintained even if members with conflicts of interest were excluded.

Votes were held for both GRADE recommendations and Good Practice Statements (GPS) using the same methodology. Voting was conducted digitally and anonymously through a binary system (agree/disagree) using an online platform (www.slido.com). When consensus was reached, the justifications and other considerations were finalized through discussion. When no consensus was reached, the GDG could propose a new recommendation through discussion and vote again. If consensus was still not reached, the treatment option is mentioned with the note that the GDG was unable to reach consensus and, therefore, no recommendation could be made.

Following GRADE methodology, recommendations are either positive (in favor) or negative (against) and labeled as strong or conditional. The strength and direction of these recommendations are based on the GRADE conclusions of the synthesized evidence (Table 3), complemented with any other considerations raised by the GDG members. The phraseology and terminology used to formulate the recommendations are outlined in Table 4. For some treatment options lacking sufficient evidence for a GRADE recommendation, GPS were developed instead. GPS was based on expert consensus rather than directly informed by the evidence gathered for these guidelines. The GDG voted on the final proposed GPS, and a 100% consensus was required. There were no amendments or deviations from the published protocol.

**Table 3:** Terminology used for GRADE certainty levels of evidence.

GRADE certainty	Definition	Terminology
High	The estimate of the effect matches the actual effect	Definitely
Moderate	The estimate of the effect probably matches the actual effect	Probably
Low	The estimate of the effect may match the actual effect	Maybe
Very low	No conclusion can be drawn	Unclear

Abbreviation: GRADE, grading of recommendations assessment, development and evaluation.

**Table 4:** Terminology used for the formulation of recommendations.

Recommendation terminology	Direction	Explanation	Terminology
Strong recommendation	In favor	Benefits clearly outweigh the harms	Recommend
Conditional recommendation	In favor	Benefits probably outweigh the harms/particular considerations limit generalization	Suggest
Conditional recommendation	Against	Harms may outweigh the benefits	Not suggest
Strong recommendation	Against	Harms definitely outweigh the benefits	Not recommend

### 2.9 Presentation of results

Data summaries are presented in a clear and consistent structure throughout the guideline. In this manuscript, recommendations and the corresponding considerations are described per treatment option. Only data on crucial efficacy outcomes are displayed in the evidence summary tables and safety data are narratively explained. The overall magnitude of effect was based on the crucial efficacy outcomes. If a treatment option was compared to both placebo/standard care and other active interventions, the magnitude of effect of the comparison of the treatment with placebo/standard care was given the highest weight when determining the overall magnitude of effect. When an intervention showed no significant difference, magnitude of effect was not applicable.

**Figure 2:**
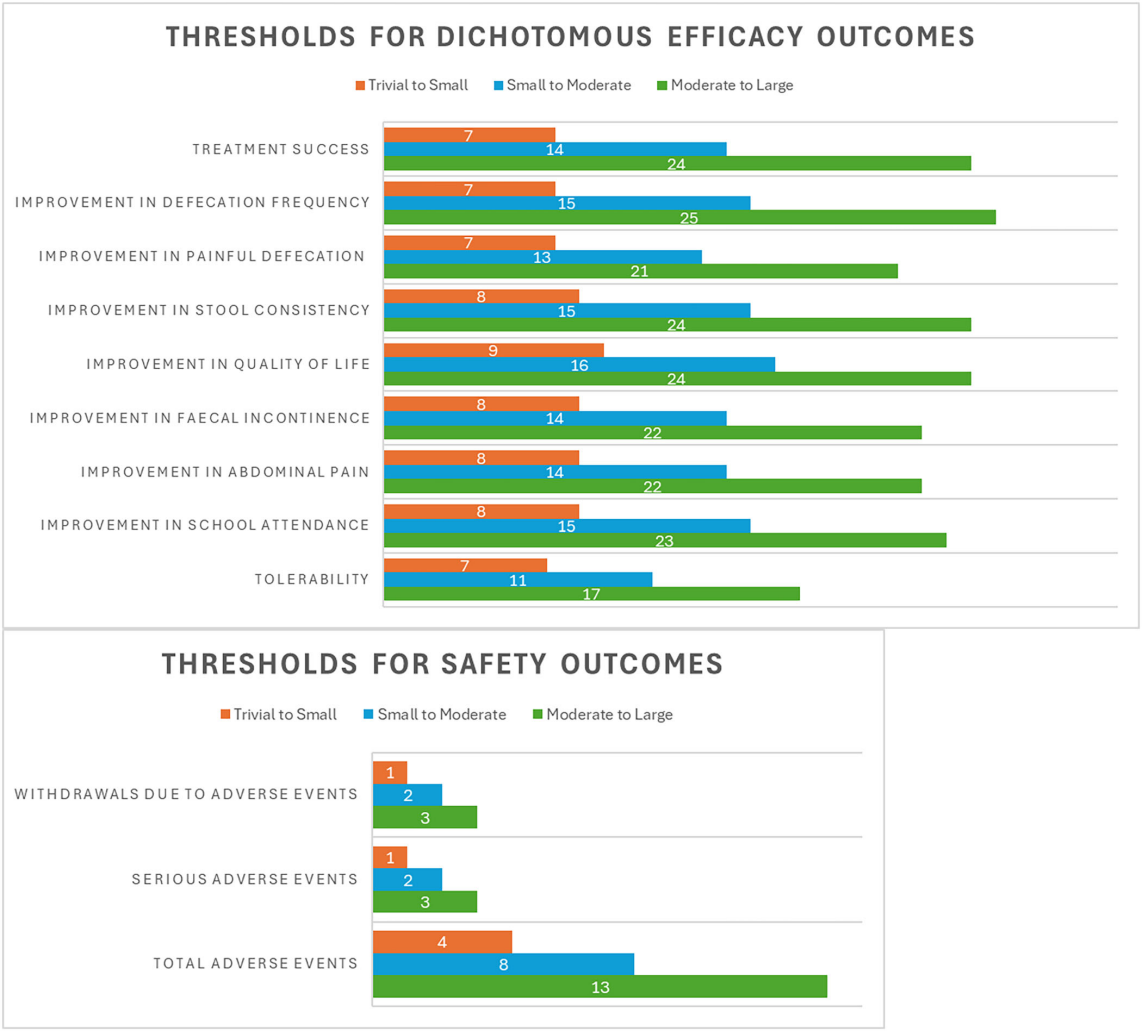
Thresholds for the dichotomous outcomes.

**Figure 3:**
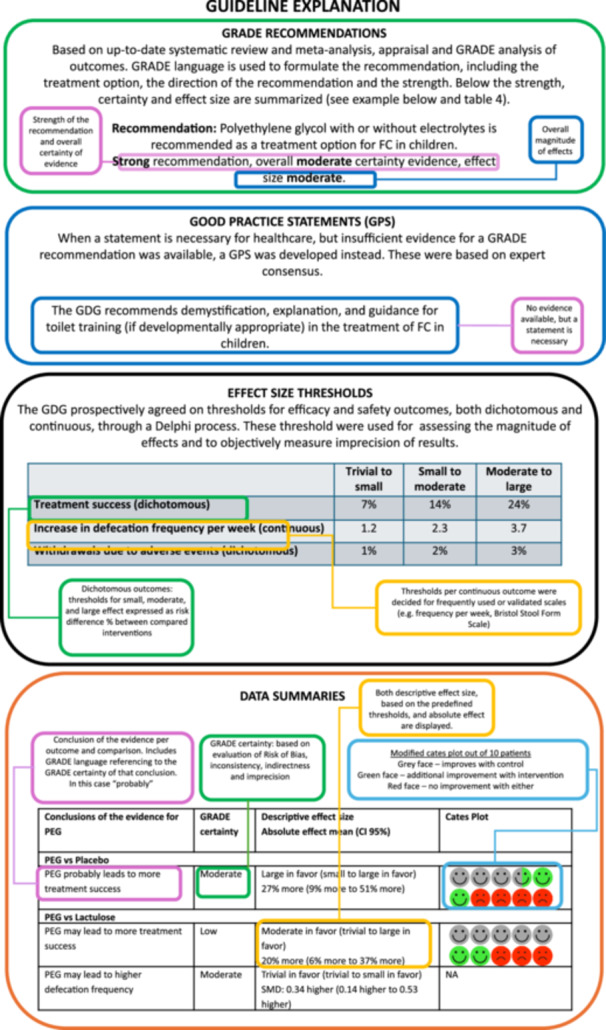
Infographic on guideline explanation. CI, confidence interval; FC, functional constipation; GDG, guideline developing group; GRADE, grading of recommendations assessment, development and evaluation; PEG, polyethylene glycol; SMD, standardized mean difference.

The treatment algorithm (Figure 1) presents a suggested stepwise sequence of interventions, intended to guide clinical decision‐making while allowing for individualization based on patient circumstances. The presentation of the guideline is explained in Figure 3. Supporting Information: File S1 includes risk of bias assessments (Figures 2 and 3, and Table 2), characteristics of included studies (Tables 3 and 4), data on primary, secondary, and safety outcomes (Tables 5, 6, 7, 8), and voting results (Table 9, 10, 11, 12, 13, 14, 15, 16). A full technical summary of evidence summaries, meta‐analyses, and grading can be found in Supporting Information: File S2.

## 3 DEFINITIONS USED IN THIS GUIDELINE

### 3.1 Refractory/therapy‐resistant constipation

Constipation that is not responding to a maximum dose of at least two laxatives of different classes for a minimum of 3 months with good compliance in a secondary or tertiary care facility.^18^, ^19^

### 3.2 Fecal impaction

a prolonged period of no voluntary passage of stool per rectum, associated with a history of hard and difficult to pass stools (Bristol stool scale number 1 or 2) and clinical evidence of hard feces (e.g., palpable abdominal mass).^20^

## 4 RESULTS

### 4.1 Pharmacological treatment: Disimpaction

#### 4.1.1 GRADE recommendations

Based on the available evidence, no GRADE recommendations could be made.

#### 4.1.2 Good practice statements

- The GDG suggests that consideration and management of **fecal impaction is vital to ensure long term therapeutic success** with any maintenance therapy. The GDG acknowledges that the lack of a clear definition for fecal impaction influences assessment in practice and is a barrier to achieving successful disimpaction.
- The GDG **suggests that high dose polyethylene glycol (PEG) or enemas can be used** as treatment options for fecal impaction.
- The GDG also **recognizes other treatments that have utility in disimpaction** and may be used under locally agreed standardized protocols.

The mechanism of action of PEG for disimpaction is identical to that of PEG used for maintenance therapy and is described in the corresponding PEG recommendation.

In these guidelines, the term “enemas” refers to retrograde enemas. If a different type of enema is intended, this will be specified explicitly. Enemas are fluids introduced rectally that contain active components increasing gut motility or intestinal fluid secretion. Some enemas contain multiple ingredients combining different mechanisms of action that cause the laxative effect. The effect typically occurs within a few minutes after the enema is administered.

##### Rationale

Several RCTs have not been included in the guideline due to the lack of definition of fecal impaction; therefore, the evidence for treatment of fecal impaction was limited. Recent literature shows that there is a lack of consensus on the definition of fecal impaction.^20^ However, there was substantial GDG experience with the use of high‐dose PEG and enemas for the treatment of fecal impaction with good efficacy. It should always be considered that per rectum therapy may be burdensome for the child and/or caregiver. Various enemas with different active substances or combinations are available, commonly including sodium docusate, sodium phosphate, sodium lauryl sulfoacetate, or sodium chloride (NaCl).^21^ However, there is no evidence regarding which enema is most effective. There is a role for several other treatments in disimpaction, such as oral laxatives or combinations of therapies and manual disimpaction under general anesthesia.^22^ Based on clinical experience, other oral laxatives that may be used for disimpaction when an alternative to PEG with or without electrolytes is needed include magnesium hydroxide, lactulose, lactitol, liquid paraffin (mineral oil), senna, bisacodyl, and sodium picosulfate.^21^, ^23^ However, evidence on their efficacy and well‐established dosages for this indication are lacking. Pharmacological therapies are recommended, either oral or rectal, for 3–6 days in children presenting with fecal impaction. Dosage plays a crucial role in the success of disimpaction, and local protocols should be consulted for correct preparation and dosing.

### 4.2 Pharmacological treatment: Maintenance

#### 4.2.1 GRADE recommendations

*Should **PEG** be used as a treatment option for FC?*

Recommendation: PEG with or without electrolytes **is recommended** as a treatment option for FC in children.

**Strong** recommendation, overall **moderate** certainty evidence, effect size **moderate** (Table 5).

**Table 5:** Data summary for PEG.

Conclusions of the evidence for PEG	GRADE certainty	Descriptive effect size absolute effect mean (CI 95%)	Cates plot
PEG versus placebo			
PEG probably leads to more treatment success	Moderate	Large in favor (small to large in favor) 27% more (9% more to 51% more)	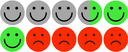
PEG versus Lactulose			
PEG may lead to more treatment success	Low	Moderate in favor (trivial to large in favor) 20% more (6% more to 37% more)	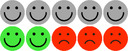
PEG may lead to higher defecation frequency	Moderate	Trivial in favor (trivial to small in favor) SMD: 0.34 higher (0.14 higher to 0.53 higher)	NA
PEG versus magnesium hydroxide			
PEG may lead to more treatment success	Low	Large in favor (small to large in favor) 29% more (10% more to 56% more)	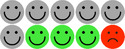
PEG may lead to less withdrawals due to adverse events	Moderate	Large in favor (large to small in favor) 10% less (14% less to 1% less)	NA
PEG versus sodium picosulfate			
There may be no difference in defecation frequency	Low	Moderate in favor (small against to large in favor) 16% more (14% less to 71% more)	NA
PEG versus sodium picosulfate + fiber			
PEG may lead to less children with higher defecation frequency	Low	Trivial against (trivial to large against) 3% less (1% less to 52% less)	NA
PEG versus fiber			
There may be no difference in defecation frequency	Low	Trivial in favor (large against to large in favor) 2% more (25% less to 50% more)	NA
PEG + fiber versus fiber			
There may be no difference in defecation frequency	Low	Moderate in favor (small against to large in favor) 8% less (22% less to 44% more)	NA
PEG versus synbiotics			
PEG may lead to higher defecation frequency per week	Low	Trivial in favor (trivial to trivial in favor) MD: 0.75 higher (0.36 higher to 1.14 higher)	NA
PEG + probiotics versus fiber			
PEG + probiotics may lead to higher defecation frequency per week	Low	Small in favor (small to small in favor) MD: 1.55 higher (1.37 higher to 1.73 higher)	NA

Abbreviations: CI, confidence interval; GRADE, grading of recommendations assessment, development and evaluation; MD, mean difference; NA, not applicable; PEG, polyethylene glycol; SMD, standardized mean difference.

PEG is an osmotic laxative. PEG is a long linear polymer and is not metabolized in the gastrointestinal tract. PEG 3350 and PEG 4000 with or without electrolytes are the most common forms used as laxatives, and they are not absorbed due to their high molecular weight. These medications form hydrogen bonds with water, inhibiting re‐absorption of water in the colon, resulting in an increased fluid volume in the intestines and softer stools. These properties are responsible for the laxative effect.

##### Summary of evidence

A total of 32 RCTs investigating PEG were included (*n* = 3302, age range 6 months–18 years). PEG was compared to 14 different interventions. A total of three studies compared PEG to placebo,^24^, ^25^, ^26^ eight to lactulose,^27^, ^28^, ^29^, ^30^, ^31^, ^32^, ^33^, ^34^ four to magnesium hydroxide,^30^, ^35^, ^36^, ^37^ one to sodium picosulfate^38^ and to the combination of sodium picosulfate and fibers,^38^ two to liquid paraffin,^39^ one to enemas,^40^ three to fibers,^38^, ^41^, ^42^ one as addition to fibers,^38^ one to synbiotics,^42^ seven to herbal medicines,^43^, ^44^, ^45^, ^46^, ^47^, ^48^, ^49^ one to dry cupping,^50^ and one to manual therapy.^51^ Two studies compared PEG without electrolytes to PEG with electrolytes,^52^, ^53^ and one compared PEG in a dose of 0.7 g/kg/day to PEG 0.3 g/kg/day.^54^

##### Efficacy

Meta‐analysis and GRADE assessment showed PEG probably leads to more treatment success compared to placebo (65% vs. 36%; relative risk [RR]: 1.74 [1.25–2.41]; number needed to treat [NNT] 4 [2–10]; large magnitude of effect, moderate certainty). PEG may also lead to more treatment success compared to lactulose (72% vs. 51%; RR 1.35 [1.11–1.64]; NNT 5 [3–16]; moderate magnitude of effect; low certainty) and magnesium hydroxide (89% vs 60%; RR: 1.50 [1.16–1.94]; NNT 3 [2–10]; large magnitude of effect; low certainty). Definitions of treatment success varied between studies (see Supporting Information: File S1—Table 3). After a sensitivity analysis, the meta‐analysis showed PEG probably leads to a trivial difference in defecation frequency (standardized mean difference [SMD]: 0.34 [0.14–0.53]; trivial magnitude of effect; moderate certainty) compared to lactulose. There may be no difference in the number of children with increased defecation frequency for PEG compared to sodium picosulfate (63% vs. 47%; RR: 1.33 [0.71–2.50]; low certainty). PEG compared to sodium picosulfate in combination with fiber may lead to a trivial difference favoring sodium picosulfate in combination with fiber for the number of patients having more than three bowel movements per week (63% vs. 94%; RR: 0.67 [0.45–0.99]; trivial magnitude of effect; low certainty).

There may be no difference in defecation frequency (as a dichotomous outcome) for PEG in combination with fiber compared to fiber alone (75% vs 60%; RR: 1.25 [0.76–2.06]; low certainty). PEG may lead to a trivial difference in defecation frequency per week compared to synbiotics (mean difference (MD): 0.75 [0.36–1.14]; trivial magnitude of effect; low certainty). PEG in combination with probiotics may lead to higher defecation frequency per week compared to fiber alone (MD: 1.55 [1.37–1.73]; small magnitude of effect; low certainty).

##### Safety

Meta‐analysis of withdrawals due to adverse events data showed PEG probably leads to less withdrawals due to adverse events compared to magnesium hydroxide (6% vs 17%; RR: 0.38 [0.16–0.92]; large magnitude of effect; moderate certainty). Meta‐analyses showed there may be no difference between PEG and lactulose for total adverse events (27% vs. 33%; RR: 0.85 [0.69–1.06]; low certainty) and no difference between PEG and herbal medicine (15% vs. 9%; RR: 1.68 [0.53–5.34]; low certainty evidence). All other safety outcomes were assessed as very low, mainly caused by very imprecise data as a result of limited number of events. Reported adverse events related to PEG included diarrhea, abdominal pain, bloating, and nausea.

##### Risk of bias

Risk of bias issues were common across all studies and were assessed as serious or very serious in most of the GRADE assessments. Common reasons included a lack of blinding, inadequate reporting of allocation concealment, and the absence of trial registration details, which hindered assessment of selective reporting.

##### Rationale

A strong recommendation for PEG was made after evaluating the benefits and harms of the treatment. PEG is the most studied therapy for FC in children, and there is moderate and low certainty evidence that PEG leads to more treatment success (with absolute effect sizes ranging from 65% to 89%) compared to placebo and two other osmotic laxatives, with a moderate to large magnitude of effect. No serious adverse events related to PEG were reported. PEG is licensed in many places and widely used across the world. There is no evidence that the use of PEG is associated with neurotoxicity, a concern raised by anecdotal reports.^55^, ^56^ It is available in neutral form and in different types of flavors.

The studies only included children aged 6 months and older, but there is widespread GDG experience from 1 month with no serious concerns with efficacy or serious adverse events. In addition, observational studies corroborate the safety data from RCTs.^57^ Therefore, the GDG recognizes that there could be a role for PEG in children younger than 6 months old. However, the lack of evidence in children of this age is key to consider before prescribing.

PEG was orally administered in all studies, with no trial reporting its use through other enteral routes. There is experience in the GDG using PEG via other enteral routes (including feeding tubes), but it should be highlighted that there may be special considerations in patients at risk for aspiration.

The volume of PEG can be larger than other laxatives, making it important to provide appropriate counseling and set clear expectations with children and caregivers. Compliance may also be compromised due to the taste of PEG (with or without electrolytes). Compliance should be monitored, and local protocols for correct preparation, dosing, and dose escalation should always be considered. Alternative osmotic drugs, such as lactulose and magnesium (hydr)oxide may be considered in case there are issues with tolerability, availability, or costs and not as a second line when PEG is not effective.

*Should **magnesium (hydr)oxide** be used as a treatment option for FC?*

Recommendation: magnesium (hydr)oxide **is suggested** as a treatment option for FC in children.

**Conditional** recommendation, overall **low** certainty evidence, effect size **small** (Table 6).

**Table 6:** Data summary for magnesium (hydr)oxide.

Conclusions of the evidence for magnesium (hydr)oxide	GRADE certainty	Descriptive effect size absolute effect mean (CI 95%)	Cates plot
Magnesium oxide versus placebo			
Magnesium oxide may lead to higher defecation frequency per week	Low	Small in favor (small to small in favor) MD: 2.15 higher (1.46 higher to 2.84 higher)	NA
Magnesium hydroxide versus PEG			
Magnesium hydroxide may lead to less treatment success	Low	Large against (large against to small against) 29% less (56% less to 10% less)	NA
Magnesium hydroxide may lead to more withdrawals due to adverse events	Moderate	Large against (small against to large against) 10% more (1% more to 14% more)	NA

Abbreviations: CI, confidence interval; GRADE, grading of recommendations assessment, development and evaluation; MD, mean difference; NA, not applicable; PEG, polyethylene glycol.

Magnesium (hydr)oxide is an osmotic laxative and also known as milk of magnesia. It is a poorly absorbed compound, which results in an osmotic gradient causing an influx of water into the intestinal lumen and thereby causing the laxative effect.

##### Summary of evidence

Six RCTs investigating magnesium (hydr)oxide were included (*n* = 406, age range 0–18 years). Three studies compared magnesium hydroxide to PEG^35^, ^36^, ^37^ and one to both PEG and lactulose.^30^ One study compared magnesium oxide to probiotics and placebo^58^ and one study compared magnesium oxide to probiotics and the combination of magnesium oxide with probiotics to probiotics alone.^59^

##### Efficacy

Meta‐analysis and GRADE assessment including four studies showed magnesium hydroxide may lead to less treatment success compared to PEG (89% vs. 60%; RR: 1.50 [1.16–1.94]; NNT 3 [2–10]; large magnitude of effect; low certainty). Magnesium oxide may lead to higher defecation frequency per week compared to placebo (one study; MD: 2.15 [1.46–2.84]; small magnitude of effect; low certainty).

##### Safety

Meta‐analysis and GRADE assessment showed magnesium hydroxide probably leads to more withdrawals due to adverse events compared to PEG (6% vs. 17%; RR: 0.38 [0.16–0.92]; large magnitude of effect; moderate certainty). All other safety outcomes were assessed as very low certainty, and no conclusions could be drawn. Reported adverse events related to magnesium (hydr)oxide included diarrhea, abdominal pain, bloating, and nausea.

##### Risk of bias

Risk of bias issues were common across all studies and were assessed as very serious in almost all GRADE assessments. Four studies were open label, performance and detection bias were assessed as high risk in these studies.^30^, ^35^, ^36^, ^37^ Inadequate reporting of allocation concealment and the absence of trial registration details were also common.

##### Rationale

A conditional recommendation for magnesium (hydr)oxide was made after evaluating the benefits and the harms of the treatment. Evidence on the effectiveness of magnesium (hydr)oxide is difficult to interpret due to limited placebo‐controlled studies. There is some direct evidence of magnesium oxide versus placebo showing some efficacy (small magnitude of effect and low certainty evidence). Magnesium hydroxide may be less effective than PEG for treatment success and probably leads to more children discontinuing the treatment. However, the absolute effect size of outcomes must be considered, as one study showed a treatment success in 60% of the children that were on magnesium hydroxide and meta‐analysis of four studies for defecation frequency showed a mean frequency per week of 4.67 (standard deviation [SD]: 2.29) at the end of treatment. No serious adverse events have been reported in the included studies. Magnesium (hydr)oxide may be considered as an alternative to PEG if PEG is not available or if patients experience issues with the palatability or the amount of volume that needs to be consumed for PEG. Caution may be needed in children with renal issues, based on isolated reports in adults.^60^, ^61^, ^62^, ^63^

*Should **prucalopride** be used as a treatment option for FC?*

Recommendation: prucalopride **is not suggested** as a treatment option for FC in children.

**Conditional** recommendation, overall **moderate** certainty evidence, no effect (Table 7).

**Table 7:** Data summary for prucalopride.

Conclusions of the evidence for prucalopride	GRADE certainty	Descriptive effect size Absolute effect mean (CI 95%)	Cates plot
Prucalopride versus placebo			
There may be no difference in treatment success	Low	Trivial in favor (trivial against to moderate in favor) 6% more (2% less to 22% more)	NA
There is probably no difference in defecation frequency per week	Moderate	Trivial in favor (trivial against to small in favor) MD: 0.50 higher (0.06 lower to 1.06 lower)	NA
There may be no difference in withdrawals due to adverse events	Low	Large against (moderate in favor to large against) 3% more (2% less to 18% more)	NA

Abbreviations: CI, confidence interval; GRADE, grading of recommendations assessment, development and evaluation; MD, mean difference; NA, not applicable.

Prucalopride is a selective, high‐affinity 5‐hydroxytryptamine (5‐HT4) receptor agonist. Serotonin, also known as 5‐HT, is a neurotransmitter found in both the central and the enteric nervous system. Prucalopride binds to 5‐HT4 receptors in the intestine, stimulating increased fluid secretion and gut motility, which facilitates stool passage and producing its laxative effect.

##### Summary of evidence

One RCT investigating prucalopride was included (*n* = 215, age range 6 months–18 years).^64^ The study compared prucalopride to a placebo.

##### Efficacy

GRADE assessment showed that there may be no difference in treatment success compared to placebo (14% vs. 8%; RR: 1.68 [0.77–3.68]; low certainty) and that there is probably no difference in defecation frequency per week (MD: 0.50 [–0.06 to 1.06]; moderate certainty).

##### Safety

GRADE assessments showed that there may be no difference in withdrawals due to adverse events (7% vs. 5%; RR: 1.61 [0.55–4.78]; low certainty). There may be no difference in serious adverse events compared to placebo (5% vs. 2%; RR: 2.52 [0.50–12.72]; low certainty). Serious treatment‐emergent adverse events consisted of abdominal pain (*n* = 1), vomiting (*n* = 1), diarrhea (*n* = 1), nausea (*n* = 1), appendicitis (*n* = 1), pneumonia (*n* = 1), dizziness (*n* = 1), syncope (*n* = 1), anxiety (*n* = 1) in the prucalopride group and of abdominal pain (*n* = 1), constipation (*n* = 1), anorectal discomfort (*n* = 1) in the placebo group. GRADE assessment showed that prucalopride may cause more side effects compared to placebo (95% vs. 57%; RR: 1.42 [1.23–1.63]; large magnitude of effect; low certainty).

Most common reported adverse events related to prucalopride included headache, pyrexia, abdominal pain, vomiting, and nausea. The study also had an open‐label safety period of an additional 16 weeks of treatment in which patients were randomized for prucalopride or PEG. During this period, a similar proportion of patients in both groups experienced adverse events (prucalopride: 64.3% vs. PEG: 61.6%).

##### Risk of bias

There was no risk of bias issues, and all domains were assessed as low risk of bias.

##### Rationale

A conditional recommendation against prucalopride was made after evaluating the lack of evidence favoring prucalopride over placebo and evaluating the benefits and the harms of the treatment. There is low certainty evidence that prucalopride is not more effective compared to placebo for treatment success and defecation frequency. It may lead to more adverse events compared to placebo, but the open‐label phase showed no difference in adverse events compared to PEG. Due to moderate certainty that prucalopride is not effective based on current evidence, routine use in FC is not recommended. Future research may be needed to consider its role as an adjunct, aligning with the clinical experiences of GDG members.

*Should **linaclotide** be used as a treatment option for FC?*

Recommendation: Linaclotide **is suggested** as a treatment option for FC in children.

**Conditional** recommendation, overall **moderate** certainty evidence, effect size **small** (Table 8).

**Table 8:** Data summary for linaclotide.

Conclusions of the evidence for linaclotide	GRADE certainty	Descriptive effect size Absolute effect mean (CI 95%)	Cates plot
Linaclotide versus placebo			
There may be no difference in treatment success	Low	Trivial in favor (trivial against to moderate in favor) 3% more (4% less to 14 more)	NA
Linaclotide may lead to higher defecation frequency per week	Moderate	Small in favor (trivial to small in favor) MD: 1.17 higher (0.45 higher to 1.89 higher)	NA
There may be no difference in withdrawals due to adverse events	Low	Moderate in favor (large in favor to large against) 2% less (5% less to 5% more)	NA

Abbreviations: CI, confidence interval; GRADE, grading of recommendations assessment, development and evaluation; MD, mean difference; NA, not applicable.

Linaclotide is a prosecretory agent that activates the guanylate cyclase‐C receptor on the epithelial channels in the intestine, increasing intestinal fluid secretion and colonic motility, thereby producing its laxative effect.

##### Summary of evidence

Three studies investigating linaclotide were included (*n* = 443, age range 2–17 years).^65^, ^66^, ^67^ All three studies compared linaclotide to placebo. One study was a phase 2 study in patients aged 6–17 years.^65^ This study investigated different dosages, of which we only included the data from the high dose group (72 μg), as this matched the dose of the phase 3 trial,^66^ also performed in children aged 6–17 years. Another study was also a phase 2 trial in children aged 2–5 years old, investigating different dosages.^67^ We only included data from the high dose group (72 μg), as this matches the dose of the ongoing phase 3 trial (72 μg) (NCT05652205).

##### Efficacy

Meta‐analysis and GRADE assessments showed that linaclotide may not lead to more treatment success compared to placebo (15% vs. 12%; RR 1.21 [0.69–2.13]; low certainty). Meta‐analysis did show that linaclotide probably leads to higher defecation frequency per week compared to placebo (MD: 1.17 [0.45–1.89]; small magnitude of effect; moderate certainty).

##### Safety

Meta‐analyses and GRADE assessments showed there may be no difference between linaclotide and placebo for serious adverse events (1% vs. 1%; RR: 1.00 [0.14–7.01]; low certainty) and total adverse events (21% vs. 15%; RR: 1.55 [0.84–2.84]; moderate magnitude of effect; low certainty). One treatment‐related severe adverse event was reported in the linaclotide group and consisted of diarrhea resulting in dehydration and hospitalization. Reported adverse events related to linaclotide included diarrhea and nausea.

##### Risk of bias

Risk of bias was assessed as not serious for all GRADE assessments, so the evidence was not downgraded for risk of bias. Almost all domains of the included trials were assessed as low risk of bias, except for performance bias and other bias in one study,^67^ which were assessed as unclear risk.

##### Rationale

A conditional recommendation for linaclotide was made after evaluating the benefits and the harms of the treatment. Meta‐analysis showed there is probably no difference in treatment success compared to placebo. However, there is direct evidence for linaclotide versus placebo showing that linaclotide probably leads to a difference in defecation frequency. As this is a critical outcome and there were no safety issues raised for the drug in the phase 3 trial investigating children aged 6–17 years, the GDG suggests that there is a role for linaclotide in the treatment of FC in children aged 6–17 years.^66^ Linaclotide is well tolerated and is taken orally as a capsule. In children aged 2–5 years, there is only data available from a phase 2 trial including 35 children.^67^ For patients who are unable to swallow the capsule, capsules can be opened and be mixed with food or liquids. Linaclotide should only be considered in children aged 6–17 years old when other pharmacological therapies have not been effective and should be prescribed by a pediatric gastroenterologist. Accessibility and cost may be limiting factors in some countries.

*Should **enemas** be used as a maintenance treatment option for FC?*

Recommendation: Enemas **are not suggested** as routine add‐on to maintenance laxative treatment for FC in children.

**Conditional** recommendation, overall **low** certainty evidence, no effect (Table 9).

**Table 9:** Data summary for enemas as maintenance therapy.

Conclusions of the evidence for enemas	GRADE certainty	Descriptive effect size absolute effect mean (CI 95%)	Cates plot
Enema + PEG versus PEG			
There may be no difference in treatment success	Low	Small in favor (trivial against to large in favor) 12% more (6% less to 40% more)	NA
There may be no difference in withdrawals due to adverse events	Low	Large against (small in favor of large against) 16% more (1% less to 100% more)	NA

Abbreviations: CI, confidence interval; GRADE, grading of recommendations assessment, development and evaluation; NA, not applicable; PEG, polyethylene glycol.

The mechanism of action of enemas for maintenance therapy is identical to that of enemas used for disimpaction and is described in the corresponding recommendation.

##### Summary of evidence

One RCT investigating enemas was included (*n* = 102, age range 8–18 years).^68^ Children with FC for at least 2 years and unresponsive to conventional treatment were included. The study compared the addition of enemas to PEG as maintenance treatment to treatment with PEG alone. Children were treated for 52 weeks. The enema contained sodium‐dioctyl sulfosuccinate and sorbitol.

##### Efficacy

GRADE assessment showed that there may be no difference for treatment success between both groups (47% vs. 35%; RR: 1.33 [0.83–2.14]; low certainty). There was no measure of spread reported for defecation frequency, and therefore no analysis could be performed.

##### Safety

GRADE assessment showed that there may be no difference in withdrawals due to adverse events between both groups (8% vs. 0%; RR: 9.00 [0.50–162.97]; low certainty). Reasons were “refusal of further application” and were therefore counted as withdrawals due to adverse events. Total adverse events have not been reported in the study.

##### Risk of bias

Risk of bias was assessed as not serious for all GRADE assessments. Performance bias and detection bias were assessed as high risk. Risk of bias was not downgraded to serious because patients could not be blinded due to the nature of the intervention. Other domains, such as randomization and selective reporting, were low risk.

##### Rationale

A conditional recommendation was made against enemas as an add‐on to maintenance laxative treatment after evaluating the lack of evidence favoring enemas as an add‐on compared to treatment with PEG alone. No conclusions could be drawn for the use of enemas as a standalone maintenance treatment, and therefore, only a GRADE recommendation for the use of enemas as an add‐on treatment for maintenance therapy could be formulated. The long‐term use of enemas as an add‐on to maintenance treatment may not lead to more treatment success, and therefore, routine use of enemas as an add‐on therapy is not suggested. However, the GDG recognizes there is a therapeutic role for enemas for FC children, a GPS was formulated (see GPS below). It should always be considered that rectal therapies may be extra burdensome and invasive for a child with FC (see general statement regarding rectal therapies).

#### 4.2.2 Good practice statements

The GDG suggests that **lactulose has a therapeutic role** for FC in children.

Lactulose is an osmotic laxative. It is a synthetic disaccharide that is fermented in the colon by bacterial enzymes into low molecular weight acids. These acids create an osmotic effect, resulting in increased luminal fluid. In addition, the acids lower the fecal pH, which stimulates colonic motility.

##### Rationale

GRADE recommendations were voted on, but the level required for consensus level was not reached. The GDG acknowledges the lack of placebo‐controlled studies and the inferiority of lactulose compared to other laxatives with moderate certainty (PEG) and low certainty (liquid paraffin) evidence. However, the efficacy data from the lactulose groups in these studies demonstrated effect sizes for treatment success greater than the mean placebo rates observed (27% on network meta‐analysis). In addition, there is global experience with the use of lactulose, and it is considered safe from birth to 18 years old. Common reported adverse events of lactulose include abdominal pain, diarrhea, and bloating. Therefore, it is suggested that lactulose may be an appropriate alternative to PEG in cases of access, age, cost, tolerability, or acceptability issues.

The GDG suggests that **bisacodyl has a therapeutic role** for FC in children.

Bisacodyl is a stimulant laxative. It belongs to the diphenylmethane class and is hydrolyzed by colonic bacteria or brush‐border enzymes into its active metabolites, which stimulate colonic peristalsis and intestinal fluid secretion.

##### Rationale

Bisacodyl has not been studied in RCTs in the pediatric population and whilst high quality observational studies are also missing, significant cross‐sectional and retrospective series together with GDG experience support its safety and potential effectiveness.^69^ Reported side effects included abdominal pain and diarrhea.^69^ There was substantial GDG experience with its use and efficacy, as an addition to osmotic laxatives or as a stand‐alone treatment. Self‐limiting side effects have been seen that resolved upon discontinuation of the medication. Bisacodyl can be considered if PEG alone is not effective, either to replace PEG or as an adjunct therapy.

The GDG suggests that **senna has a therapeutic role** for FC in children.

Senna is a stimulant laxative. It contains anthraquinones, which are metabolized by intestinal bacteria. These active metabolites create the laxative effect by stimulating colonic motility and inhibiting the absorption of water from the colon.

##### Rationale

Senna has not been studied in RCTs and whilst high quality observational studies are also missing, significant cross‐sectional and retrospective series together with GDG experience support its safety and potential effectiveness.^70^ There was substantial GDG experience with its use and efficacy, as an addition to osmotic laxative or as a stand‐alone treatment. Self‐limiting side effects (e.g., perianal skin blisters in children who are diapered and not toilet trained and abdominal cramping) have been reported that resolved upon discontinuation of the medication.^70^ It should be noted that dietary supplements containing senna leaf extract are not comparable to therapeutic preparations for effectiveness and dosages. Senna can be considered if PEG alone is not effective, either to replace PEG or as an adjunct therapy.

The GDG suggests that **sodium picosulfate has a therapeutic role** for FC in children.

Sodium picosulfate is a stimulant laxative. It belongs to the diphenylmethane class and is hydrolyzed by colonic bacteria or brush‐border enzymes into its active metabolites, which stimulate colonic peristalsis and intestinal fluid secretion.

##### Rationale

Sodium picosulfate has only been investigated in one RCT with a very small sample size and has not been compared to a placebo. Whilst high‐quality observational studies are lacking, its use in other contexts (e.g., bowel preparation for surgery) has been investigated supporting its safety and potential efficacy.^22^ In addition, there was substantial experience among GDG members with efficacious use. Some self‐limiting side effects, such as diarrhea and abdominal pain have been reported that typically resolved upon discontinuation of the medication. Therefore, sodium picosulfate can be considered if PEG alone is not effective, either to replace PEG or as an adjunct therapy.

The GDG suggests that **liquid paraffin has a therapeutic role** for FC in children. However, **routine use is not recommended** due to the potential significant side effects.

Liquid paraffin, also known as mineral oil, is a lubricant. It is an orally administered oily liquid composed of hydrocarbons derived from petroleum. It is not absorbed by the intestines and thus acts as a lubricant for feces, producing its laxative effect.

##### Rationale

There is limited RCT data with low certainty evidence (two comparison studies with lactulose) for its effectiveness. Liquid paraffin is widely used, especially when other laxatives are not well tolerated or as an adjunct therapy in treatment‐resistant cases. However, sparse but significant case reports describe adverse events, including granulomas of the intestinal tract and exogenous lipoid pneumonia.^71^, ^72^, ^73^ This is especially prevalent in children younger than 3 years of age and children with swallowing disorders at risk for aspiration. Therefore, the routine use of this agent is not suggested.

The GDG **does not suggest the use of lubiprostone** as a treatment for FC in children.

Lubiprostone targets type 2 chloride channels on enterocytes, which leads to an increased fluid secretion in the intestine, which in turn leads to stool softening and increased peristalsis.

##### Rationale

There are two RCTs available investigating lubiprostone in children: one comparing lubiprostone to placebo and another to other laxatives.^74^, ^75^ These RCTs yielded contradictory results regarding the drug's efficacy. Due to this, and the limited experience of the majority of the GDG with the drug, the routine use of lubiprostone in the treatment of FC in children is not suggested. Both studies demonstrated the drug's safety with predominately self‐limiting side effects such as nausea and headache. The only treatment‐related serious adverse events (1% of all children) reported in the lubiprostone‐treated group were single cases of hypersensitivity reaction, rash, and chest pain.^76^

The GDG **does not suggest the use of domperidone** as a treatment for FC in children.

Domperidone is a dopamine 2 antagonist that does not cross the blood‐brain barrier. It increases esophageal peristalsis, lowers esophageal sphincter pressure, gastric motility, and peristalsis, but its physiologic effect in the colon appears limited.

##### Rationale

The effectiveness and safety of domperidone as a treatment option for FC are very uncertain. Domperidone has been studied in other pediatric gastrointestinal disorders (nausea and vomiting, gastroesophageal reflux, functional dyspepsia); however, evidence regarding its efficacy in the management of FC and safety is very limited.^77^ Due to the significant safety concern of possible QTc prolongation with domperidone and the lack of efficacy and safety data, domperidone is not suggested as a treatment option for children with FC.

General statement regarding rectal therapies: The GDG acknowledges the **potential psychosocial impact of all per rectum therapies.** The decision to consider such therapies should be shared with children and their families, taking key factors into account.

##### Rationale

When considering per rectum therapies, healthcare professionals should assess whether they are appropriate on an individual basis. This assessment should carefully consider key factors such as sensory differences of the child, a history of abuse in the parent and/or child, parental and/or child discomfort, as well the child's opposition and anxiety. The assessment should be sensitive to the developmental age of the child.

The GDG suggests **enemas have a maintenance therapeutic role** for FC in select children.

The mechanism of action of enemas for maintenance therapy is identical to that of enemas used for disimpaction and is described in the corresponding recommendation.

##### Rationale

The GDG recognizes that the GRADE recommendation did not cover all the possible applications of enemas in the treatment of FC. Due to the lack of more RCTs, a GRADE recommendation was not possible, and therefore an additional GPS was formulated. There was substantial GDG experience with the use of enemas as maintenance therapy in select cases with good efficacy. It should always be considered that rectal therapies may be burdensome for a child with FC or even cause pain and anxiety (see general statement regarding rectal therapies).

The GDG suggests rectal **suppositories have a therapeutic role** for FC in select cases, either as an adjunct or as an alternative when other therapies are not effective or appropriate.

Suppositories are solid or semi‐solid medications administered rectally that dissolve to release their active components, which produce a laxative effect depending on the type of active ingredient.

##### Rationale

Whilst there is a lack of evidence supporting the efficacy and safety of rectal suppositories in children with FC, there was substantial GDG experience with its efficacious use. It should also be noted that the use of rectal suppositories may cause self‐limited discomfort or pain. In addition, there is a range of rectal suppository preparations and active ingredients available, of which bisacodyl and glycerin are most commonly used. The use of those preparations approved by the local or national medicines regulatory agencies for this indication are suggested. The GDG suggests using rectal suppositories in select patients on an occasional basis rather than routinely (keeping in mind the psychosocial impact).

The GDG suggests **transanal irrigation has a therapeutic role** for FC in select cases (where other therapies are not effective or appropriate) and under specialized expert care.

Transanal irrigation is a procedure where fluids are flushed via a catheter or conus into the rectum to mechanically clean out the colon.

##### Rationale

Despite the lack of evidence, there was substantial GDG experience with the use of transanal irrigation as maintenance therapy in select cases, and efficacy had been experienced in these patients. The general statement regarding rectal therapies is also applicable for transanal irrigation. In addition, caution is advised regarding side effects with inappropriate use (e.g., water intoxication, risk of colonic perforation).^78^, ^79^ Patient evaluation and proper training of patients and their caregivers is critical for the safe practice of this treatment.

The GDG suggests **botulinum toxin A injections have a therapeutic role** for FC in select cases (refractory to conventional treatments) under specialized expert care.

Botulinum toxin A, also known as Botox, is injected in the anal sphincter. Botulinum toxins block the release of acetylcholine from nerve endings, leading to temporary chemical paralysis of the muscle and consequent relaxation of the anal sphincter muscles.

##### Rationale

Whilst there is limited RCT data for the use of botulinum toxin A injections in children with refractory constipation,^80^ all evidence was of very low certainty, and effectiveness remains uncertain. There was substantial GDG experience with the efficacious use of botulinum toxin injections in the anal sphincter complex in children, particularly young patients, who are refractory to conventional treatments. Botulinum toxin injections should be performed under specialized expert care following the local protocols and only has a therapeutic role in children with symptoms refractory to conventional treatments.^81^, ^82^, ^83^

#### 4.2.3 Dosing regimes used in research and clinical practice

Typical dosing regimens used in research and clinical practice to support understanding of the guideline's recommendations are summarized in Table 10. It is intended for contextual reference only and must not be used as a source of prescribing guidance. Actual prescribing should always follow current national formularies, locally approved drug lists, and regulatory authorities' guidance, which may differ by country due to variations in licensing status, availability of formulations, and evolving safety information.

**Table 10:** Typical dosing regimens used in research and clinical practice for the most frequently used oral and rectal laxatives.^a^ ^,^ ^16^, ^17^

Oral laxatives	Dosage
Osmotic laxatives	
PEG 3350/4000 (with or without electrolytes)	Fecal disimpaction: 1–1.5 g/kg/day in 1 or multiple divided doses (max 6 days) Maintenance: 0.4–0.8 g/kg/day in 1 or multiple divided doses
Magnesium hydroxide	2–5 years: 0.4–1.2 g/day, in 1 or multiple divided doses 6–11 years: 1.2–2.4 g/day, in 1 or multiple divided doses 12–18 years: 2.4–4.8 g/day, in 1 or multiple divided doses
Lactulose	Neonate: 350–700 mg/kg/day in 1 dose or in 2 divided doses 1–7 months: 350–700 mg/kg/day in 1 dose or in 2 divided doses 7–18 years: 0.6–2 g/kg/day in 1 dose or in 2 divided doses
Lactitol	1–6 years: 0.5‐1 g/kg/day in 2–3 divided doses 6–12 years: 10–30 g/day in 2–3 divided doses 12–18 years: 20–60 g/day in 2–3 divided doses
Stimulant laxatives	
Bisacodyl	3–10 years: 5 mg/day, in 1 dose/day 10–11 years: 5–10 mg/day, in 1 dose/day 12–18 years: 5–15 mg/day, in 1 dose/day
Senna	Syrup 8.8 mg sennosides/5 mL or tablets 8.6 mg sennosides/tablet 2–6 years: 2.5–3.75 mL/dose (4.4–6.6 mg sennosides), 1 or 2 doses/day 6–11 years: 5–7.5 mL/dose (8.8–13.2 mg sennosides) (or 1–2 tablets), 1 or 2 doses/day 12–18 years: 5–15 mL/dose (17.6–26.4 mg sennosides) (or 1–3 tablets), 1 or 2 doses/day
Sodium picosulfate	<4 years: 0.25 mg/kg/day 4–10 years: 2.5–5 mg once/day, in 1 dose/day >10 years: 5–10 mg/day, in 1 dose/day
Prosecretory laxatives	
Linaclotide	6–17 years: 72 μg in 1 dose/day
Rectal laxatives/retrograde enemas	Dosage
Bisacodyl	2–11 years: 5 mg/day, in 1 dose >11 years: 5–10 mg/day, in 1 dose
Sodium lauryl sulfoacetate + sorbitol	1 month–1 years: 2.5 mL/dose, once/day (=0.5 enema) 1–3 years: 2.5–5 mL/dose, once/day (=0.5–1 enema) 3–18 years: 5 mL/dose, once/day (=1 enema)
Sodium docusate	1–6 years: 30–60 mg/dose, once/day 6–12 years: 50–120 mg/dose, once/day 12–18 years: 120 mg/dose, once/day
Sodium phosphate	2–4 years: 29 mL, in 1 dose/day (=0.5 pediatric enema) 5–11 years: 59 mL, in 1 dose/day (=1 pediatric enema) 12–18 years: 118 mL, in 1 dose/day (=1 adult enema)
Sodium chloride (NaCl)	Neonate <1 kg: 5 mL/dose, once/day Neonate >1 kg: 10 mL/dose, once/day >1 year: 6 mL/kg in 1 or 2 doses/day
Lubricants	Dosage
Liquid paraffin (mineral oil)	Oral: 3–18 years: 1–3 mL/kg/day, in 1 or multiple divided doses (max 90 mL/day) Rectal: 2–11 years: 30–60 mL, in 1 dose/day >11 years: 60–150 mL, in 1 dose/day

Abbreviation: PEG, polyethylene glycol.

^a^ This summary table of typical dosing regimens used in research and clinical practice is provided to support understanding of the guideline's recommendations. It is intended for contextual reference only and must not be used as a source of prescribing guidance. Actual prescribing should always follow current national formularies, locally approved drug lists, and regulatory authorities’ guidance, which may differ by country due to variations in licensing status, availability of formulations, and evolving safety information.

### 4.3 Nonpharmacological maintenance treatment

#### 4.3.1 GRADE recommendations

*Should **pro‐ and synbiotics** be used as a treatment option for FC?*

Recommendations:

a. A specific probiotic mixture (containing *Lactobacillus acidophilus, Bifidobacterium longum, and Streptococcus thermophilus*) **is suggested** as a treatment option for FC in children. **Conditional** recommendation, overall **low** certainty evidence, effect size **large**
b. A specific synbiotic preparation (multi‐strain probiotics and prebiotics*) **is suggested** as a treatment option for FC in children

*Lactobacillus casei, Lactobacillus rhamnosus, Lactobacillus plantarum, and Bifidobacterium lactis and prebiotics (fiber, polydextrose, fructo‐oligosaccharides, and galacto‐oligosaccharides)

**Conditional** recommendation, overall **low** certainty evidence, effect size **large** (Tables 11 and 12).

**Table 11:** Data summary for probiotics per strain.

Conclusions of the evidence for probiotic mixture: *L*. *acidophilus*, *B*. *longum*, *and S*. *thermophilus* a	GRADE certainty	Descriptive effect size absolute effect mean (CI 95%)	Cates plot
Probiotic mixture versus placebo			
A probiotic mixture containing *L. acidophilus, B. longum, and S. thermophilus* may lead to more treatment success	Low	Large in favor (small to large in favor) 33% more (8% more to 69% more)	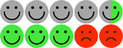
There may be no difference in defecation frequency per week	Low	Trivial in favor (trivial against to small in favor) MD: 0.30 higher (0.64 lower to 1.24 higher)	NA

Abbreviations: CI, confidence interval; GRADE, grading of recommendations assessment, development and evaluation; MD, mean difference; NA, not applicable.

^a^ *Lactobacillus acidophilus*, *Bifidobacterium longum*, and *Streptococcus thermophilus*.

**Table 12:** Data summary for synbiotics per strain.

Conclusions of the evidence for synbiotic preparation: *L*. *casei*, *L*. *rhamnosus*, *L*. *plantarum*, *and B. lactis* a and prebiotics *(fiber*, *polydextrose*, *fructo‐oligosaccharides*, *and galacto‐oligosaccharides)*	GRADE certainty	Descriptive effect size Absolute effect mean (CI 95%)	Cates plot
Synbiotic preparation versus placebo			
This specific synbiotic preparation may lead to more treatment success	Low	Large in favor (moderate to large in favor) 36% more (15% more to 67% more)	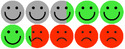

Abbreviations: CI, confidence interval; GRADE, grading of recommendations assessment, development and evaluation.

^a^ *Lactobacillus casei*, *Lactobacillus rhamnosus*, *Lactobacillus plantarum*, and *Bifidobacterium lactis.*

Probiotics are defined as “live microorganisms which when administered in adequate amounts confer a health benefit on the host.”^84^ It is proposed that an imbalance in the gut microbiota may affect colonic motility or peristaltic activity, potentially contributing to the development of constipation. Probiotics produce acids that lower the pH of the colon, which in turn may enhance colonic peristalsis. Prebiotics are defined as “a substrate that is selectively utilized by host microorganisms conferring a health benefit” and act as nutrients that support the growth of beneficial microorganisms, including certain probiotic strains.^85^ Synbiotics are preparations containing both probiotics and prebiotics.

##### Summary of evidence

Twenty RCTs investigating different probiotic strains were included. Eight studies compared one probiotic strain or a mixture of probiotic strains to a placebo (*n* = 572, age 0–18 years).^58^, ^86^, ^87^, ^88^, ^89^, ^90^, ^91^, ^92^ Three of these studies investigated a mixture of different probiotics.^87^, ^88^, ^91^ The remaining studies investigated specific strains, including *Bacillus clausii,* ^86^ *Lactobacillus reuteri,* ^89^, ^92^ and *L. casei rhamnosus*.^58^, ^90^ Three studies compared different probiotic strains to laxatives (*n* = 208, age 0–16 years).^58^, ^93^, ^94^ One study compared *L. casei rhamnosus* lcr35 to magnesium oxide.^58^ One compared *Saccharomyces boulardii* to lactulose.^93^ One study compared *L. reuteri* to lactulose.^94^ Nine studies compared the addition of probiotics to laxatives with laxatives only (*n* = 838, age 6 months–16 years).^42^, ^59^, ^93^, ^95^, ^96^, ^97^, ^98^, ^99^, ^100^ Four of those studies investigated multispecies probiotics.^42^, ^95^, ^98^, ^100^ Three studies investigated *L. reuteri* ^59^, ^97^, ^99^ and one study investigated *L. rhamnosus GG*.^96^ Investigated laxatives were PEG, lactulose, and magnesium oxide.

One study compared the addition of probiotics to a goat yoghurt (*n* = 60, age 5–15 years).^101^ One study compared two types of formulas (*n* = 95, age 12–32 months), in which the test formula contained a probiotic (*B. lactis* HN019).^102^

Three RCTs investigating different synbiotic preparations were included.^42^, ^103^, ^104^ One study compared a synbiotic preparation containing a probiotic mixture (*L. casei, L. rhamnosus, L. plantarum, and B. lactis)* and prebiotics *(fiber, polydextrose, fructo‐oligosaccharides, and galacto‐oligosaccharides)* to a placebo (*N* = 155, 4–18 years).^103^ One study compared the synbiotic preparation containing a probiotic mixture (*L. casei, L. rhamnosus, S. thermophilus, Bifidobacterium breve, L. acidophilus, Bifidobacterium infantis)* and *fructooligosaccharide* as prebiotic to liquid paraffin (*n* = 97, age 4–12 years).^104^ One study compared the synbiotic preparation containing a probiotic mixture (*L. reuteri*, *L. rhamnosus*, and *B. infantis*) and *psyllium seed husk powder* as a prebiotic to PEG (*n* = 144, age 2–12 years).^42^

##### Efficacy

When probiotic studies were subgrouped by bacterial strain, one preparation, investigated in one study, showed a significant effect favoring probiotics compared to placebo (low certainty evidence).^88^ The study compared a probiotic mixture containing *L. acidophilus, B. longum, and S. thermophilus* to placebo and reported that it may lead to more treatment success (79% vs. 46%; RR 1.72 [1.18–2.50]; large magnitude of effect; low certainty) but may not lead to a difference in defecation frequency (MD: 0.61 [–0.73, 1.95]; low certainty). All other preparations were of very low certainty when analyzed at a strain level or did not show effect and are therefore not further discussed in this section. See Supporting Information: File S2 for all analyses. When studied per probiotic strain, a multi‐strain synbiotic may lead to more treatment success compared to placebo (62% vs. 27%; RR 2.32 [1.54–3.47]; large magnitude of effect, low certainty).^103^ A different multi‐strain synbiotic may not lead to a difference in defecation frequency as addition to liquid paraffin compared to liquid paraffin alone (MD 0.02 [–0.56–0.60], low certainty).^104^

When all probiotic strains were pooled in a meta‐analysis, no conclusions could be drawn for probiotics compared to a placebo. Pooled meta‐analysis showed that probiotics may lead to higher defecation frequency per week compared to laxatives, however the difference was trivial (MD: 0.36 [0.15–0.57]; trivial magnitude of effect; low certainty). Probiotics were compared to lactulose and magnesium oxide for this meta‐analysis. Pooled meta‐analysis showed that the addition of probiotics to laxatives compared to laxatives alone may not lead to a difference in defecation frequency per week (MD: 0.12 [–0.09–0.34]; low certainty). Investigated laxatives consisted of lactulose and PEG.

##### Safety

No conclusions could be drawn for withdrawals due to adverse events due to very low certainty evidence, predominantly caused by very serious imprecision. Meta‐analysis showed there was no difference in adverse events for probiotics compared to placebo (4% vs. 8%; RR: 0.59 [0.24–1.44]; low certainty). No serious adverse events related to the interventions were reported in the included trials. Adverse events were not reported for the majority of included studies. Abdominal pain was the most commonly described adverse event, both in the intervention and control groups.

##### Risk of bias

For the comparison of probiotics to placebo, all primary outcomes were downgraded once for risk of bias. This was caused by some trials in which selective reporting and allocation concealment was unclear and one study with high risk of performance and detection bias. For all other comparisons outcomes were downgraded twice for risk of bias. Common reasons included lack of blinding in some trials, unclear allocation concealment and unclear selective reporting due to inadequate reporting or lack of information.

##### Rationale

A conditional recommendation was made for the specific probiotic mixture containing *L. acidophilus, B. longum, and S. thermophilus,* and a specific multi‐strain synbiotic preparation after evaluating the benefits and harms of the treatment. These specific multi‐strain pro‐ and synbiotic preparations may lead to more treatment success compared to placebo. The recommendation is based on a single study, with low certainty evidence.^88^, ^103^ No safety issues were reported in the included trial. Probiotic strains are generally considered safe in otherwise healthy children and are often tried by patients on empiric basis for many different conditions (e.g. gastrointestinal disorders, inflammatory disorders and allergies).^105^ Therefore, conditional GRADE recommendations were made about these specific pro‐ and synbiotic preparations, but other preparations do not have sufficient evidence at a strain level to support such recommendations.

*Should a **cow's milk‐free diet** be used as a treatment option for FC?*

Recommendation: A cow's milk‐free diet trial for 2–4 weeks **is suggested** as an adjunct treatment option for FC in children not responding to conventional therapy alone and when cow's milk allergy (CMA) is suspected.

**Conditional** recommendation, overall **low** certainty evidence, effect size **moderate** (Table 13).

**Table 13:** Data summary for cow's milk free diet.

Conclusions of the evidence for CMFD	GRADE certainty	Descriptive effect size absolute effect mean (CI 95%)	Cates plot
CMFD + PEG versus CMD + PEG			
A cow's milk‐free diet as an addition to PEG may lead to more children with higher defecation frequency	Low	Moderate in favor (small to large in favor) 24% more (11% more to 41% more)	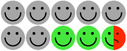

Abbreviations: CI, confidence interval; CMD, cow's milk diet; CMFD, cow's milk free diet; GRADE, grading of recommendations assessment, development and evaluation; PEG, polyethylene glycol.

CMA is a reaction to cow's milk that involves immunological mechanisms.^106^ While constipation can be one of the symptoms associated with CMA, it is rarely the primary cause of constipation, and constipation will never be the only symptom of CMA.^106^ CMA should only be diagnosed based on a complete history and physical examination.^106^ The pathophysiology of CMA‐related constipation remains unclear. It is hypothesized that pain‐related withholding and incomplete anal sphincter relaxation due to immune‐mediated inflammation affecting the rectal mucosa may play a role in the pathophysiology.^106^, ^107^, ^108^

##### Summary of evidence

Four RCTs investigating a cow's milk‐free diet were included. One study compared cow's milk free diet to cow's milk diet (*n* = 64, 0–6 years).^109^ Two studies compared the combination of a cow's milk‐free diet with PEG to a diet without restrictions on cow milk and treatment with PEG (*n* = 211, age 0–14 years).^110^, ^111^ One study compared two types of formula, one formula containing hydrolyzed protein and prebiotics and the other containing cow's milk and prebiotics (*n* = 100, age 28–300 days).^112^

##### Efficacy

GRADE assessment showed the addition of a cow's milk‐free diet to PEG may lead to more children with three or more bowel movements per week (96% vs. 71%; RR: 1.34 [1.15–1.57]; NNT 4 [2–9]; moderate magnitude of effect; low certainty) compared to a cow's milk diet. No conclusions could be drawn for treatment success for this comparison due to heterogeneity and imprecision, however both studies included in the meta‐analysis showed significantly more patients reached treatment success with a cow's milk‐free diet.^110^, ^111^

No conclusions could be drawn for the comparison of the two types of formulas due to very low certainty evidence.

##### Safety

No conclusions could be drawn for safety outcomes due to very low certainty. Adverse events were reported in only one study, 14/42 children experienced an adverse event in the hydrolyzed whey formula group and 8/47 in the cow's milk group. The types of adverse events were not reported. No serious adverse events were reported.

##### Risk of bias

All efficacy and safety outcomes were downgraded twice for risk of bias. Common reasons were unclear allocation concealment and unclear selective reporting, resulting from inadequate reporting and lack of trial registration data. In addition, the majority of the studies had an open‐label design, which was inherent to the intervention.

##### Rationale

A conditional recommendation was made for a 2–4 week trial of a cow's milk‐free diet for children who do not respond to conventional therapy alone. See the algorithm (Figure 1) for when a cow's milk‐free diet may be considered in the course of treatment. A Cow's milk‐free diet may lead to higher defecation frequency as an add‐on to laxatives. If the trial is successful, further management should follow the ESPGHAN/NASPGHAN CMA guideline.^106^ Dietary treatment options can seem harmless and therefore receive consideration as an entry‐level treatment option, particularly for motivated families. It is important to emphasize that restrictive diets may require unrealistic or even disproportionate commitment from children and should be employed with the same consideration as all active interventions, with particular caution in children with risk factors for disordered eating or malnutrition. The statement from the ESPGHAN/NASPGHAN CMA guideline is therefore highlighted: “Professional dietary counseling by a dietitian should be offered to children on a cow's milk elimination diet to prevent malnutrition and promote a varied diet leading to normal feeding behavior.” Supplementation with calcium was used in trials and should be considered during a cow's milk‐free diet.^110^

*Should **pelvic floor physiotherapy** be used as a treatment option for FC?*

Recommendation: Pelvic floor physiotherapy **is suggested** as an adjunct treatment option for FC in children.

**Conditional** recommendation, overall **low** certainty evidence, effect size **large** (Table 14).

**Table 14:** Data summary for pelvic floor physiotherapy.

Conclusions of the evidence for pelvic floor physiotherapy	GRADE certainty	Descriptive effect size absolute effect mean (CI 95%)	Cates plot
Pelvic floor physiotherapy + standard medical care versus standard medical care			
Pelvic floor physiotherapy as an addition to standard medical care may lead to more treatment success	Low	Large in favor (moderate to large in favor) 48% more (15% more to 99% more)	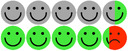

Abbreviations: CI, confidence interval; GRADE, grading of recommendations assessment, development and evaluation.

Pelvic floor physiotherapy assumes that FC can be partly caused by incorrect use of the pelvic floor and abdominal muscles (dyssynergic defecation), as well as the interaction between breathing, posture, and movement. However, proving that there is an abnormality is not required for referral, and may not be predictive of successful therapy. Treatment by a qualified pediatric pelvic floor physiotherapist will include providing information and general advice, along with toilet training, relaxation exercises, breathing exercises, and defecation‐promoting exercises on the toilet. The exercises aim to improve the function of the diaphragm, abdominal, and pelvic floor muscles, teach the correct straining and relaxation technique and toilet posture, and enhance body awareness, helping the child recognize the urge to defecate and respond appropriately.

##### Summary of evidence

Three RCTs investigating physiotherapy or manual physical therapy were included. One study compared pelvic physiotherapy as an adjunct to standard medical care to standard medical care alone (*n* = 53, age 5–15 years).^113^ Standard medical care consisted of education, demystification, dietary advice, toilet training, keeping track of bladder and bowel diaries, and the addition of PEG when needed. PEG was prescribed to 52 of 53 children (98.1%). One study compared manual physical therapy to treatment with PEG (*n* = 47, age 2–14 years).^51^ One study compared abdominal muscle training/breathing exercises/abdominal massage as an adjunct to magnesium hydroxide to magnesium hydroxide alone (*n* = 72, age 4–18 years).^114^

##### Efficacy

GRADE assessment showed pelvic physiotherapy as an addition to standard medical care may lead to more treatment success compared to standard medical care alone (92% vs. 44%; RR: 2.08 [1.34–3.21]; NNT 2 [1–6]; large magnitude of effect; low certainty). Defecation frequency was reported for only a subset of the randomized patients and could therefore not be analyzed.

##### Safety

Adverse events were not reported in the included trials. No conclusions could be drawn for withdrawals due to adverse events due very low certainty evidence as a result of a lack of events or limited number of events.

##### Risk of bias

One comparison was only downgraded once for risk of bias due to unclear allocation concealment.^113^ For the two other comparisons the outcomes were downgraded twice for risk of bias due to unclear risk of bias across the majority of the domains. All included trials were open‐label due to the nature of the intervention.

##### Rationale

A conditional recommendation was made for pelvic floor physiotherapy as adjunct therapy after evaluating the benefits and harms of the treatment. GRADE assessments showed pelvic floor physiotherapy as an adjunct to standard medical care may lead to more treatment success. The recommendation could only be based on one study, including a small number of children. No conclusions could be drawn for pelvic floor physiotherapy as a stand‐alone treatment, and therefore, the use of pelvic floor physiotherapy can only be suggested as adjunct therapy. The GDG suggests referring children with suspected dyssynergic defecation and with a developmental age of 6 years or older to a pelvic floor physiotherapist specialized in children.

*Should **abdominal transcutaneous electrical stimulation (TES)** be used as a treatment option for FC?*

Recommendation: Abdominal TES in combination with pelvic floor muscle exercises (PFME) **is suggested** as a treatment option for FC in children.

**Conditional** recommendation, overall **moderate** certainty evidence, effect size **moderate** (Table 15).

**Table 15:** Data summary for abdominal TES.

Conclusions of the evidence for ATES	GRADE certainty	Descriptive effect size absolute effect mean (CI 95%)	Cates plot
ATES + PFME versus PFME			
ATES as an addition to PFME probably leads to more treatment success	Moderate	Large in favor (small to large in favor) 30% more (10% more to 58% more)	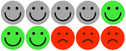
ATES as an addition to PFME probably leads to higher defecation frequency per week	Moderate	Small in favor (small to moderate in favor) MD: 1.85 higher (1.28 higher to 2.43 higher)	NA

Abbreviations: ATES, abdominal transcutaneous electrical stimulation; CI, confidence interval; GRADE, grading of recommendations assessment, development and evaluation; MD, mean difference; NA, not applicable; PFME, pelvic floor muscle exercises; TES, transcutaneous electrical stimulation.

For abdominal TES, two electrode pads are placed on the lower abdomen and two on the lower back, producing a low‐frequency electrical current.^115^ The exact mechanism of electrical stimulation is not yet fully understood, but it likely involves multiple processes that improve gastrointestinal motility, such as modulating the enteric nerves and stimulating the interstitial cells of Cajal, the “pacemaker” cells that regulate peristaltic activity.^116^, ^117^

##### Summary of evidence

Four RCTs investigating abdominal TES were included. One study compared abdominal TES to sham therapy (*N* = 33, age 7–18 years).^118^ Two studies compared abdominal TES as an addition to PFME with PFME alone (*N* = 124, age 5–13 years).^119^, ^120^ In one of these studies, patients in the control group also received sham therapy.^120^ Another study compared abdominal TES as an addition to standard therapy with standard therapy alone (*N* = 40, age 3–15 years).^115^ Standard therapy consisted of a laxative diet, probiotics, choleretic drugs, and enzymes. Generally, treatment with TES consisted of 2–3 sessions per week for 4–5 weeks. One trial treated patients daily for 6–10 min for 10 consecutive days.^115^

##### Efficacy

One study^118^ did not report any of the predefined efficacy outcomes and for one study no conclusions could be drawn. Meta‐analysis and GRADE assessment showed abdominal TES as addition to PFME probably leads to more treatment success (73% vs. 40%; RR: 1.75 [1.25–2.44]; NNT: 3 [2–10]; large magnitude of effect; moderate certainty) and to higher defecation frequency per week (MD: 1.85 [1.28–2.43]; small magnitude of effect; moderate certainty).

##### Safety

No conclusion could be drawn for the safety outcomes. Safety outcomes were only reported in two studies, and no adverse events occurred during the trials.^119^, ^120^

##### Risk of bias

The primary outcomes for abdominal TES as an addition to PFME compared to PFME alone were downgraded once for risk of bias due to unclear allocation concealment and unclear risk of selective reporting in one study.^119^ Due to the nature of the intervention, the studies could not be blinded. Other domains were low risk of bias. One study was assessed as a very serious risk of bias, due to unclear risk of bias in the majority of the domains.^115^

##### Rationale

A conditional recommendation was made for abdominal TES in combination with PFME after evaluating the benefits and harms of the treatment. Meta‐analyses showed abdominal TES in combination with PFME probably leads to more treatment success and higher defecation frequency. Evidence on abdominal TES without PFME was of very low certainty, and no conclusions could be drawn. Therefore, the use of abdominal TES can only be suggested in combination with PFME. Abdominal TES is a noninvasive treatment, and no safety issues have been raised in the included trials or in other literature. Abdominal TES in combination with PFME can be considered if pharmacological treatment alone has not been effective.

*Should **percutaneous tibial nerve stimulation (PTNS)** be used as a treatment option for FC?*

Recommendation: PTNS in combination with pelvic floor muscle exercises (PFME) **is suggested** as a treatment option for FC in children.

**Conditional** recommendation, overall **moderate** certainty evidence, effect size **small** (Table 16).

**Table 16:** Data summary for PTNS.

Conclusions of the evidence for PTNS	GRADE certainty	Descriptive effect size absolute effect mean (CI 95%)	Cates plot
PTNS + PFME versus PFME			
PTNS as an addition to PFME may lead to more treatment success	Low	Large in favor (trivial to large in favor) 26% more (3% more to 63% more)	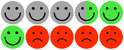
PTNS as an addition to PFME probably leads to higher defecation frequency per week	Moderate	Small in favor (trivial to moderate in favor) MD: 1.82 higher (0.82 higher to 2.82 higher)	NA
There may be no difference in withdrawals due to adverse events	Low	No effect (large in favor to large against) 0% (6% less to 26% more)	NA

Abbreviations: CI, confidence interval; GRADE, grading of recommendations assessment, development and evaluation; MD, mean difference; NA, not applicable; PFME, pelvic floor muscle exercises; PTNS, percutaneous tibial nerve stimulation.

PTNS is a minimally invasive neuromodulation technique used to treat various pelvic floor and bladder disorders.^117^, ^121^ A fine needle electrode is inserted near the medial malleolus. Low‐frequency electrical impulses are delivered via the needle electrode to stimulate the posterior tibial nerve, which indirectly stimulates the sacral nerves, regulating pelvic floor, bladder, and bowel function. It is also possible to stimulate the tibial nerve with electrode pads instead of a needle, which would make the treatment noninvasive, however this has not been studied in the included trials.^116^, ^122^

##### Summary of the evidence

One study investigating PTNS in combination with pelvic floor exercises (PFE) was included (*n* = 82, age 4–14 years).^123^ PTNS with PFE was delivered twice daily for 4 weeks. The control group received sham PTNS and PFE twice daily for 4 weeks. PFE was performed using an electromyography biofeedback method, in which an electrode is inserted through the anus.

##### Efficacy

GRADE assessments showed PTNS in combination with PFE may lead to more treatment success (62% vs. 36%; RR: 1.73 [1.08–2.77]; NNT: 4 [2–35]; large magnitude of effect; low certainty) and probably to higher defecation frequency per week (MD: 1.82 [0.82–2.82]; small magnitude of effect; moderate certainty).

##### Safety

GRADE assessment showed there may be no difference in withdrawals due to adverse events (7% vs 7%; RR: 1.00 [0.21–4.67]; low certainty) and no difference in total adverse events (7% vs 10%; RR: 0.75 [0.18–3.15]; low certainty). Reported adverse events included skin allergies, erythema, blisters, and foot numbness. No serious adverse events have been reported.

##### Risk of bias

Risk of bias was assessed as not serious, as all domains were assessed as low risk of bias, except for performance bias. Due to the nature of the intervention the treating nurse could not be blinded for the intervention.

##### Rationale

A conditional recommendation was made for PTNS in combination with PFE after evaluating the benefits and harms of the treatment. GRADE assessments showed PTNS in combination with PFE may lead to more treatment success and probably to higher defecation frequency per week. No studies investigating PTNS as a stand‐alone treatment have been performed, and therefore, the use of PTNS can only be suggested in combination with PFE. PTNS is a minimally invasive treatment, and no safety issues have been raised in the included trial or in other literature.^123^, ^124^, ^125^ PTNS in combination with PFE can be considered if pharmacological treatment alone has not been effective.

#### 4.3.2 Good practice statements

The GDG r**ecommends demystification, explanation, and guidance for toilet training** (if developmentally appropriate) in the treatment of FC in children.

##### Rationale

There is no evidence on the effectiveness of education or toilet training for the treatment of FC in children. However, it is vital to counsel children and their caregivers, explain the benign nature of the disorder, and educate them about the role of withholding behavior and the concept of overflow incontinence. It is recommended to implement a toilet training program with a reward system, encouraging the child to attempt defecation after meals.

General statement regarding diet and physical activity: The GDG **recommends a healthy, balanced diet with age‐appropriate water and fiber intake and normal physical activity** in children with FC.

##### Rationale

There is no evidence to support physical activity, fluid, or fiber intake in the treatment of FC in children beyond the recommended daily amounts. An essential initial step in evaluating a child with FC is to determine whether they consume a balanced diet with age‐appropriate amounts of both soluble and insoluble fibers, maintain sufficient fluid intake, and engage in normal daily physical activity. If these are suboptimal, this should be addressed following the recommended national guidelines for an age‐appropriate diet and physical activity before initiating other treatments.

The GDG has made a GRADE recommendation about specific preparations of probiotics and synbiotics. **Other pro‐ and synbiotic preparations do not have sufficient evidence** at a strain level to support such recommendations due to issues with precision of the data and methods of the studies.

##### Rationale

Many probiotic preparations are readily available without a prescription. However, outside of the specific probiotic mixture (*L. acidophilus, B. longum, and S. thermophilus*) and specific synbiotic preparation that had low certainty evidence for one of the critical efficacy outcomes, evidence was inconsistent, limiting an overall class recommendation. Whilst individual outcomes of individual strains did show an effect, which may be the reason for interest in these agents, further research must address the issues with adequate sample size and methods.

The GDG suggests that **behavioral interventions should not be used in isolation** but employed in select patients as an adjunct to other pharmacological therapies.

Behavioral interventions encompass a range of approaches that are tailored to the child's specific behavioral problems and aim to modify behaviors and habits that contribute to symptom persistence.

##### Rationale

There was only one RCT available showing low certainty evidence that behavioral interventions have no effect in the treatment of FC in children. However, in this study, all children with FC referred by general practitioners, school doctors, and pediatricians to a tertiary gastrointestinal outpatient clinic received behavioral therapy. Behavioral interventions as an adjunct to pharmacological therapies may be beneficial for select children. When identifying candidates for behavioral interventions, it is key to consider the developmental age of the child and the individual characteristics, such as neurodevelopmental or behavioral issues. The GDG suggests referring children with constipation and behavioral problems to a child psychologist with expertise in FC if there is poor adherence to the medical regimen, significant child/family stress inhibiting progress, and/or concern about comorbid behavioral diagnoses (e.g., phobias, anxiety disorders, or attention deficit hyperactivity disorder). It is important to highlight that children with FC who are on laxative therapy should be co‐managed by the physician and psychologist for optimum results.

The GDG suggests **not to use biofeedback alone**, but to consider it as part of a wider strategy of pelvic floor physiotherapy.

Biofeedback aims to correct dyssynergic defecation by teaching children to recognize the urge to defecate and to appropriately relax and contract the pelvic floor muscles using anorectal monitoring instruments, thereby facilitating more effective and less painful stool passage.

##### Rationale

Available evidence on biofeedback was of very low certainty, therefore no GRADE recommendations could be made. There was a GDG experience with the use of biofeedback, and the GDG acknowledges biofeedback may be useful as part of a wider strategy of pelvic floor physiotherapy in select patients. There is no role for biofeedback as a standalone treatment.

### 4.4 Surgical therapy

#### 4.4.1 GRADE recommendations

Based on the available evidence, no GRADE recommendations could be made.

#### 4.4.2 Good practice statements

The GDG **recognizes the role of surgical interventions in a very select group of patients** who are refractory to medical therapy. Prior to surgery, it is vital to ensure that the range of other treatments in this guideline have been carefully considered, a multi‐disciplinary team is involved, risks are weighed, appropriate investigations have been conducted, and that postsurgical prognosis is discussed with the child and family.

##### Rationale

There was no RCT data available on surgical interventions for the treatment of FC in children. However, surgical interventions may have a therapeutic role in a very select group of children with persistent constipation who are refractory to all other therapies when appropriately applied, and whose quality of life is severely impacted. Surgical interventions may include antegrade continence enema (ACE), sigmoidectomy, colostomy, ileostomy, subtotal colectomy, or colectomy.^19^ Before considering a surgical intervention, it is vital that the patient is appropriately investigated. Typically, the least invasive surgical treatment procedure (e.g., ACE) is considered as the first option, however this should be assessed on an individual basis and based on the underlying etiology and results of investigations. There was limited expertise within the GDG to make specific recommendations for these individual surgical options for this guideline.

### 4.5 Proposed not to make a recommendation

‐ Herbal medicine
‐ Cryotherapy
‐ Massage
‐ Dry cupping
‐ The Uniformed Services Constipation Action Plan
‐ Sacral nerve stimulation

### 4.6 No consensus

*Parasacral transcutaneous electrical nerve stimulation:* the GDG could not reach consensus after voting twice on a GRADE recommendation, and experience amongst the GDG was insufficient, limiting a GPS.

## 5 FUTURE RESEARCH GOOD PRACTICE STATEMENT

Systematic review of the evidence has exposed major evidence gaps for commonly used treatments for pediatric FC and consistent methodological deficiencies within the included studies. This is in line with the findings and future research directions described in the ESPGHAN/NASPGHAN functional abdominal pain disorder treatment guideline for children.^9^ Detailed reporting of other patient‐related factors within both intervention and placebo/control groups is needed. Categories such as disease duration at baseline, previous therapies tried, severity of symptoms at baseline, such as withholding behavior, and level of care expertise accessed previously all appear to be variable across studies. Whatever the answer to such questions are, it is vital that these are transparently reported in detail to aid better interpretation.

Similarly, reporting of broader trial protocol‐based interventional elements was also poor. Factors such as the nature and frequency of clinical assessments, education content, behavioral interventions, dietary guidance offered, prior received therapeutics, and prognostic information discussed should also be transparently described. These elements clearly varied across studies and may have contributed to the high placebo response rate, due to the nonorganic nature of FC. This is equally seen in the lack of detailed reporting of highly relevant subgroup patient characteristics. Factors such as gender, ethnic origin, or age are often reported in baseline characteristics but rarely used within analyses.

These items form a portfolio of information that are both vital for the detailed interpretation and application of study findings, as well as being inconsistently reported within studies, if reported at all. Achieving an international consensus on a standardized framework for such vital information will support consistent reporting of these critical details across this and other similarly documented functional bowel disorders.

Methodological limitations within the randomized trials significantly hinder the ability to synthesize data from multiple studies and to establish GRADE recommendations. Therefore, methodological guidance on the following key areas are proposed:

‐ Primary researchers should use internationally agreed‐upon, predefined definitions for FC, therapy‐resistant constipation, and fecal impaction when selecting study participants. For FC, this entails applying the Rome IV criteria. However, international consensus on definitions for therapy‐resistant constipation and fecal impaction is currently lacking.^18^, ^20^ In the absence of consensus, the GDG recommends using the definitions proposed in this treatment guideline.
‐ Ensure trial reporting is fully aligned with international trial reporting guidelines, with particular emphasis on core elements relevant to risk of bias assessment.^126^, ^127^, ^128^ Examples include providing comprehensive detail on randomization and allocation concealment methods, blinding methods for patients, study personnel and outcome assessors, details on study dropouts per study group and reasons why they dropped out, links to prospectively published trial registrations or protocols, as well as clear and appropriate presentation of the raw study results (numbers of events and means with measures of variance) for all study groups, instead of solely the results of statistical tests and *p*‐values.

Primary researchers should consider the previously published core outcome set and the critical (primary) outcomes identified by consensus within this guideline to ensure the most appropriate outcomes are reported, not just for the individual trial, but also to facilitate wider synthesis of the entire evidence base.^11^

‐ Sample size calculations should account for the comparison of two active treatments,^129^, ^130^ often involving PEG as the control group as an established effective treatment. Outcome thresholds should be accounted for, guided by the thresholds outlined in this publication, to ensure more appropriate and potentially larger sample sizes that will improve of outcomes.^131^
‐ PEG should be considered the comparative treatment rather than a placebo, given the clear certainty and magnitude of data supporting PEG. Primary researchers should consider comparing the therapy of interest as addition to standard pharmacological therapy (PEG) instead of performing direct comparisons between two active interventions as this is more clinically relevant.^132^

There is a significant lack of RCTs evaluating commonly used treatments, such as stimulant laxatives.^132^, ^133^ Future research should focus on assessing the efficacy and safety of these laxatives as adjuncts to PEG. Novel therapies, such as tenapanor (sodium/hydrogen exchanger isoform 3 inhibitor), mizaglifazon (sodium‐glucosecotransporter 1 inhibitor), and elobixibat (ileal bile acid transporter inhibitor) are currently being investigated in adult trials and have shown promising results in patients with chronic constipation and irritable bowel syndrome with constipation, representing potential new pediatric research.^134^ Efficacy demonstrated in adults cannot be directly translated to children, given the differences in pathophysiology and pharmacokinetics between children and adults. In addition, diagnostic criteria and primary outcome measures used in adult trials often differ from those used in pediatric studies, further limiting comparability. For example, pediatric trials on prucalopride and lubiprostone included in these guidelines have reported results that are not consistent with findings from adult studies.^64^, ^74^, ^75^, ^76^, ^135^ Several pediatric studies evaluating the safety and efficacy of tenapanor are currently ongoing (NCT05905926, NCT06553547, NCT05643534), whereas no pediatric studies are currently registered for mizagliflozin or elobixibat. The use of network meta‐analysis may support better comparisons, but this is currently limited by many of the issues above. If these improve, so too will the scope for such analysis to support future understanding, as has been seen in functional abdominal pain, for example.^136^

### 5.1 Future guideline updates

The GDG acknowledges the recommendations made in the ESPGHAN/NASPGHAN FAPD treatment guidelines regarding future guideline updates and suggests that, as a minimum, an update is commissioned within 3 years to allow completion and publication of a new guideline within 5 years.^9^ However, the substantial effort required to identify, extract, appraise, and synthesize the evidence base offers an opportunity to innovate in future guideline development.

The GDG recommends considering the option of a living review of the evidence, and consequently a living guideline,^137^ something that was first widely used during COVID‐19.^138^ Given the relatively small number of new trials each year, such an approach would provide an opportunity to promptly identify and address any significant changes in the overall certainty of evidence, and with that, potential recommendations. This concept should be promoted as a standard practice for the development of clinical guidelines in general.

Such an approach would have resource implications, requiring the sponsoring societies to consider methods for commissioning and agreeing on specific operating procedures for this innovation,^139^ with similar being considered by other societies.^140^ This may be more efficient by pooling resource needed for vital but time‐consuming steps of the process.^141^ Consideration and joint agreement between ESPGHAN and NASPGHAN would be necessary. Such an approach would be the most effective way to achieve ongoing evidence‐based guidance within clinical practice.

## DISCLAIMERS

ESPGHAN is not responsible for the practices of physicians and provides guidelines and position papers as indicators of best practice only. Diagnosis and treatment are at the discretion of the healthcare provider.

Although this paper is produced by an ESPGHAN working group, it does not necessarily represent ESPGHAN policy and is not endorsed by ESPGHAN.

The NASPGHAN clinical practice guidelines and position papers are evidence‐based decision‐making tools for managing health conditions. This document is not a disease management requirement or rule and should not be construed as establishing a legal standard of care or as encouraging, advocating for, mandating, or discouraging any particular diagnostic methodology or treatment. Our clinical practice guidelines and position papers should also not be used in support of medical complaints, legal proceedings, and/or litigation, as they were not designed for this purpose. The NASPGHAN clinical practice guidelines and position papers should also not be utilized by insurance companies or pharmacy benefit managers to deny treatment that is deemed medically necessary by a patient's physician.

The health care team, patient, and family should make all decisions regarding the care of a patient, after consideration of individual‐specific medical circumstances. While NASPGHAN makes every effort to present accurate and reliable evidence‐based information, these clinical practice guidelines and position papers are provided “as is” without any warranty of accuracy, reliability, or otherwise, either express or implied. NASPGHAN does not guarantee, warrant, or endorse the products or services of any firm, organization, or person. Neither NASPGHAN nor its officers, directors, members, employees, or agents will be liable for any loss, damage, or claim with respect to any liabilities, including direct, special, indirect, nor consequential damages, incurred in connection with the clinical practice guidelines and/or position papers or reliance on the information presented.

## CONFLICT OF INTEREST STATEMENT

Regulations for conflict of interest followed the Cochrane handbook as well as the GRADE body. These implied that not only the receipt of financial contributions of institutions with links to assessed treatment options implied a conflict, but academic involvement in research on a specific treatment option without commercial funding as well. Marc Benninga indicated conflicts of interest for PEG, lactulose, linaclotide, prucalopride, lubiprostone, enema (disimpaction) biofeedback, fiber, CBT, physiotherapy, sacral nerve stimulation, rectoanal irrigation, probiotics, due to involvement in studies assessing these therapies. Marc Benninga also indicated consulting services for Norgine, Coloplast, Wellspect, Allergan, Mallinckrodt, United Pharmaceuticals, Danone, FrieslandCampina, and HIPP. Marc Benninga also served as speaker for Abbott and Menarini. Marc Benninga is a consultant for Coloplast, Wellspect, Danone, Sensus, FrieslandCampina, Cosun, Allergan, Abbott and Mallinckrodt. Juliette Hawa indicated conflicts of interest for biofeedback, as Juliette Hawa is listed as inventor, of pelvic health training device for home use. Julie Khlevner indicated conflicts of interest for linaclotide due to involvement in studies assessing this therapy. Julie Khlevner also indicated consulting services for Abbvie and Ironwood. Alexandra Kilgore indicated conflicts of interest for prucalopride due to involvement in studies assessing this therapy. Samuel Nurko indicated conflicts of interest for PEG, lubiprostone, and linaclotide, due to involvement in studies assessing these therapies. Merit Tabbers indicated conflicts of interest for probiotics due to involvement in studies assessing this therapy. Nikhil Thapar indicated conflicts of interest for probiotics due to involvement in studies assessing this therapy. Nikhil Thapar also indicated consulting services for BioGaia (not for >12 months). The remaining authors have not declared any conflicts of interest.

## Supporting information

Supplementary Materials File 1

Supplementary Materials File 2
