# Supplementary material for: European Society for Paediatric Gastroenterology, Hepatology and Nutrition/North American Society for Pediatric Gastroenterology, Hepatology and Nutrition guidelines for treatment of functional constipation in children aged 0–18 years
Source: J Pediatr Gastroenterol Nutr. 2026 May 20;83(2):335–66. doi: 10.1002/jpn3.70447 (PMC13446596; doi:10.1002/jpn3.70447)
Supplement: Supplementary file 1 [file JPN3-83-335-s001.docx]

**Supplementary Materials File 1**

- Supplementary Figure 1. Prisma flowchart
- Supplementary Figure 2. Risk of Bias Profiles of included studies
- Supplementary Figure 3. Risk of Bias Summary of included studies
- Supplementary Table 1. Excluded studies – reasons for exclusion
- Supplementary Table 2. Risk of Bias judgements and supporting details
- Supplementary Table 3. Characteristics of included pharmacological studies
- Supplementary Table 4. Characteristics of included non-pharmacological studies
- Supplementary Table 5. Secondary outcomes of included pharmacological studies
- Supplementary Table 6. Secondary outcomes of included non-pharmacological studies
- Supplementary Table 7. Details of side effects of included pharmacological studies
- Supplementary Table 8. Details of side effects of included non-pharmacological studies
- Supplementary Table 9. Voting results
- Supplementary Text 1. Reference List Supplementary Materials

**Supplementary Figure 1 - PRISMA Flowchart**

**Supplementary Figure 2 - Risk of Bias profiles of included studies**

**Supplementary Figure 3 - Risk of bias summary of included studies**

**Supplementary Table 1. Excluded studies – reasons for exclusion**

*Pharmacological disimpaction therapy*

| **Study ID** | **Reason for Exclusion** |
| --- | --- |
| Acharyya 2018 | No definition of fecal impaction (included children in which fecal impaction was diagnosed only based on X-ray) |
| Acharyya 2021 | Wrong population (also included children with fecal incontinence irrespective of fecal mass) |
| Candy 2006 | Wrong study design (disimpaction phase was not randomized) |
| Farahmand 2010 | No definition of fecal impaction |
| Hakimzadeh 2019 | Wrong populatin (also included children with occult constipation) |
| Miller 2012 | No definition of fecal impaction (included children in which fecal impaction was diagnosed only based on X-ray) |
| Savino 2012 | No definition of fecal impaction |
| Shatnawi 2019 | No definition of fecal impaction |
| Tolia 1993 | No definition of fecal impaction |
| Vadlapudi 2022 | No definition of fecal impaction (included children in which fecal impaction was diagnosed only based on X-ray) |
| Youssef 2002 | Wrong study design |

*Pharmacological maintenance therapy*

| **Study ID** | **Reason for Exclusion** |
| --- | --- |
| Acharyya 2018 | Disimpaction |
| Azary 2011 | Wrong Intervention |
| Baaleman 2021 | Wrong Study Design |
| Bellomo-Brandao 2003 | Wrong population/ Intervention |
| Bekkali 2009 | Disimpaction |
| Burgers 2010 | Wrong Study design |
| Cinca 2013 | Wrong population (Adults) |
| Colombo 2018 | Wrong population |
| Corazziari 1996 | Wrong population |
| Dupont 2000 | Wrong study design |
| Garcia 2016 | Wrong intervention (Fecal impaction) |
| Gordon 2013 | Wrong study design |
| Gremse 2002 | No definition of FC |
| Hakimzadeh 2019 | Wrong population |
| Halabi 1999 | Wrong intervention (cisapride) |
| Hejl 1990 | Wrong Population |
| Iacovou 2017 | Wrong Intervention |
| Jouet 2008 | Wrong population |
| Keshtgar 2007 | Wrong Intervention |
| Kumar 2018 | Wrong population |
| Martino 1992 | Wrong population |
| Mathew 2019 | Wrong study design |
| Maydeo 2010 | Wrong study design |
| Miller 2012 | Disimpaction |
| Ni 2001 | Wrong intervention |
| Nolan 1991 | Wrong population |
| Nurko 2000 | Wrong intervention |
| Odeka 1997 | Wrong intervention |
| Ormarsson 2013 | Wrong population |
| Poddar 2019 | Wrong study design |
| Poli 2016 | Wrong study design |
| Radwan 2021 | Wrong population |
| Rodriguez-Herrera 2017 | Wrong population |
| Shatnawi 2019 | Wrong Population (Disimpaction) |
| Silva 2013 | Wrong intervention |
| Sondheimer 1982 | Wrong study design |
| Staiano 1991 | Wrong intervention |
| Taylor 2015 | Wrong Population |
| Thomas 2013 | Wrong study design |
| Vadlapudi 2022 | Wrong intervention (Disimpaction) |
| Vandenplas 2017 | Wrong population |
| Vandenplas 2017 | Wrong intervention |
| VanSummeren 2019 | Wrong intervention |
| Wang 2022 | Wrong intervention |
| Wegner 2018 | Wrong intervention |
| Winter 2013 | Wrong study design |
| Xinias 2017 | Wrong population |
| Youssef 2002 | Disimpaction |

*Non-pharmacological maintenance therapy*

| **Study ID** | **Reason for exclusion** |
| --- | --- |
| Beleli 2015 | Wrong study design |
| Benninga 1993 | Wrong study design |
| Broide 2001 | Wrong study design |
| Canlorbe 1962 | No access |
| Chao 2016 | Lacks definition of functional constipation |
| El Azeem 2023 | Lacks definition of functional constipation |
| Garcia 2017 | Wrong study design |
| Horvath 2013 | Wrong study design |
| Irastorza 2010 | Wrong study design |
| Kajbafzadeh 2011 | Wrong population |
| Karagiozoglou-Lampoudi 2012 | Wrong outcomes |
| Leong 2011 | Wrong study design |
| Li 2022 | Wrong population |
| Loening-Baucke 1995 | Wrong study design |
| Lu 2011 | Wrong study design |
| Nolan 1996 | Wrong population |
| Olgac 2020 | Wrong outcomes |
| Poddar 2023 | Wrong study design |
| Ramsay 1999 | Wrong study design |
| Ruan 2018 | Wrong outcomes |
| Saneian 2013 | Wrong study design |
| Stepurina 2018 | Wrong intervention |
| Taniriverdi 2014 | Wrong study design |
| Toporovski 2021 | Wrong study design |
| Van Summeren 2019 | Wrong intervention (cost-effectiveness study) |
| Van Summeren 2020 | Lacks definition of functional constipation |
| Vignec 1952 | Wrong study design |
| Wagner 1988 | Wrong study design |
| Zoppi 1998 | Wrong intervention |
| Wang 2022 | Wrong study design (quasi-randomised study) |

**Supplementary Table 2. Risk of bias judgements and supporting details**

*Pharmacological disimpaction therapy studies*

| **Bekkali 2009** | |
| --- | --- |
| **Bias** | **Authors’ judgement and support for judgement** |
| Random sequence generation (selection bias) | Low risk |
|  | Author confirmed computer-generated randomisation. |
| Allocation concealment (selection bias) | Low risk |
|  | Author responded that they used the sealed envelopes method for allocation of participants. |
| Blinding of participants and personnel (performance bias) | High risk |
|  | Open label, patients and personnel could not be blinded due to differences in intervention. |
| Blinding of outcome assessment (detection bias) | High risk |
|  | Open label, patients and personnel could not be blinded due to differences in intervention. |
| Incomplete outcome data (attrition bias) | Low risk |
|  | All reasons for dropouts are stated. |
| Selective reporting (reporting bias) | High risk |
|  | Trial registration reported (NTR602). Results reported appropriately. No safety data reported. Only abdominal pain was measured. |
| Other bias | Low risk |
|  | Baseline characteristics are balanced. |

*Pharmacological maintenance therapy studies*

| **Ala 2015** | |
| --- | --- |
| **Bias** | **Authors’ judgement and support for judgement** |
| Random sequence generation (selection bias) | Low risk |
|  | dived randomly by random block of four |
| Allocation concealment (selection bias) | Unclear |
|  | Nothing mentioned |
| Blinding of participants and personnel (performance bias) | High risk |
|  | Open label |
| Blinding of outcome assessment (detection bias) | High risk |
|  | Open label |
| Incomplete outcome data (attrition bias) | Unclear |
|  | Unclear how many drop outs per group |
| Selective reporting (reporting bias) | Unclear |
|  | No protocol was found |
| Other bias | Low risk |
|  | No significant differences for baseline characteristics |

| **Bekkali 2018** | |
| --- | --- |
| **Bias** | **Authors’ judgement and support for judgement** |
| Random sequence generation (selection bias) | Low risk |
|  | Randomization numbers were generated by a licensed Clinical Research Organization |
| Allocation concealment (selection bias) | Low risk |
|  | Randomization number allocation was performed by an independent employee of the clinical research organization via telephone |
| Blinding of participants and personnel (performance bias) | Low Risk |
|  | Patients and study personnel were blinded. Packaging, labeling and dose per sachet were identical. |
| Blinding of outcome assessment (detection bias) | Low Risk |
|  | Patients and study personnel was blinded. Packaging, labeling and dose per sachet were identical. |
| Incomplete outcome data (attrition bias) | High risk |
|  | 1/3rd of patients withdrew due to 'other' no further info given regarding this. >20% of intervention group withdrew. Asked author, no specific reasons available |
| Selective reporting (reporting bias) | Low Risk |
|  | Key efficacy outcomes and a safety outcome reported |
| Other bias | Low Risk |
|  | Baseline demographics balanced |

| **Benninga 2022** | |
| --- | --- |
| **Bias** | **Authors’ judgement and support for judgement** |
| Random sequence generation (selection bias) | Low risk |
|  | Patients were assigned to treatment groups using randomization code and stratification scheme generated by the Randomization and Trial Supply Management system. |
| Allocation concealment (selection bias) | Low risk |
|  | Stratification scheme generated by the Randomization and Trial Supply Management system (ClinPhone; Parexel, Waltham, MA) |
| Blinding of participants and personnel (performance bias) | Low risk |
|  | Patients and parents were blinded, identical containers with and as identical soft gelatin capsules. Investigators were blinded as well. |
| Blinding of outcome assessment (detection bias) | Low risk |
|  | Patients and parents were blinded, identical containers with and as identical soft gelatin capsules. Investigators were blinded as well. |
| Incomplete outcome data (attrition bias) | Low risk |
|  | Reasons were reported. For some patients 'no discontinuation reason provided'. Patients were asked and didn’t give a reason. |
| Selective reporting (reporting bias) | Low risk |
|  | Key outcomes reported. Study per protocol. |
| Other bias | Low risk |
|  | Baseline characteristics balanced |

| **Blanco-Diaz 2020** | |
| --- | --- |
| **Bias** | **Authors’ judgement and support for judgement** |
| Random sequence generation (selection bias) | Low risk |
|  | Computer generated random sequence |
| Allocation concealment (selection bias) | Low risk |
|  | Sealed opaque envelopes |
| Blinding of participants and personnel (performance bias) | High risk |
|  | Open label |
| Blinding of outcome assessment (detection bias) | High risk |
|  | Open label |
| Incomplete outcome data (attrition bias) | Unclear |
|  | Unclear. Only report compliance and attendance rates. At every outcome measurement point, the number of patients differ. Unclear if patients were lost to follow-up. |
| Selective reporting (reporting bias) | High risk |
|  | No protocol found. They do not mention anything about recording side effects during the study (only the Symptom Severity Score), but do say in their conclusion that Manual Therapy has no side effects. |
| Other bias | Low risk |
|  | Baseline demographics balanced |

| **Bongers 2009** | |
| --- | --- |
| **Bias** | **Authors’ judgement and support for judgement** |
| Random sequence generation (selection bias) | Low risk |
|  | Computerized randomization was used to generate a sequence of random group assignment for consecutive patients This computer program based on the biased coin method used minimization to achieve a balanced randomization on 2 factors, gender and age (13 years versus 13 years). |
| Allocation concealment (selection bias) | Low risk |
|  | Contact with the authors: concealed envelopes |
| Blinding of participants and personnel (performance bias) | High risk |
|  | Open label |
| Blinding of outcome assessment (detection bias) | High risk |
|  | Open label |
| Incomplete outcome data (attrition bias) | Low risk |
|  | 4 patients in IG group refused further application, no reasons provided. Contact with authors: patients were asked but did not provide a reason. Number too small that it could have affected the results. |
| Selective reporting (reporting bias) | Low risk |
|  | The trial was registered retrospectively, authors confirmed that protocol was made prospectively. Primary outcome was reported as predefined in the protocol. |
| Other bias | Low risk |
|  | Baseline demographics balanced |

| **Bu 2007** | |
| --- | --- |
| **Bias** | **Authors’ judgement and support for judgement** |
| Random sequence generation (selection bias) | Low risk |
|  | Computer-generated randomization list |
| Allocation concealment (selection bias) | Unclear |
|  | Not reported |
| Blinding of participants and personnel (performance bias) | Low risk |
|  | Three interventions with similar appearances and placed into identical capsules. |
| Blinding of outcome assessment (detection bias) | Low risk |
|  | Three interventions with similar appearances and placed into identical capsules |
| Incomplete outcome data (attrition bias) | Unclear |
|  | Numbers per group reported, no imbalance. Reasons provided, however unclear which reasons belong to which patient/group. Two patients suffered from actue gastroenteritis, unclear in which group. |
| Selective reporting (reporting bias) | Unclear |
|  | No protocol found or trial registration. |
| Other bias | Low risk |
|  | Baseline demographics balanced |

| **Cao 2018** | |
| --- | --- |
| **Bias** | **Authors’ judgement and support for judgement** |
| Random sequence generation (selection bias) | Low risk |
|  | Computer generated |
| Allocation concealment (selection bias) | Low risk |
|  | Any randomization and allocation information were concealed in opaque sealed envelopes |
| Blinding of participants and personnel (performance bias) | Low risk |
|  | Patient and study personnel masked. Participants in the placebo group got placebo, the same size, dose, color, flavor, and appearance as the lactulose in the treatment group. |
| Blinding of outcome assessment (detection bias) | Low risk |
|  | Patient and study personnel masked. Participants in the placebo group got placebo, the same size, dose, color, flavor, and appearance as the lactulose in the treatment group. |
| Incomplete outcome data (attrition bias) | Low risk |
|  | All drop outs per group reported with reasons. Represented in flow diagram. |
| Selective reporting (reporting bias) | Unclear |
|  | No protocol found. Key safety data and outcomes reported. |
| Other bias | Low risk |
|  | Baseline demographics balanced |

| **Cassettari 2019** | |
| --- | --- |
| **Bias** | **Authors’ judgement and support for judgement** |
| Random sequence generation (selection bias) | Low risk |
|  | Subjects were randomly assigned into five treatment groups by a mathematical algorithm |
| Allocation concealment (selection bias) | Unclear |
|  | Not reported |
| Blinding of participants and personnel (performance bias) | Low risk |
|  | Patients and personnel blinded. Biomass looked identical as a thick, white, homogenous mass. No substantial variation in colour, taste, and smell. |
| Blinding of outcome assessment (detection bias) | Low risk |
|  | Patients and personnel blinded. Biomass looked identical as a thick, white, homogenous mass. No substantial variation in colour, taste, and smell. |
| Incomplete outcome data (attrition bias) | Low risk |
|  | All drop outs per group reported with reasons. |
| Selective reporting (reporting bias) | Low risk |
|  | Key outcomes and safety data reported per protocol. |
| Other bias | Low risk |
|  | Baseline demographics balanced |

| **Dehghani 2014** | |
| --- | --- |
| **Bias** | **Authors’ judgement and support for judgement** |
| Random sequence generation (selection bias) | Low risk |
|  | Patients were randomly assigned by a computer-generated method with the individual patient as the unit of randomization |
| Allocation concealment (selection bias) | Low risk |
|  | Each regimen packed by pharmacist with a special code, so that neither the physician nor patient knew what regimen was consigned to each subject. |
| Blinding of participants and personnel (performance bias) | Low risk |
|  | Both intervention and control group received treatment with the same colour, taste, and smell (as syrup). Study personnel was also blinded. |
| Blinding of outcome assessment (detection bias) | Low risk |
|  | Both intervention and control group received treatment with the same colour, taste, and smell (as syrup). Study personnel was also blinded. |
| Incomplete outcome data (attrition bias) | Low risk |
|  | All drop outs per group reported with reasons. Represented in flow diagram. |
| Selective reporting (reporting bias) | Unclear risk |
|  | Study protocol present, but trial registration in 2013 and started including in 2011. Outcomes reported. |
| Other bias | Low risk |
|  | Baseline demographics balanced. |

| **Dehghani 2019** | |
| --- | --- |
| **Bias** | **Authors’ judgement and support for judgement** |
| Random sequence generation (selection bias) | Low risk |
|  | Block randomisation |
| Allocation concealment (selection bias) | Low risk |
|  | Word ‘allocation’ used to describe joining group without further discussion. Contacted author, answer: allocation concealment was done by numbered drug containers. |
| Blinding of participants and personnel (performance bias) | Low risk |
|  | Drug and placebo prepared with similar organoleptic properties; packaged in identical containers. Patients and personnel blinded. |
| Blinding of outcome assessment (detection bias) | Low risk |
|  | Drug and placebo prepared with similar organoleptic properties; packaged in identical containers. Patients and personnel blinded. |
| Incomplete outcome data (attrition bias) | Low risk |
|  | Flow of patients including randomised and assessed, drop outs reported with reasons given in flow chart. |
| Selective reporting (reporting bias) | Low risk |
|  | Key outcomes and safety data reported per protocol. |
| Other bias | Low risk |
|  | Baseline demographics balanced. |

| **Dheivamani 2021** | |
| --- | --- |
| **Bias** | **Authors’ judgement and support for judgement** |
| Random sequence generation (selection bias) | Low risk |
|  | Block Randomisation via computer-generated codes |
| Allocation concealment (selection bias) | Low risk |
|  | Allocation concealment performed using sealed envelopes |
| Blinding of participants and personnel (performance bias) | Low risk |
|  | Open label trial |
| Blinding of outcome assessment (detection bias) | Low risk |
|  | Open label trial |
| Incomplete outcome data (attrition bias) | Low risk |
|  | All drop-outs reported per group as represented in flow chart |
| Selective reporting (reporting bias) | Low risk |
|  | Key outcomes and safety outcomes reported per protocol. |
| Other bias | Low risk |
|  | Baseline demographics balanced. |

| **Dupont 2005** | |
| --- | --- |
| **Bias** | **Authors’ judgement and support for judgement** |
| Random sequence generation (selection bias) | Unclear |
|  | Randomisation but with no specified method - only reference to a 'randomisation list' |
| Allocation concealment (selection bias) | Unclear |
|  | States randomly allocated but no specific detail. |
| Blinding of participants and personnel (performance bias) | Low risk |
|  | Double-dummy design due to difference in taste |
| Blinding of outcome assessment (detection bias) | Low risk |
|  | Double-dummy design |
| Incomplete outcome data (attrition bias) | Low risk |
|  | All drop-outs reported per group as represented in flow chart |
| Selective reporting (reporting bias) | Unclear |
|  | No protocol found but key outcomes and safety data reported. |
| Other bias | Low risk |
|  | Baseline demographics balanced. |

| **Dzeichciarz 2015** | |
| --- | --- |
| **Bias** | **Authors’ judgement and support for judgement** |
| Random sequence generation (selection bias) | Low risk |
|  | Block Randomisation |
| Allocation concealment (selection bias) | Low risk |
|  | Allocation via concealed lists without elaboration. Contacted author, answer: used concealed list which means that random generated list and sequentially numbered, opaque, sealed envelopes was kept by the person not involved in entering patients into the trial. |
| Blinding of participants and personnel (performance bias) | High Risk |
|  | Open label |
| Blinding of outcome assessment (detection bias) | High Risk |
|  | Open label |
| Incomplete outcome data (attrition bias) | Low Risk |
|  | Flow chart available and drop outs reported. |
| Selective reporting (reporting bias) | Low Risk |
|  | Study protocol present. Key outcomes and safety data reported. |
| Other bias | Low Risk |
|  | Baseline demographics balanced. |

| **Di Lorenzo 2020 (abstract only)** | |
| --- | --- |
| **Bias** | **Authors’ judgement and support for judgement** |
| Random sequence generation (selection bias) | Low risk |
|  | Computer generated |
| Allocation concealment (selection bias) | Low risk |
|  | Central allocation |
| Blinding of participants and personnel (performance bias) | Low risk |
|  | Participant, Care Provider, Investigator, Outcomes Assessor were blinded. Matching placebo. |
| Blinding of outcome assessment (detection bias) | Low risk |
|  | Participant, Care Provider, Investigator, Outcomes Assessor were blinded. Matching placebo. |
| Incomplete outcome data (attrition bias) | Low risk |
|  | After answer of author: drop outs equal between groups and reasons reported. |
| Selective reporting (reporting bias) | Low risk |
|  | After answer of authors: Key outcomes and safety outcomes reported as per protocol. |
| Other bias | Low risk |
|  | Baseline demographics balanced. |

| **Di Lorenzo 2024** | |
| --- | --- |
| **Bias** | **Authors’ judgement and support for judgement** |
| Random sequence generation (selection bias) | Low risk |
|  | Randomisation was by block randomisation (block size four) |
| Allocation concealment (selection bias) | Low risk |
|  | Assigned (1:1) by use of an interactive web response system (IWRS; Premier Research International Interactive Response Technologies, East Hartford, CT, USA) to receive either linaclotide or placebo. A sponsor randomization personnel generated the randomisation schedule and provided it to IWRS for implementation. The randomization sequence was not visible to any staff at the investigational site. |
| Blinding of participants and personnel (performance bias) | Low risk |
|  | Linaclotide and placebo were provided in matching capsules with identical appearance. Participants, site investigators, study outcome assessors, and bioanalytical representatives (ie, those analysing the data) were masked to study treatment |
| Blinding of outcome assessment (detection bias) | Low risk |
|  | Linaclotide and placebo were provided in matching capsules with identical appearance. Participants, site investigators, study outcome assessors, and bioanalytical representatives (ie, those analysing the data) were masked to study treatment |
| Incomplete outcome data (attrition bias) | Low risk |
|  | Dropout rate balanced, and reasons provided in flow-chart |
| Selective reporting (reporting bias) | Low risk |
|  | Study and outcomes reported per protocol |
| Other bias | Low risk |
|  | Baseline demographics balanced |

| **Di Lorenzo 2024b** | |
| --- | --- |
| **Bias** | **Authors’ judgement and support for judgement** |
| Random sequence generation (selection bias) | Low risk |
|  | Randomisation was by statistical programming. |
| Allocation concealment (selection bias) | Low risk |
|  | Implemented by the interactive web response system vendor, which identified each patient by randomization number and included the corresponding treatment intervention assignment. |
| Blinding of participants and personnel (performance bias) | Unclear |
|  | double‐blind. patient was dispensed the blinded study intervention. No mention of differences between interventions and placebo. |
| Blinding of outcome assessment (detection bias) | Low risk |
|  | Carers were the outcome assessors and were masked to study treatment. |
| Incomplete outcome data (attrition bias) | Low risk |
|  | Low and balanced dropout rate balanced with reasons provided |
| Selective reporting (reporting bias) | Low risk |
|  | Study and outcomes reported per protocol |
| Other bias | Unclear |
|  | Some imbalances for basic efficacy variables. |

| **Elkaragy 2024** | |
| --- | --- |
| **Bias** | **Authors’ judgement and support for judgement** |
| Random sequence generation (selection bias) | Low risk |
|  | Randomisation using a computer-assisted, simple parallel randomization method. |
| Allocation concealment (selection bias) | Low risk |
|  | Concealment of allocation was implemented by providing the treatment to the participants in opaque, sealed, serially numbered, nonlabelled jars/bottles of equal appearance and weight (after calculation of the corresponding appropriate dose). This was done by one of the investigators who had no further involvement in the research conduct, in collaboration with the clinical pharmacy department. |
| Blinding of participants and personnel (performance bias) | High risk |
|  | Concealment in the study was intended for the participants the involved investigator was not blinded. single‐blinded. |
| Blinding of outcome assessment (detection bias) | Low risk |
|  | Patients and their parents/legal guardians were outcome assessors and were masked to study treatment. |
| Incomplete outcome data (attrition bias) | Low risk |
|  | Low and balanced dropout rate balanced with reasons provided |
| Selective reporting (reporting bias) | Low risk |
|  | First dose response time and responders rate are in protocol but not in paper. But no mention of the definition of the responders. |
| Other bias | Low risk |
|  | Baseline demographics balanced. |

| **Esmaeilidooki 2016** | |
| --- | --- |
| **Bias** | **Authors’ judgement and support for judgement** |
| Random sequence generation (selection bias) | Unclear |
|  | Stated ‘Simple Randomisation' without specified method. |
| Allocation concealment (selection bias) | Unclear |
|  | Stated 'Random allocation' without specified method. |
| Blinding of participants and personnel (performance bias) | High risk |
|  | Open label |
| Blinding of outcome assessment (detection bias) | High risk |
|  | Open label |
| Incomplete outcome data (attrition bias) | Low risk |
|  | All drop-outs reported per group as represented in flow chart |
| Selective reporting (reporting bias) | Low risk |
|  | Protocol available. Key safety data and outcomes reported. |
| Other bias | Low risk |
|  | Baseline demographics balanced. |

| **Farahmand 2007** | |
| --- | --- |
| **Bias** | **Authors’ judgement and support for judgement** |
| Random sequence generation (selection bias) | Unclear |
|  | Unclear how randomisation occurred. |
| Allocation concealment (selection bias) | Unclear |
|  | Not mentioned |
| Blinding of participants and personnel (performance bias) | High risk |
|  | Open Label Trial |
| Blinding of outcome assessment (detection bias) | High risk |
|  | Open Label Trial |
| Incomplete outcome data (attrition bias) | Unclear |
|  | No drop outs reported but 'lost to follow up' mentioned in |
| Selective reporting (reporting bias) | Unclear |
|  | No protocol. Key safety data and outcomes reported. |
| Other bias | Low risk |
|  | Baseline demographics balanced. |

| **Foroughi 2022** | |
| --- | --- |
| **Bias** | **Authors’ judgement and support for judgement** |
| Random sequence generation (selection bias) | Unclear |
|  | Simple Randomisation method used to divide patients into 4 groups. No specified method. |
| Allocation concealment (selection bias) | Unclear |
|  | Not mentioned |
| Blinding of participants and personnel (performance bias) | Low risk |
|  | Drugs were prepared in identical packages and unlabelled sachets with only codes. patient and researcher were blinded. no mention of taste differences between the interventions. |
| Blinding of outcome assessment (detection bias) | Low risk |
|  | As above |
| Incomplete outcome data (attrition bias) | Unclear |
|  | Not mentioned. |
| Selective reporting (reporting bias) | Unclear |
|  | Protocol, key outcomes present. Safety Data not reported |
| Other bias | Low risk |
|  | Baseline demographics balanced. But do miss 3 patients in group B. |

| **Gomes 2011** | |
| --- | --- |
| **Bias** | **Authors’ judgement and support for judgement** |
| Random sequence generation (selection bias) | Low risk |
|  | Mentions randomisation without specified method. Contacted authors: low tech (coins heads or tails) |
| Allocation concealment (selection bias) | Unclear |
|  | Not mentioned. Contacted authors: still unclear. |
| Blinding of participants and personnel (performance bias) | High risk |
|  | Non-blind Trial |
| Blinding of outcome assessment (detection bias) | High risk |
|  | Non-blind Trial |
| Incomplete outcome data (attrition bias) | High risk |
|  | Losses reported but no reason given. No flow chart. |
| Selective reporting (reporting bias) | High risk |
|  | Protocol available. No Safety Data reported. |
| Other bias | Low risk |
|  | Baseline demographics balanced. |

| **Imanieh 2022** | |
| --- | --- |
| **Bias** | **Authors’ judgement and support for judgement** |
| Random sequence generation (selection bias) | Low risk |
|  | Permuted block randomization was used for randomly allocating the participants to the two groups. |
| Allocation concealment (selection bias) | Low risk |
|  | Allocation concealment was achieved using the dark envelope method. |
| Blinding of participants and personnel (performance bias) | Low risk |
|  | Containers with the same shape, colour, and weight, so that the containers and solutions would not be distinguishable (no mention of taste). The patients, physicians, and the analyst were not aware of the ingredients of the solutions, only the pharmacist. |
| Blinding of outcome assessment (detection bias) | Low risk |
|  | Containers with the same shape, colour, and weight, so that the containers and solutions would not be distinguishable (no mention of taste). The patients, physicians, and the analyst were not aware of the ingredients of the solutions, only the pharmacist |
| Incomplete outcome data (attrition bias) | Low risk |
|  | Reasons provided. No imbalance in number of patients per group (6 vs 2) |
| Selective reporting (reporting bias) | Unclear |
|  | Primary outcome in paper: not fulfilling Rome IV criteria anymore. In protocol primary outcomes are all Rome IV criteria (and more) separately. |
| Other bias | High risk |
|  | Most of the baseline demographics balanced. Duration of functional constipation differs between groups: 74% of IG and 44% CG had been suffering from functional constipation for more than 12 months. |

| **Jarzebicka 2019** | |
| --- | --- |
| **Bias** | **Authors’ judgement and support for judgement** |
| Random sequence generation (selection bias) | Low risk |
|  | Computer generated |
| Allocation concealment (selection bias) | Low risk |
|  | The investigators faxed a request for randomization to central randomization centre (CRC). Study staff assigned the patient to the appropriate place on the list according to centre and block to learn the randomization arm. In return, treating physicians received a fax back with the treatment arm for the patient. |
| Blinding of participants and personnel (performance bias) | High risk |
|  | Open label |
| Blinding of outcome assessment (detection bias) | High risk |
|  | Open label |
| Incomplete outcome data (attrition bias) | Unclear |
|  | Withdrawals reported with partial explanations. No reasons given for other. |
| Selective reporting (reporting bias) | Low risk |
|  | Study protocol present. Key outcomes and safety data reported. |
| Other bias | Low risk |
|  | Baseline demographics balanced. |

| **Karami 2009** | |
| --- | --- |
| **Bias** | **Authors’ judgement and support for judgement** |
| Random sequence generation (selection bias) | Unclear |
|  | Mentions systematic and random sampling. Unclear how. |
| Allocation concealment (selection bias) | Unclear |
|  | Not mentioned |
| Blinding of participants and personnel (performance bias) | High risk |
|  | Single blinded. Unclear how blinded and medication vastly different and as such would likely make high risk |
| Blinding of outcome assessment (detection bias) | High risk |
|  | Single blinded. Unclear how blinded and medication vastly different and as such would likely make high risk |
| Incomplete outcome data (attrition bias) | High risk |
|  | No reasons reported for dropouts |
| Selective reporting (reporting bias) | High risk |
|  | No protocol found. Safety was not an outcome measure in this study. 4month follow up data not reported |
| Other bias | Unclear |
|  | Not all baseline data reported per group (eg. age). Also unclear if reported baseline data is for all randomized patients. |

| **Khodadad 2010** | |
| --- | --- |
| **Bias** | **Authors’ judgement and support for judgement** |
| Random sequence generation (selection bias) | Unclear |
|  | Randomisation was generated by their biostatistics consultant. However, unclear how randomisation occurred. |
| Allocation concealment (selection bias) | Unclear |
|  | Not reported |
| Blinding of participants and personnel (performance bias) | Unclear |
|  | Physicians and nurses were blinded. Bottles and sachets were similar in shape, taste and colour. Only label indicating A or B. However: Group A received 1.5 ml/kg/day oral liquid paraffin plus placebo, group B received 1 sachet synbiotic per day, Group C received 1.5 ml/kg/day oral liquid paraffin and 1 sachet synbiotic per day. Group B seemed to only receive a sachet with synbiotics, no placebo for laxative. |
| Blinding of outcome assessment (detection bias) | Unclear |
|  | Physicians and nurses were blinded. Bottles and sachets were similar in shape, taste and colour. Only label indicating A or B. However: Group A received 1.5 ml/kg/day oral liquid paraffin plus placebo, group B received 1 sachet synbiotic per day, Group C received 1.5 ml/kg/day oral liquid paraffin and 1 sachet synbiotic per day. Group B seemed to only receive a sachet with synbiotics, no placebo for laxative. |
| Incomplete outcome data (attrition bias) | Low risk |
|  | All drop-outs reported per group as represented in flow chart with reasons. |
| Selective reporting (reporting bias) | Low risk |
|  | Key outcomes (efficacy and safety) reported as per protocol |
| Other bias | Low risk |
|  | Baseline characteristics balanced |

| **Kokke 2008** | |
| --- | --- |
| **Bias** | **Authors’ judgement and support for judgement** |
| Random sequence generation (selection bias) | Low risk |
|  | Use of sequential numbers allocated to the patients at study entry and coordinated by the logistics manager of Numico Research using a block design. |
| Allocation concealment (selection bias) | Unclear |
|  | Nothing mentioned |
| Blinding of participants and personnel (performance bias) | Low risk |
|  | Patients and personnel blinded. Clear description of how both interventions were packed and prepared. Products could not be distinguished. |
| Blinding of outcome assessment (detection bias) | Low risk |
|  | Patients and personnel blinded. Clear description of how both interventions were packed and prepared. Products could not be distinguished. |
| Incomplete outcome data (attrition bias) | Low Risk |
|  | Flow-diagram with reasons. |
| Selective reporting (reporting bias) | Unclear |
|  | No protocol found. |
| Other bias | Unclear |
|  | Baseline characteristics only reported for patients who reached end of study. |

| **Kubota 2020** | |
| --- | --- |
| **Bias** | **Authors’ judgement and support for judgement** |
| Random sequence generation (selection bias) | Low risk |
|  | Block randomisation |
| Allocation concealment (selection bias) | Unclear |
|  | Not mentioned |
| Blinding of participants and personnel (performance bias) | Low risk |
|  | All caregivers, patients, research staff, and physicians were blinded to which treatment group the patients belonged. Identical matching placebo and probiotic. bottles were matched and taste/texture were matched. |
| Blinding of outcome assessment (detection bias) | Low risk |
|  | All caregivers, patients, research staff, and physicians were blinded to which treatment group the patients belonged. Identical matching placebo and probiotic. bottles were matched and taste/texture were matched. |
| Incomplete outcome data (attrition bias) | Unclear |
|  | Unclear to which group the excluded patients belonged to. Only 3 patients. |
| Selective reporting (reporting bias) | Unclear |
|  | No protocol found or trial registration details |
| Other bias | Low risk |
|  | Baseline characteristics balanced |

| **Lee 2022** | |
| --- | --- |
| **Bias** | **Authors’ judgement and support for judgement** |
| Random sequence generation (selection bias) | Low risk |
|  | Randomization was implemented automatically using Random Allocation Software 2.0 (Informer Technologies, Inc, Dallas, TX, USA) with a random block size |
| Allocation concealment (selection bias) | Unclear |
|  | Not reported |
| Blinding of participants and personnel (performance bias) | High risk |
|  | Open label |
| Blinding of outcome assessment (detection bias) | High risk |
|  | Open label |
| Incomplete outcome data (attrition bias) | High risk |
|  | High amount of drop-outs, imbalance > 20% and reasons not specified for each group |
| Selective reporting (reporting bias) | Low risk |
|  | Protocol found. Key outcomes reported as per protocol. Safety data also reported in study. |
| Other bias | Low risk |
|  | "There were no differences in age, disease duration, or other clinical characteristics among the 3 groups." There were sex differences between combination therapy and s. boulardii. They adjusted for sex in their Cox Regression analysis. |

| **Loening-Baucke 2006** | |
| --- | --- |
| **Bias** | **Authors’ judgement and support for judgement** |
| Random sequence generation (selection bias) | Unclear |
|  | Mentions randomization, but not how |
| Allocation concealment (selection bias) | Low risk |
|  | Randomization was performed by children drawing a sealed envelope with an enclosed assignment. |
| Blinding of participants and personnel (performance bias) | High risk |
|  | Open label |
| Blinding of outcome assessment (detection bias) | High risk |
|  | Open label |
| Incomplete outcome data (attrition bias) | Low risk |
|  | Flow diagram with drop-outs reported and reasons provided. Imbalance in number of dropouts, however due to difference in drug. |
| Selective reporting (reporting bias) | Unclear |
|  | No protocol found. |
| Other bias | Low risk |
|  | Baseline demographics balanced. |

| **Modin 2018** | |
| --- | --- |
| **Bias** | **Authors’ judgement and support for judgement** |
| Random sequence generation (selection bias) | Low risk |
|  | Computer-generated randomization blocks of 10 children |
| Allocation concealment (selection bias) | Unclear |
|  | Unclear if the investigational drug pharmacist was not involved and pharmacist was not blinded for age/weight. |
| Blinding of participants and personnel (performance bias) | Low risk |
|  | Drugs had similar taste and consistency, identical packaging. Children, parents, and investigators were blinded. |
| Blinding of outcome assessment (detection bias) | Low risk |
|  | Drugs had similar taste and consistency, identical packaging. Children, parents, and investigators were blinded. |
| Incomplete outcome data (attrition bias) | Low risk |
|  | Reasons for dropout provided. No imbalance in dropouts. |
| Selective reporting (reporting bias) | Low risk |
|  | According to protocol. Only safety was not included in protocol, but was an outcome in article. |
| Other bias | Low risk |
|  | Baseline demographics balanced |

| **Mozaffarpur 2012** | |
| --- | --- |
| **Bias** | **Authors’ judgement and support for judgement** |
| Random sequence generation (selection bias) | Unclear |
|  | Systematic randomisation |
| Allocation concealment (selection bias) | Unclear |
|  | Not reported |
| Blinding of participants and personnel (performance bias) | High risk |
|  | Open Label Trial |
| Blinding of outcome assessment (detection bias) | High risk |
|  | Open Label Trial |
| Incomplete outcome data (attrition bias) | Low risk |
|  | Reasons for drop-out are clear and no imbalance |
| Selective reporting (reporting bias) | Low risk |
|  | Key outcomes (efficacy and safety) reported as per protocol |
| Other bias | Low risk |
|  | Baseline characteristics balanced |

| **Mugie 2014** | |
| --- | --- |
| **Bias** | **Authors’ judgement and support for judgement** |
| Random sequence generation (selection bias) | Low risk |
|  | Randomization was organized using a central interactive web-based, voice-response system, which applied a minimization algorithm and generated a medication number to ensure blinding. |
| Allocation concealment (selection bias) | Low risk |
|  | Author answered: pharmacy allocated the drugs |
| Blinding of participants and personnel (performance bias) | Low risk |
|  | Patients and investigators were blinded to treatment allocation. Placebo was identical in taste and appearance to prucalopride. |
| Blinding of outcome assessment (detection bias) | Low risk |
|  | Patients and investigators were blinded to treatment allocation. Placebo was identical in taste and appearance to prucalopride. |
| Incomplete outcome data (attrition bias) | Low risk |
|  | Reasons mentioned per patient and per group, no imbalance in number of dropouts |
| Selective reporting (reporting bias) | Low risk |
|  | Key outcomes reported. Study as per protocol |
| Other bias | Low risk |
|  | Baseline characteristics balanced. |

| **Nasri 2022** | |
| --- | --- |
| **Bias** | **Authors’ judgement and support for judgement** |
| Random sequence generation (selection bias) | Unclear |
|  | Not clear how patients were randomly assigned |
| Allocation concealment (selection bias) | Unclear |
|  | Not reported |
| Blinding of participants and personnel (performance bias) | High risk |
|  | Open label |
| Blinding of outcome assessment (detection bias) | High risk |
|  | Open label |
| Incomplete outcome data (attrition bias) | Low risk |
|  | No drop outs reported. |
| Selective reporting (reporting bias) | High risk |
|  | Protocol available. However, safety data not reported, was described in protocol as a secondary outcome + in methods section. |
| Other bias | Low risk |
|  | Baseline demographics balanced |

| **Nimrouzi 2015** | |
| --- | --- |
| **Bias** | **Authors’ judgement and support for judgement** |
| Random sequence generation (selection bias) | Low risk |
|  | Block randomization |
| Allocation concealment (selection bias) | Unclear |
|  | Not reported |
| Blinding of participants and personnel (performance bias) | High risk |
|  | Open label |
| Blinding of outcome assessment (detection bias) | High risk |
|  | Open label |
| Incomplete outcome data (attrition bias) | Low risk |
|  | Reasons per group reported. No imbalance per group. |
| Selective reporting (reporting bias) | Unclear |
|  | No protocol found |
| Other bias | Unclear |
|  | Baseline characteristics not reported for all included patients, only the ones that reached study end. |

| **Nurko 2008** | |
| --- | --- |
| **Bias** | **Authors’ judgement and support for judgement** |
| Random sequence generation (selection bias) | Low risk |
|  | Randomisation by random blocks of 20 patients |
| Allocation concealment (selection bias) | Unclear |
|  | Not reported |
| Blinding of participants and personnel (performance bias) | Low risk |
|  | No difference in color, appearance, or taste among different dosages and placebo. Identical bottles. Research team and patients were blinded. |
| Blinding of outcome assessment (detection bias) | Low risk |
|  | No difference in color, appaearance, or taste among different dosages and placebo. Identical bottles.  Research team and patients were blinded |
| Incomplete outcome data (attrition bias) | Low risk |
|  | Reason for drop-out reported per patient and no imbalance. |
| Selective reporting (reporting bias) | Unclear |
|  | No protocol found |
| Other bias | Low risk |
|  | Baseline demographics balanced |

| **Olgac 2013** | |
| --- | --- |
| **Bias** | **Authors’ judgement and support for judgement** |
| Random sequence generation (selection bias) | Unclear |
|  | Says random, but not how random occurred |
| Allocation concealment (selection bias) | Unclear |
|  | Not mentioned |
| Blinding of participants and personnel (performance bias) | High risk |
|  | Open Label Trial |
| Blinding of outcome assessment (detection bias) | High risk |
|  | Open Label Trial |
| Incomplete outcome data (attrition bias) | Low risk |
|  | Reasons for drop-out are clear and no imbalance |
| Selective reporting (reporting bias) | Unclear |
|  | No protocol found or trial registration details |
| Other bias | Low risk |
|  | Baseline characteristics balanced |

| **Pitzalis 1995** | |
| --- | --- |
| **Bias** | **Authors’ judgement and support for judgement** |
| Random sequence generation (selection bias) | Unclear |
|  | Unclear how randomisation occurred |
| Allocation concealment (selection bias) | Unclear |
|  | Not reported |
| Blinding of participants and personnel (performance bias) | High risk |
|  | Open label |
| Blinding of outcome assessment (detection bias) | High risk |
|  | Open label |
| Incomplete outcome data (attrition bias) | Unclear |
|  | Reasons not reported per group |
| Selective reporting (reporting bias) | Unclear |
|  | No protocol found |
| Other bias | Unclear |
|  | Baseline characteristics only reported for patietns who reachted study end |

| **Quitadamo 2012** | |
| --- | --- |
| **Bias** | **Authors’ judgement and support for judgement** |
| Random sequence generation (selection bias) | Low risk |
|  | Randomly assigned according to an automatically generated randomization list |
| Allocation concealment (selection bias) | Unclear |
|  | Not reported |
| Blinding of participants and personnel (performance bias) | High risk |
|  | Open label |
| Blinding of outcome assessment (detection bias) | High risk |
|  | Open label |
| Incomplete outcome data (attrition bias) | Low risk |
|  | Clear reasons for drop-out per patient. Difference between both groups > 20%, but this is a result of the difference in drug (bad taste). |
| Selective reporting (reporting bias) | Unclear |
|  | No protocol found |
| Other bias | Low risk |
|  | Baseline demographics balanced |

| **Rafati 2011** | |
| --- | --- |
| **Bias** | **Authors’ judgement and support for judgement** |
| Random sequence generation (selection bias) | Low risk |
|  | Random table was used to randomize the patients |
| Allocation concealment (selection bias) | Unclear |
|  | Not reported |
| Blinding of participants and personnel (performance bias) | High risk |
|  | Open label |
| Blinding of outcome assessment (detection bias) | High risk |
|  | Open label |
| Incomplete outcome data (attrition bias) | High risk |
|  | Unclear reasons for drop-outs in liquid paraffin group |
| Selective reporting (reporting bias) | High risk |
|  | No protocol found. Table 3: "need to additive drugs". However nothing was reported in methods section about additive drugs (evidence of plan deviation). |
| Other bias | Unclear |
|  | Baseline characteristics only reported for patients that made study end |

| **Ratamangkol 2009** | |
| --- | --- |
| **Bias** | **Authors’ judgement and support for judgement** |
| Random sequence generation (selection bias) | Low risk |
|  | Computer-generated randomization list in mix block sizes by a nonparticipating statistician. |
| Allocation concealment (selection bias) | Low risk |
|  | Treatment allocation was prepared in separated sealed, opaque sequentially numbered envelops. Dispensed by a blinded nurse. |
| Blinding of participants and personnel (performance bias) | High risk |
|  | Could not be blinded, because medications were administered to children in different ways. |
| Blinding of outcome assessment (detection bias) | High risk |
|  | Could not be blinded, because medications were administered to children in different ways. |
| Incomplete outcome data (attrition bias) | Low risk |
|  | Reasons for drop-out are reported. Drop-outs equal per group. |
| Selective reporting (reporting bias) | Unclear |
|  | No protocol found |
| Other bias | Unclear |
|  | Baseline characteristics only reported for patients who reached study end |

| **Saneian 2012** | |
| --- | --- |
| **Bias** | **Authors’ judgement and support for judgement** |
| Random sequence generation (selection bias) | Low risk |
|  | Systematic randomization using the randomization software |
| Allocation concealment (selection bias) | Unclear |
|  | Not reported |
| Blinding of participants and personnel (performance bias) | High risk |
|  | Open label |
| Blinding of outcome assessment (detection bias) | High risk |
|  | Open label |
| Incomplete outcome data (attrition bias) | Unclear |
|  | Unclear how many patients randomized, nothing mentioned about dropouts |
| Selective reporting (reporting bias) | High risk |
|  | No protocol found. Treatment success was not reported as how it was predefined in the methods. Safety was not described as an outcome in methods, but side effects are reported in results section. |
| Other bias | Low risk |
|  | Baseline demographics balanced |

| **Saneian 2021** | |
| --- | --- |
| **Bias** | **Authors’ judgement and support for judgement** |
| Random sequence generation (selection bias) | Low risk |
|  | Block randomization was done with a computer‑generated random number list prepared by an investigator with no clinical involvement in the trial. |
| Allocation concealment (selection bias) | Unclear |
|  | Not reported |
| Blinding of participants and personnel (performance bias) | Unclear |
|  | Unclear how clinicians who enrolled the patients or assessing the outcomes, and the parents were blinded |
| Blinding of outcome assessment (detection bias) | Unclear |
|  | Unclear how clinicians who enrolled the patients or assessing the outcomes, and the parents were blinded |
| Incomplete outcome data (attrition bias) | Low risk |
|  | Reasons are reported and number of dropouts is balanced |
| Selective reporting (reporting bias) | High risk |
|  | No protocol found, registration number leads to another study. In methods: "all of suspected adverse events were recorded", but only diarrhoea is reported as side effect in results. |
| Other bias | Unclear |
|  | Baseline characteristics only reported for patients that made study end. |

| **Savino 2012** | |
| --- | --- |
| **Bias** | **Authors’ judgement and support for judgement** |
| Random sequence generation (selection bias) | Low risk |
|  | Separate computer generated randomized lists were used for the 3 age groups. |
| Allocation concealment (selection bias) | Low |
|  | Not reported. Contacted authors: central randomization by an external party |
| Blinding of participants and personnel (performance bias) | High risk |
|  | Open label for patients. Only the doctor who performed evaluation was blinded. |
| Blinding of outcome assessment (detection bias) | High risk |
|  | The doctor who performed the evaluation was not involved in the allocation of treatment and remained blinded as to the type of treatment received by patients during the study. However, most outcomes are patient reported and patients/parents were not blinded. |
| Incomplete outcome data (attrition bias) | Low risk |
|  | Reasons are reported and number of dropouts is balanced |
| Selective reporting (reporting bias) | Low risk |
|  | Protocol available. Key efficacy outcomes and safety outcomes reported as per protocol. However in results they have added 'adequate relief' as an outcome. |
| Other bias | Unclear |
|  | Baseline characteristics are not reported for all randomized patients (patients who withdrew before receiving treatment are not described). Contacted authors: no data available. |

| **Shahamat 2016** | |
| --- | --- |
| **Bias** | **Authors’ judgement and support for judgement** |
| Random sequence generation (selection bias) | Low risk |
|  | Block randomisation |
| Allocation concealment (selection bias) | Unclear |
|  | Not mentioned |
| Blinding of participants and personnel (performance bias) | High risk |
|  | Reported that only methodologist and statistician who assessed and analysed were blinded |
| Blinding of outcome assessment (detection bias) | High risk |
|  | Reported that only methodologist and statistician who assessed and analysed were blinded |
| Incomplete outcome data (attrition bias) | Low risk |
|  | Reasons for drop-out are clear and no imbalance |
| Selective reporting (reporting bias) | Unclear |
|  | No protocol found or trial registration details |
| Other bias | Low risk |
|  | Baseline demographics balanced |

| **Strisciuglio 2021** | |
| --- | --- |
| **Bias** | **Authors’ judgement and support for judgement** |
| Random sequence generation (selection bias) | Low risk |
|  | Predefined block randomisation list |
| Allocation concealment (selection bias) | Unclear |
|  | Each centre opened the randomisation letters in sequential order, however unclear if letters were opaque and sealed |
| Blinding of participants and personnel (performance bias) | High risk |
|  | Open label |
| Blinding of outcome assessment (detection bias) | High risk |
|  | Open label |
| Incomplete outcome data (attrition bias) | Unclear |
|  | No reasons reported for the patients who dropped out immediately after randomisation, and not reported how many per group |
| Selective reporting (reporting bias) | Low risk |
|  | Protocol available and followed. Efficacy and safety outcomes were reported as planned. |
| Other bias | High |
|  | Baseline characteristics are not reported for all randomized patients (patients who withdrew immediately after randomisation are not described). After the initial 14 days of treatment, the participants received self-directed variable amounts of the agent, which could have affected the composition of the treatment groups. |

| **Thomson 2007** | |
| --- | --- |
| **Bias** | **Authors’ judgement and support for judgement** |
| Random sequence generation (selection bias) | Low risk |
|  | The random sequence group was computer generated before the start of recruitment using a block size of four patients |
| Allocation concealment (selection bias) | Low risk |
|  | Randomisation numbers were sent to the investigator sites with number stored in sealed code-break envelopes. |
| Blinding of participants and personnel (performance bias) | Low risk |
|  | Says who and how blinded and matched placebo was obtained. |
| Blinding of outcome assessment (detection bias) | Low risk |
|  | Says who and how blinded and matched placebo was obtained. |
| Incomplete outcome data (attrition bias) | Unclear |
|  | Unclear what "advanced straight to period III" means. Unclear if these patients were dropouts during period I. |
| Selective reporting (reporting bias) | Unclear |
|  | No protocol found |
| Other bias | Low risk |
|  | Baseline demographics balanced |

| **Tavassoli 2021** | |
| --- | --- |
| **Bias** | **Authors’ judgement and support for judgement** |
| Random sequence generation (selection bias) | Low risk |
|  | Randomly allocated by the permuted randomisation method (with block sizes of four) |
| Allocation concealment (selection bias) | Low risk |
|  | A random number list generated by using a computer was used to assign participants to two arms. The researcher conducting randomization was not involved in other parts of the study. |
| Blinding of participants and personnel (performance bias) | High risk |
|  | Clinician was not blinded. Only containers were similar, nothing mentioned about taste and appearance. |
| Blinding of outcome assessment (detection bias) | High risk |
|  | Clinician was not blinded. Only containers were similar, nothing mentioned about taste and appearance. |
| Incomplete outcome data (attrition bias) | Low risk |
|  | Reasons for dropout provided. Number of dropouts is balanced |
| Selective reporting (reporting bias) | Unclear |
|  | The only efficacy outcome reported in the protocol was bowel frequency. In the study there were a lot more outcomes, unknown if these outcomes were predefined. |
| Other bias | Low risk |
|  | Baseline characteristics balanced. |

| **Treepongkaruna 2014** | |
| --- | --- |
| **Bias** | **Authors’ judgement and support for judgement** |
| Random sequence generation (selection bias) | Low risk |
|  | Randomisation list of treatment allocation codes prepared by the contract research organisation responsible for operational management of the study. |
| Allocation concealment (selection bias) | Unclear |
|  | Not reported |
| Blinding of participants and personnel (performance bias) | Low risk |
|  | Clear methods of how blinded and who (both patient and doctor) |
| Blinding of outcome assessment (detection bias) | Low risk |
|  | Clear methods of how blinded and who (both patient and doctor) |
| Incomplete outcome data (attrition bias) | Low risk |
|  | Reasons are reported and number of dropouts is balanced. |
| Selective reporting (reporting bias) | Low risk |
|  | Protocol available. Safety outcomes not reported in protocol, but are reported in article. |
| Other bias | Low risk |
|  | Baseline demographics balanced |

| **Urganci 2005** | |
| --- | --- |
| **Bias** | **Authors’ judgement and support for judgement** |
| Random sequence generation (selection bias) | Unclear |
|  | Not reported how randomisation occurred |
| Allocation concealment (selection bias) | Unclear |
|  | Not reported |
| Blinding of participants and personnel (performance bias) | High risk |
|  | Open label |
| Blinding of outcome assessment (detection bias) | High risk |
|  | Open label |
| Incomplete outcome data (attrition bias) | Unclear |
|  | Unclear how many patients reached study end |
| Selective reporting (reporting bias) | High risk |
|  | No protocol found and side effects were not adequately reported even though it was mentioned in the methods that side effects would be monitored. |
| Other bias | Low risk |
|  | Baseline demographics balanced |

| **Uhm 2007** | |
| --- | --- |
| **Bias** | **Authors’ judgement and support for judgement** |
| Random sequence generation (selection bias) | Unclear |
|  | Not reported how randomisation occurred |
| Allocation concealment (selection bias) | Unclear |
|  | Not reported |
| Blinding of participants and personnel (performance bias) | High risk |
|  | Open label |
| Blinding of outcome assessment (detection bias) | High risk |
|  | Open label |
| Incomplete outcome data (attrition bias) | Unclear |
|  | Unclear how many patients reached study end |
| Selective reporting (reporting bias) | Unclear |
|  | No protocol found |
| Other bias | Low risk |
|  | Baseline demographics balanced |

| **Ustundag 2010** | |
| --- | --- |
| **Bias** | **Authors’ judgement and support for judgement** |
| Random sequence generation (selection bias) | Unclear |
|  | Randomization was performed by the use of sequential numbers allocated to the patients at the study entry. |
| Allocation concealment (selection bias) | Unclear |
|  | Not reported |
| Blinding of participants and personnel (performance bias) | High risk |
|  | Open label |
| Blinding of outcome assessment (detection bias) | High risk |
|  | Open label |
| Incomplete outcome data (attrition bias) | High risk |
|  | No reasons for drop-out reported |
| Selective reporting (reporting bias) | High risk |
|  | No protocol found. However, treatment success was given as outcome in methods, however no data reported in results. |
| Other bias | Unclear |
|  | Baseline characteristics only reported for patients that made study end. |

| **Voskuijl 2004** | |
| --- | --- |
| **Bias** | **Authors’ judgement and support for judgement** |
| Random sequence generation (selection bias) | Low risk |
|  | Patients were randomly assigned to received either lactulose or PEG, not described how. Contacted authors: block randomisation |
| Allocation concealment (selection bias) | Low risk |
|  | Unlabelled numbered boxes with unlabelled sachets were prepared by the AMC pharmacy and handed out to patients after randomisation. |
| Blinding of participants and personnel (performance bias) | High risk |
|  | Says double blinded. Not clear who exactly and how both meds were identical (only both unlabelled, not taste and smell etc). Contacted authors: smell/taste etc was not the same. But difficult to make them identical. |
| Blinding of outcome assessment (detection bias) | High risk |
|  | Says double blinded. Not clear who exactly and how both meds were identical (only both unlabelled, not taste and smell etc). Contacted authors: smell/taste etc was not the same. But difficult to make them identical. |
| Incomplete outcome data (attrition bias) | Low risk |
|  | Reasons reported, numbers balanced. Both groups 1 patient 'reason unknown' |
| Selective reporting (reporting bias) | Unclear |
|  | No protocol found |
| Other bias | Low risk |
|  | Baseline demographics balanced |

| **Wang 2007** | |
| --- | --- |
| **Bias** | **Authors’ judgement and support for judgement** |
| Random sequence generation (selection bias) | Low risk |
|  | The statistical software SAS was used to constructed random digit tables |
| Allocation concealment (selection bias) | Low risk |
|  | Central allocation, children received drugs from drug administer. Packages were similar. |
| Blinding of participants and personnel (performance bias) | Unclear |
|  | Same outer packaging and labels for the two drugs. Unclear if taste the same and if researchers were blinded (not reported) |
| Blinding of outcome assessment (detection bias) | Unclear |
|  | Same outer packaging and labels for the two drugs. Unclear if taste the same and if researchers were blinded (not reported) |
| Incomplete outcome data (attrition bias) | Unclear |
|  | No reason for drop out reported of 26 patients. Drop outs were equally divided. For the remaining drop outs the reasons were not reported per group. |
| Selective reporting (reporting bias) | Unclear |
|  | No protocol found |
| Other bias | Low risk |
|  | Baseline demographics balanced |

***Non-pharmacological therapy studies***

| **Abediny 2016*** | |
| --- | --- |
| **Bias** | **Authors’ judgement and support for judgement** |
| Random sequence generation (selection bias) | Unclear |
|  | Not reported how in the abstract |
| Allocation concealment (selection bias) | Unclear |
|  | Not reported how in the abstract |
| Blinding of participants and personnel (performance bias) | High |
|  | Single blinded. Parents not blinded. |
| Blinding of outcome assessment (detection bias) | High |
|  | Single blinded. Parents not blinded. |
| Incomplete outcome data (attrition bias) | Unclear |
|  | Not reported in the abstract |
| Selective reporting (reporting bias) | Unclear |
|  | No protocol found |
| Other bias | Unclear |
|  | Baseline demographics not reported in abstract |

*Only abstract was in English. Not able to translate the full text.

| **Banaszkiewicz 2005** | |
| --- | --- |
| **Bias** | **Authors’ judgement and support for judgement** |
| Random sequence generation (selection bias) | Low risk |
|  | The allocation sequence and randomization list were computer-generated by investigators at the Medical University of Warsaw. |
| Allocation concealment (selection bias) | Unclear |
|  | Allocation concealment was achieved by the use of study products with similar appearances and tastes, which were packed identically and which were indistinguishable from each other. Throughout the duration of the study, all investigators, participants, outcome assessors, and data analysts were blinded to the assigned treatment |
| Blinding of participants and personnel (performance bias) | Low risk |
|  | Study products were packed identically and which were indistinguishable from each other. Throughout the duration of the study, all investigators, participants, outcome assessors, and data analysts were blinded to the assigned treatment |
| Blinding of outcome assessment (detection bias) | Low risk |
|  | Study products were packed identically and which were indistinguishable from each other. Throughout the duration of the study, all investigators, participants, outcome assessors, and data analysts were blinded to the assigned treatment |
| Incomplete outcome data (attrition bias) | Low risk |
|  | Drop outs reported, with reason. No imbalance between groups. |
| Selective reporting (reporting bias) | Unclear |
|  | No protocol or trial registration found. Primary and secondary outcomes reported including statistical analysis plan. |
| Other bias | Low risk |
|  | No baseline differences between groups |

| **Basturk 2007** | |
| --- | --- |
| **Bias** | **Authors’ judgement and support for judgement** |
| Random sequence generation (selection bias) | Unclear |
|  | Says random but now how random occurred |
| Allocation concealment (selection bias) | Low risk |
|  | Code numbers of drugs were unknown to doctor, nurse and patient. Only manufacturer knew. |
| Blinding of participants and personnel (performance bias) | Low risk |
|  | Drugs that were completely same in colour, smell, taste, and packaging properties but had one of the two different code numbers on them were used. The ingredients of the drugs were unknown to the doctor, nurse, and the patient, and which code number included which ingredient was known to the manufacturer only. |
| Blinding of outcome assessment (detection bias) | Low risk |
|  | Drugs that were completely same in colour, smell, taste, and packaging properties but had one of the two different code numbers on them were used. The ingredients of the drugs were unknown to the doctor, nurse, and the patient, and which code number included which ingredient was known to the manufacturer only. |
| Incomplete outcome data (attrition bias) | Unclear |
|  | Drop outs reported, no reasons provided. No imbalance between groups. |
| Selective reporting (reporting bias) | Unclear |
|  | No protocol found or trial registration. Primary and secondary outcomes as reported in methods. |
| Other bias | Low risk |
|  | No baseline differences between groups |

| **Blanco-Diaz 2020** | |
| --- | --- |
| **Bias** | **Authors’ judgement and support for judgement** |
| Random sequence generation (selection bias) | Low risk |
|  | Computer generated random sequence |
| Allocation concealment (selection bias) | Low risk |
|  | Sealed opaque envelopes |
| Blinding of participants and personnel (performance bias) | High risk |
|  | Open label |
| Blinding of outcome assessment (detection bias) | High risk |
|  | Open label |
| Incomplete outcome data (attrition bias) | Unclear |
|  | Unclear. Only report compliance and attendance rates. At every outcome measurement point, the number of patients differ. Unclear if patients were lost to follow-up. |
| Selective reporting (reporting bias) | High risk |
|  | No protocol found. They do not mention anything about recording side effects during the study (only the Symptom Severity Score), but do say in their conclusion that Manual Therapy has no side effects. |
| Other bias | Low risk |
|  | Baseline demographics balanced |

| **Bongers 2007** | |
| --- | --- |
| **Bias** | **Authors’ judgement and support for judgement** |
| Random sequence generation (selection bias) | Low risk |
|  | Computer randomisation |
| Allocation concealment (selection bias) | Unclear |
|  | Not reported |
| Blinding of participants and personnel (performance bias) | Low risk |
|  | Formula cans were labelled with codes to mask identity of the study feedings. Neither the parent nor the physicians were aware of the composition of the formula until the entire study was completed. Taste was made the same for both. |
| Blinding of outcome assessment (detection bias) | Low risk |
|  | Formula cans were labelled with codes to mask identity of the study feedings. Neither the parent nor the physicians were aware of the composition of the formula until the entire study was completed. Taste was made the same for both. |
| Incomplete outcome data (attrition bias) | Low risk |
|  | Drop outs reported, reasons provided. No imbalance between groups. |
| Selective reporting (reporting bias) | Unclear |
|  | No protocol found or trial registration. Primary and secondary outcomes as reported in methods. |
| Other bias | Low risk |
|  | No baseline differences between groups |

| **Bourkheili 2021** | |
| --- | --- |
| **Bias** | **Authors’ judgement and support for judgement** |
| Random sequence generation (selection bias) | Low risk |
|  | Computer-generated randomisation code |
| Allocation concealment (selection bias) | Unclear |
|  | Not mentioned |
| Blinding of participants and personnel (performance bias) | High risk |
|  | Open label |
| Blinding of outcome assessment (detection bias) | High risk |
|  | Open label |
| Incomplete outcome data (attrition bias) | Low risk |
|  | Only one drop out in CG, but no reason reported. Only one patient will not have big impact on the results. |
| Selective reporting (reporting bias) | High risk |
|  | Protocol found. But primary outcome of the paper (treatment success) was not reported in protocol. Also no safety data reported, but in methods they do describe 'parents were advised to contact the therapist if their children experienced any signs and symptoms such as nausea, vomiting, diarrhoea, abdominal pain and skin symptoms.' |
| Other bias | Low risk |
|  | Baseline demographics balanced. Do miss data of the one patient that was lost to follow up, but only one will not have big impact on the results. |

| **Bu 2007** | |
| --- | --- |
| **Bias** | **Authors’ judgement and support for judgement** |
| Random sequence generation (selection bias) | Low risk |
|  | Computer-generated randomization list |
| Allocation concealment (selection bias) | Unclear |
|  | Not reported |
| Blinding of participants and personnel (performance bias) | Low risk |
|  | Three interventions with similar appearances and placed into identical capsules. |
| Blinding of outcome assessment (detection bias) | Low risk |
|  | Three interventions with similar appearances and placed into identical capsules |
| Incomplete outcome data (attrition bias) | Unclear |
|  | Numbers per group reported, no imbalance. Reasons provided, however unclear which reasons belong to which patient/group. Two patients suffered from actue gastroenteritis, unclear in which group. |
| Selective reporting (reporting bias) | Unclear |
|  | No protocol found or trial registration. |
| Other bias | Low risk |
|  | Baseline demographics balanced |

| **Cai 2018** | |
| --- | --- |
| **Bias** | **Authors’ judgement and support for judgement** |
| Random sequence generation (selection bias) | Low risk |
|  | Block randomisation |
| Allocation concealment (selection bias) | Low risk |
|  | Sealed opaque envelopes |
| Blinding of participants and personnel (performance bias) | Low risk |
|  | Double blinded. “We blinded the random result twice, named the drugs as drug A and drug B instead of the real name in the first level, and named the groups as group 1 and group 2 instead of treatment or placebo group in the second level. The second level could be unblinded for analysis, while the first level should be unblinded until trial summary.” |
| Blinding of outcome assessment (detection bias) | Low risk |
|  | Double blinded. “We blinded the random result twice, named the drugs as drug A and drug B instead of the real name in the first level, and named the groups as group 1 and group 2 instead of treatment or placebo group in the second level. The second level could be unblinded for analysis, while the first level should be unblinded until trial summary.” |
| Incomplete outcome data (attrition bias) | Unclear |
|  | Only reasons provided for the drop outs was: “Forty-eight cases in FAS (full analysis set) were excluded from the PPS (per protocol set) due to major protocol violations and poor medicine compliance” Dropout in herbal group: 34/360, dropout in placebo: 16/120. No imbalance, but no exact reasons provided and not per group/patient. |
| Selective reporting (reporting bias) | Unclear |
|  | Protocol registered and key safety data and outcomes reported. However for their secondary efficacy results the number of patients are lower than the number of patients stated for either the FAS or PPS analysis. Unclear why the remaining patients were not included as they stated in their methods that “Efficiency measure data were analysed based on FAS and PPS”. |
| Other bias | Low risk |
|  | Baseline demographics balanced. |

| **Canbulat 2017** | |
| --- | --- |
| **Bias** | **Authors’ judgement and support for judgement** |
| Random sequence generation (selection bias) | Unclear |
|  | Not reported |
| Allocation concealment (selection bias) | Unclear |
|  | Not reported |
| Blinding of participants and personnel (performance bias) | High |
|  | Not blinded |
| Blinding of outcome assessment (detection bias) | High |
|  | Not blinded |
| Incomplete outcome data (attrition bias) | Low risk |
|  | Drop outs reported, reasons provided. No imbalance between groups. |
| Selective reporting (reporting bias) | Unclear |
|  | No protocol found |
| Other bias | Low risk |
|  | Baseline demographics balanced. |

| **Cassettari 2019** | |
| --- | --- |
| **Bias** | **Authors’ judgement and support for judgement** |
| Random sequence generation (selection bias) | Low risk |
|  | Subjects were randomly assigned into five treatment groups by a mathematical algorithm |
| Allocation concealment (selection bias) | Unclear |
|  | Not reported |
| Blinding of participants and personnel (performance bias) | Low risk |
|  | Patients and personnel blinded. Biomass looked identical as a thick, white, homogenous mass. No substantial variation in colour, taste, and smell. |
| Blinding of outcome assessment (detection bias) | Low risk |
|  | Patients and personnel blinded. Biomass looked identical as a thick, white, homogenous mass. No substantial variation in colour, taste, and smell. |
| Incomplete outcome data (attrition bias) | Low risk |
|  | All drop outs per group reported with reasons. |
| Selective reporting (reporting bias) | Low risk |
|  | Key outcomes and safety data reported per protocol. |
| Other bias | Low risk |
|  | Baseline demographics balanced |

| **Castilla 2021 (abstract only)** | |
| --- | --- |
| **Bias** | **Authors’ judgement and support for judgement** |
| Random sequence generation (selection bias) | Unclear |
|  | Reported “simple random allocation", however unclear how randomisation occurred. |
| Allocation concealment (selection bias) | Unclear |
|  | Not reported. |
| Blinding of participants and personnel (performance bias) | Unclear |
|  | Not reported. |
| Blinding of outcome assessment (detection bias) | Unclear |
|  | Not reported. |
| Incomplete outcome data (attrition bias) | Low risk |
|  | All patients who were randomised, were included in the final analyses. |
| Selective reporting (reporting bias) | Unclear |
|  | No protocol found |
| Other bias | Unclear |
|  | No baseline characteristic data reported. |

| **Chmielewska 2011** | |
| --- | --- |
| **Bias** | **Authors’ judgement and support for judgement** |
| Random sequence generation (selection bias) | Low risk |
|  | Block randomization, with a block size of 6, was done with a computer-generated random number list prepared by an investigator with no clinical involvement in the trial. |
| Allocation concealment (selection bias) | Low risk |
|  | The randomization sequence was concealed until all data were analysed. Study intervention products were prepared in sachets centrally by the hospital pharmacy at the Medical University of Warsaw by independent personnel not involved in the conduct of the trial. |
| Blinding of participants and personnel (performance bias) | Unclear |
|  | The active product and placebo were packaged in identical sachets and labelled with one of two codes. The appearance and texture of the dry products were identical. When mixed with water, the preparation of GNN turned into a substance of jelly-like consistency; however, this only happened if the solution was not consumed within a few minutes, which was the recommended time limit for consumption.  Contact with authors confirmed that after mixing with water, the consistence of placebo was not like the one of glucomannan (if not consumed directly after preparation). The participants were not informed of the viscosity of the preparations. |
| Blinding of outcome assessment (detection bias) | Low risk |
|  | Both the participants and researchers conducting the study, one of whom also performed data analysis, were blinded. packaging was identical, dry products were also identical. |
| Incomplete outcome data (attrition bias) | Low risk |
|  | Flow chart. Drop outs reported with reasons. No imbalance between groups. |
| Selective reporting (reporting bias) | Low risk |
|  | Protocol available. Key safety data and efficacy outcomes reported as per protocol. |
| Other bias | Low risk |
|  | Baseline demographics balanced. |

| **Clarke 2009** | |
| --- | --- |
| **Bias** | **Authors’ judgement and support for judgement** |
| Random sequence generation (selection bias) | Unclear |
|  | Says randomised, but not reported how |
| Allocation concealment (selection bias) | Unclear |
|  | Not reported |
| Blinding of participants and personnel (performance bias) | High risk |
|  | Unclear what the placebo treatment was and who were blinded. Treating physician could not be blinded, therefore high risk. |
| Blinding of outcome assessment (detection bias) | Unclear |
|  | Unclear what the placebo treatment was and who were blinded. Patients were blinded. |
| Incomplete outcome data (attrition bias) | Low risk |
|  | No drop outs |
| Selective reporting (reporting bias) | Unclear risk |
|  | No protocol found |
| Other bias | Low risk |
|  | Baseline demographics balanced. |

| **Coccorullo 2010** | |
| --- | --- |
| **Bias** | **Authors’ judgement and support for judgement** |
| Random sequence generation (selection bias) | Low risk |
|  | Computer generated |
| Allocation concealment (selection bias) | High risk |
|  | Not reported in paper.  Answer author: The randomization was performed in person by the study personnel. All the study personnel was blinded to the intervention, since the sponsor provided both the product and the placebo in anonymous kits that had to be allocated sequentially, according to the randomization list. The study personnel had a list with sequential numbers and kit codes to assign. The study personnel had access to the randomization list. |
| Blinding of participants and personnel (performance bias) | Low risk |
|  | Answer author: The pharmacy was not involved. All the study personnel was blinded to the intervention, since the sponsor provided both the product and the placebo in anonymous kits that had to be allocated sequentially, according to the randomization list. The study personnel had a list with sequential numbers and kit codes to assign. The study personnel had access to the randomization list. |
| Blinding of outcome assessment (detection bias) | Low risk |
|  | Answer author: The pharmacy was not involved. All the study personnel was blinded to the intervention, since the sponsor provided both the product and the placebo in anonymous kits that had to be allocated sequentially, according to the randomization list. The study personnel had a list with sequential numbers and kit codes to assign. The study personnel had access to the randomization list. |
| Incomplete outcome data (attrition bias) | Low risk |
|  | No drop outs reported. |
| Selective reporting (reporting bias) | Low risk |
|  | Protocol. Key safety data and outcomes reported. |
| Other bias | Low risk |
|  | Baseline demographics balanced. |

| **Croffie 2005** | |
| --- | --- |
| **Bias** | **Authors’ judgement and support for judgement** |
| Random sequence generation (selection bias) | Unclear |
|  | Says randomised, but not reported how |
| Allocation concealment (selection bias) | Unclear |
|  | Not reported |
| Blinding of participants and personnel (performance bias) | High risk |
|  | No mention of blinding in text or language akin to blinding. |
| Blinding of outcome assessment (detection bias) | High risk |
|  | No mention of blinding in text or language akin to blinding. |
| Incomplete outcome data (attrition bias) | Low risk |
|  | All drop outs reported with reasons. |
| Selective reporting (reporting bias) | Unclear |
|  | No protocol found |
| Other bias | Low risk |
|  | Baseline demographics balanced. |

| **De Abreu 2021** | |
| --- | --- |
| **Bias** | **Authors’ judgement and support for judgement** |
| Random sequence generation (selection bias) | Low risk |
|  | Randomization was performed by shuffling blocks of 4 sealed, sequentially numbered brown envelopes. |
| Allocation concealment (selection bias) | Low risk |
|  | Allocation was performed by independent research professional. Sealed opaque envelopes were used according to the randomization sequence. |
| Blinding of participants and personnel (performance bias) | High risk |
|  | Sham PTNS. However, treating physician needed to know the group allocation, because the electrodes needed to be placed on the scapular (CG) region instead of sacral (IG). Treating physician was not part of rest of the study. |
| Blinding of outcome assessment (detection bias) | Low risk |
|  | Parents, patients, and post-treatment evaluator were blinded to group allocation. Sham treatment. Researcher who carried out post-treatment evaluation was unaware of treatment allocation, some for data analysis. |
| Incomplete outcome data (attrition bias) | Low risk |
|  | No imbalance, reason for dropout provided |
| Selective reporting (reporting bias) | High risk |
|  | Protocol found. No safety data reported |
| Other bias | Low risk |
|  | Baseline demographics balanced. See supplementary files. |

| **Dehghani 2012** | |
| --- | --- |
| **Bias** | **Authors’ judgement and support for judgement** |
| Random sequence generation (selection bias) | Low risk |
|  | Computer generated |
| Allocation concealment (selection bias) | Unclear |
|  | Not reported |
| Blinding of participants and personnel (performance bias) | High risk |
|  | Patients and parents were not blinded, only paediatric gastroenterologist who evaluated the protocols at the end of treatment was blinded. Unclear if researcher was blinded. |
| Blinding of outcome assessment (detection bias) | High risk |
|  | Patients and parents were not blinded, only paediatric gastroenterologist who evaluated the protocols at the end of treatment was blinded. Unclear if researcher was blinded. |
| Incomplete outcome data (attrition bias) | Low risk |
|  | No drop outs reported. |
| Selective reporting (reporting bias) | Unclear |
|  | No protocol found or trial registration details. |
| Other bias | Low risk |
|  | Baseline demographics balanced. |

| **Dehghani 2019** | |
| --- | --- |
| **Bias** | **Authors’ judgement and support for judgement** |
| Random sequence generation (selection bias) | Low risk |
|  | Block randomisation |
| Allocation concealment (selection bias) | Low risk |
|  | Word ‘allocation’ used to describe joining group without further discussion. Contacted author, answer: allocation concealment was done by numbered drug containers. |
| Blinding of participants and personnel (performance bias) | Low risk |
|  | Drug and placebo prepared with similar organoleptic properties; packaged in identical containers. Patients and personnel blinded. |
| Blinding of outcome assessment (detection bias) | Low risk |
|  | Drug and placebo prepared with similar organoleptic properties; packaged in identical containers. Patients and personnel blinded. |
| Incomplete outcome data (attrition bias) | Low risk |
|  | Flow of patients including randomised and assessed, drop outs reported with reasons given in flow chart. |
| Selective reporting (reporting bias) | Low risk |
|  | Key outcomes and safety data reported per protocol. |
| Other bias | Low risk |
|  | Baseline demographics balanced. |

| **Engelenburg 2017** | |
| --- | --- |
| **Bias** | **Authors’ judgement and support for judgement** |
| Random sequence generation (selection bias) | Low risk |
|  | Computer generated |
| Allocation concealment (selection bias) | Unclear risk |
|  | Not clear if allocation was concealed. |
| Blinding of participants and personnel (performance bias) | High risk |
|  | Open label. Practitioners and patients were not blinded (not possible). |
| Blinding of outcome assessment (detection bias) | High risk |
|  | Open label. Practitioners and patients were not blinded (not possible). Study used patient reported outcomes. Outcome assessor was blinded and independent. |
| Incomplete outcome data (attrition bias) | Low risk |
|  | Flow chart. Drop outs reported with reasons. |
| Selective reporting (reporting bias) | Low |
|  | Trial registration numbers reported and protocol found. Primary outcome reported as per protocol. |
| Other bias | Low risk |
|  | Baseline demographics balanced. |

| **Esmaeilidooki 2016** | |
| --- | --- |
| **Bias** | **Authors’ judgement and support for judgement** |
| Random sequence generation (selection bias) | Unclear |
|  | Stated ‘Simple Randomisation' without specified method. |
| Allocation concealment (selection bias) | Unclear |
|  | Stated 'Random allocation' without specified method. |
| Blinding of participants and personnel (performance bias) | High risk |
|  | Open label |
| Blinding of outcome assessment (detection bias) | High risk |
|  | Open label |
| Incomplete outcome data (attrition bias) | Low risk |
|  | All drop-outs reported per group as represented in flow chart |
| Selective reporting (reporting bias) | Low risk |
|  | Protocol available. Key safety data and outcomes reported. |
| Other bias | Low risk |
|  | Baseline demographics balanced. |

| **Fabrizio 2022** | |
| --- | --- |
| **Bias** | **Authors’ judgement and support for judgement** |
| Random sequence generation (selection bias) | Low risk |
|  | Computer generated randomization |
| Allocation concealment (selection bias) | Low risk |
|  | Intervention schedules were provided in sealed envelopes for each study site. Study formula was assigned by opening the next sequential envelope at the study site. |
| Blinding of participants and personnel (performance bias) | Low risk |
|  | Study formulas, each designated by two unique codes known only to the sponsor, were dispensed to parents at randomization. Neither the product labels nor the sealed envelopes allowed direct unblinding by the study site. Personnel responsible for monitoring the study were also blinded to study product identification. |
| Blinding of outcome assessment (detection bias) | Low risk |
|  | Study formulas, each designated by two unique codes known only to the sponsor, were dispensed to parents at randomization. Neither the product labels nor the sealed envelopes allowed direct unblinding by the study site. Personnel responsible for monitoring the study were also blinded to study product identification. |
| Incomplete outcome data (attrition bias) | Unclear |
|  | No imbalance, but Reasons for drop out unclear. Ask authors. |
| Selective reporting (reporting bias) | Unclear |
|  | No protocol found. Key outcomes reported as described in methods. |
| Other bias | Low risk |
|  | Baseline demographics balanced |

| **Gan 2022** | |
| --- | --- |
| **Bias** | **Authors’ judgement and support for judgement** |
| Random sequence generation (selection bias) | Unclear |
|  | Not reported |
| Allocation concealment (selection bias) | Unclear |
|  | Not reported |
| Blinding of participants and personnel (performance bias) | High risk |
|  | Single blinded. Assume only the patients were blinded, placebo controlled. No mention of how and who blinded and if placebo was matched. |
| Blinding of outcome assessment (detection bias) | High risk |
|  | Single blinded. Assume only the patients were blinded, placebo controlled. No mention of how and who blinded and if placebo was matched. |
| Incomplete outcome data (attrition bias) | Unclear |
|  | Unclear why patients 'incorrectly enrolled' and if they had already received treatment or not, in results section they say 'incomplete data'. |
| Selective reporting (reporting bias) | Low risk |
|  | Trial registration number reported. They do report trial was registered at clinicaltrials.gov. That is not correct. Instead it is registered at Chinese trial registry. Outcomes match protocol. |
| Other bias | Low risk |
|  | Baseline demographics balanced. |

| **Guerra 2011** | |
| --- | --- |
| **Bias** | **Authors’ judgement and support for judgement** |
| Random sequence generation (selection bias) | Low risk |
|  | Computer generated |
| Allocation concealment (selection bias) | Unclear |
|  | Allocation sequence computer generated, but unclear if allocation was concealed. |
| Blinding of participants and personnel (performance bias) | Low risk |
|  | The two products, goat yogurt with or without B. longum were identical in weight, colour, smell, taste and package. All doctors and children involved were unaware of the treatment administered. |
| Blinding of outcome assessment (detection bias) | Low risk |
|  | The two products, goat yogurt with or without B. longum were identical in weight, colour, smell, taste and package. All doctors and children involved were unaware of the treatment administered. |
| Incomplete outcome data (attrition bias) | Low risk |
|  | Only 1 withdrawal in the control group, no reason provided. But only 1, so not expected to have impacted the results. |
| Selective reporting (reporting bias) | Unclear |
|  | No protocol found or trial registration details. |
| Other bias | Low risk |
|  | Baseline demographics balanced. |

| **Iacono 1998** | |
| --- | --- |
| **Bias** | **Authors’ judgement and support for judgement** |
| Random sequence generation (selection bias) | Low risk |
|  | Computer generated |
| Allocation concealment (selection bias) | Low risk |
|  | The milk was supplied in bottles coded A or B by the hospital dispensary |
| Blinding of participants and personnel (performance bias) | Unclear |
|  | Says double blinded, but unclear how both treatments were matched. Only that the bottles were coded A or B. Not if taste, smell etc were matched. Researchers were unaware of treatment assignment. |
| Blinding of outcome assessment (detection bias) | Unclear |
|  | Says double blinded, but unclear how both treatments were matched. Only that the bottles were coded A or B. Not if taste, smell etc were matched. Researchers were unaware of treatment assignment. |
| Incomplete outcome data (attrition bias) | Low risk |
|  | No dropouts |
| Selective reporting (reporting bias) | Unclear |
|  | No protocol found |
| Other bias | Unclear |
|  | Data reported for all patients (cross-over study). Therefore unclear how baseline characteristics were divided between the two groups. |

| **Imanieh 2022** | |
| --- | --- |
| **Bias** | **Authors’ judgement and support for judgement** |
| Random sequence generation (selection bias) | Low risk |
|  | Permuted block randomization was used for randomly allocating the participants to the two groups. |
| Allocation concealment (selection bias) | Low risk |
|  | Allocation concealment was achieved using the dark envelope method. |
| Blinding of participants and personnel (performance bias) | Low risk |
|  | Containers with the same shape, colour, and weight, so that the containers and solutions would not be distinguishable (no mention of taste). The patients, physicians, and the analyst were not aware of the ingredients of the solutions, only the pharmacist. |
| Blinding of outcome assessment (detection bias) | Low risk |
|  | Containers with the same shape, colour, and weight, so that the containers and solutions would not be distinguishable (no mention of taste). The patients, physicians, and the analyst were not aware of the ingredients of the solutions, only the pharmacist |
| Incomplete outcome data (attrition bias) | Low risk |
|  | Reasons provided. No imbalance in number of patients per group (6 vs 2) |
| Selective reporting (reporting bias) | Unclear |
|  | Primary outcome in paper: not fulfilling Rome IV criteria anymore. In protocol primary outcomes are all Rome IV criteria (and more) separately. |
| Other bias | High risk |
|  | Most of the baseline demographics balanced. Duration of functional constipation differs between groups: 74% of IG and 44% CG had been suffering from functional constipation for more than 12 months. |

| **Jadresin 2018** | |
| --- | --- |
| **Bias** | **Authors’ judgement and support for judgement** |
| Random sequence generation (selection bias) | Low risk |
|  | Random allocation software |
| Allocation concealment (selection bias) | Low risk |
|  | Sealed opaque envelopes |
| Blinding of participants and personnel (performance bias) | Low risk |
|  | Both interventions were of the same taste, colour, smell, identical packaging, produced by producer not involved with the rest of the study. All study personnel, parents and guardians were unaware of the group assignments |
| Blinding of outcome assessment (detection bias) | Low risk |
|  | Both interventions were of the same taste, colour, smell, identical packaging, produced by producer not involved with the rest of the study. All study personnel, parents and guardians were unaware of the group assignments |
| Incomplete outcome data (attrition bias) | Low risk |
|  | Drop outs reported, with reason. No imbalance. |
| Selective reporting (reporting bias) | High risk |
|  | Protocol is for both functional abdominal pain and FC. Paper only reports FC patients. Primary outcomes in protocol do not match primary outcomes in paper. |
| Other bias | Unclear |
|  | No difference between age and gender between IG and CG. However, age and gender were the only baseline characteristics that were provided. More information needed. |

| **Khan 2020** | |
| --- | --- |
| **Bias** | **Authors’ judgement and support for judgement** |
| Random sequence generation (selection bias) | Low risk |
|  | Random number spreadsheet |
| Allocation concealment (selection bias) | Unclear |
|  | Not reported |
| Blinding of participants and personnel (performance bias) | High risk |
|  | Open label |
| Blinding of outcome assessment (detection bias) | High risk |
|  | Open label |
| Incomplete outcome data (attrition bias) | Unclear |
|  | Not reported |
| Selective reporting (reporting bias) | Unclear |
|  | No protocol found or trial registration details. |
| Other bias | Unclear |
|  | Baseline demographics not reported per group, but for whole cohort. |

| **Khodadad 2010** | |
| --- | --- |
| **Bias** | **Authors’ judgement and support for judgement** |
| Random sequence generation (selection bias) | Unclear |
|  | Randomisation was generated by their biostatistics consultant. However, unclear how randomisation occurred. |
| Allocation concealment (selection bias) | Unclear |
|  | Not reported |
| Blinding of participants and personnel (performance bias) | Unclear |
|  | Physicians and nurses were blinded. Bottles and sachets were similar in shape, taste and colour. Only label indicating A or B. However: Group A received 1.5 ml/kg/day oral liquid paraffin plus placebo, group B received 1 sachet synbiotic per day, Group C received 1.5 ml/kg/day oral liquid paraffin and 1 sachet synbiotic per day. Group B seemed to only receive a sachet with synbiotics, no placebo for laxative. |
| Blinding of outcome assessment (detection bias) | Unclear |
|  | Physicians and nurses were blinded. Bottles and sachets were similar in shape, taste and colour. Only label indicating A or B. However: Group A received 1.5 ml/kg/day oral liquid paraffin plus placebo, group B received 1 sachet synbiotic per day, Group C received 1.5 ml/kg/day oral liquid paraffin and 1 sachet synbiotic per day. Group B seemed to only receive a sachet with synbiotics, no placebo for laxative. |
| Incomplete outcome data (attrition bias) | Low risk |
|  | All drop-outs reported per group as represented in flow chart with reasons. |
| Selective reporting (reporting bias) | Low risk |
|  | Key outcomes (efficacy and safety) reported as per protocol |
| Other bias | Low risk |
|  | Baseline characteristics balanced |

| **Kokke 2008** | |
| --- | --- |
| **Bias** | **Authors’ judgement and support for judgement** |
| Random sequence generation (selection bias) | Low risk |
|  | Use of sequential numbers allocated to the patients at study entry and coordinated by the logistics manager of Numico Research using a block design. |
| Allocation concealment (selection bias) | Unclear |
|  | Not mentioned |
| Blinding of participants and personnel (performance bias) | Low risk |
|  | Clear description of how both interventions were packed and prepared. Products could not be distinguished. |
| Blinding of outcome assessment (detection bias) | Low risk |
|  | Clear description of how both interventions were packed and prepared. Products could not be distinguished. |
| Incomplete outcome data (attrition bias) | Low risk |
|  | All drop-outs reported per group as represented in flow chart |
| Selective reporting (reporting bias) | Unclear |
|  | No protocol |
| Other bias | Unclear |
|  | Baseline characteristics reported for patients who reached end of study. |

| **Kubota 2010** | |
| --- | --- |
| **Bias** | **Authors’ judgement and support for judgement** |
| Random sequence generation (selection bias) | Low risk |
|  | Block randomisation |
| Allocation concealment (selection bias) | Unclear |
|  | Not mentioned |
| Blinding of participants and personnel (performance bias) | Low risk |
|  | All caregivers, patients, research staff, and physicians were blinded to which treatment group the patients belonged. Identical matching placebo and probiotic. bottles were matched and taste/texture were matched. |
| Blinding of outcome assessment (detection bias) | Low risk |
|  | All caregivers, patients, research staff, and physicians were blinded to which treatment group the patients belonged. Identical matching placebo and probiotic. bottles were matched and taste/texture were matched. |
| Incomplete outcome data (attrition bias) | Unclear |
|  | Unclear to which group the excluded patients belonged to. Only 3 patients. |
| Selective reporting (reporting bias) | Unclear |
|  | No protocol found or trial registration details |
| Other bias | Low risk |
|  | Baseline characteristics balanced |

| **Ladi-Seyedian 2020** | |
| --- | --- |
| **Bias** | **Authors’ judgement and support for judgement** |
| Random sequence generation (selection bias) | Low risk |
|  | Block randomisation |
| Allocation concealment (selection bias) | Unclear |
|  | Not reported |
| Blinding of participants and personnel (performance bias) | High risk |
|  | Open label |
| Blinding of outcome assessment (detection bias) | High risk |
|  | Open label |
| Incomplete outcome data (attrition bias) | Low risk |
|  | No dropouts |
| Selective reporting (reporting bias) | Unclear |
|  | No protocol found or trial registration details |
| Other bias | Low risk |
|  | Baseline characteristics balanced |

| **Lee 2022** | |
| --- | --- |
| **Bias** | **Authors’ judgement and support for judgement** |
| Random sequence generation (selection bias) | Low risk |
|  | Randomization was implemented automatically using Random Allocation Software 2.0 (Informer Technologies, Inc, Dallas, TX, USA) with a random block size |
| Allocation concealment (selection bias) | Unclear |
|  | Not reported |
| Blinding of participants and personnel (performance bias) | High risk |
|  | Open label |
| Blinding of outcome assessment (detection bias) | High risk |
|  | Open label |
| Incomplete outcome data (attrition bias) | High risk |
|  | High amount of drop-outs, imbalance > 20% and reasons not specified for each group |
| Selective reporting (reporting bias) | Low risk |
|  | Protocol found. Key outcomes reported as per protocol. Safety data also reported in study. |
| Other bias | Low risk |
|  | "There were no differences in age, disease duration, or other clinical characteristics among the 3 groups." There were sex differences between combination therapy and s. boulardii. They adjusted for sex in their Cox Regression analysis. |

| **Loening-Baucke 1990** | |
| --- | --- |
| **Bias** | **Authors’ judgement and support for judgement** |
| Random sequence generation (selection bias) | Unclear |
|  | Sealed envelopes with 4x4-inch cards indicating either conventional therapy alone or conventional therapy with biofeedback training were used for randomization. More information is needed. |
| Allocation concealment (selection bias) | Low risk |
|  | Sealed envelopes |
| Blinding of participants and personnel (performance bias) | High risk |
|  | Open label |
| Blinding of outcome assessment (detection bias) | High risk |
|  | Open label |
| Incomplete outcome data (attrition bias) | Low risk |
|  | Drop outs reported with reason, no imbalance. |
| Selective reporting (reporting bias) | Unclear |
|  | No protocol found or trial registration details |
| Other bias | Low risk |
|  | Baseline characteristics balanced. There were more girls in biofeedback group compared to conventional (9/22 vs 1/19), but treatment success between boys and girls did not differ in the biofeedback group. For standard treatment sex has not a big impact. |

| **Loening-Baucke 2004** | |
| --- | --- |
| **Bias** | **Authors’ judgement and support for judgement** |
| Random sequence generation (selection bias) | Unclear |
|  | Only reported that patients were randomised by envelope. More information is needed. |
| Allocation concealment (selection bias) | Unclear |
|  | With envelopes, but not mentioned whether the envelopes were sealed or not. |
| Blinding of participants and personnel (performance bias) | Unclear |
|  | Says double blinded, but unclear who exactly were blinded. Both treatments had similar capsules, but unsure about same taste etc. |
| Blinding of outcome assessment (detection bias) | Unclear |
|  | Says double blinded, but unclear who exactly were blinded. Both treatments had similar capsules, but unsure about same taste etc. |
| Incomplete outcome data (attrition bias) | Unclear |
|  | Drop outs reported without reason, no imbalance (6 vs 7) |
| Selective reporting (reporting bias) | Unclear |
|  | No protocol found or trial registration details |
| Other bias | Low risk |
|  | Baseline characteristics balanced |

| **Lojanatorn 2023** | |
| --- | --- |
| **Bias** | **Authors’ judgement and support for judgement** |
| Random sequence generation (selection bias) | Low risk |
|  | A computer generated list |
| Allocation concealment (selection bias) | Low risk |
|  | Allocation was performed by an independent pharmacy |
| Blinding of participants and personnel (performance bias) | High risk |
|  | Could not create identical containers (see discussion) |
| Blinding of outcome assessment (detection bias) | High risk |
|  | Could not create identical containers (see discussion) |
| Incomplete outcome data (attrition bias) | Low risk |
|  | No imbalance >20% and reason of dropout provided |
| Selective reporting (reporting bias) | Unclear |
|  | States that trial registration is published retrospectively. Trial registered retrospectively (at 19 February 2021 and first recruitment 1 February 2021). |
| Other bias | Low risk |
|  | Baseline demographics balanced |

| **Mao 2015** | |
| --- | --- |
| **Bias** | **Authors’ judgement and support for judgement** |
| Random sequence generation (selection bias) | Unclear |
|  | Says random, but not how random occurred |
| Allocation concealment (selection bias) | Unclear |
|  | Not reported |
| Blinding of participants and personnel (performance bias) | High risk |
|  | Open Label Trial |
| Blinding of outcome assessment (detection bias) | High risk |
|  | As Above |
| Incomplete outcome data (attrition bias) | Unclear |
|  | Not reported |
| Selective reporting (reporting bias) | Unclear |
|  | No protocol found or trial registration details |
| Other bias | Low risk |
|  | Baseline characteristics balanced |

| **Mozaffarpur 2012** | |
| --- | --- |
| **Bias** | **Authors’ judgement and support for judgement** |
| Random sequence generation (selection bias) | Unclear |
|  | Systematic randomisation |
| Allocation concealment (selection bias) | Unclear |
|  | Not reported |
| Blinding of participants and personnel (performance bias) | High risk |
|  | Open Label Trial |
| Blinding of outcome assessment (detection bias) | High risk |
|  | Open Label Trial |
| Incomplete outcome data (attrition bias) | Low risk |
|  | Reasons for drop-out are clear and no imbalance |
| Selective reporting (reporting bias) | Low risk |
|  | Key outcomes (efficacy and safety) reported as per protocol |
| Other bias | Low risk |
|  | Baseline characteristics balanced |

| **Nasri 2022** | |
| --- | --- |
| **Bias** | **Authors’ judgement and support for judgement** |
| Random sequence generation (selection bias) | Unclear |
|  | Not clear how patients were randomly assigned |
| Allocation concealment (selection bias) | Unclear |
|  | Not reported |
| Blinding of participants and personnel (performance bias) | High risk |
|  | Open label |
| Blinding of outcome assessment (detection bias) | High risk |
|  | Open label |
| Incomplete outcome data (attrition bias) | Low risk |
|  | No drop outs reported. |
| Selective reporting (reporting bias) | High risk |
|  | Protocol available. However, safety data not reported, was described in protocol as a secondary outcome + in methods section. |
| Other bias | Low risk |
|  | Baseline demographics balanced |

| **Nimrouzi 2015** | |
| --- | --- |
| **Bias** | **Authors’ judgement and support for judgement** |
| Random sequence generation (selection bias) | Low risk |
|  | Block randomization |
| Allocation concealment (selection bias) | Unclear |
|  | Not reported |
| Blinding of participants and personnel (performance bias) | High risk |
|  | Open label |
| Blinding of outcome assessment (detection bias) | High risk |
|  | Open label |
| Incomplete outcome data (attrition bias) | Low risk |
|  | Reasons per group reported. No imbalance per group. |
| Selective reporting (reporting bias) | Unclear |
|  | No protocol found |
| Other bias | Unclear |
|  | Baseline characteristics not reported for all included patients, only the ones that reached study end. |

| **Olgac 2013** | |
| --- | --- |
| **Bias** | **Authors’ judgement and support for judgement** |
| Random sequence generation (selection bias) | Unclear |
|  | Says random, but not how random occurred |
| Allocation concealment (selection bias) | Unclear |
|  | Not mentioned |
| Blinding of participants and personnel (performance bias) | High risk |
|  | Open Label Trial |
| Blinding of outcome assessment (detection bias) | High risk |
|  | Open Label Trial |
| Incomplete outcome data (attrition bias) | Low risk |
|  | Reasons for drop-out are clear and no imbalance |
| Selective reporting (reporting bias) | Unclear |
|  | No protocol found or trial registration details |
| Other bias | Low risk |
|  | Baseline characteristics balanced |

| **Qiao 2021** | |
| --- | --- |
| **Bias** | **Authors’ judgement and support for judgement** |
| Random sequence generation (selection bias) | Low risk |
|  | According to the sequence generated by Random Allocation Software (version 1.0.0), the grouping was randomized in a ratio of 1:1, which was performed by a nonrecruited researcher |
| Allocation concealment (selection bias) | Unclear |
|  | Not reported |
| Blinding of participants and personnel (performance bias) | Low risk |
|  | The placebo group received a placebo designed to match the CHM group based on appearance, weight, colour, taste, and odour, including 5% drug ingredients and 95% dextrin. Randomisation was performed by a nonrecruited researcher. Patients, researchers, evaluators, and sponsors did not know which patients received which treatments. |
| Blinding of outcome assessment (detection bias) | Low risk |
|  | The placebo group received a placebo designed to match the CHM group based on appearance, weight, colour, taste, and odour, including 5% drug ingredients and 95% dextrin. Randomisation was performed by a nonrecruited researcher. Patients, researchers, evaluators, and sponsors did not know which patients received which treatments. |
| Incomplete outcome data (attrition bias) | Low risk |
|  | No imbalance. Reasons provided for dropouts |
| Selective reporting (reporting bias) | Unclear |
|  | Unclear defined outcomes: full remission and improvement were predefined, however in the results they use recovery rate. Unclear what recovery rate refers to. Protocol found, seems to meet protocol. |
| Other bias | Low risk |
|  | Baseline demographics balanced |

| **Quitadamo 2012** | |
| --- | --- |
| **Bias** | **Authors’ judgement and support for judgement** |
| Random sequence generation (selection bias) | Low risk |
|  | Randomly assigned according to an automatically generated randomization list |
| Allocation concealment (selection bias) | High risk |
|  | Not mentioned in the paper.  Answer author: The pharmacy was not involved. The randomization was performed in person by the study personnel. Patients were allocated sequentially, according to the randomization list. The study personnel had access to the randomization list. |
| Blinding of participants and personnel (performance bias) | High risk |
|  | Open label |
| Blinding of outcome assessment (detection bias) | High risk |
|  | Open label |
| Incomplete outcome data (attrition bias) | Low risk |
|  | Clear reasons for drop-out per patient. Difference between both groups > 20%, but this is a result of the difference in drug (bad taste). |
| Selective reporting (reporting bias) | Unclear |
|  | No protocol found |
| Other bias | Low risk |
|  | Baseline demographics balanced |

| **Reeves 2022** | |
| --- | --- |
| **Bias** | **Authors’ judgement and support for judgement** |
| Random sequence generation (selection bias) | Low risk |
|  | Block randomisation protocol. |
| Allocation concealment (selection bias) | High risk |
|  | Not concealed random list. Clinicians would conduct their visit with the patient, diagnose functional constipation, and offer enrolment on the study. They would review the block randomization figure and see which treatment was next (IG or CG). |
| Blinding of participants and personnel (performance bias) | High risk |
|  | Could not be blinded. |
| Blinding of outcome assessment (detection bias) | High risk |
|  | Could not be blinded. |
| Incomplete outcome data (attrition bias) | Unclear |
|  | Unclear how many per group. No randomized numbers per group. No reasons provided. |
| Selective reporting (reporting bias) | Low risk |
|  | Key outcome measurements reported as per protocol. Rest of the study also as per protocol. |
| Other bias | Unclear |
|  | Baseline demographics not provided of all patients who were randomized. |

| **Russo 2017** | |
| --- | --- |
| **Bias** | **Authors’ judgement and support for judgement** |
| Random sequence generation (selection bias) | Low risk |
|  | Automatically generated randomization |
| Allocation concealment (selection bias) | High risk |
|  | Not mentioned in the paper.  Answer author: The pharmacy was not involved. The randomization was performed in person by the study personnel. Patients were allocated sequentially, according to the randomization list. The study personnel had access to the randomization list. |
| Blinding of participants and personnel (performance bias) | High risk |
|  | Open Label Trial |
| Blinding of outcome assessment (detection bias) | High risk |
|  | Open Label Trial |
| Incomplete outcome data (attrition bias) | Low risk |
|  | Reasons for drop-out are clear and no imbalance |
| Selective reporting (reporting bias) | Unclear |
|  | No protocol found or trial registration details |
| Other bias | Low risk |
|  | Baseline demographics balanced. |

| **Sadeghzadeh 2014** | |
| --- | --- |
| **Bias** | **Authors’ judgement and support for judgement** |
| Random sequence generation (selection bias) | Unclear |
|  | Says random, but not how random occurred |
| Allocation concealment (selection bias) | Unclear |
|  | Not mentioned |
| Blinding of participants and personnel (performance bias) | Unclear |
|  | Says double blinded, but unclear who exactly were blinded. Both treatments had similar sachets, but nothing mentioned if taste, colour etc or similar. |
| Blinding of outcome assessment (detection bias) | Unclear |
|  | Says double blinded, but unclear who exactly were blinded. Both treatments had similar sachets, but nothing mentioned if taste, colour etc or similar. |
| Incomplete outcome data (attrition bias) | Low risk |
|  | Reasons for drop-out are clear and no imbalance |
| Selective reporting (reporting bias) | Unclear |
|  | No protocol found or trial registration details |
| Other bias | Unclear |
|  | Baseline characteristics not reported for all included patients, only the ones that reached study end. Only age and gender reported. |

| **Saneian 2021** | |
| --- | --- |
| **Bias** | **Authors’ judgement and support for judgement** |
| Random sequence generation (selection bias) | Low risk |
|  | Block randomization was done with a computer‑generated random number list prepared by an investigator with no clinical involvement in the trial. |
| Allocation concealment (selection bias) | Unclear |
|  | Not reported |
| Blinding of participants and personnel (performance bias) | Unclear |
|  | Unclear how clinicians who enrolled the patients or assessing the outcomes, and the parents were blinded |
| Blinding of outcome assessment (detection bias) | Unclear |
|  | Unclear how clinicians who enrolled the patients or assessing the outcomes, and the parents were blinded |
| Incomplete outcome data (attrition bias) | Low risk |
|  | Reasons are reported and number of dropouts is balanced |
| Selective reporting (reporting bias) | High risk |
|  | No protocol found, registration number leads to another study. In methods: "all of suspected adverse events were recorded", but only diarrhoea is reported as side effect in results. |
| Other bias | Unclear |
|  | Baseline characteristics only reported for patients that made study end. |

| **Savino 2005** | |
| --- | --- |
| **Bias** | **Authors’ judgement and support for judgement** |
| Random sequence generation (selection bias) | Unclear |
|  | Says random, but not how randomisation occurred |
| Allocation concealment (selection bias) | Low risk |
|  | Randomised to IG/CG via sealed envelopes |
| Blinding of participants and personnel (performance bias) | High risk |
|  | Not mentioned - assumed to be open label. No conceivable way to blind. |
| Blinding of outcome assessment (detection bias) | High risk |
|  | Not mentioned - assumed to be open label. No conceivable way to blind. |
| Incomplete outcome data (attrition bias) | Unclear |
|  | Drop-outs reported without reasons given, no imbalance. |
| Selective reporting (reporting bias) | Unclear |
|  | No protocol found or trial registration details |
| Other bias | Low risk |
|  | Baseline demographics balanced. |

| **Sevilla 2022** | |
| --- | --- |
| **Bias** | **Authors’ judgement and support for judgement** |
| Random sequence generation (selection bias) | Low risk |
|  | Computer generated randomization sequence in blocks of 10 |
| Allocation concealment (selection bias) | Unclear |
|  | Not reported |
| Blinding of participants and personnel (performance bias) | High risk |
|  | Open label |
| Blinding of outcome assessment (detection bias) | High risk |
|  | Open label |
| Incomplete outcome data (attrition bias) | Low risk |
|  | Two dropouts in IG, reasons not reported. Not expected to have an impact on results. |
| Selective reporting (reporting bias) | Low risk |
|  | Critical outcomes reported. Protocol found, outcomes reported as per protocol. |
| Other bias | Low risk |
|  | Baseline demographics balanced |

| **Shahamat 2016** | |
| --- | --- |
| **Bias** | **Authors’ judgement and support for judgement** |
| Random sequence generation (selection bias) | Low risk |
|  | Block randomisation |
| Allocation concealment (selection bias) | Unclear |
|  | Not mentioned |
| Blinding of participants and personnel (performance bias) | High risk |
|  | Reported that only methodologist and statistician who assessed and analysed were blinded |
| Blinding of outcome assessment (detection bias) | High risk |
|  | Reported that only methodologist and statistician who assessed and analysed were blinded |
| Incomplete outcome data (attrition bias) | Low risk |
|  | Reasons for drop-out are clear and no imbalance |
| Selective reporting (reporting bias) | Unclear |
|  | No protocol found or trial registration details |
| Other bias | Low risk |
|  | Baseline demographics balanced |

| **Sharifi-Rad 2018** | |
| --- | --- |
| **Bias** | **Authors’ judgement and support for judgement** |
| Random sequence generation (selection bias) | Low risk |
|  | Block randomisation |
| Allocation concealment (selection bias) | Low risk |
|  | Computer-generated list of random numbers was used to allocate participants |
| Blinding of participants and personnel (performance bias) | High risk |
|  | Patients, parents and physicians blind as well as outcome assessors. Physiotherapists were not. |
| Blinding of outcome assessment (detection bias) | Low risk |
|  | As Above |
| Incomplete outcome data (attrition bias) | Low risk |
|  | All drop-outs reported per group as represented in flowchart with reasons. |
| Selective reporting (reporting bias) | Low risk |
|  | Protocol available. Key safety outcomes reported. |
| Other bias | Low risk |
|  | Baseline demographics balanced. |

| **Silva 2013** | |
| --- | --- |
| **Bias** | **Authors’ judgement and support for judgement** |
| Random sequence generation (selection bias) | Unclear |
|  | Table of randomised numbers created by individual external to study to determine random distribution sequence of patients |
| Allocation concealment (selection bias) | Unclear |
|  | The information remained the exclusive knowledge of one research assistant, who used these numbers to allocate patients by order of study entry immediately after receiving informed consent, and was made known to the researchers only after the statistical analysis. Need to know if research assistant is involved or not. |
| Blinding of participants and personnel (performance bias) | High risk |
|  | Open label |
| Blinding of outcome assessment (detection bias) | High risk |
|  | Says analysis of the two groups at the end of the follow-up period for primary and secondary outcome measures was blind, but not reported how. |
| Incomplete outcome data (attrition bias) | Low risk |
|  | Reasons for drop-out are clear and no imbalance |
| Selective reporting (reporting bias) | Unclear |
|  | No protocol found or trial registration details |
| Other bias | Low risk |
|  | Baseline demographics balanced. |

| **Souza 2018** | |
| --- | --- |
| **Bias** | **Authors’ judgement and support for judgement** |
| Random sequence generation (selection bias) | Low risk |
|  | Computer generated random number table |
| Allocation concealment (selection bias) | Unclear |
|  | Says assigned to blocks. Unclear how. |
| Blinding of participants and personnel (performance bias) | Low risk |
|  | Double blind. Identical packaging and coding standardised. Reported |
| Blinding of outcome assessment (detection bias) | Low risk |
|  | Double blind. Identical packaging and coding standardised. Reported |
| Incomplete outcome data (attrition bias) | Low risk |
|  | All drop-outs reported per group as represented in flowchart with reasons. |
| Selective reporting (reporting bias) | Low risk |
|  | Protocol available. Key safety outcomes reported. |
| Other bias | Low risk |
|  | Baseline demographics balanced. |

| **Sunic 2002** | |
| --- | --- |
| **Bias** | **Authors’ judgement and support for judgement** |
| Random sequence generation (selection bias) | Unclear |
|  | Says random but no rationale or method given |
| Allocation concealment (selection bias) | Unclear |
|  | Says allocated but no rationale or method given |
| Blinding of participants and personnel (performance bias) | High risk |
|  | Open label |
| Blinding of outcome assessment (detection bias) | High risk |
|  | Open label |
| Incomplete outcome data (attrition bias) | Unclear |
|  | Not reported |
| Selective reporting (reporting bias) | High risk |
|  | No protocol found. No safety outcomes reported. |
| Other bias | Low risk |
|  | Baseline demographics balanced. |

| **Tabbers 2011** | |
| --- | --- |
| **Bias** | **Authors’ judgement and support for judgement** |
| Random sequence generation (selection bias) | Low risk |
|  | Block randomisation performed by Danone Research prior to the study onset |
| Allocation concealment (selection bias) | Low risk |
|  | Central allocation. |
| Blinding of participants and personnel (performance bias) | Low risk |
|  | The two treatments were identical in weight, colour, smell, taste and package. All doctors, research staff and patients involved are unaware of the treatment administered to the patient. |
| Blinding of outcome assessment (detection bias) | Low risk |
|  | The two treatments were identical in weight, colour, smell, taste and package. All doctors, research staff and patients involved are unaware of the treatment administered to the patient. |
| Incomplete outcome data (attrition bias) | Low risk |
|  | 5/79 in IG dropped out with reason 'lost to follow up' and 6/80 in CG. For other drop outs (6 in IG and 4 in CG) reasons were provided. |
| Selective reporting (reporting bias) | Low risk |
|  | Protocol published as separate articles. Key outcomes reported as per protocol (safety and efficacy) |
| Other bias | Low risk |
|  | Baseline characteristics balanced |

| **Tavassoli 2021** | |
| --- | --- |
| **Bias** | **Authors’ judgement and support for judgement** |
| Random sequence generation (selection bias) | Low risk |
|  | Randomly allocated by the permuted randomisation method (with block sizes of four) |
| Allocation concealment (selection bias) | Low risk |
|  | A random number list generated by using a computer was used to assign participants to two arms. The researcher conducting randomization was not involved in other parts of the study. |
| Blinding of participants and personnel (performance bias) | High risk |
|  | Clinician was not blinded. Only containers were similar, nothing mentioned about taste and appearance. |
| Blinding of outcome assessment (detection bias) | High risk |
|  | Clinician was not blinded. Only containers were similar, nothing mentioned about taste and appearance. |
| Incomplete outcome data (attrition bias) | Low risk |
|  | Reasons for dropout provided. Number of dropouts is balanced |
| Selective reporting (reporting bias) | Unclear |
|  | The only efficacy outcome reported in the protocol was bowel frequency. In the study there were a lot more outcomes, unknown if these outcomes were predefined. |
| Other bias | Low risk |
|  | Baseline characteristics balanced. |

| **Tjokronegoro 2020** | |
| --- | --- |
| **Bias** | **Authors’ judgement and support for judgement** |
| Random sequence generation (selection bias) | Low risk |
|  | The randomization list was established with a permutation block of constant length (6 subjects per block). |
| Allocation concealment (selection bias) | Low risk |
|  | The list was placed in a concealed envelope and was stored until the end of study. |
| Blinding of participants and personnel (performance bias) | Low risk |
|  | The probiotics and placebo were manufactured by Novell Pharmaceutical Laboratories as identical powder with similar appearance and taste, which were packed in identical aluminium sachets. Throughout the study, investigator, participants, and data analyst were blinded to the assigned treatment. |
| Blinding of outcome assessment (detection bias) | Low risk |
|  | The probiotics and placebo were manufactured by Novell Pharmaceutical Laboratories as identical powder with similar appearance and taste, which were packed in identical aluminium sachets. Throughout the study, investigator, participants, and data analyst were blinded to the assigned treatment. |
| Incomplete outcome data (attrition bias) | Low risk |
|  | In balance. Almost all reasons provided. Only of 1 patient no reason provided for drop out. |
| Selective reporting (reporting bias) | Unclear |
|  | Protocol not found |
| Other bias | Low risk |
|  | Baseline demographics balanced |

| **Ustundag 2010** | |
| --- | --- |
| **Bias** | **Authors’ judgement and support for judgement** |
| Random sequence generation (selection bias) | Unclear |
|  | Randomization was performed by the use of sequential numbers allocated to the patients at the study entry. |
| Allocation concealment (selection bias) | Unclear |
|  | Not reported |
| Blinding of participants and personnel (performance bias) | High risk |
|  | Open label |
| Blinding of outcome assessment (detection bias) | High risk |
|  | Open label |
| Incomplete outcome data (attrition bias) | High risk |
|  | No reasons for drop-out reported |
| Selective reporting (reporting bias) | High risk |
|  | No protocol found. However, treatment success was given as outcome in methods, however no data reported in results. |
| Other bias | Unclear |
|  | Baseline characteristics only reported for patients that made study end. |

| **Van der Plas 1996** | |
| --- | --- |
| **Bias** | **Authors’ judgement and support for judgement** |
| Random sequence generation (selection bias) | Unclear |
|  | Says random but no rationale or method given |
| Allocation concealment (selection bias) | Unclear |
|  | Not mentioned |
| Blinding of participants and personnel (performance bias) | High risk |
|  | Open label. But could not be blinded. |
| Blinding of outcome assessment (detection bias) | High risk |
|  | Open label. But could not be blinded. |
| Incomplete outcome data (attrition bias) | Low risk |
|  | Reasons for drop-out are clear and no imbalance |
| Selective reporting (reporting bias) | Unclear |
|  | No protocol found or trial registration details |
| Other bias | Low risk |
|  | Baseline characteristics balanced |

| **Van Dijk 2008** | |
| --- | --- |
| **Bias** | **Authors’ judgement and support for judgement** |
| Random sequence generation (selection bias) | Low risk |
|  | A computer-based system was used to generate a sequence of random group assignment for consecutive patients. |
| Allocation concealment (selection bias) | Low risk |
|  | A research assistant performed a telephone call to a randomization centre and revealed the allocation to parents immediately. |
| Blinding of participants and personnel (performance bias) | High risk |
|  | Open label. But could not be blinded. |
| Blinding of outcome assessment (detection bias) | High risk |
|  | Open label. But could not be blinded. |
| Incomplete outcome data (attrition bias) | Low risk |
|  | All drop-outs reported per group as represented in flowchart with reasons. |
| Selective reporting (reporting bias) | Low risk |
|  | Protocol. Key safety outcomes reported. |
| Other bias | Low risk |
|  | Baseline characteristics balanced |

| **Weber 2014** | |
| --- | --- |
| **Bias** | **Authors’ judgement and support for judgement** |
| Random sequence generation (selection bias) | Low risk |
|  | Random number table |
| Allocation concealment (selection bias) | Unclear |
|  | Says assigned to 1:1 blocks, unclear how allocation happened. |
| Blinding of participants and personnel (performance bias) | Low risk |
|  | Double blinded. Patients and parents blinded. Both IG and CG labelling was standardised, products resembles each other and administered in an identical manner. |
| Blinding of outcome assessment (detection bias) | Low risk |
|  | Double blinded. Patients and parents blinded. Both IG and CG labelling was standardised, products resembles each other and administered in an identical manner. |
| Incomplete outcome data (attrition bias) | Low risk |
|  | All drop-outs reported per group as represented in flow chart with reasons. |
| Selective reporting (reporting bias) | Unclear |
|  | No protocol found or trial registration details |
| Other bias | Low risk |
|  | Baseline characteristics balanced |

| **Wegner 2018** | |
| --- | --- |
| **Bias** | **Authors’ judgement and support for judgement** |
| Random sequence generation (selection bias) | Low risk |
|  | Randomly assigned according to automatically generated randomisation list |
| Allocation concealment (selection bias) | Unclear |
|  | Not mentioned |
| Blinding of participants and personnel (performance bias) | Unclear |
|  | States double blind but only States "matching placebo and macrogol". More information needed if and how personnel was blinded. |
| Blinding of outcome assessment (detection bias) | Unclear |
|  | States double blind but only States "matching placebo and macrogol". More information needed if and how personnel was blinded. |
| Incomplete outcome data (attrition bias) | Low risk |
|  | All drop-outs reported per group as represented in flow chart with reasons. |
| Selective reporting (reporting bias) | Low risk |
|  | Protocol and NCT registration present in article. Safety and primary outcome reported per protocol |
| Other bias | Low risk |
|  | Baseline characteristics balanced |

| **Wojtyniak 2017** | |
| --- | --- |
| **Bias** | **Authors’ judgement and support for judgement** |
| Random sequence generation (selection bias) | Low risk |
|  | The randomization list was generated by an investigator with no clinical involvement in the trial, via a computer program (StatsDirect) with an allocation ratio of 1:1 and with a block of 6 |
| Allocation concealment (selection bias) | Low risk |
|  | The allocation sequence was concealed from the researchers responsible for enrolling and assessing participants in sequentially numbered, white, opaque, sealed, and stapled envelope. |
| Blinding of participants and personnel (performance bias) | Low risk |
|  | Identical capsules with an identical taste, smell, and appearance. |
| Blinding of outcome assessment (detection bias) | Low risk |
|  | Identical capsules with an identical taste, smell, and appearance. |
| Incomplete outcome data (attrition bias) | Low risk |
|  | All drop-outs reported per group as represented in flow chart with reasons - provided in appendix at article end |
| Selective reporting (reporting bias) | Low risk |
|  | Protocol available. Key safety outcomes reported. |
| Other bias | Low risk |
|  | Baseline characteristics balanced |

| **Xu 2015** | |
| --- | --- |
| **Bias** | **Authors’ judgement and support for judgement** |
| Random sequence generation (selection bias) | Unclear |
|  | Unclear how children were randomised exactly |
| Allocation concealment (selection bias) | Unclear |
|  | Not reported |
| Blinding of participants and personnel (performance bias) | High risk |
|  | Open-label. Unable to do blinded trial. |
| Blinding of outcome assessment (detection bias) | High risk |
|  | Open-label. Unable to do blinded trial. |
| Incomplete outcome data (attrition bias) | Unclear |
|  | Not reported. |
| Selective reporting (reporting bias) | Unclear |
|  | No protocol found or trial registration details |
| Other bias | Unclear |
|  | Not reported |

| **Young 1998** | |
| --- | --- |
| **Bias** | **Authors’ judgement and support for judgement** |
| Random sequence generation (selection bias) | Unclear |
|  | Not mentioned |
| Allocation concealment (selection bias) | Unclear |
|  | Not mentioned |
| Blinding of participants and personnel (performance bias) | High risk |
|  | Open label |
| Blinding of outcome assessment (detection bias) | High risk |
|  | Open label |
| Incomplete outcome data (attrition bias) | Unclear |
|  | Not reported |
| Selective reporting (reporting bias) | Unclear |
|  | No protocol found or trial registration details |
| Other bias | Unclear |
|  | Not reported per group |

| **Yu 2023** | |
| --- | --- |
| **Bias** | **Authors’ judgement and support for judgement** |
| Random sequence generation (selection bias) | Low risk |
|  | Sequence generated by Random Allocation Software (version 1.0.0), the grouping was randomized in a 1:1 ratio |
| Allocation concealment (selection bias) | Low risk |
|  | Asked author: Central allocation |
| Blinding of participants and personnel (performance bias) | High risk |
|  | Patients with different treatments were assigned to different rooms or different periods to prevent communication between patients. The treatment process was completed by nurses and they did not participate in the collection and evaluation of results. Evaluators did not participate in the treatment process. Control group received a sham PTNS. But treating nurse cannot have ben blinded for intervention. Also unclear what how sham was exactly performed. |
| Blinding of outcome assessment (detection bias) | Low risk |
|  | Patients with different treatments were assigned to different rooms or different periods to prevent communication between patients. The treatment process was completed by nurses and they did not participate in the collection and evaluation of results. Evaluators did not participate in the treatment process. Control group received a sham PTNS. |
| Incomplete outcome data (attrition bias) | Low risk |
|  | In balance. Only of two drop outs no reason provided (out of total of 9) |
| Selective reporting (reporting bias) | Low risk |
|  | Key outcomes reported as per protocol. Safety not reported in protocol, but reported in paper (methods and results) |
| Other bias | Low risk |
|  | Baseline demographics balanced |

| **Zaja 2021** | |
| --- | --- |
| **Bias** | **Authors’ judgement and support for judgement** |
| Random sequence generation (selection bias) | Low risk |
|  | Automatically generated randomisation list |
| Allocation concealment (selection bias) | Unclear |
|  | Not reported |
| Blinding of participants and personnel (performance bias) | Low risk |
|  | Group-2 received placebo, consisting of an identical formulation in all aspects, except excluded the live bacteria. The study personnel, healthcare providers, patients and parents were blinded to the study group allocation. |
| Blinding of outcome assessment (detection bias) | Low risk |
|  | Group-2 received placebo, consisting of an identical formulation in all aspects, except excluded the live bacteria. The study personnel, healthcare providers, patients and parents were blinded to the study group allocation. |
| Incomplete outcome data (attrition bias) | Low risk |
|  | No dropouts. |
| Selective reporting (reporting bias) | Low risk |
|  | Protocol found. Outcomes reported as per protocol. |
| Other bias | Low risk |
|  | Baseline demographics balanced |

**Supplementary Table 3. Characteristics of included pharmacological studies**

*Disimpaction*

| **Study ID** | **Participants**  (age, number, definition FC) | **Interventions**  (dosage, treatment period, concomitant therapy) | **Treatment success** (definition + time of measurement) | **Withdrawals due to AE** | **Defecation frequency** (definition + time of measurement) |
| --- | --- | --- | --- | --- | --- |
| **Rectal enema vs PEG (oral)** | | | | | |
| Bekkali 2009 (1) | Age 4 – 16 years, N=90  FC definition: Rome III  FI definition: large amount of hard stool in the rectum (fecaloma) | Rectal enema: dioctylsulfosuccinate sodium, once daily for 6 days (60 ml children < 6 years, and 120 ml for children of 6 years and older)  PEG (oral): PEG3350 + electrolytes 1.5 gr/kg per day for 6 days.  Maintenance therapy was started in both groups after 6 days of disimpaction: PEG3350 + electrolytes 0.5 g/kg/day for at least 2 weeks (follow-up period) | Def: absence of fecaloma on digital rectal examination (DRE). If children scared to undergo second DRE, X-ray performed  Time: 6 days | Reported  Time: 3 weeks | Def: frequency per week, mean (SD)  Time: 3 weeks (2 weeks after week of disimpaction treatment) |

*Maintenance therapy*

| **Study** | **Participants**  (age, number, definition FC) | **Intervention**  (dosage, treatment duration, concomitant therapy) | **Treatment success**  (definition + time of measurement) | **Withdrawals due to AE** | **Defecation frequency** (definition + time of measurement) |
| --- | --- | --- | --- | --- | --- |
| **PEG vs Lactulose** | | | | | |
| Dheivamani 2021 (2) | Age 2 – 12 years, N=100, Rome IV | PEG 3350: 0.7 g/kg once/day  Lactulose 0.7 g/kg once/day  Treatment duration: 4 weeks | Def: Response rate: more than 2 bowel movements per week Time: 4 weeks | Reported | Def: frequency per week, mean (SD).  Time: 4 weeks |
| Dupont 2005 (3) | Age 6 months – 3 years, N=98, FC defined as ‘< 1 stool per day  for more than 1 month in children 6 - 12 months old and < 3 stools per week for more than 3 months in children aged  13 months - 3 years’ | PEG 4000: one sachet of 4 g/sachet Lactulose: one sachet of  3.33 g/sachet. Dose could be increased.  Treatment duration: 3 months | NR | Reported | Def: frequency per week, median (IQR).  Reported separately for ages 6 mo – 12 mo and 13 mo – 3 y  Time: 3 months |
| Jarzebicka 2019 (4) | Age 6 months – 6 years, N=102, Rome III | PEG: up to 8 kg, 5 g/day; 8  to 12 kg, 10 g/day; 12 to 20 kg, 15 g/day; >20 kg, 20 g/day,  divided as 2 doses.  Lactulose: 1 mL/kg, twice a day.  4 weeks | Def: 3 or more stools per week and an improvement in stool consistency of at least 2 types in the Bristol scale were considered good clinical outcome  Time: 4 weeks | Reported | Def: frequency per week, mean (SD)  Time: at week 4 |
| Saneian 2012 (5) | Age 1 – 16 years, N=90, Rome III | PEG:  1 cc /kg/day  Lactulose: 1 cc /kg/day  MgOH: 1 cc /kg/day  Dosage could be increased to 3 cc/kg/day)  Treatment duration: 4 weeks  Patients were disimpacted at start of treatment if necessary. | Def: defecation equal or more than 3 times a week without pain and bleeding,  in addition with fecal incontinence less than twice a month at the end of one month treatment. No data reported | NR | Def: increase in frequency per week (SD)  Time: 4 weeks |
| Treepongkaruna 2014 (6) | Age 12 months – 36 months, N=88, FC defined as ‘either a stool frequency of ≤2 per week persisting for at least 3 months OR the presence of pebble-like, hard stools, painful defecation or faecal incontinence for at least 3 months’ | PEG 4000: 8 g per day  Lactulose: 3,3 g per day  4 weeks | Not reported | Reported | Def: frequency per day, mean (SD)  Time: 4 weeks |
| Uhm 2007 (translated from Chinese) (7) | Age unclear. Only reported mean age at diagnosis: 5,5 years. N=56, FC defined according to IOWA-criteria | PEG 4000: 0,5 g/kg per day  Lactulose: 1,5ml/kg daily, divided in two doses per day.  Treatment duration: unclear. Patients could be weaned off medication during the 12-month study period. | Def: number of painless bowel movements without medication for at least one month. Number of painless bowel movements is at least 3 times per week, fecal incontinence is less than 2 times per month, and no abdominal pain.  Time: after 12 months | Reported | NR |
| Voskuijl 2004 (8) | Age 6 months – 15 years, N=100, FC defined as ‘at least 2 out of 4 of the following symptoms for the last 3 months: < 3 bowel movements per week; encopresis > 1/week; large amounts of stool every 7–30 days (large enough to clog the toilet); and palpable abdominal or rectal mass on physical examination. | PEG 3350: Between 6 months and 6 years of age: 2,95 g per day  >6 years: 5.6 g per day  Lactulose:  Between 6 months and 6 years of age: 6 g  >6 years: 12 g per day  Treatment duration: 8 weeks | Def: defecation frequency >3/week and encopresis (1 or less every two weeks).  Time: 8 weeks | Reported | Def: frequency per week, mean (SD)  Time: 8 weeks |
| Wang 2007 (translated from Chinese) (9) | Age 8 – 18 years, N=227, FC defined as ‘passing type 1-3 stool as per Bristol stool chart and having ≤ 2 bowel motions for 2 consecutive weeks. | PEG 4000: 2 packs (20g) taken once a day  Lactulose: 15ml once a day for three days, followed by maintenance dose of 10ml twice a day  Treatment duration: 2 weeks | Def: Stool properties returned to normal  Time: 2 weeks | NR | NR |
| **PEG vs placebo** | | | | | |
| Nurko 2008 (10) | Age 4 – 16 years old,  N=103, FC defined as ‘at least 3 months ≤ 3 spontaneous bowel movements (BM) per week and 1 or more associated symptoms that included straining, hard stools sensation of incomplete evacuation, production of large bowel movements that may obstruct the toilet, or painful defecation’ | PEG 3350: 0.2 g/kg or 0.4 g/kg or 0.8 g/kg per day  Placebo  Treatment duration: 2 weeks | Def: three or more bowel movements during the second week of treatment  Time: 2 weeks | Reported | Reported, but no exact data available for analysis. |
| Modin 2018 (11) | Age 2 – 16,  N= 115,  Rome III | PEG 3350: 0.8 g/kg per day  Placebo: identical to PEG  At least 8 weeks, after that patients could be weaned off medication | Def: the absence of any Rome III criteria, with or without use of medication  Time: 24 weeks | Reported | Def: frequency per week, mean (SD)  Time: 24 weeks |
| Thomson 2007 (12) | Age 2 – 11 years,  N=51,  Rome criteria | PEG 3350 + electrolytes: 6.9 g powder per sachet. Number of sachets depended on age and increased by a dosing regimen during the first week to 4 – 6 sachets per day.  Placebo: identical to PEG  Dosage could be adjusted in the second week to determine a dose at which symptoms of constipation as defined by the Rome criteria noted above did not occur.  Treatment duration: 2 weeks  Children were excluded from the study if they had current or previous fecal impaction. | NR | NR | Def: frequency per week, mean (SD)  Time: 2 weeks |
| **PEG vs Magnesium hydroxide (MgOH)** | | | | | |
| Gomes 2011 (13) | Age 1 – 15 years,  N=38,  Rome III | PEG 4000: 0.5 g/kg per day  MgOH: 3 mL/kg per day  Treatment duration: 6 months | NR | Reported | Def: frequency per week, mean (SD)  Time: 6 months |
| Loening-baucke 2006 (14) | Age 4 – 18 years,  N=79,  Rome III | PEG 3350: 0.7 g/kg per day  MgOH: 2 mL/kg  Treatment duration: unclear, patients could be off medication at end of study | NR | Reported | Def: frequency per week, mean (SD)  Time: 6 months |
| Ratanamongkol 2009 (15) | Age 1 – 4 years  N=94, | PEG 4000: 0.5 g/kg  MgOH: 3 mL/kg per day  Treatment duration: unclear, patients could be off medication at end of study | Def: the proportion of patients who had ≥ three bowel movements per week, ≤ two episodes of fecal incontinence per month, and no painful defecation, with or without laxative therapy  Time: 4 weeks | Reported | Def: frequency per week, median (IQR)  Time: 4 weeks |
| **PEG vs Liquid paraffin** | | | | | |
| Karami 2009 (16) | Age 1 – 10 years,  N=126,  FC defined as ‘stool  frequency less than 2 times per week with  fecal hard consistency, encopresis two or  more than two times per month, palpable fecal  impaction in abdomen or rectum’ | PEG: 0.8 g/kg twice per day  Liquid paraffin: 1 cc/kg twice per day  Treatment duration: unclear | NR | NR | Def: frequency per week, mean (SD)  Time: 4 weeks |
| Rafati 2011 (17) | Age 2 to 12 years,  N=160,  FC defined as ‘less than 3 stools per week, more than 1 encopresis per week or palpable abdominal or rectal fecal mass on physical examination’ | PEG 3350 1.0-1.5 g/kg per day  Liquid paraffin: 1.0-1.5 ml/kg per day  Treatment duration: 4 months | Unclear definition | Reported | Def: frequency per week, mean (SD)  Time: 4 months |
| **PEG vs Herbal medicine** | | | | | |
| Dehghani 2019 (18) | Age 4 – 12 years, N=92, Rome III | PEG: syrup (40% w/v) with a dose of 1 mL/kg body weight/day  Black Strap Molasses: syrup (40%w/v) with a dose of 1 mL/kg body weight/day  Treatment duration: 4 weeks  Concomitant therapy: toilet training and nutritional advice in both groups. | Def: not fulfilling the Rome III criteria  Time: 4 weeks | Reported | Def: number of patients with ≤2 bowel movements/week  Time: 4 weeks |
| Esmaeilidooki 2016 (19) | Age 2 – 15 years,  N=109,  Rome III | PEG 4000: 0.7 – 0.8 g/kg per day  Cassia Fistula‘s Emulsion: 1 cc/kg per day  Treatment duration: 4 weeks | Def: No longer fulfilling Rome III criteria  Time: 4 weeks | Reported | Def: frequency per week, mean (SD)  Time: 4 weeks |
| Nasri 2022 (20) | Age 2 – 15 years,  N=120 Rome IV | PEG 4000: 0.7 g/kg (unclear if its 0.7 g/kg three times per day, or if 0.7 g/kg is divided in three times during the day)  LaxaPlus Barij®: 1 mL/kg daily divided into three doses for <30 kg, 10 mL three times daily for >30 kg  Treatment duration: 8 weeks | NR | Reported | Def: frequency, unclear if per day or per week, mean (SD)  Time: 8 weeks |
| Imanieh 2022 (21) | Age 1 – 18 years, N=100, Rome IV | PEG: initial dosage was 1 ml/kg per day. R. damascene and brown sugar syrup: 0.1g damask rose petals and  0.85g brown sugar per 1 mL solution. Initial dosage was 1 ml/kg per day.  Both groups: If no response, the dosage was increased to 2 ml/kg.  Treatment duration: 4 weeks | Def: having fewer than two of the Rome IV criteria after treatment  Time: 4 weeks | Reported | Def: number of patients with 2 or fewer defecations per week  Time: 4 weeks |
| Nimrouzi 2015 (22) | Age 2 – 12,  N=120,  Rome III | PEG 4000: 0.4 g/kg per day  D. Sophia seed: 2 g for 2-4 years old,  3 g for 4-12 years old  Treatment duration: 8 weeks | Def: Improvement of constipation for at least 3 bowel movements, soft stool and  convenient defecation, no soiling and bloody stool per week as well as exiting the Rome III criteria for constipation after the third week.  Time: 8 weeks | Reported | Def: frequency per week, median (IQR)  Time: 3 weeks |
| Saneian 2021 (23) | Age 2 – 15 years,  N=60, Rome IV | PEG 4000: 0.7 g/kg per day  Golghand®: 0.5 g/kg per day  Treatment duration: 8 weeks | NR | Reported | Def: frequency per week, mean (SD)  Time: 8 weeks |
| Tavassoli 2021 (24) | Age 4 – 10 years,  N=140, Rome III | PEG: 1 g/kg per day  Viola Flower Syrup: 5 cc 3 times per day  Treatment duration: 4 weeks | NR | Reported | Def: frequency per week, mean (SD)  Time: 4 weeks |
| **PEG vs sodium picosulfate vs fiber** | | | | | |
| Cassettari 2019 (25) | Age 5 – 10, N=80, Rome IV | Group 1: PEG 3350 + electrolytes, dosage NR  Group 2: Sodium picosulfate, dosage NR  Group 3: Green banana biomass (GBB), 30 g per day  Group 4: PEG + GBB, dosage NR  Group 5: Sodium picosulfate + GBB, dosage NR  Treatment duration: 8 weeks  Concomitant therapy: dietary advice | NR | NR | Reported as dichotomous outcome.  Def: number of patients having more than 3 bowel motions as week.  Time: after 8 weeks |
| **PEG vs Fiber** | | | | | |
| Quitadamo 2012 (26) | Age 4 – 10 years, N=100, Rome III | PEG 3350 + electrolytes: 0,5 g/kg per day, increase up to 1.0 g/kg per day if necessary  Fiber mixture: 16,8 g per day, increase up to 22,4 g per day if necessary  Treatment duration: 8 weeks  Concomitant therapy: disimpaction before start treatment. Rescue therapy: enemas if no defecation for > 3 days | Def: 3 or more bowel movements per week, 2 or higher stool consistency grade on BSFS, absence of fecal incontinence, abdominal pain, pain on defecation, and fecal bleeding.  Time: 8 weeks | Reported | Def: frequency per week, mean (SD)  Time: 8 weeks |
| **PEG vs microenema** | | | | | |
| Strisciuglio 2021 (27)  *Promelaxin microenema* | Age 6 months – 4 years, N=158, Rome III | PEG: 4 g/day 6-12 months and 4-8 g/day for 12-48 months age Promelaxin: 2.5 mg (2ml) for children 6-12 months, 5mg (4ml) for 12-48 months with a maximum of 10 g (8ml) was given daily  Treatment duration: for 2 weeks. After these two weeks participants received the same daily dosage on-demand for 6 more weeks. On demand treatment was defined as the need for PEG or Promelaxin after 48 h without a fecal evacuation.  Concomitant therapy: dietary advice and toilet training recommendation. | Def: at least 3 evacuations per week and an average increase of at least one evacuation per week as compared to baseline after two weeks of treatment.  Time: 2 weeks | Reported | No data reported. |
| **PEG (with addition of probiotics) vs fiber vs synbiotic** | | | | | |
| Foroughi 2022 (28) | Age 2 – 12 years, N=144, Rome IV | PEG: 6 g per day PEG + probiotics: 6 g per day + 109 CFU bacterial probiotics (mixture of different stems)  fiber: Psyllium Seed Husk Powder 6 g per day  synbiotic (two component): Psyllium Seed Husk Powder 6 g per day day + 109 CFU bacterial probiotics (mixture of different stems)  Treatment duration: 3 weeks  Concomitant therapy: dietary advice and toilet training were provided to all parents. | NR | NR | Def: frequency per week, mean (SD)  Time: 3 weeks |
| **PEG4000 vs PEG3350 + electrolytes** | | | | | |
| Bekkali 2018 (29) | Age 6 months - 16 years, N=97, FC defined as ‘defecation frequency <3 times per week’ | PEG4000: sachets containing 4g of PEG with a molecular weight of 4000g/mol PEG3350+Electrolytes: sachets containing 2.95 g of PEG with a molecular weight of 3350 g/mol and electrolytes: 37.5 mg potassium chloride, 73 mg sodium chloride, 284 mg sodium sulfate, and 84 mg sodium hydrogen carbonate.  Treatment duration: 52 weeks  Concomitant therapy: rectal enemas first 3 days of treatment. Rescue medication (enema or oral dose of 5mg bisacodyl) if defecation did not occur within 3 consecutive days. | Def: defecation frequency of ≥3 per week with <1 episode of fecal incontinence.  Time: 52 weeks | Reported | Def: frequency per week, mean (SD)  Time: 52 weeks |
| Savino 2012 (30) | Age 2 – 16 years, N=96, Rome III | PEG4000: 0.7 g/kg/day; in children >20 kg same daily dose with a maximum limit of  30 g daily. PEG3350+Electrolytes: 6.9 g per sachet. 1 sachet per day in children aged 2–6 years; 2 sachets in children aged 7–11 years; 4 sachets in children aged 12–16 years.  Treatment duration: 4 weeks  Concomitant therapy: disimpaction treatment was initiated if fecal impaction was established. | Def: resolution of faecal impactment and Adequate relief of constipation in terms of normalized frequency (≥3 BM per week).  Time: 4 weeks  No data reported, only in figure. | Reported | Def: frequency per week, mean (SD)  Time: over the 4 weeks of treatment |
| **PEG 0.7 g/kg/day vs PEG 0.3 g/kg/day** | | | | | |
| Dziechciarz 2015 (31) | Age 1 – 18 years, N=92 , Rome III | PEG normal dose: 0.7 g/kg/day PEG low dose: 0.3 g/kg/day  Treatment duration: 6 weeks  Both groups: dose of PEG was adjusted and/or an additional laxative was administered if <3 BM per week or >3 loose stools per day were observed  Concomitant therapy: in case of fecal  impaction an enema (once per day, for a maximum of 3 days) was recommended- prior to randomisation. And children were instructed to sit on the toilet for 5 minutes after each meal. | Def: 3 or more bowel movements per week with no fecal soiling during  the last week of the intervention.  Time: 6 weeks | Reported | Def: frequency per week, mean (SD)  Time : 6 weeks |
| **Lactulose vs placebo** | | | | | |
| Cao 2018 (32) | Age 2 – 6 years, N=100, Rome III | Lactulose: 5 ml (3,3 g) per day  Placebo: same size, dose, color, flavor, and appearance  Treatment duration: 6 weeks | NR | NR | Def: frequency per day change from baseline, mean. Unclear if spread is reported as range or SD.  Time: 6 weeks |
| **Lactulose vs lactitol** | | | | | |
| Pitzalis 1995 (33) | Age 8 months – 16 years, N=51, chronic FC defined as ‘less than 3,5 weekly evacuations’ | Lactulose: 500 mg/kg/day as a single morning dose increased if necessary up to 750 mg/kg/day.  Lactitol: 50 mg/kg/day as a single dose in the morning, increased if necesary to 400 mg/kg/day.  Treatment duration: 4 weeks  Dietary advice and education for toilet training were given to parents. | NR | NR | Def: frequency per week, mean (SD)  Time: 4 weeks |
| **Lactulose vs liquid paraffin** | | | | | |
| Farahmand 2007 (34) | Age 2 – 12 years, N=247, FC defined as ‘having at least two out of  four of the following symptoms, for the last 3 months: < 3 bowel movements/week;  fecal soiling > 1 times per week, large amounts  of stool every 7-30 days and palpable abdominal or  rectal fecal mass on physical examination’ | Lactulose: 1-2  ml/kg twice per day Liquid paraffin: 1-2  ml/kg twice per day  Treatment duration: 8 weeks  At the first visit, patients received one or two enema daily for two days to clear any rectal fecal impaction. (30 cc / 10 kg weight of paraffin oil for enema.  Dose adjustment: increase or decrease of volume of each drug by 25% every 3 days as required, to yield, 1 or 2, firm–loose, stools. Instructions were given to increase daily fiber intake to an amount of grams equal to their age plus 10. | Def: defecation frequency 3 or more per week and encopresis 1 or less every two weeks.  Time: 8 weeks | Reported | Def: frequency per week, mean (SD)  Time: 8 weeks |
| Urganci 2005 (35) | Age 2 – 12 years, N=40, chronic constipation defined as symptoms of at least 3 months duration including at least two of the following: hard stools, painful defecation, rectal  bleeding, encopresis and fewer than three bowel movements  per week. | Lactulose: 1 mL/kg, twice per day.  Liquid paraffin: 1 mL/kg, twice per day.  Treatment duration: 8 weeks  Each drug was increased or decreased  by 25% every 3 days as required, to yield, 1 or 2, firm– loose, stools. Instructions were given  to increase daily fiber intake to an amount of grams equal to their age plus 10. | Def: clearance of the impaction (more than three bowel movements per week and improvement in stool consistency).  Time: last 4 weeks of treatment | Reported | Def: frequency per week, mean (SD)  Time: last 4 weeks of treatment |
| **Lactulose vs fibers** | | | | | |
| Kokke 2008 (36) | Age 1 – 13 years, N=135 , FC defined as 2 of 4 criteria: stool frequency less than 3 times per week,  fecal incontinence 2 or more times per week, periodic passage  of large amounts of stool at least once every 7 to 30 days, or a palpable abdominal or rectal mass | Lactulose: 10 g/125 mL  Fiber mixture: 10 g/125 mL  Patients with a weight <15 kg received 1 bottle (125 mL, 10 g fibers) daily, those with a weight between 15 kg and 20 kg received 2 bottles (250 mL, 20 g) daily, and those with a weight above 20 kg received 3 bottles (375 mL, 30 g) daily. The study product was taken at breakfast and, in the case of 2 or more bottles, also at lunch.  Treatment duration: 8 weeks + 4 weeks weaning period. Total of 12 weeks.  Enema was given in case of rectal impaction before start treatment.  Rescue medication: macrogol 3350 in no improvement after 3 weeks.  If persistent diarrhea was reported, the original dose was reduced by 50%. | NR | Reported | Def: frequency per week. Unclear if median/mean. No measure of spread reported. |
| Üstündağ 2010 (37) | Age 4 – 16 years, N=68, Rome III | Lactulose: 1 ml/kg/day, in divided doses Fibers: partially hydrolyzed guar gum (PHGG), for children between 4-6 years: 3 g/day; 6-12 years: 4 g/day; and 12-16 years: 5 g/day.  Treatment duration: 4 weeks  In case of rectal impaction, an enema was given at the first visit. If persistent diarrhea was reported, the original dose was reduced by 50%. two groups were given an equal diet with fiber. However, as dietary fiber can bind fluid, the group given PHGG was recommended to increase their fluid intake. | Def: soft to formed stool consistency, absence of pain, stool withholding and blood in the stool, and no palpable rectal or abdominal mass.  No data reported | Reported | Def: frequency per week, mean (SD)  Time: 4 weeks |
| **Lactulose as addition to PEG vs PEG alone** | | | | | |
| Ala 2015 (38) | Age 1 – 12 years, N=200 , Rome III | Lactulose + PEG: lactulose max dose 3 cc/kg/day, twice daily, PEG maximum dose 0.7 g/kg /day, 13.8 - 40 g/day, twice daily.  PEG: maximum dose 0.7 g/kg /day, 13.8 - 40 g/day, twice daily.  Treatment duration: 4 weeks  In case of fecal impaction: disimpaction with suppository bisacodyl and then laxative therapy.  Dietary advice given and toilet training discussed face to face and in pamphlets. | Def: ≥ 3 bowel movements per week, ≤ 2 episodes of fecal incontinence per month without abdominal pain  Time: 4 weeks | Reported | NR |
| **Lactulose vs probiotics** | | | | | |
| Lee 2022 (39) | Age 6 months – 10 years, N=187, Rome IV | Lactulose: 1.34 g/mL of lactulose. The starting dose was 1 mL/ kg/day, dosage change was allowed according to any clinical improvement noted during the follow-up period. Probiotic: Bioflor 250 mg powder containing 5 × 109 colony forming units of S. boulardii per sachet. Up to 2 years old, 2 sachets/day; > 2 years old, 3 sachets/day)  Lactulose + probiotic: same as above.  Treatment duration: 12 weeks  All patients: glycerin enemas for disimpaction before the intervention.  Drug changes were made when there was poor treatment outcome, poor compliance, and/or other side effects. | Def: ≥ 3 defecations per week (and in toilet-trained children, no incontinence episodes)  Time: 12 weeks | Reported | Def: frequency per week, mean (SD)  Time: 2 weeks |
| Olgac 2013 (40) | Age 4 – 16 years, N=61, Rome III | Lactulose: 1 mL/kg/d Probiotic: 10^8 CFU L. reuteri DSM 17938 per day  Treatment duration: 4 weeks  Toilet training and dietary advice were given.  Rescue medication: enema or MgO for no defecation >3 days | Not reported | Reported | Def: frequency per week, mean (SD)  Time: 4 weeks |
| **Magnesiumoxide (MgO) vs probiotics** | | | | | |
| Bu 2007 (41) | Age 0 - 10 years. N=45, FC defined as ‘stool frequency of <3 times per week for >2 months  and at least one of the following minor criteria: anal fissures  with bleeding due to constipation, fecal soiling, or passage of  large and hard stool)’ | MgO: 50mg/kg/d  Probiotics: 8x10^8 CFU/d L. rhamnosus lcr35 Placebo: starch in content  Treatment duration: 4 weeks  Rescue medication: Lactulose (1mL/kg/d) if no defecation >3 days and glycerin enema if no defecation >5 days | Def: ≥ 3 spontaneous defecations per week with no episodes of fecal soiling in the fourth week.  Time: 4 weeks | Reported | Def: frequency per week, mean (SD)  Time: 4 weeks |
| Kubota 2020 (42) | Age 6 months – 6 years, N=60, Rome IV | MgO + placebo: 30 mg/kg/day + lactose hydrate (placebo)  Probiotics + placebo:  10^8 CFU L. reuteri DSM 17938 in 5 drops oil suspension + lactose hydrate  MgO + probiotics: : 10^8 CFU L. reuteri DSM 17938 in 5 drops oil suspension twice a day + MgO (30mg/kg) plus lactose hydrate + lactulose hydrate  Treatment duration: 4 weeks  Rescue medication: glycerin suppository for no defecation >3 days | NR | Not reported | Def: change from baseline to endpoint as least square mean.  Time: 4 weeks |
| **Liquid paraffin vs herbal medicine** | | | | | |
| Mozaffarpur 2012 (43) | Age 4 – 13 years, N=81, Rome III | Liquid paraffin: ml/kg/day in 2 doses Herbal: cassia fistula emulsion 0.1 g/kg/day in 3 doses, adjusted to response  Treatment duration: 3 weeks  The treatments started with demystification.  If any fecal mass was found, disimpaction was done with normal  saline.  Regular toilet sittings for 5 minutes after each meal and diet changes were recommended to all the children.  Excluded when ‘acceleration of constipation’. | Def: not fulfilling Rome III criteria anymore  Time: 3 weeks | Reported | Def: frequency per week, mean (SD)  Time: 3 weeks |
| **Liquid paraffin vs synbiotics** | | | | | |
| Khodadad 2010 (44) | Age 4 – 12 years, N=97, Rome III | Liquid paraffin: 1.5mL/kg/day  Synbiotics: 1x10^9 CFU multispecies probiotic and fructo-oligosaccharides  Treatment duration: 4 weeks  Dietary and  toilet training advice was given to all patients similarly. Toilet training consisted of sitting on  the toilet 3 times per day for 5 minutes after  each meal. | Def: ≥3 BMs per week, ≤2 incontinence per month and no abdominal pain  Time: 4 weeks | Reported | Def: frequency per week, mean (SD)  Time: 4 weeks |
| **Prucalopride vs placebo** | | | | | |
| Mugie 2014 (45) | Age 6 months – 18 years, N=215, Rome III | Prucalopride: <50 kg 0.04 mg/kg once daily, >50 kg 2 mg tablet once daily  Placebo: <50 kg 0.04 mg/kg once daily, >50 kg 2 mg tablet once daily  If the child was <50 kg, dose could be increased to 0.06 mg/kg or decreased to 0.02 mg/kg after 4 weeks, based on treatment response and the presence of safety/ tolerability concerns.  Treatment duration: 8 weeks  Rescue therapy: no bowel movement for 3 or more consecutive days, 5 mg bisacodyl or 7.5 mg/mL sodium picosulfate droplets (1 droplet per 5 kg body mass) was allowed. | Def: mean spontaneous bowel movement frequency of 3 or more/week and a mean fecal incontinence frequency of 1 or less/2 weeks during weeks 5–8 of the double-blind period. Fecal incontinence was taken into account only after the acquisition of toileting skills.  Time: 8 weeks | Reported | Def: frequency per week, change from baseline value to mean value across weeks 1-8, mean (SD)  Time: 8 weeks |
| **Lubiprostone vs placebo** | | | | | |
| Benninga 2022 (46) | Age 6 – 17 years, N=606 , Rome III | Lubiprostone: <50 kg, doses of 12 micrograms twice/day. 50 kg or more, doses of 24 micrograms twice/day. Doses needed to be administered at least 5 hours apart with meals and more than 8 ounces (240 mL) of fluid.  Placebo: same as above.  Treatment duration: 12 weeks  Dose could be increased to 24 microgram in patients who reported no treatment-related AEs and <3 SBMs after 1 week of treatment.  Rescue therapy: prohibited during the first 24h after the first dose of study drug. After that it was allowed if no bowel  movement was observed in the past 3 days.  Study participants  were instructed not to change their diet or  lifestyle. | Def: overall Spontaneous Bowel Movement (SBM) response, defined as an increase of 1 or more SBM/wk compared with baseline and 3 or more SBMs/wk for at least 9 weeks, including 3 of the final 4 treatment weeks.  Time: 12 weeks | Reported | Def: see treatment success. |
| **Lubiprostone vs conventional therapy** | | | | | |
| Elkaragy 2024 (47) | Age 8 – 18 years, N=280, Rome IV | Lubiprostone: weight-based dose calculated for their weight at the time of enrolment. Patients weighing <50 and ≥50 kg were administered lubiprostone 8 mcg TID or 24 mcg BID, respectively.  Conventional therapy: patients were randomized to one of three laxative therapies: lactulose 1–3 mL/kg/day divided into two to four times [maximum 60 mL/day], bisacodyl tablet [5mg/tablet] at a dose of 2 tab/day for <12 years or 3 tab/day for ≥12 years, or sodium picosulfate 0.75% drops in a daily dose of 2.5–20 mg/day). | Improvement of constipation manifested as improved SBM defined as having ≥1 SBM/week. Increase in frequency compared with the baseline and maintaining ≥3 SBMs/week for at least 8 weeks, including the last four study weeks and 4 weeks of follow‐up. | Reported | Change in Spontaneous Bowel Movement, mean ± SD |
| **Linaclotide vs placebo** | | | | | |
| Di Lorenzo 2020 (48) | Age 6 – 17 years, N=173, Rome III | Dose A: linaclotide 9 – 18 μg/day (depending on age)  Dose B: linaclotide 18 – 36 μg/day (depending on age) Dose C: 36 – 72 μg/day  (depending on age)  Adult dose: linaclotide 145 μg (12-17 years old)  Placebo: once/day  Treatment duration: 4 weeks | NR | Reported | Def: change from baseline in frequency per week, mean (SD)  Time: 4 weeks |
| Di Lorenzo 2024 (49) | Age 6 – 17 years, N=328, modified Rome III | Linaclotide: 72 μg once/day  Placebo: once/day  Treatment duration: 12 weeks  Advice: dietary changes, adequate fluid intake, increased physical activity, and  adequate time for bowel movements. Patients and caregivers were instructed to  maintain them throughout the study  Rescue therapy: permitted when at  least 72 h had passed since the patient’s previous bowel  movement or when their symptoms became intolerable. | Def: proportion of participants who no longer fulfil modified Rome III criteria for functional constipation at the end of the study intervention period  Time: 12 weeks | Reported | Def: change from baseline in frequency per week, mean (SD)  Time: 12 weeks |
| Di Lorenzo 2024b (50) | Age 2 – 5 years, N=35, modified Rome III | Group 1: linaclotide 18 μg per day  Group 2: linaclotide 36 μg per day  Group 3: linaclotide 72 μg per day  Group 4: placebo  Treatment duration: 4 weeks | Def: not fulfilling Rome III criteria at the end of the treatment period  Time: 4 weeks | Reported | Def: change from baseline in frequency per week, mean (SD)  Time: 4 weeks |
| **Enemas as addition to PEG vs PEG alone** | | | | | |
| Bongers 2009 (51) | Age 8 – 18 years, N=102, FC defined as ‘presence of at least 2 of the 4 symptoms: spontaneous defecation frequency < 3 per week, fecal  incontinence episodes ≥ 2 per week, passage of large-diameter  stools that might obstruct the toilet, and palpable  abdominal or rectal mass on physical examination’ | Enema + PEG: 120 ml sodium-dioctyl sulfosuccinate and sorbitol 3 times/week during the first 3 months. Frequency then reduced by one enema per week every three months.  PEG: starting dose of 0.5 g/kg/day. Insufficient treatment, dose increased to a max of 1.5 g/kg. A rectal enema or bisacodyl suppository of 5 mg was  only prescribed in case of reoccurrence of fecal impaction (control group only).  Education and behavioral strategies were given in both groups.  Treatment duration: 52 weeks | Def: 3 or more bowel movements per week, and less than 1 incontinence episode per week, irrespective of laxative use  Time: 52 weeks | Reported | Def: frequency per week, mean. No SD reported.  Time: 52 weeks |
| **Domperidone as addition to PEG vs PEG alone** | | | | | |
| Dehghani 2014 (52) | Age 0 – 12 years, N=105, Rome III | Intervention: domperidone syrup 0.15 mL/kg three times a day for 3 months + PEG: 0.6 g/kg/day two times a day for 6 months  Control: PEG 0.6 g/kg/day two times a day for 6 months + placebo with the same color, taste, and smell as domperidone with the same dose (as syrup) for 3 months.  Treatment duration: 6 months | Def: not meeting Rome III criteria  Time: 6 months | Reported | Def: number of patients that reported ≥ 3 episodes of defecation per week  Time: 6 months |

**Supplementary Table 4. Characteristics of included non-pharmacological studies**

| **Study ID** | **Participants** | **Intervention** (comparison, dosage, treatment period, concomitant therapy) | **Treatment success** (definition + time of measurement) | **Withdrawals due to AE reported?** | **Defecation frequency** |
| --- | --- | --- | --- | --- | --- |
| **Cow’s milk free diet vs normal diet** | | | | | |
| Iacono 1998 (53) | Age 0 – 6 years, N=65, FC defined as ‘chronic fecal retention (one bowel movement every 3 to 15 days), often associated with abdominal symptoms (abdominal pain, painful defecation, and so forth). | Intervention: soy-milk 5-10L over 2 weeks Control: cow’s milk 5-10L over 2 weeks  Treatment duration: 2 weeks pre cross-over, 1 week wash-out before cross-over, 2 weeks post cross-over  Rescue therapy: no response to the soy-milk diet, high doses of laxatives | NR | Not reported pre-crossover | Def: number of bowel movements per 2 weeks, median (IQR)  Time: 2 weeks  Not reported pre-crossover |
| **Formula hydrolyzed whey + prebiotics vs Formula cow’s milk protein + prebiotics** | | | | | |
| Fabrizio 2022 (54) | Age 28 – 300 days, N=100, FC defined as ‘at least two grade 1 stools (using 5-point stool consistency scale where; hard =1, formed =2, mushy =3, unformed or seedy =4, watery =5) over the last 10-day period OR two or more stools of a minimum grade 2 consistency (using the 5-point stool consistency scale) AND 48 consecutive hours without a bowel movement over the last 10-day period’ | Intervention: formula consisting of hydrolyzed cow’s milk protein and prebiotic blend (polydextrose and galactooligosaccharide)  Control: normal formula based on cow’s milk and prebiotic blend (polydextrose and galactooligosaccharide)  Treatment duration: 2 weeks  Concurrent therapy: laxatives were not prohibited. Oral laxatives (Intervention: n=1, Control: n=1). Rectal stimulation or suppository (Intervention: n=2, Control: n=2) | NR | Reported | Def: frequency per day, mean (SE)  Time: 2 weeks |
| **Addition of cow’s milk free diet to laxative treatment** | | | | | |
| Bourkheili 2021 (55) | Age 4 – 14 years, N=71, Rome III | Intervention: cow’s milk-free and dairy-free diet plus 30 mg/kg/day of calcium syrup (Calciram, Ramo Pharmin Company, IR Iran) for four consecutive weeks  Control: no restrictions in consuming cow’s milk and dairy products  Both groups: PEG 1g/kg/day for four weeks and high-fiber foods  Treatment duration: 4 weeks | Def: not meeting the Rome III criteria  Time: 4 weeks | Reported | Def: number of patients with 2 or less defecations per week  Time: 4 weeks |
| Dehghani 2012 (56) | Age 0 – 14 years, N=140, Rome III | Intervention: Cow’s milk free diet Control: no restrictions in consuming cow’s milk and dairy products  Both groups: PEG 0.5 g/kg/day  Treatment duration: 4 weeks | Def: not meeting Rome III criteria  Time: 4 weeks | Reported | Def: number of patients with 3 or more defecations per week  Time: 4 weeks |
| **Fiber vs placebo** | | | | | |
| Chmielewska 2011 (57) | Age 3 – 16 years, N=80, Rome III | Intervention: glucomannan 2.52 g/day Control: placebo (maltodextrin, 2.52 g/d)  Treatment duration: 4 weeks  Concomitant therapy: 58% was on laxatives during study | Def: ≥3 bowel movements with no episodes of soiling during the last week  Time: 4 weeks | Reported | Def: bowel movements per week, median (IQR)  Time: 4 weeks |
| Weber 2014 (58) | Age 4 – 12 years, N=57, Rome III | Intervention: fiber mixture (fructo-oligosaccharides, inulin, gum Arabic, resistant starch, soy polysaccharide and cellulose) Control: placebo (maltodextrin, 3.8g/d <18kg bw, 7.6g/d >18kg bw) | Def: a patient maintaining normal bowel habits without the use of stool softeners or enemas.  Time: 4 weeks | Reported | Def: bowel movements per day, mean (SD)  Time: 4 weeks |
| Loening-Baucke 2004 (59) | Age 4 – 12 years, N=46, Rome III | Intervention: glucomannan 100 mg/kg/day, max 5 g/day and rounded to the nearest 500mg Control: placebo (maltodextrin)  Treatment duration: 4 weeks and 4 more weeks after cross-over (no washout)  All patients received toilet training. | Def: ≥3 BMs/wk and ≤1 soiling episode/3 wk with no abdominal pain, rated by physician  Time: 8 weeks  No data reported pre cross-over | Reported | Def: frequency of bowel movements per week, mean (SD)  Time: 8 weeks  No data reported pre cross-over |
| Da Silva Souza 2018 (60) | Age 6 – 24 months, N=38, FC defined as ‘the elimination of hard stools associated with one of the following characteristics: pain or straining while passing stools, scybalous stools, cylindrical and cracked or cylindrical and thick stools and stool frequency less than three times per week’ | Intervention: fructo-oligosaccharides, dosage of 6, 9, or 12 g/d based on weight groups of 6.0–8.9 kg, 9.0–11.9 kg or over 12.0 kg, respectively Placebo: maltodextrin, 6, 9, or 12 g/d same weight groups  Treatment duration: 4 weeks | Def: a normal bowel pattern at the end of the study, i.e., predominantly soft, amorphous or cylindrical stools without cracks as well as the absence of pain or difficulty passing stools  Time: 4 weeks | Reported | Def: frequency per week, mean (SD)  Time: 4 weeks |
| **Fiber vs laxatives** | | | | | |
| Cassetari 2018 (25) | Age 5 – 10, N=80, Rome IV | PEG 3350 + electrolytes: dosage NR Sodium picosulfate: dosage NR Green banana biomass (GBB): 30 g per day PEG + GBB: unclear Sodium picosulfate + GBB: unclear  Treatment duration: 8 weeks  Concomitant therapy: dietary advice | NR | NR | Def: number of patients having more than 3 bowel motions per week.  Time: after 8 weeks |
| Kokke 2008 (36) | Age 1 – 13 years, N=135, FC defined as 2 of 4 criteria: stool frequency less than 3 times per week, fecal incontinence 2 or more times per week, periodic passage of large amounts of stool at least once every 7 to 30 days, or a palpable abdominal or rectal mass | Intervention: fiber mixture: 10 g/125 mL Control: lactulose 10 g/125 mL  Patients with a weight <15 kg received 1 bottle (125 mL, 10 g fibers) daily, those with a weight between 15 kg and 20 kg received 2 bottles (250 mL, 20 g) daily, and those with a weight above 20 kg received 3 bottles (375 mL, 30 g) daily. The study product was taken at breakfast and, in the case of 2 or more bottles, also at lunch.  Treatment duration: 8 weeks + 4 weeks weaning period. Total of 12 weeks.  Enema was given in case of rectal impaction before start treatment.  Rescue medication: macrogol 3350 in no improvement after 3 weeks.  If persistent diarrhea was reported, the original dose was reduced by 50%. | NR | Reported | Def: frequency per week. Unclear if median/mean. No measure of spread reported. |
| Quitadamo 2012 (26) | Age 4 – 10 years, N=100, Rome III | Intervention: Fiber mixture: 16,8 g per day, increase up to 22,4 g per day if necessary Control: PEG 3350 + electrolytes: 0,5 g/kg per day, increase up to 1.0 g/kg per day if necessary  Treatment duration: 8 weeks  Concomitant therapy: disimpaction before start treatment.  Rescue therapy: enemas if no defecation for > 3 days | Def: 3 or more bowel movements per week, 2 or higher stool consistency grade on BSFS, absence of fecal incontinence, abdominal pain, pain on defecation, and fecal bleeding.  Time: 8 weeks | Reported | Def: Per week, mean (SD)  Time: 8 weeks |
| Üstündağ 2010 (37) | Age 4 – 16 years, N=68, Rome III | Intervention: partially hydrolyzed guar gum (PHGG), for children between 4-6 years: 3 g/day; 6-12 years: 4 g/day; and 12-16 years: 5 g/day. Control: lactulose: 1 ml/kg/day, in divided doses  Treatment duration: 4 weeks  In case of rectal impaction, an enema was given at the first visit. If persistent diarrhea was reported, the original dose was reduced by 50%. two groups were given an equal diet with fiber. However, as dietary fiber can bind fluid, the group given PHGG was recommended to increase their fluid intake. | Def: soft to formed stool consistency, absence of pain, stool withholding and blood in the stool, and no palpable rectal or abdominal mass.  No data reported | Reported | Def: frequency per week, mean (SD)  Time: 4 weeks |
| Foroughi 2022 (28) | Age 2 – 12 years, N=144, Rome IV | Group 1 (Fiber): Psyllium Seed Husk Powder 6 g per day Group 2 (Laxative): PEG 6 g per day Group 3 (Laxative + probiotics): 6 g per day + 109 CFU bacterial probiotics (mixture of different stems) Group 4 (synbiotics): Psyllium Seed Husk Powder 6 g per day day + 109 CFU bac- terial probiotics (mixture of different stems)  Concomitant therapy: dietary advice and toilet training were provided to all parents. | NR | NR | Def: frequency per week, mean (SD)  Time: 3 weeks |
| **Increased fluid intake vs control** | | | | | |
| Young 1998 (61) | Age 2 – 12 years, N=108, FC defined as ‘simple constipation of a moderate to severe degree as determined by a Constipation Assessment Scale score of 8 or greater’ | Intervention: increased water intake Intervention: increased hyperosmolar liquid intake Control: control  Treatment duration: 3 weeks | NR | NR | Def: frequency per week, mean No SD reported  Time: 3 weeks |
| **Probiotics vs placebo** | | | | | |
| Bu 2007 (41) | Age 0 – 10 years, N=45, FC defined as ‘stool frequency of <3 times per week for >2 months and at least one of the following minor criteria: anal fissures with bleeding due to constipation, fecal soiling, or passage of large and hard stool. | Intervention: 8x10^8 CFU/d *L. casei rhamnosus* lcr35 Control 1: magnesium oxide 50mg/kg/d Control 2: placebo (starch)  Treatment duration: 4 weeks  Rescue therapy: lactulose (1mL/kg/d) if no defecation >3 days and glycerin enema if no defecation >5 days | Def: ≥ 3 spontaneous defecations per week with no episodes of fecal soiling in the fourth week.  Time: 4 weeks | Reported | Def: frequency per week, mean (SD)  Time: 4 weeks |
| Coccorullo 2010 (62) | Age 6 months – 18 years, N=44, Rome III | Intervention: 10^8 CFU *L. reuteri* DSM 17938 in 5 drops oil suspension Control: placebo in 5 drops oil suspension  Treatment duration: 8 weeks  Rescue therapy: glycerin suppository for no defecation >5 days | Def: ≥3 defecations per week  Time: 8 weeks | Reported | Def: frequency per day  Unclear if mean or median and no spread reported  Time: 8 weeks |
| Gan 2022 (63) | Age 4 – 12 years, N=100, Rome III | Intervention: probiotic chewable tablets twice/day containing *L. acidophilus DDS-1^R^ and B. Lactis UABla-12^TM^* 5 × 109 CFU/tablet Control: chewable placebo tablet twice/day  Treatment duration: 4 weeks | NR | Reported | Def: frequency per week, mean  No SD reported  Time: 4 weeks |
| Lojanatorn 2023 (64) | Age 1 – 5 years old, N=39, Rome IV | Intervention: B. clausii 2 billion spores in 5 mL once/day Control: placebo once/day  Treatment duration: 4 weeks  Rescue therapy: sodium chloride enema once if the child did not defecate for three or more consecutive days (10 mL for children aged 1-2 years, and 20 mL for children aged 3-5 years)  Both groups: caregivers were educated on appropriate fiber and fluid intake, toilet training in developmentally appropriate normal children aged >2-3 years | Def: at least 3 defecations per week and stool consistency at least grade 3 on the Bristol stool chart  Time: 4 weeks | Reported | Def: frequency per week, mean (SD)  Time: 4 weeks |
| Tabbers 2011 (65) | Age 3 – 16 years, N=154, Rome III | Intervention: Activia (125g pet pot) with *B. lactis* DN-173 010 at least 4,25*10^9 CFU and yoghurt starter cultures 2 pots per day Control: milk-based, nonfermented dairy product (125 g per pot) 2 pots per day  Treatment duration: 3 weeks  Concomitant therapy: no treatment for FC <2 weeks before start of the study | Def: 3 or more bowel movements per week and <1 fecal incontinence episode in 2 weeks over the last 2 weeks of product consumption  Time: 3 weeks | Reported | Def: increase in bowel movements per week from baseline to week 3, mean (SD)  Time: 3 weeks |
| Tjokronegoro 2020 (66) | Age 4 – 10 years, N=78, Rome III | Intervention: *L. acidophilus, B. longum, and S. thermophylus* 1× 109cfu/day twice/day Control: placebo (maltodextrin) twice/day  Treatment duration: 4 weeks  Both groups: disimpaction with bisacodyl suppositories twice before start treatment | Def: overall improvement was defined as decreased constipation severity score >60% at the end of evaluation.  Time: 4 weeks | Reported | Def: frequency per week, mean (SD)  Time: 4 weeks |
| Wojtyniak 2017 (67) | Age 0 – 5 years, N=94, Rome III | Intervention: *L. rhamnosus* Lcr35 8 × 10^8 CFU Control: placebo (milk powder and 1% magnesium stearate)  Treatment duration: 4 weeks  Rescue therapy: PEG 1.5mg/kg/d as single dose for no defecation >3 days | Def: ≥3 spontaneous stools per week, without episodes of fecal soiling (in toilet-trained children), in the last week of the intervention  Time: 4 weeks | Reported | Def: frequency per week, median (IQR)  Time: 4 weeks |
| Zaja 2021 (68) | Age 10 – 18 years, N=31, Rome IV*  *All included patients had anorexia nervosa and were female | Intervention: *L. reuteri* DSM17938 108 CFU twice daily as chewable tablet Control: placebo  Treatment duration: 3 months  Rescue therapy: glycerin suppository if no defecation for > 5 days  Both groups: conventional nutritional intervention, consisting of serving normal food under the supervision of nurses that calculated the daily caloric intake through 5-6 meals, and additional enteral nutrition (standard polymeric enteral formula) | Def: relief of constipation, defined as a drop-out from Rome-IV criteria  Time: 3 months | Reported | Def: frequency per week, mean and median Unclear if range is IQR or normal range.  Time: 6 months |
| **Probiotics vs laxatives** | | | | | |
| Lee 2022 (39) | Age 6 months – 10 years, N=187, Rome IV | Probiotics: 5 × 109 CFU *S. boulardii* per sachet was used. Up to 2 years old, 2 sachets/day; over 2 years old, 3 sachets/day. Dosage was not adjusted according to clinical outcomes. Laxative only: lactulose (1.34 g/mL), 1 mL/ kg/day. Dosage change was allowed according to any clinical improvement. Probiotics + laxative: 5 × 109 CFU *S. boulardii* + lactulose 1 mL/kg/day.  Treatment duration: 12 weeks  Both groups: glycerin enemas for disimpaction before the intervention  Drug changes were made when there was poor treatment outcome, poor compliance, and/or other side effects. Patients were then counted as withdrawals. | Def: ≥ 3 defecations per week (and in toilet-trained children, no incontinence episodes)  Time: 12 weeks | Reported | Def: frequency per week, mean (SD)  Time: 12 weeks |
| Olgaç 2013 (69) | Age 4 – 16 years. N=61, Rome III | Intervention: 10^8 *CFU L. reuteri* DSM 17938 Laxative: lactulose 1 mL/kg/d  Treatment duration: 4 weeks  Both groups received toilet training and dietary advice.  Rescue therapy: enema or MgO for no defecation >3 days | NR | Reported | Def: frequency per week, mean (SD)  Time: 4 weeks |
| **Probiotics as addition to laxatives** | | | | | |
| Abediny 2016 (70) | Age 4 – 12 years, N=90, Rome III | Intervention: *multispecies probiotic* + PEG4000 (0.7-1.5 g/kg/d) Control: PEG4000 (0.7-1.5 g/kg/d)  Treatment duration: 4 weeks | NR | NR | Def: frequency per week  No data reported. |
| Banaszkiewicz 2005 (71) | Age 2 – 16 years, N=84, FC defined as ‘<3 BMs per week for at least 12 weeks’ | Intervention: 10^9 CFU of *Lacticaseibacillus rhamnosus* plus 1 mL/kg/day of 70% lactulose  Control: placebo plus 1 mL/kg/day of 70% lactulose  Treatment duration: 12 weeks  Both groups: rectal disimpaction before start of intervention | Def: ≥3 spontaneous BMs per week with no episodes of fecal soiling  Time: 12 weeks | Reported | Def: frequency per week, mean (SD)  Time: 12 weeks |
| Foroughi 2022 (28) | Age 2 – 12 years, N=144, Rome IV | Group 1 (Fiber): Psyllium Seed Husk Powder 6 g per day Group 2 (Laxative): PEG 6 g per day Group 3 (Laxative + probiotics): 6 g per day + 109 CFU bacterial probiotics (mixture of different stems) Group 4 (synbiotics): Psyllium Seed Husk Powder 6 g per day day + 109 CFU bac- terial probiotics (mixture of different stems)  Concomitant therapy: dietary advice and toilet training were provided to all parents. | NR | NR | Def: frequency per week, mean (SD)  Time: 3 weeks |
| Jadrešin 2018 (72) | Age 2 – 16 years, N=33, Rome III | Intervention: 10^8 CFU *L. reuteri* DSM 17938 plus lactulose 1-3ml/kg/d. Control: placebo plus lactulose 1-3ml/kg/d  Treatment duration: 12 weeks | Def: absence of symptoms at the end of study  Time: 12 weeks | Reported | Def: frequency  No data reported  Time: 12 weeks |
| Kubota 2020 (42) | Age 6 months – 6 years, N=60, Rome IV | Probiotics: 10^8 CFU *L. reuteri* DSM 17938 in 5 drops oil suspension twice a day plus lactose hydrate Laxative: MgO (30mg/kg) and lactose hydrate only Probiotics + Laxative: 10^8 CFU *L. reuteri* DSM 17938 in 5 drops oil suspension twice a day plus MgO (30mg/kg) plus lactose hydrate  Treatment duration: 4 weeks  Rescue therapy: glycerin suppository for no defecation >3 days | NR | NR per group | Def: change from baseline to endpoint, least square mean (95% CI) |
| Russo 2017 (73) | Age 4 – 12 years, N=55, Rome III | Intervention: probiotic mixture (3 strains of *bifidobacteria*) plus PEG4000 0.4-0.8 g/kg/d Control: PEG4000 0.4-0.8 g/kg/d  Treatment duration: 8 weeks  Both groups: toilet training  Rescue therapy: enema for no defecation >3 days | Def: ≥3 defecation per week, stool consistency ≥ grade 3 on BSFS, and no episodes of abdominal pain, fecal incontinence, painful defecation, and rectal bleeding  Time: 8 weeks | Reported | Def: frequency per week, mean (SD)  Time: 8 weeks |
| Wegner 2018 (74) | Age 3 – 7 years, N=129, Rome III | Intervention: 10^8 CFU *L. reuteri* DSM 17938 plus 10g/d PEG Control: placebo plus 10g/d PEG  Treatment duration: 8 weeks  Rescue therapy: enema after 5 days without defecation | NR | Reported | Def: frequency per week, mean (SD)  Time: 8 weeks |
| Sadeghzadeh 2014 (75) | Age 4 – 12 years, N=56, Rome III | Intervention: *multispecies probiotic* of 7 strains plus lactulose (1 mL/kg/d) Control: placebo plus lactulose (1 mL/kg/d)  Treatment duration: 4 weeks | NR | Reported | Def: comparison bowel movements between beginning and end of 4th week. Unclear if per week/per day, or if its increase in defecation  Time: 4 weeks |
| **Probiotics as addition to a goat yoghurt** | | | | | |
| Guerra 2011 (76) | Age 5 – 15 years, N=60, Rome III | Intervention: goat yogurt supplemented with 10^ 9 CFU/mL *B. longum* daily Control: goat yogurt only (with classical yogurt starters, *Lactobacillus delbrueckii subspecies bulgaricus* and *Streptococcus thermophilus*)  Treatment duration: 5 weeks | NR | Reported | Def: grouped per category (≤2, 3-6, ≥7 bowel movements per week)  Time: Grouped per category (≤2, 3-6, ≥7 BM’s)/wk  Time: 5 weeks  No data reported, only with figure. |
| **Formula intact protein + probiotic + PEG vs Formula hydrolyzed whey protein + PEG** | | | | | |
| Sevilla 2022 (77) | Age 12 – 32 months, N=96, Rome III | Intervention: Test formula (Friso Comfort Next) consisted of intact protein, 20% milk fat, a fibre mixture of galacto-oligosaccharides (GOS), inulin and carob bean gum (CBG), 100% lactose and a probiotic (B. lactis HN019). Three times a day.  Control: control formula (Similac Comfort) consisted of partially hydrolysed whey (pHW), 2’-fucosyl- lactose (2’-FL) and reduced lactose compared to the test formula. Three times a day.  Treatment duration: 8 weeks  Concurrent therapy both groups: disimpaction with PEG3350 1.5 g/kg/day and PEG3350 during first 4 weeks. First 2 weeks 0.4 g/kg/day, last 2 weeks 0.8 g/kg/day | Def: meeting less than 2 of the Rome III criteria (inverted from paper: data were presented as patients still fulfilling Rome criteria)  Time: 8 weeks | Reported | Def: frequency per week, mean (SD)  Time: 8 weeks |
| **Formula with prebiotics and hydrolyzed whey protein vs standard formula** | | | | | |
| Bongers 2007 (78) | Age 3 – 20 weeks, N=38, FC defined as ‘at least one of the following symptoms: defecation frequency < 3/week; painful defecation; abdominal or rectal palpable mass | Intervention: infant formula (Omneo/Conformil), mix of prebiotic fibres (galacto-oligosaccharides and long chain fructo-oligosaccharides), sn-2 palmitic acid and hydrolyzed whey protein Control: standard formula  Treatment duration: 3 weeks | NR | Reported | Def: frequency per week, mean (SD)  Time: 3 weeks |
| Savino 2005 (79) | Age 0 – 16 weeks, N=123, FC defined as ‘stool frequency of less than 1 stool a day’ | Intervention: infant formula (Omneo/Conformil), mix of prebiotic fibres, (galacto-oligosaccharides and long chain fructo-oligosaccharides), sn-2 palmitic acid and hydrolyzed whey protein Control: standard formula  Treatment duration: 2 weeks | NR | Reported | Def: frequency per day, mean (SD)  Time: 2 weeks |
| **Synbiotics vs placebo** | | | | | |
| Baştürk 2017 (80) | Age 4 – 18 years, N=155, Rome III | Intervention: symbiotic preparation consisting of probiotic mixture *(Lactobacillus casei, L. rhamnosus, L. plantarum, and Bifidobacterium lactis)* and prebiotics *(fiber, polydextrose, fructo-oligosaccharides, and galacto-oligosaccharides)* Control: placebo  Treatment duration: 4 weeks  Both groups: received toilet training and dietary advice.  Rescue therapy: fleet enema (paraffin oil 15-30 mL/y) | Def: complete benefit by resolution of all complaints of the patients ( weekly number of defecation ≥ 3, softening in the stool consistency (Bristol ≥ 4 points), and weekly encopresis ≤ 1)  Time: 4 weeks | Reported | Def: Number of patients with >3 stools per week  Time: 4 weeks |
| **Synbiotics vs laxative** | | | | | |
| Khodadad 2010 (44) | Age 4 – 12 years, N=97, Rome III | Group 1 (Synbiotics): 1x10^9 CFU multispecies probiotic and fructo-oligosaccharides Group 2 (Laxative): Liquid paraffin 1.5 mL/kg/day Group 3 (Synbiotics + laxative): 1x10^9 CFU multispecies probiotic and fructo-oligosaccharides + Liquid paraffin 1.5 mL/kg/day  Treatment duration: 4 weeks  Dietary and toilet training advice was given to all patients similarly. Toilet training consisted of sitting on  the toilet 3 times per day for 5 minutes after  each meal. | Def: ≥3 BMs per week, ≤2 incontinence per month and no abdominal pain  Time: 4 weeks | Reported | Def: frequency per week, mean (SD)  Time: 4 weeks |
| **Behavioral therapy as addition to conventional laxative treatment** | | | | | |
| Van Dijk 2008 (81) | Age 4 – 18 years, N=134, FC defined as ‘2 of 4 criteria: defecation frequency < 3 times per week, fecal incontinence ≥ 2 times per week, passage of large amounts of stool at least once every 7 to 30 days (large enough to clog the toilet), or a palpable abdominal or rectal fecal mass | Intervention: behavioral therapy (12 visits in 22 weeks) + conventional pharmacological therapy as the control group Control: disimpaction with enemas at start, maintenance PEG3350 1-2 sachet/day (1 sachet 10 g) and if necessary enema or bisacodyl suppositories, same FU sessions.  Both groups: received toilet training  Rescue therapy: enema of suppository if no defecation > 3 day | Def: ≥3 BM/week and ≤ 1 episodes of fecal incontinence per 2 weeks irrespective of laxative use.  Time: 22 weeks | Reported | Def: frequency per week, mean (95% CI)  Time: 22 weeks |
| **Biofeedback as addition to conventional laxative treatment** | | | | | |
| Loening-Baucke 1990 (82) | Age 5 – 16 years old, N=43, FC defined as ‘≥2 soiling episodes per week, evidence of a huge amount of fecal material in the rectal ampulla at rectal examination, and abnormal defecation (abnormal contraction of the external anal sphincter and pelvic floor during defecation attempts)’ | Intervention: Addition of biofeedback (2-6 weekly sessions) to magnesiumhydroxide. Control: disimpaction + magnesiumhydroxide (dose adjusted to have daily bowel movement without soiling)  Treatment duration: 6 months  Both groups: received toilet training | Def: ≥3 bowel movements per week and ≤2 soiling episodes per month while not receiving laxatives for 4 weeks  Time: 7 months | Reported | NR |
| Sunic-Omejc 2002 (83) | Age 5 – 15 years, N=49, FC defined as ‘meet at least two of the following criteria: defecation frequency less than 3 times per week, two or more episodes of soiling and/or encopresis per week, periodic evacuation of large volume stools, at least once every 7 – 10 days, and palpable abdominal rectal mass’ | Intervention: addition of biofeedback (1 session + home exercises) to lactulose. Control: lactulose with dose titration and weekly follow-up  Treatment duration: 12 weeks  Both groups: received toilet training and dietary advice for high fiber diet  Rescue therapy: enema if no defecation for more than 3 days. | Def: ≥3 bowel movements per week and ≤2 soiling episodes per month without laxatives  Time: 12 weeks | Reported | NR |
| Van der Plas 1996 (84) | Age 5 – 16 years, N=192, FC defined as ‘two of these four criteria: stool frequency less than three per week, two or more soiling and/or encopresis episodes per week, periodic passage of very large amounts of stool at least once every 7–30 days, or a palpable abdominal or rectal mass’ | Intervention: addition of biofeedback (5 sessions) to lactitol. Control: first disimpcation with 3-7 days enema. Maintenance treatment with lactitol betagalactoside sorbitol and weekly FU  Treatment duration: 6 weeks | Def: ≥3 bowel movements per week and ≤2 soiling episodes per month while not receiving laxatives for 4 weeks  Time: 6 weeks | Reported | NR |
| **Biofeedback vs no biofeedback** | | | | | |
| Castilla 2021 (85) | Age range unclear. Median age (IQR) 10.5 (6), N=25, Rome IV with no response to pharmacological therapy (for more than two years) | Intervention: biofeedback - no further detail provided, apart from that 10 sessions (mean) were delivered per patient Control: no biofeedback. No further information. | Def: ≥3 bowel movements per week and ≤2 soiling episodes per month while not receiving laxatives for 4 weeks  Time: unclear | NR | NR |
| **Biofeedback at home + in laboratory vs biofeedback in laboratory** | | | | | |
| Croffie 2005 (86) | Age 6 – 14 years, N=36, FC defined as ‘defined as less than 3 bowel movements per week, with or without overflow incontinence, and not improving, despite compliance with conventional therapy, including laxative and toilet behavior modification, for at least 6 months’ | Intervention: addition of biofeedback at home daily with portable EMG to biofeedback in laboratory. Control: biofeedback in laboratory (5 sessions at 2-week intervals).  Treatment duration: 10 weeks, outcome assessment 8 weeks later.  Concomitant medication: laxatives were continued, weaned after last session  Both groups: toilet training was previously advised. | Def: ≥3 bowel movements per week with no discomfort and <2 soiling episodes per month, and no, or only rare use of laxatives.  Time: 4 months | NR | Def: number of bowel movements per week, mean. Unclear if SD or SE.  Time: 4 months |
| **Pelvic physiotherapy + standard medical care vs standard medical care** | | | | | |
| Van Engelenburg 2017 (87) | Age 5 – 15 years, N=53, Rome III | Intervention: addition of pelvic floor physiotherapy (max 6 sessions in 6 months) to PEG  Control: standard medical care including education, demystification, dietary advice, toilet training, keeping track of bladder and bowel diaries, and when needed prescription of PEG (PEG 0.3–0.8 g/kg body weight per day).  Treatment duration: 6 months  Concomitant: both groups received disimpaction with high dose PEG if large fecal mass was present at intake  Both groups: received toilet training and dietary advice on effective fiber and fluid intake | Def: absence of FC according to the 6 Rome III criteria, irrespective of PEG use  Time: 6 months | Reported | Def: number of patients with 3 or more bowel movements per week (of the patients who had <3 bowel movements per week at baseline)  Time: 6 months |
| **Abdominal muscle training/breathing exercises/abdominal massage + laxative vs laxative** | | | | | |
| Silva 2013 (88) | Age 4 - 18 years, N=72, Rome III | Intervention: addition of physiotherapy: isometric training of the abdominal muscles, diaphragmatic breathing exercises and abdominal massage (twice weekly sessions). Control: disimpaction with enema 1-5 days, maintenance magnesium hydroxide  Treatment duration: 6 weeks  Both groups: received toilet training and dietary advice on high fiber intake and water intake  Rescue therapy: enema if needed | NR | Reported | Def: number of days per week with defecation, mean (SD)  Time: 6 weeks |
| **Manual therapy vs laxative** | | | | | |
| Blanco Diaz 2020 (89) | Age 2 – 14 years, N=47, Rome III | Intervention: manual physical therapy was performed by a physiotherapist. Nine 30 minute sessions, weekly in the first two months, biweekly in the third month.  Control: PEG (0.5 g/kg/day, range 0.2–0.8) 2 months until obtaining a regular defecation habit and followed by a phase of medication withdrawal.  Treatment duration: 3 months  Concurrent therapy in both groups: Both groups(all patients): (1) 3 days disimpaction with PEG (1-1.5g/kg/day in 2 doses), (2) behavioral management consisting of modification of defacatory habits, establishing routine of visiting bathroom after meals, (3) diet rich in fibers and generous liquid intake | NR | NR | Def: frequency per week, median (IQR)  Time: 3 months |
| **Abdominal transcutaneous electrical stimulation vs sham therapy** | | | | | |
| Clarke 2009 (90) | Age 7 – 18 year, N=33, Rome II | Intervention: 12x20 minutes session of abdominal interferential electrical stimulation Control: sham stimulation  Treatment duration: 4 weeks  Concomitant therapy: there was medication use in 26/33 children | NR | NR | NR |
| **Parasacral nerve stimulation vs sham therapy** | | | | | |
| De Abreu 2021 (91) | Age 5 – 17 years, N=40, Rome IV | Intervention: standard urotherapy + parasacral nerve stimulation (20 minute session, 3 times a week, 20 sessions in total) Control: standard urotherapy + sham parasacral nerve stimulation  Treatment duration: 7 weeks  Both groups: toilet training and dietary advice on fiber rich foods | Def: Number of patients without FC according to Rome IV after treatment  Time: 7-9 weeks | Reported | Def: number of patients with less than two bowel movements per week  Time: 7-9 weeks |
| **Abdominal electrical stimulation + pelvic floor muscle exercises (PFME) vs pelvic floor muscle exercises** | | | | | |
| Ladi-Seyedian 2020 (92) | Age 5 – 13 years, N=34, Rome IV | Intervention: abdominal interferential electrical stimulation (twice weekly, 5 weeks) and pelvic floor muscle exercises Control: pelvic floor muscle exercises (same sessions).  Treatment duration: 5 weeks  Concomitant therapy: mild laxatives were advised if refractory to diet intervention  Both groups: received toilet training and dietary advice | Def: number of patients not fulfilling Rome IV criteria  Time: 6 months | Reported | Def: number of bowel movements, mean (SD)  Time: 6 months |
| Sharifi-Rad 2018 (93) | Age 5 – 13 years, N=90, Rome III | Intervention: abdominal interferential electrical stimulation (twice weekly, 5 weeks) and pelvic floor muscle exercises Control: sham stimulation and pelvic floor muscle exercises  Treatment duration: 5 weeks  Concomitant therapy: PEG if necessary. | Def: number of patients not fulfilling Rome III criteria  Time: 6 months | Reported | Def: Number of bowel movements, median (IQR)  Time: 6 months |
| **Abdominal electrical stimulation + standard therapy vs standard therapy** | | | | | |
| Khan 2020 (94) | Age 3 – 15 years, N=80, Rome IV | Group 1: Addition of abdominal cryotherapy (-10 °С) 6-10 minutes daily for 10 sessions Group 2: Addition of abdominal percutaneous electroneuro-stimulation 6-10 minutes daily for 10 sessions Group 3: Addition of abdominal cryotherapy and percutaneous electroneuro-stimulation Group 4: standard therapy: laxatives, diet, probiotics, choleretic drugs, enzymes  Treatment duration: 10 days | Def: independent stools and no encopresis  Time: unlcear | NR | NR |
| **Tibial nerve stimulation + pelvic floor muscle exercises vs pelvic floor muscle exercises (PFME)** | | | | | |
| Yu 2023 (95) | Age 4 – 14 years, N=82, Rome IV | Intervention: percutaneous Tibial Nerve Stimulation (PTNS) with (PFE) twice daily for 4 weeks. Control: Sham PTNS + PFE. PFE was performed using an electromyography biofeedback method, in which an electrode is inserted through the anus. 20-40 hours of progressive resistance training. These hours would be best spread over 4 weeks, with 15 minutes of exercises twice per day.  Treatment duration: 4 weeks | Def: Full remission was defined as SBM ≥3 per week along with most or all secondary outcomes recovered. Improvement was defined as SBM ≥3 per week with at least 1 secondary outcome recovered.  Time: 16 weeks (4 weeks treatment, 12 weeks follow-up) | Reported | Def: changes in spontaneous bowel movements per week from baseline, mean (95% CI) |
| **Herbal medicine vs laxative** | | | | | |
| Dehghani 2019 (18) | Age 4 – 12 years, N=92, Rome III | Intervention: Black Strap Molasses syrup (40%w/v) 1 mL/kg body weight/day Laxative: PEG syrup (40% w/v) 1 mL/kg body weight/day  Treatment duration: 4 weeks  Concomitant therapy: toilet training and nutritional advice in both groups. | Def: not fulfilling the Rome III criteria  Time: 4 weeks | Reported | Def: number of patients with ≤2 BM/week  Time: 4 weeks |
| Esmaeilidooki 2016 (19) | Age 2 – 15 years, N=109, Rome III | Intervetion: Cassia Fistula‘s Emulsion 1 cc/kg per day Laxative: PEG 4000: 0.7 – 0.8 g/kg per day  Treatment duration: 4 weeks | Def: No longer fulfilling Rome III criteria  Time: 4 weeks | Reported | Def: frequency per week, mean (SD)  Time: 4 weeks |
| Nasri 2022 (20) | Age 2 – 15 years, N=120, Rome IV | Intervention: LaxaPlus Barij® 1 mL/kg daily divided into three doses for <30 kg, 10 mL three times daily for >30 kg Laxative: PEG 4000 0.7 g/kg three times per day  Treatment duration: 8 weeks | NR | Reported | Def: frequency, unclear if per day or per week, mean (SD)  Time: 8 weeks |
| Imanieh 2022 (21) | Age 1 – 18 years, N=100, Rome IV | Intervention: R. damascene and brown sugar syrup: 0.1g damask rose petals and 0.85g brown sugar per 1 mL solution. Initial dosage was 1 ml/kg per day. Laxative: PEG initial dosage was 1 ml/kg per day.  Both groups: If no response, the dosage was increased to 2 ml/kg.  Treatment duration: 4 weeks | Def: having fewer than two of the ROME IV criteria after treatment  Time: 4 weeks | Reported | Def: number of patients with 2 or fewer defecations per week  Time: 4 weeks |
| Nimrouzi 2015 (22) | Age 2 – 12, N=120, Rome III | Intervention: D. Sophia seed 2 g for 2-4 years old, 3 g for 4-12 years old Laxative: PEG 4000 0.4 g/kg per day  Treatment duration: 8 weeks | Def: Improvement of constipation for at least 3 bowel movements, soft stool and convenient defecation, no soiling and bloody stool per week as well as exiting the Rome III criteria for constipation after the third week.  Time: 8 weeks | Reported | Def: frequency per week, median (IQR)  Time: 3 weeks |
| Saneian 2021 (23) | Age 2 – 15 years, N=60, Rome IV | Intervention: Golghand® 0.5 g/kg per day Laxative: PEG 4000 0.7 g/kg per day  Treatment duration: 8 weeks | NR | Reported | Def: frequency per week, mean (SD)  Time: 8 weeks |
| Tavassoli 2021 (24) | Age 4 – 10 years, N=140, Rome III | Intervention: Viola Flower Syrup: 5 cc 3 times per day Laxative: PEG 1 g/kg per day  Treatment duration: 4 weeks | NR | Reported | Def: frequency per week, mean (SD)  Time: 4 weeks |
| Mozaffarpur 2012 (43) | Age 4 – 13 years, N=81, Rome III | Intervention: cassia fistula emulsion 0.1 g/kg/day in 3 doses, adjusted to response  Laxative: liquid paraffin ml/kg/day in 2 doses  Treatment duration: 3 weeks  The treatments started with demystification.  If any fecal mass was found, disimpaction was done with normal saline.  Regular toilet sittings for 5 minutes after each meal and diet changes were recommended to all the children.  Excluded when ‘acceleration of constipation’. | Def: not fulfilling Rome III criteria anymore  Time: 3 weeks | Reported | Def: frequency per week, mean (SD)  Time: 3 weeks |
| **Herbal vs placebo** | | | | | |
| Cai 2018 (96) | Age 1 – 14 years, N=480, adjusted Rome IV and food retention syndrome (in traditional Chinese medicine) | Intervention: Xiao’er Biantong granules Control: placebo  Treatment duration: 2 weeks  Rescue therapy: glycerine enema if no stool for 5 days (then considered noneffective) | Def: symptom score (consist of def frequency, consistency, straining, fecal incontinence) decrease of at least 90% compared to baseline.  Time: 2 weeks | Reported | Def: number of children with ≥3 bowel movements per week  Time: 2 weeks |
| Qiao 2021 (97) | Age 4 – 14 years, N=200, Rome IV criteria | Intervention: XiaojiDaozhi Decoction, a mixture of 12 herbs. Twice daily, with fasting before a meal. Control: placebo, twice daily  Treatment duration: 8 weeks  Concurrent therapy: toilet training and adjusted diet, fiber intake 20 g/day. | Def: fulfilling 2 of the main outcomes and most of the secondary outcomes (main outcomes: improvement of frequency of defecation without western medication and patient satisfaction with their bowel movements. Secondary outcomes: bowel movements, large diameter or scybalous stools, painful or hard bowel movements, excessive volitional stool retention, and encopresis.  Time: 8 weeks | Reported | Def: frequency per week, mean (SD) |
| **Abdominal and acupressure point massage + traditional Chinese medicine vs traditional Chinese medicine** | | | | | |
| Mao 2015 (98) | Age 4 – 13 years, N=94, Rome III | Intervention: addition of abdominal and acupressure point massage 25-30 min once a day Control: traditional Chinese medicine (Xingqi Daozhi Tongfu Fang) twice a day  Treatment duration: 2 weeks  Concurrent therapy both groups: toilet training, dietary advice to increase water, fiber, vegetable, and fruit intake, and advice to exercise more | Def: completely cured if decrease of severity score ≥95%, defecation frequency if 1/day or back to normal pattern, soft or mushy stools without straining. | NR | NR |
| Xu 2015 (99) | Age 4 – 11 years, N=122, Rome III | Intervention: addition of abdominal and acupressure point massage daily 25-30 min Control: oral administration of traditional Chinese medicine twice daily  Treatment duration: 2 weeks  Concurrent therapy both groups: toilet training, dietary advice to eat light and easy digestible food, increase water, fiber, vegetable and fruit intake, and advice to exercise more. | Def: completely cured, decrease of symptom score ≥95% and bowel frequency once/day or back to normal pattern.  Time: 2 weeks | NR | NR |
| **Foot reflexology massage + toilet/diet/motivation training vs toilet/diet/motivation training** | | | | | |
| Canbulat Sahiner 2017 (100) | Age 3 – 6 years, N=40, Rome III | Intervention: addition of 10 minute foot reflexology massage 5 days a week Control: toilet training, diet advice and motivation training with reward system 30 min once per week  Treatment duration: 4 weeks  Concurrent therapy both groups: toilet training, dietary advice: lot of water and daily fruit and vegetables, honey with water every morning, legumes and max two slices whole wheat bread at least twice per day. Also, pasta, white rice, strawberries, banana, apple, potato, carrot, white bread, biscuits, and cake should not be eaten. | NR | Reported | Def: number of patients with more than 2 bowel movements per week  Time: 4 weeks |
| **Dry cupping vs laxatives** | | | | | |
| Shahamat 2016 (101) | Age 4 – 18 years, N=120, Rome III | Intervention: cupping every other day 8 minutes (14 sessions of which 12 by parents) Control: PEG 0.4 g/kg  Treatment duration: 4 weeks  Concurrent therapy both groups: toilet training, routine nutritional and behavioral recommendations  Rescue therapy: exclusion if no bowel movement for 7 days or fecal impaction at any stage. | Def: not fulfilling the Rome III criteria  Time: 12 weeks | Reported | Def: number of patients with 2 or more bowel movements per week.  Time: 12 week  Data not adequately reported, unclear. |
| **Uniformed Services Constipation Action Plan vs standard care** | | | | | |
| Reeves 2022 (102) | Age 4 – 18 years, N=134 (number of children reaching end of study, number of children that were initially randomized was not reported), Rome IV | Intervention: patients received a Uniformed Services Constipation Action Plan (USCAP) Control: patients treated based on the clinician’s definition for standard of care and did not receive a constipation action plan  Treatment duration: 4 months  Concurrent therapy: all participants were offered the use of a defecation posture modification device (DPMD). 23 subjects (40%) of the treatment group and 40 (55%) of controls reported using the DPMD. The clinic designed constipation education videos that were provided to all participants. | Def: not meeting the Rome IV criteria for functional constipation  Time: 4 months | Unclear: not reported per group or if due to an adverse event | Not reported |

**Supplementary Table 5. Secondary outcomes of included pharmacological studies**

*Disimpaction*

| Study | Painful defecation | Stool consistency | Quality of Life | Fecal incontinence | Abdominal pain | School attendance | Tolerability |
| --- | --- | --- | --- | --- | --- | --- | --- |
| *Enema vs PEG (oral)* | | | | | |  |  |
| Bekkali 2009 | NR | Number of patients with watery stools Enema: 4/41 PEG: 13/39 | NR | Frequency per week  Enema: 4.9 (5.4 )  PEG: 5.7 (5.9) | Number of patients with abdominal pain  Enema: 23/41 PEG: 17/39 | NR | Struggle to administer oral or rectal treatment  Enema: 24/38 PEG: 17/31 |

*Maintenance therapy*

| Study ID | Painful defecation | Stool consistency | Quality of Life | Fecal incontinence | Abdominal pain | School attendance | Tolerability |
| --- | --- | --- | --- | --- | --- | --- | --- |
| PEG vs Placebo | | | | | |  |  |
| Modin 2018 | NR | NR | NR | NR | NR | NR | NR |
| Nurko 2008 | NR | Reported on a scale from 0-4 (0 = too loose, watery to 4 = very hard). Mean (SD)  Dose 0.4: 1.7 (0.6)  Dose 0.8: 1.5 (0.7)  Placebo: 2.4 (0.9) | NR | Episodes of fecal incontinence per week, mean (SD):  Dose 0.4: 1.8 (2.6)  Dose 0.8: 3.5 (7.8)  Placebo: 1.4 (3.7) | Cramping on a scale of 0-4 (0 = none to 4 =  very painful), mean (SD)  Dose 0.4: 0.6 (1.0)  Dose 0.8: 0.4 (0.7)  Placebo: 1.3 (1.3) | NR | NR |
| Thomson 2007 | Not reported pre cross-over | Not reported pre cross-over | NR | Not reported pre cross-over | Not reported pre cross-over | NR | NR |
| PEG vs Lactulose | | | | | | | |
| Dheivamani 2021 | Number of patients with painful defecation:  PEG: 13/50 Lactulose: 24/50 | NR | NR | NR | NR | NR | Tolerability to the study medications on a 4-point Likert scale  PEG:  Poor: 0  Fair: 1  Good: 24  Excellent: 23  Lactulose:  Poor: 1  Fair: 5  Good: 19  Excellent: 20 |
| Dupont 2005 | NR | NR | NR | NR | Abdominal pain disappearance in patients with abdominal pain at baseline.  PEG: 9/11 Lactulose: 3/8 | NR | NR |
| Jarzebicka 2019 | Number of patients with painful defecation:  PEG: 4/51 Lactulose: 2/51 | Stool consistency according to the Bristol Stool Form Scale (BSFS) (scale 1-7). Median (no IQR reported)  PEG: 4  Lactulose: 4 | NR | NR | NR | NR | NR |
| Saneian 2012  *Compares PEG vs Lactulose vs Magnesium hydroxide* | NR | NR | NR | NR | Reported as side effect, prevalence of abdominal pain  PEG: 2  Lactulose: 14  MgOH: 17 | NR | NR |
| Treepongkaruna 2014 | NR | Rating of stool consistency compared to baseline: 0 = harder stool, 1 = no change from baseline, 2 = softer stool. Number of patients with improved stool consistency  PEG: 24/43  Lactulose: 27/44 | NR | NR | Number of cramps per week. Mean (SD)  PEG: 0.14 ± 0.35  Lactulose: 0.43 ± 0.79 | NR | Poor compliance if the patient took <70% of the scheduled amount of medication intake in week 4 or <80% over the entire treatment duration  PEG: 3/44  Lactulose: 3/44 |
| Uhm 2007 | NR | NR | NR | Unclear data | NR | NR | NR |
| Voskuijl 2004 | Number of patients with painful defecation:  PEG: 7/50 Lactulose: 21/50 | Reported, but only in figure. No data available. | NR | Frequency per week. Mean (SD)  PEG: 3.11 (5.41)  Lactulose: 2.84 (3.59) | Number of patients with abdominal pain  PEG: 16/50  Lactulose: 25/50 | NR | Bad palatability according to the patients  PEG: 15/50  Lactulose: 5/50 |
| Wang 2007 | NR | NR | NR | NR | NR | NR | NR |
| PEG vs Magnesium hydroxide | | | | |  |  |  |
| Gomes 2011 | NR | Unclear | NR | Unclear | Unclear | NR | Number of patients with bad compliance: unclear.  Number of patients with persistent refusal of medication PEG: 0/17  MgOH: 4/21 |
| Loening-Baucke 2006 | NR | NR | NR | Fecal incontinence frequency per week, mean (SD). After 12 months.  PEG: 1.4 (3.5)  MgOH: 0.5 (1.6) | Unclear | NR | Number of patients who continued to refuse the drug after 12 months  PEG: 2/39  MgOH: 14/40 |
| Ratanamongkol 2009 | Number of patients with episodes of painful defecations. After 4 weeks.  PEG: 2/47 MgOH: 11/47 | NR | NR | Number of patients with episodes of fecal incontinence. After 4 weeks.  PEG: 1/47  MgOH: 1/47 | Number of patients reporting abdominal pain. After 4 weeks.  PEG: 9/47  MgOH: 14/47 | NR | Compliance rate: number of patients who received more than 80% of the medication throughout the study.  PEG: 41/47  MgOH: 31/47 |
| PEG vs fiber | | | | | |  |  |
| Quitadamo 2012  *Fiber mixture* | Number of patients who reported painful stools  PEG: 4/50 Fiber: 7/50 | NR | NR | NR | NR | NR | Patient acceptance: number of patients who refused to take the drug  PEG: 2/50  Fiber mixture: 14/50 |
| PEG 4000 vs PEG3350 + electrolytes | | | | | |  |  |
| Bekkali 2018 | NR | NR | NR | NR | NR | NR | Withdrawals due to lack of compliance:  PEG 4000: 0  PEG 3350 + E: 1 |
| Savino 2012 | Number of days with painful stools. Mean (SD). Unclear if frequency reported per week or per month.  PEG 4000: 2.3 (3.8)  PEG 3350 + E: 3.2 (4.0) | NR | NR | Number of days with fecal incontinence. Unclear if frequency reported per week or per month.  Mean (SD).  PEG 4000: 0.5 (1.2)  PEG 3350 + E: 0.6 (0.9) | Number of days with abdominal. Unclear if frequency reported per week or per month.  Mean (SD)  PEG 4000: 2.8 (3.8)  PEG 3350 + E: 3.9 (3.7) | NR | Difficulty in administration:  PEG 4000 (N=49): 47 no difficulty, 1 mild difficulty, 1 severe difficulty  PEG 3350 + E (N=42): 22 no difficulty, 17 mild difficulty, 3 severe difficulty  Palatability (5 point scale and ease of administration):  PEG 4000 (N=49): 21 good/very good, 27 not good/not bad, 1 bad/very bad  PEG 3350 + E (N=42): 1 good/very good, 30 not good/not bad, 11 bad/very bad  Compliance (number of patients who took >80% of the described dosage):  PEG 4000: 48/49  PEG 3350 + E: 37/42 |
| PEG vs herbal medicine | | | | | |  |  |
| Dehghani 2019  *Black strap molasses (BSM) (sugarcane extract)* | Number of patients reporting painful *or* hard stools:  PEG: 3/47  BSM: 10/45 | Number of patients reporting painful *or* hard stools:  PEG: 3/47  BSM: 10/45 | NR | NR | NR | NR | NR |
| Esmaeilidooki 2016  *Cassia’s fistula emulsion* | Severity of pain during defecation measured on VAS scale (0-100), mean (SD)  PEG: 6.54 (11.98)  Cassia: 4.74 (8.66) | Stool consistency measured on VAS scale (0-100):  PEG: 14.35 (16.8)  Cassia: 9.48 (14.6) | NR | Frequency per week, mean (SD)  PEG: 1.96 (4.3)  Cassia: 1.02 (3.45) | NR | NR | Compliance of the drugs according to VAS pattern, scoring 1 (very good) to 5 (very bad), mean (SD)  PEG: 1.88 (1.02)  Cassia: 2.33 (1.42)  Dropouts due to taste of drug  PEG: 2/57  Cassia: 3/57 |
| Imanieh 2022  *R. damascena and brown sugar syrup* | No history of painful *or* hard bowel movements after 4 weeks of treatment  PEG: 41/50  R. damascena: 44/50 | No history of painful *or* hard bowel movements after 4 weeks of treatment  PEG: 41/50  R. damascena: 44/50 | NR | Unclear | NR | NR | Feeling of bad taste:  PEG: not reported  R. damascena: 14/50  Dropouts due to bad taste:  PEG: 0/50  R. damascena: 5/50 |
| Nasri 2022  *LaxaPlus Barij®* | Number of patients with existence of pain during defecation  PEG: 19/60  LaxaPlus Barij®: 19/60 | NR | NR | NR | NR | NR | NR |
| Nimrouzi 2015  *D. Sohpia seed* | Frequency of painful defecations per week, median (IQR).  PEG: 0 (0-3)  D. Sophia Seed: 0.5 (0-2) | Number of hard stools per week, median (IQR).  PEG: 2 (0-3)  D. Sophia Seed: : 1 (0-2.75) | NR | Frequency per week, median (IQR).  PEG: 0 (0-0)  D. Sophia Seed: 0 (0-0) | NR | NR | Number of patients who disliked the taste  PEG: 5/53  D. Sophia Seed: 17/56 |
| Saneian 2021  *Goleghand®* | Number of patients with painful defecation  PEG: 6/30  Goleghand®: 9/30 | NR | NR | NR | NR | NR | NR |
| Tavassoli 2021  *Viola flower syrup* | Number of painful defecations per week, mean (SD)  PEG: 0.40 (0.94)  Syrup: 0.25 (1.01) | Number of hard stools per week, mean (SD).  PEG: 0.56 (1.15)  Syrup: 0.53 (1.13) | NR | Number of fecal soiling per week, mean (SD)  PEG: 0.4 (1.25)  Syrup: 0.34 (1.27) | NR | NR | Incidence of unpleasant taste  PEG: 2/66  Syrup: 1/67 |
| PEG vs Liquid paraffin | | | | | |  |  |
| Karami 2009 | NAR | NAR | NR | Frequency per month, mean (SD)  PEG: 3.9 (0.3)  Liquid paraffin: 3.9 (0.3) | NR | NR | NR |
| Rafati 2011 | NR | NR | NR | Number of patients with fecal incontinence at 30^th^ day of treatment  PEG: 12/80  Liquid paraffin: 10/78 | NAR | NR | NR |
| PEG vs microenema | | | | | | | |
| Strisciuglio 2021  *Promelaxin* | NR | Improved stool consistency: patients who experienced an increase, as compared to baseline, of one or more points on the Amsterdam Stool Form Scale (ASFS) or BSFS.  PEG: 37/77  Promelaxin: 38/76 | Only reported quality of life of parents. | NR | NR | NR | Compliance: the ratio between treatment administered vs. planned, mean (SD).  PEG: 84.32% (29.10)  Promelaxin: 85.07% (25.23) |
| Enema as addition to PEG | | | | | | | |
| Bongers 2009  *Enema: sodium-dioctyl*  *sulfosuccinate and sorbitol* | Number of patients with painful defecation  PEG + enema: 11/50  PEG: 17/50 | NR | NR | Number of patients with fecal incontinence of less than 1 per week.  No data reported, only in figure. | Number of patients with abdominal pain  PEG + enema: 17/50  PEG: 22/50 | NR | Only patients with PEG + enema answered the question: “I find the application of a rectal enema terrible.” Based on a 5-point Likert scale.  Very to extremely terrible: 15% of children Quite terrible: 11%  No problem at all: 74% |
| PEG vs fiber vs probiotics | | | | | | | |
| Foroughi 2022 *fiber: psyllium*  *Probiotics: L.reuteri, L.*  *rhamnosus, and Bifidobacterium infantis* | Number of painless bowel movements per week, mean (SD)  PEG: 6,08 (1.079)  PEG + probiotics: 6.36 (0.683)  Fiber: 4.50 (1.483)   Fiber + probiotics: 5.19 (1.261) | NR | NR | NR | NR | NR | NR |
| PEG vs sodium picosulphate (SP) vs fibers | | | | | | | |
| Cassetari 2019 *Fibers: green banana biomass (GBB)* | Number of patients with painful defecation PEG: 4 /16  SP: 2/17  GBB: 4/15  GBB+PEG: 3/16  GBB+SP: 1/16 | Number of patients with BSFS score higher than 2. PEG: 11/16  SP: 13/17 GBB: 13/15  GBB+PEG: 15/16  GBB+SP: 13/16 | NR | Number of patients with > 1 episode of fecal incontinence per week  PEG: 4/16  SP: 5/17 GBB: 5/15 GBB+PEG: 2/16 GBB+SP: 2/16 | Number of patients with abdominal pain  PEG: 2/16  SP: 5/17 GBB: 2/15  GBB+PEG: 2/16  GBB+SP: 4/16 | NR | NR |
| PEG vs dry cupping | | | | | | | |
| Shahamat 2016 | Number of patients with painful or hard bowel movements  PEG: 10/60  Cupping: 7/60 | Number of patients with painful or hard bowel movements PEG: 10/60  Cupping: 7/60 | NR | Number of patients with 1 ≤ episode of fecal incontinence/week  PEG: 50/60  Cupping: 55/60 | NR | NR | NR |
| Lactulose vs placebo | | | | | | | |
| Cao 2018 | NR | Difference in stool consistency from baseline measured by BSFS, mean (range)  Lactulose: 1.6 (0.9 to 2.3)  Placebo: 0.5 (0.2 to 0.9) | NR | NR | Difference in abdominal pain from baseline, based on scale (0=no pain at all, 3=continuous pain), mean (range).  Lactulose: -0.2  (-0.5 to -0.1)  Placebo: -0.1  (-0.3 to -0.1) | NR | NR |
| Lactulose vs fibers | | | | | |  |  |
| Kokke 2008  *Fiber mixture* | NR | BSFS, mean. No SD reported  Lactulose: 4.0  Fiber: 3.6 | NR | Number of patients with 1 or more fecal incontinence episodes per week.  Lactulose: 5/70  Fiber: 9/65 | Abdominal pain (0 =not at all,1 = sometimes, 2 =often, and 3=continuous), mean. No SD reported.  Lactulose: 1.39  Fiber: 1.49 | NR | Number of patients who refused to drink the yoghurt:  Lactulose: 11/70  Fiber: 22/65  Taste, rated on a scale of 1–10, median (range).  Lactulose: 7 (1-10)  Fiber: 8 (1-10) |
| Ustundag 2010  *Partially hydrolysed guargum (PHG)* | NR | BSFS, mean (SD)  Lactulose: 4.3 (0.6)  PHG: 3.9 (0.7) | NR | NR | Number of patients with abdominal pain  Lactulose: 3/33  PGH: 5/35 | NR | NR |
| Lactulose vs liquid paraffin | | | | | | | |
| Farahmand 2007 | NR | NR | NR | Fecal incontinence frequency per week, in the last 4 weeks, mean (SD).  Lactulose: 3 (4.1) Liquid paraffin: 0 (0) | NR | NR | Number of subjects who reported a bad palatability of study medication  Lactulose: 8/120  Liquid paraffin: 5/127 |
| Urganci 2005 | NR | Stool consistency on a scale of 1-3 (1=hard, 2=firm, 3=loose), mean (SD). During last 4 weeks.  Lactulose: 2,21 (0,4)  Liquid paraffin: 2,29 (0,2) | NR | NR | NR | NR | NR |
| Lactulose vs lactitol | | | | | | | |
| Pitzalis 1995 | Number of patients with painful defecation  Lactulose: 8/24  Lactitol: 6/27 | Number of patients with a stool consistency on a scale of 1-4 (1=hard, 2=normal, 3=soft, 4=liquid).  Lactulose (n=23): hard n=3, normal n=15, soft n=1  Lactitol (n=19): hard n=8, normal n=14, soft n=1 | NR | Number of patients with fecal incontinence  Lactulose: 15/24  Lactitol: 12/27 | Number of patients with abdominal pain  Lactulose: 15/24  Lactitol: 9/27 | NR | *Drug acceptance* (1=bad, 2=mediocre, 3=good, 4=optimal)  Lactulose (n=19): bad n=2, mediocre n=4, good n=9, optimal n=4  Lactitol (n=23): bad n=0, mediocre n=5, good n=14, optimal n=4  *Palatabilitly of the drug* (1=bad, 2=mediocre, 3=good, 4=optimal)  Lactulose (n=19): bad n=2, mediocre n=3, good n=10, optimal n=4  Lactitol (n=23): bad n=0, mediocre n=4, good n=15, optimal n=4 |
| Lactulose vs probiotics | | | | | | | |
| Lee 2022  *S. boulardii* | Number of painful defecations per week, mean (SD).  Lactulose: 3.38 (1.23)  Probiotics: 2.92 (1.04) | BSFS, mean (SD).  Lactulose: 3.38 (1.23)  Probiotics: 2.92 (1.04) | NR | Frequency per week, mean (SD).  Lactulose: 0.53 (1.69)  Probiotics: 0.96 (3.63) | NR | NR | NAR |
| Olgac 2013  *L. reuteri* | Unclear definition | BSFS, mean (SD)  Lactulose: 3.5 (0.2)  Probiotics: 3.5 (0.2) | KINDL QOL survey (The Improved Quality of  Life Survey for Children and Families), scale of 0-100. Mean, no SD reported.  Lactulose: 77  Probiotics: 78 | Unclear definition | Unclear definition | NR | NR |
| Lactulose as addition to PEG | | | | | | | |
| Ala 2015 | NR | NR | NR | NR | NR | NR | NR |
| Magnesiumoxide vs probiotics | | | | | | | |
| Bu 2007  Compares MgO vs probiotics vs placebo  *Probiotics: L. rhamnosus lcr35* | NR | Percentage of hard stools, mean (SD) MgO: 23.5 (7.9)  Probiotics: 22.4 (14.7)  Placebo: 75.5 (6.1) | NR | Frequency, unclear if per week or per month.  MgO: 2.7 (5.1)  Probiotics: 2.1 (3.8) Placebo: 2.7 (1.4) | Frequency, unclear if per week or per month.  MgO: 4.87 (3.7)  Probiotics: 1.9 (1.6) Placebo: 6.7 (3.3) | NR | NR |
| Kubota 2020  *Compares MgO vs probiotics vs MgO + probiotics*  *Probiotics: L. reuteri* | NR | BSFS, change from baseline to endpoint, least square mean (95% CI)  MgO: 1.61 (0.93 – 2.28)  Probiotics: 0.62 (-0.07 – 1.32)  MgO + probiotics: 0.88 (0.17 - 1.59) | NR | NR | NR | NR | NR |
| Liquid paraffin vs herbal medicine | | | | | | | |
| Mozaffarpur 2012  *Cassia fistula emulsion* | Pain severity, reported on VAS score (0-100), mean (SD)  Liquid paraffin: 20.1 (19.9)  Herbal: 4.8 (8.5) | Stool consistency, reported on VAS score (0-100), 0=soft. Mean (SD)  Liquid paraffin: 25.4 (22)  Herbal: 11.9 (16.8) | NR | Fecal incontinence frequency per week, mean (SD).  Liquid paraffin: 6.4 (11.1)  Herbal: 3 (9.1) | NR | NR | Parents were asked to explain the acceptance and tolerance of drugs on scale of 1-7: taking drug, with willingness = 1.  Vomiting, if anyway takes it = 7. Mean (SD).  Liquid paraffin: 2.4 (1.3)  Herbal: 2.2 1(1.5) |
| Liquid paraffin vs synbiotics | | | | | | | |
| Khodadad 2010 *Multispecies probiotics + fructo-oligosaccharides* | Number of patients with painful defecations  Liquid paraffin: 2/29  Synbiotics: 3/31 | Number of patients with hard stools  Liquid paraffin: 2/29  Synbiotics: 7/31 | NR | Frequency per week, mean (SD)  Liquid paraffin: 0.24 (1.3)  N=29  Synbiotics: 0.06 (0.25)  N=31 | Number of patients with abdominal pain  Liquid paraffin: 4/29  Synbiotics: 2/31 | NR | NR |
| Lubiprostone vs placebo | | | | | |  |  |
| Benninga 2022 | Painfulness of spontaneous bowel movements (4-point scale: 1=mild and 4=severe), mean change from baseline (SD).  Lubiprostone:  -0.81 (1.02) Placebo: -0.65 (1.1) | NR | NR | Frequency per 2 weeks, mean change from baseline (SD)  Lubiprostone: 0.04 (0.37)  Placebo: 0.07 (0.48) | Abdominal pain (4-point scale with 1=mild and 4=severe), mean change from baseline (SD)  Lubiprostone:  -0.42 (0.84)  Placebo: -0.35 (0.76) | NR | NR |
| Lubiprostone vs conventional therapy | | | | | | | |
| Elkargy 2024 | NR | Change in BSF from baseline  minimum–maximum (median).  IG: 0–3 (2) CG: 0–3 (1) | NR | NR | NR | NR | NR |
| Prucalopride vs placebo | | | | | | | |
| Mugie 2014 | Change from baseline of level of defecation pain (scale 0-5), mean (SD)  Prucalopride:  -0.6 (1.36)  Placebo: -0.4 (0.94) | BSFS, mean change from baseline (SD).  Prucalopride: 0.6 (1.41)  Placebo: 0.1 (1.17) | PedsQL questionnaire, mean change from baseline (SD).  *Patient reported*  Prucalopride: 3.9 (13.8)  Placebo: 2.7 (12.4)  *Parent reported*  Prucalopride: 6.5 (13.9)  Placebo: 4.1 (14.2) | Frequency per 2 weeks, mean change from baseline (SD)  Prucolopride: 8.7 (36.85)  Placebo: 13,9 (64.91) | Level of abdominal pain (Wong–Baker Faces Pain Rating Scale 0-5), mean change from baseline (SD)  Prucalopride:  -0.2 (0.76)  Placebo: -0.3 (0.94) | NR | NR |
| Linaclotide vs placebo | | | | | | | |
| Di Lorenzo 2020 | NR | BSFS (1-7), mean change form baseline (SD) Lin: 1.16 (1.51)  Placebo: 0.40 (1.51) | NR | Change from baseline in 4-week fecal incontinence daytime per day, mean (SD) Lin: PEG: 0,17 (0,3) (n=10) Placebo: -0,03 (0,08) (n=11) | Abdominal pain on scale of 0-4 0=none, 4=a lot. Mean change from baseline (SD) Lin: -0.12 (0.88)  Placebo: -0.43 (0.85) | NR | NR |
| Di Lorenzo 2024 | NR | BSFS (1-7), mean (SD) Lin: 3.5 (0.94)  Placebo: 3.08 (0.85) | NR | Number of patients with fecal incontinence at the end of treatment (12 weeks)  Lin: 28/136 Placebo: 26/136 | Abdominal pain on scale of 0-4 0=none, 4=a lot. Mean change from baseline (SD)  Lin: -0.53 (0.76)  Placebo: -0.34 (0.73) | NR | NR |
| Di Lorenzo 2024b | NR | BSFS (1-7), mean change form baseline (SD) Lin 72μg: 1.71 (1.90) (n=10)  Placebo: 0.60 (0.94) (n=7) | NR | Proportion of days with fecal  Incontinence, treatment overall Lin: 0.05 (0.06) (n=10) Placebo: 0 (n=6) | NR | NR | NR |
| Domperidone as addition to PEG | | | | | | | |
| Dehghani 2014 | Number of patients with history of hard and painful bowel movements  PEG + domperidone: 10/52  PEG + placebo: 11/53 | Number of patients with history of hard and painful bowel movements  PEG + domperidone: 10/52  PEG + placebo: 11/53 | NR | Number of patients with ≥1 dirty underwear per week  PEG + domperidone: 10/52  PEG + placebo: 7/53 | NR | NR | NR |

**Supplementary Table 6. Secondary outcomes of included non-pharmacological studies**

| Study | Painful defecation | Stool consistency | Quality of Life | Fecal incontinence | Abdominal pain | School attendance | Tolerability |
| --- | --- | --- | --- | --- | --- | --- | --- |
| Probiotics vs placebo | | | | | |  |  |
| Lojanatorn 2023 | Painful defecation per week, median (IQR) Probiotics: 0,5 (0-2) Placebo: 0 (0-2) | Bristol stool grade, on scale of 1-7 (1= very hard, 7=very loose), mean (SD) Probiotics: 2,8 (1,2) Placebo: 2,8 (1,2) | NR | NR | NR | NR | NR |
| Tabbers 2011 | Number of children with pain during defecation Probiotics: 36/79 Placebo: 31/80 | Mean stool consistency, based on Bristol stool scale (1-7), mean. No SD reported. Probiotics: 3.3 Placebo: 3.5 | NR | Proportion of patients with episodes of fecal incontinence. Probiotics: 27/79 Placebo: 36/80 | Number of children with abdominal pain Probiotics: 43/79 Placebo: 40/80 | NR | NR |
| Tjokronegoro 2020 | Number of patients with painful defecation Probiotics: 8/39 Placebo: 19/39 | Number of patients with normal stool consistency. Probiotics: 27/39 Placebo: 17/39 | NR | Number of patients who had stool incontinence Probiotics: 2/39 Placebo: 7/39 | NR | NR | Compliance was checked by interview and counting the sachets returned by the parents. Not reported what was considered as good compliance. Not reported per group: “63% had good compliance” |
| Zaja 2021 | NR | Number of patients with normal stool consistency. Probiotics: 12/15 Placebo: 14/16 | NR | NR | NR | NR | NR |
| Bu 2007 *L. rhamnosus lcr35 vs Placebo vs MgO* | NR | Percentage of hard stools, mean (SD) Probiotics: 22.4 (14.7) Placebo: 75.5 (6.1) MgO: 23.5 (7.9) | NR | Frequency, unclear if per week or for whole 4 weeks of treatment. Probiotics: 2.1 (3.8) Placebo: 2.7 (1.4) MgO: 2.7 (5.1) | Frequency, unclear if per week or for whole 4 weeks of treatment. Probiotics: 1.9 (1.6) Placebo: 6.7 (3.3) MgO: 4.87 (3.7) | NR | NR |
| Wojtyniak 2017 | Pain during defecation per week, median (IQR) Probiotics: 0.0 (0.0-1.0) Placebo: 0.0 (0.0-1.0) | Bristol Stool Form Scale (1-7), median (IQR). Probiotics: 3.5 (2.8, 4.0) Placebo: 3.7 (3.0, 4.0) | NR | Fecal soiling per week, median (IQR) Probiotics: 0.0 (0.0, 0.0) Placebo: 0.0 (0.0, 0.0) | Abdominal pain, median (IQR) Probiotics: 0.0 (0.0, 0.0) Placebo: 0.0 (0.0, 0.0) | NR | NR |
| Gan 2022 | NR | Bristol Stool Score (3-5 = normal stools) For each child, the ratio of the number of occurrences to the total number of stools (%) was calculated.  Probiotics: 80% normal stools Placebo: 61 normal stools | NR | NR | NR | NR | NR |
| Coccorullo 2010 | NR | Reported hard stools  Probiotics: 18.2% Placebo: NR | NR | NR | NR | NR | Excellence compliance: no violation of the protocol for the study product intake Probiotics: 94.6%  Placebo: 86.9% |
| Probiotics vs laxatives | | | | | | | |
| Kubota 2020 *L. reuteri vs MgO* | NR | BSFS, change from baseline to endpoint, least square mean (95% CI) Probiotics: 0.62 (-0.07 – 1.32)  MgO: 1.61 (0.93 – 2.28) | NR | NR | NR | NR | NR |
| Lee 2022 *S. boulardii vs Lactulose* | Number of painful defecations per week, mean (SD). Probiotics: 0.68 (0.75) Lactulose: 0.48 (0.5) Probiotics + Lactulose: 0.64 (0.97) | BSFS (1-7). Mean (SD). Probiotics: 2.92 (1.04) Lactulose: 3.38 (1.23) Probiotics + lactulose: 3.54 (1.32) | NR | Frequency per week, mean (SD) Probiotics: 0.96 (3.63) Lactulose: 0.53 (1.69) Probiotics + Lactulose: 0.56 (1.66) | NR | NR | Number of patients with drug changes due to poor treatment outcome, poor compliance, and/or other side effects. Unclear what the exact reasons were for drug change per patient.  S. boulardii: n=23  Lactulose: n=3  Combination: n=7 |
| Olgaç 2013 *L. reuteri vs lactulose* | Reduction rate in % (no further information) Probiotics: 80% Lactulose: 68% | BSFS (1-7). Mean (SD). Probiotics: 3.5 (0.2) Lactulose: 3.5 (0.2) | KIND QOL survey (0-100), high scores = high QoL. Mean, but no SD reported.  Probiotics: 77 Lactulose: 78 | Reduction rate in % (no further information) Probiotics: 8% Lactulose: 14% | Reduction rate in % (no further information) Probiotics: 64% Lactulose: 29% | NR | NR |
| Probiotics as addition to laxatives | | | | | | | |
| Abediny 2016 *Multispecies and PEG* | NR | Number of patients with hard stools Probiotics+PEG: 5/45 PEG: 8/45 | NR | NR | Number of patients with abdominal pain Probiotics+PEG: 7/45 PEG: 16/45 | NR | NR |
| Banaszkiewicz 2005 *Lactobacillus GG and lactulose* | NR | NR | NR | Frequency per week, mean (SD)  Probiotics+lactulose: 0.8 (1.8) Probiotics+placebo: 0.3 (0.9) | NR | NR | NR |
| Jadrešin 2018 *L. reuteri and lactulose* | NR | NR | NR | NR | Abdominal pain on scale (scale not reported), median (IQR) Probiotics + lactulose: 0.2 (0-2) Lactulose: 0.5 (0-2) | NR | Compliance discussed with withdrawals but no reasons given. Probiotics + lactulose: 2/18 Lactulose: 4/15 |
| Foroughi 2022 *Laxative: PEG* *Fiber: psyllium Probiotics: L.reuteri, L. rhamnosus, and Bifidobacterium infantis* | Painless bowel movements per week, mean (SD) Probiotics+PEG: 6.36 (0.683) PEG: 6.08 (1.079)  PEG + probiotics: 6,36 (0,683) Psyllium: 4,50 (1,483)  Synbiotics: 5,19 (1,261) | NR | NR | NR | NR | NR | NR |
| Kubota 2020  *Multispecies and Magnesiumoxide* | NR | Change from baseline to endpoint, least square mean (95% CI) Probiotics+MgO: 0.88 (0.17, 1.59) MgO: 1.61 (0.93 – 2.28) | NR | NR | NR | NR | NR |
| Russo 2017  *Multispecies and PEG* | NR | BSFS (1-7), mean (SD) Probiotics+PEG: 4.2 (0.5) PEG: 4.2 (0.5) | NR | Number of patients with fecal incontinence Probiotics + PEG: 2/25 PEG: 1/25 | Number of patients with abdominal pain Probiotics +PEG: 1/25 PEG: 2/25 | NR | Refused due to bad taste:  Probiotic+PEG: 1/25  PEG: 1/25 |
| Sadeghzadeh 2014  *Multi species and lactulose* | NR | Hard, normal, soft stools (1-3), mean (SD) Probiotics+lactulose: 0.88 (0.45) Lactulose+placebo: 0.63 (0.50) | NR | Number of patients with fecal incontinence. Only looked at patients who had fecal incontinence before intervention.  Probiotics + lactulose: 4/15 Lactulose+placebo: 7/9 | Number of patients with abdominal pain. Only looked at patients who had abdominal pain before intervention. Probiotics + lactulose: 7/16 Lactulose+placebo: 12/14 | NR | NR |
| Wegner 2018 *L. reuteri + PEG* | Number of patients with painful defecation Probiotics+PEG: 13/65 PEG: 8/64 | Number of patients passing hard stools Probiotics+PEG: 7/65 PEG: 3/64 | NR | Number of patients with presence of fecal incontinence:  Probiotics + PEG: 17/59 PEG: 11/61 | Number of patients with abdominal pain Probiotics + PEG: 19/65  PEG: 25/64 | NR | Withdrawals due to lack of compliance:  Probiotics + PEG: 5/65  PEG: 1/64 |
| Probiotics as addition to goat yoghurt | | | | | | | |
| Guerra 2011 *B. longum* | Defecation pain: No numbers reported | Number of patients with Bristol stool scale < 4 (based on 5 point scale) No numbers reported |  | NR | Abdominal pain: No numbers reported | NR | NR |
| Formula with intact protein + probiotic + PEG vs Formula with hydrolyzed whey + PEG | | | | | | | |
| Sevilla 2022 | Number of subjects indicating to suffer from painful defecation Formula 1: 6/47 Formula 2: 6/48 | Number of subjects who reported to have a hard stool on one or more occasions throughout the intervention: Formula 1: 10/47 Formula 2: 14/48 | NR | Number of subjects indicating to suffer from fecal incontinence (defined as passing stool whilst asleep): Formula 1: 10/47 Formula 2: 14/48 | NR | NR | NR |
| Herbal medicine vs laxative | | | | | | | |
| Dehghani 2019 *Black strap molasses (sugarcane extract) vs PEG* | Number of patients with hard or painful stools BSM: 10/45 PEG: 3/47 | Number of patients with hard or painful stools BSM: 10/45 PEG: 3/47 | NR | NR | NR | NR | NR |
| Esmaeilidooki 2016  *Cassia’s fistula emulsion vs PEG* | Severity of pain measured on VAS score (0-100), mean (SD)  Cassia’s fistula: 4,74 (8,66)  PEG: 6,54 (11,98) | Measured on Visual Analog Scale (0-100). 0 = softer.  Cassia’s fistula: 9,48 (14,6) PEG: 14,35 (16,8) | NR | Frequency per week, mean (SD) Cassia’s fistula emulsion: 1,02 (3,45)  PEG: 1,96 (4,3) | NR | NR | Compliance of the drugs according to VAS pattern, scoring 1 (very good) to 5 (very bad), mean (SD) Cassia’s fistula: 2,33 (1,42)  PEG: 1,88 (1,02)  Dropouts due to taste of drug  Cassia’s fistula: 3/57 PEG: 2/57 |
| Imanieh 2022  *R. damascena and brown sugar syrup vs PEG* | No history of painful or hard bowel movements after 4 weeks of treatment R damascena: 44/50  PEG: 41/50 | No history of painful or hard bowel movements after 4 weeks of treatment R damascena: 44/50  PEG: 41/50 | NR | Unclear data | NR | NR | Number of patients who dropped out due to bad taste R. damascena: 5/50 PEG: 0/50  Feeling of bad taste:  R. damascene: 14/50 PEG: NR |
| Mozaffarpur 2012  *Cassia fistula emulsion vs Liquid Paraffin* | Pain severity, reported on VAS score (0-100), mean (SD) Cassia fistula: 4.8 (8.5) Liquid paraffin: 20.1 (19.9) | Stool consistency, reported on VAS score (0-100), 0=soft. Mean (SD) Cassia’s fistula: 11.9 (16.8) Liquid Paraffin: 25.4 (22) | NR | Frequency per week, mean (SD) Cassia’s fistula: 4.8 (8.5) Liquid paraffin: 20.1 (19.9) | NR | NR | NR |
| Nasri 2022  *LaxaPlus Barij® vs PEG* | Number of patients with existence of pain during defecation LaxaPlus Barij®: 19/60 PEG: 19/60 | NR | NR | NR | NR | NR | NR |
| Nimrouzi 2015  *D. Sohpia seed vs PEG* | Frequency of painful defecations per week, median (IQR) D. Sophia seed: 0,5 (0-2) PEG: 0 (0-3) | Frequency of hard stool per week, median (IQR) D. Sophia seed: 1 (0-2.75) PEG: 2 (0-3) | NR | Frequency per week, median (IQR) D. Sophia seed: 0 (0-0)  PEG: 0 (0-0) | NR | NR | Number of patients who disliked the taste.  D. Sohpia seed: 17/56 PEG: 5/53 |
| Saneian 2021  *Goleghand® vs PEG* | Number of patients with painful defecation Goleghand®: 9/30 PEG: 6/30 | NR | NR | NR | NR | NR | NR |
| Tavassoli 2021  *Viola flower syrup vs PEG* | Number of painful defecations per week, mean (SD)  VFS: 0,25 (1,01)  PEG: 0,40 (0,94) | Number of hard stools per week, mean (SD)  VFS: 0.53 (1.13) PEG: 0.56 (1.15) | NR | Number of fecal soiling per week, mean (SD) Viola flower syrup: 0,34 (1,27) PEG: 0,4 (1,25) | NR | NR | Incidence of unpleasant taste  VFS: 1/67 PEG: 2/66 |
| Herbal medicine vs placebo | | | | | | | |
| Cai 2018 *Xiao’er Biantong granules* | NR | Dry stool (1 and 2 of Bristol Stool Scale). Disappearance rate of dry stool, n(%).  XBG: 236/360 Placebo: 11/120 | NR | Disappearance rate of fecal incontinence, n(%).  XBG: 3/5 Placebo: 0/3 | NR | NR | NR |
| Qiao 2021 *XiaojiDaozhi Decoction* | Number of patients with painful or hard bowel movements  XiaojiDaozhi Decoction: 33/100  Placebo: 47/100 | Number of patients with painful or hard bowel movements  XiaojiDaozhi Decoction: 33/100  Placebo: 47/100 | NR | Number of patients with encopresis  XiaojiDaozhi Decoction: 6/100  Placebo: 15/100 | Unclear | NR | Number of patients lost to follow-up due to low compliance (no further specific reason)  XiaojiDaozhi Decoction: 6/100  Placebo: 5/100 |
| Manual physical therapy vs laxative | | | | | | | |
| Blanco Diaz 2020 | NR | Bristol Stool form Scale (modified 1-5 scale). Median (IQR)  Manual physical therapy: 4 (3-4)  PEG: 4 (3-4) | PedsQL questionnaire, scale of 0-100, higher scores indicate better QoL. Unclear if parent or child filled in questionnaire. Median (IQR).  Manual physical therapy: 89 (82 – 94)  PEG: 59 (50 -63) | NR | NR | NR | NR |
| Cow’s milk free diet vs cow’s milk diet | | | | | | | |
| Iacono 1998 | Categorized: (1) mushy/liquid, (2) soft, (3) hard and difficulty and pain on passing stools. Number of patients per group.  Not reported pre cross-over.  CMFD:  Group 1: n=2 Group 2: n=42 Group 3: n=21  CMD:  Group 1: n=0 Group 2: n=0 Group 3: n=65 | Categorized: (1) mushy/liquid, (2) soft, (3) hard and difficulty and pain on passing stools. Number of patients per group.  Not reported pre cross-over.  CMFD:  Group 1: n=2 Group 2: n=42 Group 3: n=21  CMD:  Group 1: n=0 Group 2: n=0 Group 3: n=65 | NR | NR | NR | NR | NR |
| Cow’s milk free diet + PEG vs Cow’s milk diet + PEG | | | | | | | |
| Bourkheili 2021 | Unclear data | Unclear data | NR | Unclear data | NR | NR | NR |
| Dehghani 2012 | Number of patients with painful or hard bowel movements  CMFD: 9/70  CMD: 32/70 | Number of patients with painful or hard bowel movements  CMFD: 9/70  CMD: 32/70 | NR | Number of patients with ≥ 1 episode/week  CMFD: 2/70  CMD: 15/70 | NR | NR | NR |
| Formula with partially hydrolyzed cow’s milk protein + prebiotics mix vs Formula based on cow’s milk + prebiotics mix | | | | | | | |
| Fabrizio 2022 | Participants who ever cried, fussed or appeared in pain while having or attempting to have a bowel movement No numbers reported | Parent-reported stool consistency was scaled as, mean (SE): hard, 1; formed, 2; mushy, 3; unformed or seedy, 4; watery. Formula 1: 3.4 (0.1) Formula 2: 3.0 (0.1) | NR | NR | NR | NR | NR |
| Fluid intake | | | | | | | |
| Young 1998 *Increased water intake vs hyperosmolar liquid vs control* | NR | Stool consistency on Stool Consistency Continuum (1= watery, 7/8 = hard), mean (no SD reported) Increased water: 5.79 Hyperosmolar: 6.3 Control: NR | NR | NR | NR | NR | NR |
| Parasacral transcutaneous electrical nerve stimulation (PTENS) vs sham therapy | | | | | | | |
| De Abreu 2021 | Number of patients with pain/straining during defecation after treatment PTENS: 0/20 Sham: 0/20 | Number of patients with stool type 1 or 2 on Bristol Stool Scale (hard) after treatment PTENS: 5/20 Sham: 10/20 | NR | Number of patients with episode of fecal incontinence after treatment PTENS: 2/20 Sham: 4/20 | NR | NR | NR |
| Abdominal transcutaneous electrical stimulation (TES) vs sham therapy | | | | | | | |
| Clarke 2009 | NR | NR | Parental and child perceived PedsQL (0-100), mean. no SD reported TES Parental: 70.1  Child: 81.1 Sham  Parental: 70.2  Child: 78.1 | NR | NR | NR | NR |
| Abdominal transcutaneous electrical stimulation (TES) as addition to pelvic floor muscle exercise (PFME) | | | | | | | |
| Ladi-Seyedian 2020 | Number of patients with painful defecation:  TES + PFME: 2/17 PFME: 6/17 | Number of patients with abnormal stool form:  TES + PFME: 3/17 PFME: 8/17 | NR | Number of patients with fecal soiling TES + PFME: 0/17 PFME: 1/17 | NR | NR | NR |
| Sharifi-Rad 2018 | Number of patients with painful or hard bowel movements TES + PFME: 6/45 PFME: 14/44 | Number of patients with painful or hard bowel movements TES + PFME: 6/45 PFME: 14/44 | Constipation-related QOL score, median (IQR). No scale reported. TES + PFME: 64 (5) PFME: 62 (6) | Number of patients with ≥1 episodes per week TES + PFME: 12/45 PFME: 27/44 | NR | NR | NR |
| Abdominal transcutaneous electrical stimulation (TES) and cryotherapy and standard therapy (ST) | | | | | | | |
| Khan 2020 | Number of patients with painful defecation:  TES + ST: 0/20 Cryotherapy + ST: 0/20 ST: 0/20 | NR | NR | Number of patients with fecal incontinence:  TES + ST: 4/20 Cryotherapy + ST: 2/20 ST: 6/20 | Number of patients with abdominal pain:  TES + ST: 4/20 Cryotherapy + ST: 2/20 ST: 5/20 | NR | NR |
| Percutaneous tibial nerve stimulation (PTNS) as addition to Pelvic Floor Exercises (PFE) | | | | | | | |
| Yu 2023 | Number of patients with painful or hard bowel movements PTNS + PFE: 33/42 PFE: 24/42 | Number of patients with painful or hard bowel movements PTNS + PFE: 33/42 PFE: 24/42 | NR | Number of patients with encopresis PTNS + PFE: 36/42  PFE: 23/42 | NR | NR | Number of patients withdrawn due to low compliance PTNS + PFE: 2/42 PFE: 2/42 |
| Self-monitoring and reward system to increase fiber intake vs standard dietary advice | | | | | | | |
| Sullivan 2012 | NR | NR | NR | NR | NR | NR | NR |
| Additional effect of behavioral therapy to laxatives | | | | | | | |
| Van Dijk 2008 | NR | NR | NR | Number of episodes per week, mean (95% CI) Behavioral + PEG: 8.6 (4.0–18.3) PEG: 6.4 (3.5–11.7) | NR | NR | NR |
| Biofeedback vs no biofeedback | | | | | | | |
| Castilla 2021 (abstract only) | NR | NR | NR | NR | NR | NR | NR |
| Additional effect of biofeedback to laxatives | | | | | | | |
| Loening-Baucke 1990  *Vs magnesiumhydroxide* | NR | NR | NR | Frequency per week, mean (SD) Biofeedback + MgOH: 1 (1) MgOH: 3 (6) | NR | NR | NR |
| Sunic-Omejc 2002 *vs Lactulose* | NR | NR | NR | NR | NR | NR | NR |
| Van der Plas 1996 *vs lactitol* | NR | NR | NR | NR | NR | NR | NR |
| Additonal effect of biofeedback at home to biofeedback in laboratory | | | | | | | |
| Croffie 2005 | NR | NR | NR | Number of soiling episodes per week, mean.  Unclear if SD or SE. Biofeedback home + laboratory: 0.08 (0.08) Laboratory feedback: 0.08 (0.08) | NR | NR | NR |
| Fiber vs placebo | | | | | | | |
| Chmielewska 2011 *Glucomannan* | Episodes per week, median (IQR). Fiber: 0 (0-1) Placebo: 0 (0) | BSFS (1-7), mean (SD) Fiber: 3.1 (1.1)  Placebo: 3.2 (1.0) | NR | NR | Episodes per week, median (IQR)  Fiber: 0 (0-2)  Placebo: 0 (0-1) | NR | 1 patient discontinued from fiber group due to "bad taste" |
| Loening-Bauke 2004  *Glucomannan* | NR | NR pre cross-over | NR | NR pre cross-over | NR pre cross-over | NR | NR |
| Weber 2014  *Fiber mixture* | - | BSFS (1-7) subgrouped as non-hardened (4-7)  Fiber: 12/27 Placebo: 4/30 | NR | NR | NR | NR | NR |
| Da Silva Souza  *Fructo-oligosaccharides* | % of bowel movements, mean (SD) Fiber: 14.68 ± 29 Placebo: 28.39 (43.82) | as % of BMs with soft stool consistency, mean (SD) Fiber: 73.38 (29.38)  Placebo: 55.38 (36.32) | NR | NR | NR | NR | All participants who completed the 4-week intervention (n = 36) consumed more than 80% of the delivered amount of FOS or placebo. |
| Fiber vs laxative | | | | | | | |
| Kokke 2008 *Fiber mixture vs lactulose* | NR | BSFS (1-7), mean, SD not reported.  Fiber: 3.6 Lactulose: 4.0 | NR | Number of patients with 1 or more fecal incontinence episodes per week. Fiber: 9/70  Lactulose: 5/70 | NR | NR | NR |
| Üstündağ 2010  *Partially hydrolysed guargum vs lactulose* | NR | NR | NR | NR | Number of patients with abdominal pain  Fiber: 5/35  Lactulose: 3/33 | NR | NR |
| Cassetari 2019  *Green banana biomass vs PEG and vs Sodium Picosulfate* | Number of patients who reported painful stools GBB: 4/15  PEG: 4/16  SP: 2/17 | Number of patients with BSFS higher than 1 or 2 (hard stools) GBB: 13/15  PEG: 11/16 SP: 13/17 | NR | NR | Number of patients with abdominal pain GBB: 5/15 PEG: 2/16  SP: 5/17 | NR | NR |
| Quitadamo 2012  *Fiber mixture vs PEG* | Number of children with painful defecation Fiber: 7/50 PEG: 4/50 | BSFS (1-7), mean (SD)  Fiber: 3.5 (0.2)  PEG: 3.7 (1.0) |  | Frequency of fecal incontinence per week, mean (SD) Fiber: 0.3 (1.1)  PEG: 0.2 (1.3) | Number of patients with abdominal pain  Fiber: 5/36  PEG: 6/47 | NR | NR |
| Formula with prebiotics and hydrolyzed whey protein vs standard formula | | | | | | | |
| Savino 2005 *Galactooligosaccharides and fructo-oligosaccharides* | NR | Number of patients with hard, formed or watery/runny stools  New formula: 38/55 hard, 14/55 formed, 3/55 runny  Standard formula: 23/40 hard, 13/40 formed, 4/40 runny | NR | NR | NR | NR | NR |
| Bongers 2007 *Galactooligosaccharides*  *and*  *fructo-oligosaccharides* | Number of patients who had no painful defecation  New formula: 7/20  Standard formula: 5/15 | Number of patients with improvement of hard to soft stools New formula: 9/10 Standard formula: 5/10 | NR | NR | NR | NR | NR |
| Synbiotics vs placebo | | | | | | | |
| Baştürk 2017 | Number of patients with painful defecation  Synbiotics: 16/77 Placebo: 27/78 | NAR | NR | NAR | Number of patients with abdominal pain  Synbiotics: 4/77  Placebo: 41/78 |  |  |
| Synbiotics and laxative | | | | | | | |
| Khodadad 2010 *Multispecies probiotics + fructo-oligosaccharides*  *Laxative: liquid paraffin* | Number of patients with painful defecations  Synbiotics: 3/31  Liquid paraffin: 2/29  Synbiotics+Liquid paraffin: 4/37 | Number of patients with hard stools  Synbiotics: 7/31 Liquid paraffin: 2/29  Synbiotics+Liquid paraffin: 4/37 | NR | Frequency per week, mean (SD)  Synbiotics: 0.06 (0.25) Liquid paraffin: 0.24 (1.3) Synbiotics+Liquid paraffin: 0 (0) | Number of patients with abdominal pain  Synbiotics: 2/31 Liquid paraffin: 4/29 Synbiotics+Liquid paraffin: 5/37 | NR | NR |
| Abdominal and acupressure point massage as addition to traditional Chinese medicine | | | | | | | |
| Mao 2015 | NR | NR | NR | NR | NR | NR | NR |
| Xu 2015 | NR | NR | NR | NR | NR | NR | NR |
| Foot reflexology massage as addition to toilet/diet/motivation training | | | | | | | |
| Canbulat Sahiner 2017 | NR | Number of patients with normal or soft stools Foot reflexology massage + standard therapy: 15/20  Standard therapy: 18/20 | NR | NR | NR | NR | Compliance was assessed for toilet/diet/motivation training per week as yes/no for compliance.  No difference for toilet training and compliance to motivation.  Control group followed the diet more closely  and the difference was statistically significant from the  second week. |
| Pelvic floor physiotherapy as standard medical care | | | | | | | |
| Van Engelenburg 2017  *Standard medical care: education, dietary advice, toilet training, PEG* | Painful/hard stools: number of patients with improvement from baseline  Physio: 15/15 Physio + standard medical care: 10/17 | Painful/hard stools: number of patients with improvement from baseline  Physio: 15/15 Physio + standard medical care:: 10/17 | NR | Fecal incontinence: number of patients with improvement from baseline  Physio: 13/15 Physio + standard medical care:: 10/15 | NR | NR | NR |
| Abdominal muscle training/breathing exercises/abdominal massage as addition to laxatives | | | | | | | |
| Silva 2013  *Laxative: Magnesium Hydroxide (MgO)* | NR | NR | NR | NR | NR | NR | NR |
| Dry cupping vs laxative | | | | | | | |
| Shahamat 2016  *Laxative: PEG* | Number of patients with painful or hard bowel movements  Dry cupping: 7/60 PEG: 10/60 | Number of patients with painful or hard bowel movements  Dry cupping: 7/60 PEG: 10/60 | NR | Not adequately reported | NR | NR | NR |
| Uniformed Services Constipation Action Plan vs standard care | | | | | | | |
| Reeves 2022 | NR | Number of patients with BFSF score of 3 or 4  USCAP: 44/57 Standard care: 43/73 | Measured with PedsQL Gastrointestinal Symptoms (PedsGIQL), scale of 0-100. Higher score: better gastrointestinal-specific Quality of Life, mean (SD) USCAP: 72.5 (16.7)  Standard Care: 69.2 (17.9) | Number of patients with Fecal Incontinence reduction USCAP: 18/57 Standard Care: 15/73 | NR | NR | Number of patients who reported complete 4 months adherence to pharmacotherapy. Only reported for patients reaching study end.  IG: 33/57  CG: 21/73 |

**Supplementary Table 7. Details of side effects of included pharmacological studies**

*Disimpaction*

| Study | Intervention | Control |
| --- | --- | --- |
| Enema vs PEG (oral) | | |
| Bekkali 2009 | Only reported abdominal pain soon after treatment: 31/38 | Only reported abdominal pain soon after treatment: 15/31 |

*Maintenance therapy*

| Study | Intervention | Control |
| --- | --- | --- |
| PEG vs Placebo | | |
| Modin 2018 | SAE  None  AE (only reported for gastrointestinal symptoms)  The reported adverse events possibly related to study drug were: abdominal pain N=6 and bloating N=1  Total number of children with AE related to gastrointestinal tract: 28% | SAE  None  AE (only reported for gastrointestinal symptoms)  The reported adverse events possibly related to study drug were: abdominal pain N=22  and bloating N=0  Total number of children with AE related to gastrointestinal tract: 69% |
| Nurko 2008 | SAE  Dose 0.2 g/kg: hospitalization due to impaction N=2  AE  Dose 0.4 g/kg: number of children with an AE = 16/27 (59.3%) Dose 0.8 g/kg: number of children with an AE = 17/26 (65.4%)  Type of AE not reported per patient | SAE  Hospitalization due to exacerbation of bipolar disorder and depression N=1  AE  Number of children with an AE = 14/24 (58.3%) Type of AE not reported per patient |
| Thomson 2007 | NR pre cross-over.  During complete trial: 31/49 (63.3%) | NR pre cross-over  During complete trial: 28/49 (57.1%) |
| PEG vs Lactulose | | |
| Dheivamani 2021 | SAE  NR  AE  Number of children with an AE: 1/50  Fever N=1 | SAE  NR  AE  None |
| Dupont 2005 | SAE  None  AE  Number of children with an AE: 2/51  Diarrhea N=2 | SAE  None  AE  Number of children with an AE: 3/45  Diarrhea N=2, anorexia N=1 |
| Jarzebicka 2019 | SAE  None  AE  Number of children with an AE: 27/46  Prevalence of AE’s: abdominal pain N=10, Diarrhea N=6, Nausea/vomiting N=1, Bloating/gas N=20, Irritation of the anal area N=2 | SAE  None  AE  Number of children with an AE: 38/49  Prevalence of AE’s: abdominal pain N=17, Diarrhea N=3, Nausea/vomiting N=1, Bloating/gas N=35, Irritation of the anal area N=9 |
| Saneian 2012  *PEG vs Lactulose vs Magnesium hydroxide (MgOH)* | SAE  NR  AE  Number of children with an AE: NR  Number of children with specific AE: Bloated N=2, stomach irritation N=1, abdominal pain N=1 | SAE  NR  AE  Number of children with an AE: NR  Number of children with specific AE (Lactulose): Nausea and vomiting N=1, Bloated N=17, diarrhea N=1, Stomach irritation N=2, abdominal pain N=14  Number of children with specific AE (MgOH): Bloated N=1, diarrhea N=5, Stomach irritation N=1, abdominal pain N=17 |
| Treepongkaruna 2014 | SAE  Pneumonia N=1, Road traffic accident N=1  AE  Number of children with an AE: 27/44  Anal dilatation N=14, Upper respiratory tract infections N=9, Anal fissure N=9, Faecaloma N=5,  Hard faeces N=3, Anal skin tags N=5, Rhinorrhoea N=3, Vomiting N=3 | SAE  Varicella infection N=1  AE  Number of children with an AE: 26/44  Anal dilatation N=10, Upper respiratory tract infections N=9, Anal fissure N=6, Faecaloma N=7, Hard faeces N=4, Anal skin tags N=1, Rhinorrhoea N=1 |
| Uhm 2007 | SAE  None  AE  Number of children with an AE: 2/24  Nausea N=1, Diarrhea N=1 | SAE  None  AE  Number of children with an AE: 5/32  Nausea N=1, Diarrhea N=2, Abdominal discomfort N=2 |
| Voskuijl 2004 | SAE  None  AE  NR | SAE  None  AE  NR |
| Wang 2007 | SAE  None  AE  NR | SAE  None  AE  NR |
| PEG vs Magnesium hydroxide | | |
| Gomes 2011 | SAE  NR  AE  NR | SAE  NR  AE  NR |
| Loening-Baucke 2006 | SAE  None  AE  NAR. Allergic N=1 Transient diarrhea which disappeared with dose reduction was reported. Not reported per group. | SAE  None  AE  NAR  Transient diarrhea which disappeared with dose reduction was reported. Not reported per group. |
| Ratanamongkol 2009 | SAE  None  AE  Number of children with an AE: 20/46  Number of children with specific AE: Diarrhoea N=2, Abdominal pain N=9, bloating N=13, nausea N=4 | SAE  None  AE  Number of children with an AE: 24/43  Number of children with specific AE: Diarrhoea N=12, abdominal pain N=14, Bloating N=13, Nausea N=9 |
| PEG vs fiber | | |
| Quitadamo 2012  *Fiber mixture* | SAE  NR  AE  None | SAE  NR  AE  None |
| PEG 4000 vs PEG3350 + electrolytes | | |
| Bekkali 2018 | SAE  Number of children with a SAE: 0/49  AE  Number of children with an AE: 28/49  Number of children with specific AE: Abdominal pain N=3, Diarrhoea N=1, Mouth ulceration N=1, Nausea N=2, Vomiting N=4, Influenza like illness N=1, Pyrexia N=9, Ear infection N=1, Gastroeneritis N=2, Gastroenterisitis viral N=3, Influenza N=2, Nasopharyngitis N=5, Respiratory tract infection N=2, Upper respiratory tract infection N=3, Urinary tract infection N=1, Varicella N=1, Viral infection N=1, Headache N=3, Polyuria N=1, Oropharyngeal pain N=2, Eczema N=1 | SAE  Number of children with a SAE: 2/48  Prevalence of AE’s: Dehydration N=2, Upper respiratory infection N=1, Metabolic acidosis N=1, Constipation N=1  AE  Number of children with an AE: 28/48  Number of children with specific AE: Diarrhoea N=1, Nausea N=3, Toothache N=1, Vomiting N=2, Influenza like illness N=1, Pyrexia N=4, Bronchitis N=2, Ear infection N=1, Eye infection N=1, Gastroenteritis N=5, Gastroenteritis viral N=3, Influenza N=1, Nasopharyngitis N=4, Pseudocroup N=2, Upper respiratory tract infection N=1, Urinary tract infectrion N=1, Varicella N=3, Viral infection N=1, Dehydration N=1, Headache N=1, Polyuria N=1, Cough N=1, Eczema N=1 |
| Savino 2012 | SAE  NR  AE  Number of children with an AE: 1/50  Diarrhoea and vomiting N=1 | SAE  NR  AE  Number of children with an AE: 1/46  Abdominal pain N=1 |
| PEG vs herbal medicine | | |
| Dehghani 2019  *Black strap molasses (BSM) (sugarcane extract)* | SAE  NR  AE  Number of children with an AE: 7/47  Abdominal pain N=7 | SAE  NR  AE  Number of children with an AE: 4/45  Abdominal pain N=4 |
| Esmaeilidooki 2016  *Cassia’s fistula emulsion* | SAE  NR  AE  Number of children with specific AE: Diarrhoea N=15/57, Abdominal pain N=5/57 | SAE  NR  AE  Number of children with specific AE: Diarrhoea N=13/52, Abdominal pain 2/52 |
| Imanieh 2022  *R. damascena and brown sugar syrup* | NAR | NAR |
| Nasri 2022  *LaxaPlus Barij®* | NR | NR |
| Nimrouzi 2015  *D. Sohpia seed* | NR  10 patients received bisacodyl suppositories after seven days of no bowel movement. Unlcear if these cases were new onset. | NR  3 patients received bisacodyl suppositories after seven days of no bowel movement. Unlcear if these cases were new onset. |
| Saneian 2021  *Goleghand®* | NR | NR |
| Tavassoli 2021  *Viola flower syrup* | SAE  NR  AE  Number of patients with specific AE: Abdominal pain N=6, Loose stools N=2, Nausea N=2, Vomiting N=1, Unpleasant taste N=2 | SAE  NR  AE  Number of patients with specific AE: Abdominal pain N=1, Unpleasant taste N=1 |
| PEG vs liquid paraffin | | |
| Karami 2009 | NAR | NAR |
| Rafati 2011 | NR | NR |
| PEG vs microenema | | |
| Strisciuglio 2021  *Enema: Promelaxin* | SAE  Number of children with an SAE: 0/77  AE  Number of events reported by patients: N=107 | SAE  Number of children with an SAE: 2/76 Type of SAE not reported  AE  Number of events reported by patients: N=76 |
| Enema as addition to PEG | | |
| Bongers 2009  *Enema: sodium-dioctyl*  *sulfosuccinate and sorbitol* | NR | NR |
| PEG vs fiber vs probiotics | | |
| Foroughi 2022 *fiber: psyllium*  *Probiotics: L.reuteri, L.*  *rhamnosus, and Bifidobacterium infantis* | NR | NR |
| PEG vs sodium picosulfate (SP) vs fiber | | |
| Cassettari 2019 *Dietary: green banana biomass (GBB)* | SAE  PEG: none  PEG + GBB: none  AE  PEG: none  PEG + GBB: none | SAE  SP: none  GBB: none  SP + GBB: none  AE  SP: none  GBB: none  SP + GBB: none |
| PEG vs dry cupping | | |
| Shahamat 2016 | NR | NR |
| Lactulose vs placebo | | |
| Cao 2018 | SAE  0/50  AE  Number of children with specific AE: anal dilatation N=11/50, upper respiratory tract infections N=8/50, fecaloma N=9/50, anal fissure N=7/50, hard feces N=4/50, rhinorrhea N=1/50 | SAE  0/50  AE Number of children with specific AE: anal dilatation N=8/50, upper respiratory tract infections N=6/50, fecaloma N=6/50, anal fissure N=5/50, hard feces N=2/50, rhinorrhea N=2/50 |
| Lactulose vs dietary | | |
| Kokke 2008  *Fiber mixture* | SAE  0/70  AE  Number of children with an AE: 2/70  Persistent diarrhea N=2 | SAE  0/65  AE  Number of children with an AE: 1/65  Persistent diarrhea N=1 |
| Ustundag 2010  *Partially hydrolysed guargum (PHG)* | NR | NR |
| Lactulose vs liquid paraffin | | |
| Farahmand 2007 | SAE  0/120  AE  NAR (only data in figure, no numbers)  “Significantly more adverse events were reported by patients using lactulose compared with patients on liquid paraffin.” | SAE  0/127  AE  NAR (only data in figure, no numbers) |
| Urganci 2005 | SAE  NR  AE  NAR (adverse events only reported in context of how it influenced compliance, not as outcome) | SAE  NR  AE  NAR (adverse events only reported in context of how it influenced compliance, not as outcome) |
| Lactulose vs lactitol | | |
| Pitzalis 1995 | NR | NR |
| Lactulose vs probiotics | | |
| Lee 2022  *S. boulardii* | SAE  NR  AE  NAR (not reported per group) | SAE  NR  AE  NAR (not reported per group) |
| Olgac 2013  *L. reuteri* | SAE  NR  AE  NAR (not reported per group) | SAE  NR  AE  NAR (not reported per group) |
| Lactulose as addition to PEG | | |
| Ala 2015 | SAE  NR  AE  Number of children with an AE: 15/100  “Adverse effects such as abdominal pain, diarrhea, and flatulence”. Not reported how many per adverse event. | SAE  NR  AE  Number of children with an AE: 0/100 |
| Magnesiumoxide vs probiotics | | |
| Bu 2007  Compares MgO vs probiotics vs placebo  *Probiotics: L. rhamnosus lcr35* | SAE  NR  AE  Number of children with an AE: 1/18  Mild diarrhea N=1 | SAE  NR  AE (Probiotics)  Number of children with an AE: 0/18  AE (Placebo)  Number of children with an AE: 0/9 |
| Kubota 2020  *Compares MgO vs probiotics vs MgO + probiotics*  *Probiotics: L. reuteri* | SAE  NR  AE  NAR (only reported that no AE’s related to any  treatment was observed) | SAE  NR  AE  NAR (only reported that no AE’s related to any  treatment was observed) |
| Liquid paraffin vs herbal medicine | | |
| Mozaffarpur 2012  *Cassia fistula emulsion* | SAE  0/40  AE  Number of children with a specific AE: Anal oily leakage N=27/40, abdominal pain N=3/40, extra saliva N=2/41, headache N=1/40, drug intolerance accompanied with upper respiratory infection N=1 | SAE  0/41  AE Number of children with a specific AE: Diarrhea N=12/40, abdominal pain N=3/41, sputum-like stool N=1/41 |
| Liquid paraffin vs synbiotics | | |
| Khodadad 2010 *Multispecies probiotics + fructo-oligosaccharides* | NR | NR |
| Lubiprostone vs placebo | | |
| Benninga 2022 | SAE  11/400  Hepatotoxicity N=1, Anaphylactoid reaction N=1, Decreased consciousness N=1, Dehydration and IBS-C N=1, Fecaloma and rash N=1, Fecaloma N=2, Ulcerative colitis N=1, Suicidal ideation N=2, Coxsackie virus N=1  AE  Number of children with an AE: 239/400  AE’s occurring in >5% of children: headache N=34/400, Nausea N=257/400, Vomiting N=45/400, Abdominal pain N=42/400 | SAE  7/195  NR  AE  Number of children with an AE: 114/195  AE’s occurring in >5% of children: headache N=10/195, nausea N=14/195, vomiting N=12/195, abdominal pain N=23/195 |
| Lubiprostone vs conventional therapy | | |
| Elkargy 2024 | AE  62/135  Colicky abdominal pain and headaches were the most prevalent side effects in the current study, followed by nausea  SAE  0/135 | AE  65/136  Colicky abdominal pain and headaches were the most prevalent side effects in the current study, followed by nausea  SAE  0/136 |
| Prucalopride vs placebo | | |
| Mugie 2014 | SAE  Number of children with a SAE: 5/106  Number of children with specific SAE: abdominal pain N=1, vomiting N=, diarrhea N=1, Nausea N=1, Appendicitis N=1, Pneumonia N=1, Dizziness N=1, Syncope N=1, Anxiety N=1  AE  Number of children with an AE: 101/106  Number of children with specific AE: Headache N=17, Pyrexia N=15, Abdominal pain N=14, Vomiting N=15, Nausea N=10, Viral infection N=6, Cough N=6, Diarrhea N=6, Nasopharyngitis N=3, Pharyngitis N=3, Bronchitis N=2, Upper respiratory tract infection N=2, Constipation N=2 | SAE  Number of children with a SAE:2/107  Number of children with specific SAE: Abdominal pain N=1, Constipation N=1, Anorectal discomfort N=1  AE  Number of children with an AE: 72/107  Number of children with specific AE: Headache N=9, Pyrexia N=3, Abdominal pain N=13, Vomiting N=5  Nausea N=6, Viral infection N=5, Cough N=2  Diarrhea N=6, Nasopharyngitis N=2, Pharyngitis N=6  Bronchitis N=7, Upper respiratory tract infection N=5, Constipation N=3 |
| Linaclotide vs placebo | | |
| Di Lorenzo 2020 | SAE  Number of children with a SAE: 0/39  AE  Number of children with an AE: 15/39 Not reported for all AE’s. Diarrhea N=4, Headache N=4, fecaloma N=2 | SAE  Number of children with a SAE: 0/41  AE  Number of children with an AE: 9/41 Not reported for all AE’s. Headache N=1, vomiting N=1, alanine aminotransferase increased N=1, aspartate aminotransferase increased N=1 |
| Di Lorenzo 2024 | SAE  Number of children with a SAE: 2/164  Diarrhea N=1, Faecaloma N=1  AE  Number of children with an AE: 28/164  Number of children with a Treatment Related AE: diarrhea N=6, Nausea N=2, Abdominal discomfort N=1, COVID-19 N=1, dehydration N=1 | SAE  Number of children with a SAE: 2/164  Suicide attempt N=2, suicidal ideation N=1  AE  Number of children with an AE: 35/164  Number of children with a Treatment related AE: diarrhea N=2, abdominal distention N=1, dizziness N=1, headache N=1 |
| Di Lorenzo 2024b | SAE  Number of children with a SAE: 0/13  AE  Number of children with an AE: 2/13 Number of children with specific AE: upper abdominal pain (N=1), diarrhea (N=1), ear infection (N=1) | SAE  Number of children with specific AE: 0/27  AE  Number of children with an AE: 1/8 (developmental delay N=1) |
| Domperidone as addition to PEG | | |
| Dehghani 2014 | SAE  NR  AE  Number of children with an AE: 0/52 | SAE  NR  AE  Number of children with an AE: 0/52 |

**Supplementary Table 8. Details of side effects of included non-pharmacological studies**

| Study | Intervention | Control |
| --- | --- | --- |
| Probiotics vs placebo | | |
| Lojanatorn 2023 | SAE  0/21  AE  Number of children with an AE: 3/21 Urticaria N=1, abdominal pain N=1, dropout due to severe abdominal N=1 | SAE  0/18  AE  Number of children with an AE: 1/18 Vomiting N=1 |
| Tabbers 2011 | SAE  Not reported per group: 2 SAE’s, probably not related to consumption of the study drugs occurred Broken arm N=1, gynecological pain caused by a gynecological cyst N=1  AE  Number of children with an AE: 4/79 Gastroenteritis N=1, nausea/vomiting N=3 | SAE  Not reported per group: 2 SAE’s, probably not related to consumption of the study drugs occurred Broken arm N=1, gynecological pain caused by a gynecological cyst N=1  AE  Number of children with an AE: 6/80 Gastroenteritis N=3, nausea/vomiting N=2, candida-infection of anorectal region N=1 |
| Tjokronegoro 2020 | SAE  0/39  AE  Number of children with an AE: 2/39 Mild abdominal pain N=2 | SAE  0/39  AE  Number of children with an AE: 6/39 Mild abdominal pain N=4, mild diarrhea N=2 |
| Zaja 2021 | SAE  0/15  AE  Number of children with an AE: 0/15 | SAE  0/16  AE  Number of children with an AE: 0/16 |
| Bu 2007 *L. rhamnosus lcr35 vs placebo vs MgO* | NR | NR |
| Wojtyniak 2017 | SAE  0/48  AE  Number of children with an AE: 0/48 | SAE  0/46  AE  Number of children with an AE: 3/46 Change in stool odor N=1, abdominal pain and flatulence N=1, loss of appetite N=1 |
| Gan 2022 | NR | NR |
| Coccorullo 2010 | NR | NR |
| Probiotics vs laxative | | |
| Kubota 2020 *L. reuteri vs MgO* | SAE  NR  AE  Number of children with an AE: 0/20 | SAE  NR  AE  Number of children with an AE: 0/21 |
| Lee 2022 *S. boulardii vs Lactulose* | SAE  NR  AE Number of children with an AE: NR per group and per patient. Abdominal pain = 20.9%, 11.3%, and 1.8% at weeks 2, 6, and 12, respectively. Diarrhea = 6.3% and 4.7% at weeks 2 and 6, respectively. Abdominal distension = 4.4% at week 2, and  Vomiting = 1.3% at week 2).  There were no intergroup differences. | SAE  NR  AE Number of children with an AE: NR per group and per patient. Abdominal pain = 20.9%, 11.3%, and 1.8% at weeks 2, 6, and 12, respectively. Diarrhea = 6.3% and 4.7% at weeks 2 and 6, respectively. Abdominal distension = 4.4% at week 2, and  Vomiting = 1.3% at week 2).  There were no intergroup differences. |
| Olgaç 2013 *L. reuteri vs lactulose* | SAE  NR  AE Number of children with an AE: 0/25 | SAE  NR  AE Number of children with an AE: 0/28 |
| Probiotics as addition to laxatives | | |
| Abediny 2016 *Multispecies and PEG* | NR | NR |
| Banaszkiewicz 2005 *Lactobacillus GG and lactulose* | SAE  NR  AE  Number of patients with an AE: 4/43 Abdominal pain N=3, vomiting N=1 | SAE  NR  AE  Number of patients with an AE: 6/41  5 abdominal pain, 1 head ache |
| Jadrešin 2018 *L. reuteri and lactulose* | SAE  NR  AE  Number of patients with an AE: 0/18 | SAE  NR  AE  Number of patients with an AE: 0/18 |
| Foroughi 2022 *Laxative: PEG* *Fiber: psyllium*  *Probiotics: L.reuteri, L.*  *rhamnosus, and Bifidobacterium infantis* | NR | NR |
| Kubota 2020  *Multispecies and Magnesiumoxide* | NR | NR |
| Russo 2017  *Multispecies and PEG* | No data reported  No significant clinical adverse effects, except for transient diarrhea, which disappeared with dose reduction | No data reported  No significant clinical adverse effects, except for transient diarrhea, which disappeared with dose reduction |
| Sadeghzadeh 2014  *Multi species and lactulose* | SAE  NR  AE  Number of patients with an AE: 0/28 | SAE  NR  AE  Number of patients with an AE: 0/28 |
| Wegner 2018 *L. reuteri + PEG* | SAE  0/65  AE  Number of patients with an AE: 2/65 Abdominal pain N=2 | SAE  0/64  AE  Number of patients with an AE: 2/65 |
| Probiotics as addition to goat yoghurt | | |
| Guerra 2011 | NR | NR |
| Formula 1 intact protein + PEG vs Formula 2 hydrolyzed whey + PEG | | |
| Sevilla 2022 | SAE  Number of children with an AE: 0/47  AE  Number of patients with an AE: 0/47 | SAE  Number of children with an AE: 0/48  AE  Number of patients with an AE: 0/48 |
| Herbal medicine vs laxative | | |
| Dehghani 2019 *Black strap molasses (sugarcane extract) vs PEG* | SAE  NR  AE  Number of patients with an AE: 4/45 Abdominal pain N=4 | SAE  NR  AE  Number of patients with an AE: 7/47 Abdominal pain N=7 |
| Esmaeilidooki 2016  *Cassia’s fistula emulsion vs PEG* | SAE  NR  AE  Number of patients with an AE: 15/52 Diarrhoea N=13, abdominal pain N=2 | SAE  NR  AE  Number of patients with an AE: 20/66 Diarrhoea N=15, abdominal pain N=5 |
| Imanieh 2022  *R. damascena and brown sugar syrup vs PEG* | SAE  NR  AE  Number of patients with an AE: 0/50 | SAE  NR  AE  NR |
| Mozaffarpur 2012  *Cassia fistula emulsion vs Liquid Paraffin* | NR | NR |
| Nasri 2022  *LaxaPlus Barij® vs PEG* | NR | NR |
| Nimrouzi 2015  *D. Sohpia seed vs PEG* | NR | NR |
| Saneian 2021  *Goleghand® vs PEG* | NR | NR |
| Tavassoli 2021  *Viola flower syrup vs PEG* | SAE  NR  AE  Number of patients with an AE: 1/67 Abdominal pain N=1 | SAE  NR  AE  Number of patients with an AE: 12/66 6 abdominal pain N=6, loose stools N=2, nausea N=2, vomiting N=1 |
| Herbal medicine vs placebo | | |
| Cai 2018 | SAE  NR  AE  Number of patients with an AE: 7/360  Type of adverse events not reported for all adverse events and for which group. Only ones reported were: loose stool N=1, diarrhea N= 3, and vomit N=1 | SAE  NR  AE  Number of patients with an AE: 2/120 Type of adverse events not reported for all adverse events and for which group. Only ones reported were: loose stool N=1, diarrhea N= 3, and vomit N=1 |
| Qiao 2021 | SAE  0/100  AE  Number of patients with an AE: 11/100 Vomiting N=5, abdominal distention N=4, mild diarrhea N=2  Cold and fever were found in 6 patients, however unclear to which group these patients belong. | SAE  0/100  AE  Number of patients with an AE: 3/100  Vomiting N=3  Cold and fever were found in 6 patients, however unclear to which group these patients belong. |
| Manual physical therapy vs laxative | | |
| Blanco Diaz 2020  Laxative: PEG | NR | NR |
| Cow’s milk free diet vs cow’s milk diet | | |
| Iacono 1998 | NR | NR |
| Cow’s milk free diet + PEG vs Cow’s milk diet + PEG | | |
| Bourkheili 2021 | NR | NR |
| Dehghani 2012 | NAR | NAR |
| Formula 1 (partially hydrolyzed cow’s milk protein) + prebiotics mix vs Formula 2 (cow’s milk-based) vs prebiotics mix | | |
| Fabrizio 2022 | SAE  NR  AE  Number of patients with an AE: 14/42 No reasons provided | SAE  NR  AE  Number of patients with an AE: 8/47 No reasons provided |
| Increased water intake vs hyperosmolar liquid vs control | | |
| Young 1998 | NR | NR |
| Parasacral transcutaneous nerve stimulation (PTENS) vs sham therapy | | |
| De Abreu 2021 | NR | NR |
| Abdominal transcutaneous electrical stimulation vs sham therapy | | |
| Clarke 2009 | NR | NR |
| Abdominal transcutaneous electrical stimulation as addition to pelvic floor muscle exercise | | |
| Sharifi-Rad 2018 | SAE  NR  AE  Number of patients with an AE: 0/45 | SAE  NR  AE  Number of patients with an AE: 0/45 |
| Lady-Seyedian 2020 | SAE  NR  AE  Number of patients with an AE: 0/17 | SAE  NR  AE  Number of patients with an AE: 0/17 |
| Abdominal transcutaneous electrical stimulation and cryotherapy and standard therapy | | |
| Khan 2020 | NR | NR |
| Tibial nerve stimulation as addition to Pelvis Floor Exercises | | |
| Yu 2023 | SAE  0/42  AE  Number of patients with an AE: 3/42 Skin allergies + erythema + blisters N=1 and foot numbness N=2 | SAE  0/42  AE  Number of patients with an AE: 4/42 Skin allergies + erythema + blisters N=2 and foot numbness N=2 |
| Self-monitoring and reward system to increase fiber intake vs standard dietary advice | | |
| Sullivan 2012 | NR | NR |
| Additional effect of behavioral therapy to laxatives | | |
| Van Dijk 2008 | NR | NR |
| Biofeedback vs no biofeedback | | |
| Castilla 2021 (abstract only) | NR | NR |
| Additional effect of biofeedback to laxatives | | |
| Loening-Baucke 1990  *Vs magnesiumhydroxide* | NR | NR |
| Sunic-Omejc 2002 *vs Lactulose* | NR | NR |
| Van der Plas 1996 *vs lactitol* | NR | NR |
| Additional effect of biofeedback at home to biofeedback in laboratory setting | | |
| Croffie 2005 | NR | NR |
| Fiber vs placebo | | |
| Chmielewska 2011 *Glucomannan* | SAE  1/40 Rotavirus acute gastroenteritis requiring hospital admission for intravenous rehydration N=1.  AE  Number of patients with an AE: NR Total of adverse events: Gastroenteritis N=1, Vomiting N=1, Bronchitis N=2, Otitis media N=1 | SAE  0/40  AE  Number of patients with an AE: NR Total of adverse events: Gastroenteritis N=2, Vomiting N=1, Bronchitis N=1, Pruritus N=1 |
| Loening-Bauke 2004  *Glucomannan* | SAE  0/27  AE  Number of patients with an AE: 0/27 | SAE  0/19  AE  Number of patients with an AE: 0/19 |
| Weber 2014  *Fiber mixture* | SAE  0/27  AE  NR | SAE  0/30  AE  NR |
| Da Silva Souza 2018 | SAE  NR  AE  Number of children with an AE: 4/19 Pneumonia N=1, abdominal distention and flatulence N=2, vomiting N=1 | SAE  NR  AE  Number of children with an AE: 0/19 |
| Fiber vs laxatives | | |
| Kokke 2008 *Fiber mixture vs lactulose* | SAE  0/65  AE  Number of children with an AE: 1/65  Persistent diarrhea N=1 | SAE  0/70  AE  Number of children with an AE: 2/70 Persistent diarrhea N=2 |
| Üstündağ 2010  *Partially hydrolysed guargum vs lactulose* | NR | NR |
| Cassetari 2019  *Green banana biomass vs PEG and vs Sodium Picosulfate* | SAE  GBB: 0/15  AE  GBB: 0/15 | SAE  PEG: 0/16  SP: 0/17  AE  PEG: 0/16  SP: 0/17 |
| Quitadamo 2012  *Fiber mixture vs PEG* | SAE  NR  AE  0/36 | SAE  NR  AE  0/47 |
| Formula with prebiotic and hydrolyzed whey protein vs standard formula | | |
| Savino 2005 *Galactooligosaccharides*  *and*  *fructo-oligosaccharides* | SAE  0/69  AE  Number of children with an AE: 0/69 | SAE  0/54  AE  Number of children with an AE: 0/54 |
| Bongers 2007 *Galactooligosaccharides*  *and*  *fructo-oligosaccharides* | SAE  0/20  AE  Number of children with an AE: 0/20 | SAE  0/15  AE  Number of children with an AE: 0/15 |
| Synbiotics vs placebo | | |
| Baştürk 2017 | SAE  NR  AE  Number of children with an AE: 0/77 | SAE  NR  AE  Number of children with an AE: 0/78 |
| Synbiotics vs laxative | | |
| Khodadad 2010 *Multispecies probiotics + fructo-oligosaccharides*  *Laxative: liquid paraffin* | NR | NR |
| Abdominal and acupressure point massage as addition to traditional Chinese medicine | | |
| Mao 2015 | NR | NR |
| Xu 2015 | NR | NR |
| Foot reflexology massage as addition to toilet/diet/motivation training | | |
| Canbulat Sahiner 2017 | NR | NR |
| Pelvic physiotherapy as addition to standard medical care vs standard medical care | | |
| Van Engelenburg 2017  *Standard medical care: education, dietary advice, toilet training, PEG* | NR | NR |
| Abdominal muscle training/breathing exercises/abdominal massage as addition to laxatives | | |
| Silva 2013  *Laxative: Magnesium Hydroxide (MgO)* | NR | NR |
| Dry cupping vs laxative | | |
| Shahamat 2016  *Laxative: PEG* | NR | NR |
| Uniformed Services Constipation Action Plan vs standard care | | |
| Reeves 2022 | NR | NR |

**Supplementary Table 9. Voting results**

| **Treatment option** | **Voting results on statements** |
| --- | --- |
| *Pharmacological treatment: Disimpaction* | |
| Part 1 of GPS | 15/15 |
| Part 2 of GPS | 14/14 |
| Part 3 of GPS | 14/14 |
| *Pharmacological and non-pharmacological maintenance treatment* | |
| PEG | 12/12 |
| Lactulose | 14/14 |
| Magnesium (hydr)oxide | 15/15 |
| Bisacodyl | 15/15 |
| Senna | 15/15 |
| Sodium picosulfate | 15/15 |
| Liquid paraffin | 15/15 |
| Prucalopride | 13/13 |
| Lubiprostone | 13/13 |
| Linaclotide | 12/12 |
| Enema (GRADE) | 14/14 |
| Enema (GPS) | 14/14 |
| Domperidone | 15/15 |
| Botox | 13/15 |
| Suppositories | 15/15 |
| Transanal irrigation | 15/15 |
| Probiotics (GRADE) | 15/15 |
| Probiotics (GPS) | 15/15 |
| Prebiotics | Consensus reached to included in generic statement, no voting procedure |
| Fiber | Consensus reached to included in generic statement, no voting procedure |
| Fluid | Consensus reached to included in generic statement, no voting procedure |
| Synbiotics (GRADE) | 12/15 |
| Synbiotics (GPS) | 15/15 |
| Herbal medicine | No voting procedure* |
| Parasacral transcutaneous electrical nerve stimulation | Vote 1: 9/15, vote 2: 11/15 --> no consensus reached |
| Abdominal transcutaneous electrical stimulation | 15/15 |
| Percutaneous tibial nerve stimulation | 15/15 |
| Cow’s milk free diet | 15/15 |
| Behavioral interventions | 15/15 |
| Pelvic floor physiotherapy | 12/14 |
| Biofeedback | 13/13 |
| Cryotherapy | No voting procedure* |
| Massage | No voting procedure* |
| Dry cupping | No voting procedure* |
| The Uniformed Services Constipation Action Plan | No voting procedure* |
| Physical activity | Consensus reached to included in generic statement, no voting procedure |
| Toilet training | Consensus reached to included in generic statement, no voting procedure |
| *Surgery* | |
| Surgery | 14/14 |

*The GDG decided not to cast votes for these treatment options as they were considered to be out of scope or because availability of the treatment was uncertain.

1	Bekkali NL, van den Berg MM, Dijkgraaf MG, et al. Rectal fecal impaction treatment in childhood constipation: enemas versus high doses oral PEG. Pediatrics 2009;124(6):e1108-15.

2	Dheivamani N, Thomas W, Bannerjii R, et al. Efficacy of polyethylene glycol 3350 as compared to lactulose in treatment of ROME IV criteria-defined pediatric functional constipation: A randomized controlled trial. Indian J Gastroenterol 2021;40(2):227-33.

3	Dupont C, Leluyer B, Maamri N, et al. Double-blind randomized evaluation of clinical and biological tolerance of polyethylene glycol 4000 versus lactulose in constipated children. J Pediatr Gastroenterol Nutr 2005;41(5):625-33.

4	Jarzebicka D, Sieczkowska-Golub J, Kierkus J, et al. PEG 3350 Versus Lactulose for Treatment of Functional Constipation in Children: Randomized Study. J Pediatr Gastroenterol Nutr 2019;68(3):318-24.

5	Saneian H, Mostofizadeh N Comparing the efficacy of polyethylene glycol (PEG), magnesium hydroxide and lactulose in treatment of functional constipation in children. Journal of Research in Medical Sciences 2012;17(145-9.

6	Treepongkaruna S, Simakachorn N, Pienvichit P, et al. A randomised, double-blind study of polyethylene glycol 4000 and lactulose in the treatment of constipation in children. BMC Pediatr 2014;14(153.

7	Uhm JH Comparison of polyethylene glycol 4000 and lactulose for treatment of chronic functional constipation in children. Korean Journal of Pediatrics 2007;50(8):752-56.

8	Voskuijl W, de Lorijn F, Verwijs W, et al. PEG 3350 (Transipeg) versus lactulose in the treatment of childhood functional constipation: a double blind, randomised, controlled, multicentre trial. Gut 2004;53(11):1590-4.

9	Wang BX, Wang MG, Jiang MZ, et al. [Forlax in the treatment of childhood constipation: a randomized, controlled, multicenter clinical study]. Zhongguo Dang Dai Er Ke Za Zhi 2007;9(5):429-32.

10	Nurko S, Youssef NN, Sabri M, et al. PEG3350 in the treatment of childhood constipation: a multicenter, double-blinded, placebo-controlled trial. J Pediatr 2008;153(2):254-61, 61.e1.

11	Modin L, Walsted AM, Dalby K, et al. Polyethylene Glycol Maintenance Treatment for Childhood Functional Constipation: A Randomized, Placebo-controlled Trial. J Pediatr Gastroenterol Nutr 2018;67(6):732-37.

12	Thomson MA, Jenkins HR, Bisset WM, et al. Polyethylene glycol 3350 plus electrolytes for chronic constipation in children: a double blind, placebo controlled, crossover study. Arch Dis Child 2007;92(11):996-1000.

13	Gomes PB, Duarte MA, Melo Mdo C Comparison of the effectiveness of polyethylene glycol 4000 without electrolytes and magnesium hydroxide in the treatment of chronic functional constipation in children. J Pediatr (Rio J) 2011;87(1):24-8.

14	Loening-Baucke V, Pashankar DS A randomized, prospective, comparison study of polyethylene glycol 3350 without electrolytes and milk of magnesia for children with constipation and fecal incontinence. Pediatrics 2006;118(2):528-35.

15	Ratanamongkol P, Lertmaharit S, Jongpiputvanich S Polyethylene glycol 4000 without electrolytes versus milk of magnesia for the treatment of functional constipation in infants and young children: a randomized controlled trial. Asian Biomedicine 2009;3(4):391-99.

16	Karami H, Khademloo M, Niari P Polyethylene Glycol versus Paraffin for the Treatment of Childhood Functional Constipation. Iran J Pediatr 2009;19(3):255-61.

17	Rafati M, Karami H, Salehifar E, et al. Clinical efficacy and safety of polyethylene glycol 3350 versus liquid paraffin in the treatment of pediatric functional constipation. Daru 2011;19(2):154-8.

18	Dehghani SM, Bahroloolomifard MS, Yousefi G, et al. A randomized controlled double blinded trial to evaluate efficacy of oral administration of black strap molasses (sugarcane extract) in comparison with polyethylene glycol on pediatric functional constipation. J Ethnopharmacol 2019;238(111845.

19	Esmaeilidooki MR, Mozaffarpur SA, Mirzapour M, et al. Comparison Between the Cassia Fistula`s Emulsion With Polyethylene Glycol (PEG4000) in the Pediatric Functional Constipation: A Randomized Clinical Trial. Iran Red Crescent Med J 2016;18(7):e33998.

20	Nasri P, Saeidi S, Saneian H, et al. Comparative Evaluation between the LaxaPlus Barij(®) and Polyethylene Glycol (4000) in the Pediatric Functional Constipation in Children 2-15 Years Old. J Res Pharm Pract 2021;10(4):180-84.

21	Imanieh MH, Honar N, Mohagheghzadeh A, et al. Rosa damascena together with brown sugar mitigate functional constipation in children over 12 months old: A double-blind randomized controlled trial. J Ethnopharmacol 2022;298(115582.

22	Nimrouzi M, Sadeghpour O, Imanieh MH, et al. Flixweed vs. Polyethylene Glycol in the Treatment of Childhood Functional Constipation: A Randomized Clinical Trial. Iran J Pediatr 2015;25(2):e425.

23	Saneian H, Ghaedi S, Famouri F, et al. Comparing the Effect of a Herbal-based Laxative (Goleghand®) and Polyethylene Glycol on Functional Constipation among Children: A Randomized Controlled Trial. J Res Pharm Pract 2021;10(1):43-49.

24	Tavassoli S, Eftekhari K, Karimi M, et al. Effectiveness of Viola Flower Syrup Compared with Polyethylene Glycol in Children with Functional Constipation: A Randomized, Active-Controlled Clinical Trial. Evid Based Complement Alternat Med 2021;2021(9915289.

25	Cassettari VMG, Machado NC, Lourenção P, et al. Combinations of laxatives and green banana biomass on the treatment of functional constipation in children and adolescents: a randomized study. J Pediatr (Rio J) 2019;95(1):27-33.

26	Quitadamo P, Coccorullo P, Giannetti E, et al. A randomized, prospective, comparison study of a mixture of acacia fiber, psyllium fiber, and fructose vs polyethylene glycol 3350 with electrolytes for the treatment of chronic functional constipation in childhood. Journal of Pediatrics 2012;161(4):710-15.e1.

27	Strisciuglio C, Coppola V, Russo M, et al. Promelaxin Microenemas Are Non-inferior to Oral Polyethylene Glycol for the Treatment of Functional Constipation in Young Children: A Randomized Clinical Trial. Front Pediatr 2021;9(753938.

28	Foroughi M, Ardakani AT, Taghizadeh M, et al. Comparing the Effect of Polyethylene Glycol, Psyllium Seed Husk Powder, and Probiotics on Constipation in Children. Iran J Pediatr 2022;32(5).

29	Bekkali NLH, Hoekman DR, Liem O, et al. Polyethylene Glycol 3350 With Electrolytes Versus Polyethylene Glycol 4000 for Constipation: A Randomized, Controlled Trial. J Pediatr Gastroenterol Nutr 2018;66(1):10-15.

30	Savino F, Viola S, Erasmo M, et al. Efficacy and tolerability of peg-only laxative on faecal impaction and chronic constipation in children. A controlled double blind randomized study vs a standard peg-electrolyte laxative. BMC Pediatr 2012;12(178.

31	Dziechciarz P, Horvath A, Szajewska H Polyethylene glycol 4000 for treatment of functional constipation in children. J Pediatr Gastroenterol Nutr 2015;60(1):65-8.

32	Cao Y, Liu SM Lactulose for the treatment of Chinese children with chronic constipation: A randomized controlled trial. Medicine (Baltimore) 2018;97(52):e13794.

33	Pitzalis G, Deganello F, Mariani P, et al. [Lactitol in chronic idiopathic constipation in children]. Pediatr Med Chir 1995;17(3):223-6.

34	Farahmand F A randomised trial of liquid paraffin versus lactulose in the treatment of chronic functional constipation in children. Acta Medica Iranica 2007;45(3):183-88.

35	Urganci N, Akyildiz B, Polat TB A comparative study: the efficacy of liquid paraffin and lactulose in management of chronic functional constipation. Pediatr Int 2005;47(1):15-9.

36	Kokke FTM, Scholtens PAMJ, Alles MS, et al. A dietary fiber mixture versus lactulose in the treatment of childhood constipation: a double-blind randomized controlled trial. Journal of Pediatric Gastroenterology and Nutrition 2008;47(5):592-97.

37	Ustundag G, Kuloglu Z, Kirbas N, et al. Can partially hydrolyzed guar gum be an alternative to lactulose in treatment of childhood constipation? Turkish Journal of Gastroenterology 2010;21(4):360-64.

38	Ala S, Haghighat M, Dehghani SM, et al. Response and Recurrence Rate After Treatment With Polyethylene Glycol Versus Polyethylene Glycol Plus Lactulose in Children With Chronic Functional Constipation: A Randomized Controlled Trial. J Compr Ped 2015;6(2):6-10.

39	Lee KJ, Ryoo E, Lee YM, et al. Saccharomyces boulardii and Lactulose for Childhood Functional Constipation: A Multicenter Randomized Controlled Trial. J Neurogastroenterol Motil 2022;28(3):454-62.

40	Asburce M, Olgac B, Sezer OB, et al. Comparison of probiotic and lactulose treatments in children with functional constipation and determination of the effects of constipation treatment on quality of life. [Turkish]. Cocuk Sagligi ve Hastaliklari Dergisi 2013;56(1):1-7.

41	Bu LN, Chang MH, Ni YH, et al. Lactobacillus casei rhamnosus Lcr35 in children with chronic constipation. Pediatrics International 2007;49(4):485-90.

42	Kubota M, Ito K, Tomimoto K, et al. Lactobacillus reuteri DSM 17938 and magnesium oxide in children with functional chronic constipation: A double-blind and randomized clinical trial. Nutrients 2020;12(225).

43	Mozaffarpur SA, Naseri M, Esmaeilidooki MR, et al. The effect of cassia fistula emulsion on pediatric functional constipation in comparison with mineral oil: A randomized, clinical trial. DARU, Journal of Pharmaceutical Sciences 2012;20(83).

44	Khodadad A, Sabbaghian M Role of synbiotics in the treatment of childhood constipation: A double-blind randomized placebo controlled trial. Iranian Journal of Pediatrics 2010;20(4):387-92.

45	Mugie SM, Korczowski B, Bodi P, et al. Prucalopride is no more effective than placebo for children with functional constipation. Gastroenterology 2014;147(6):1285-95.e1.

46	Benninga MA, Hussain SZ, Sood MR, et al. Lubiprostone for Pediatric Functional Constipation: Randomized, Controlled, Double-Blind Study With Long-term Extension. Clin Gastroenterol Hepatol 2022;20(3):602-10.e5.

47	Elkaragy ES, Shamseya MM, Metwally RH, et al. Efficacy of lubiprostone for functional constipation treatment in adolescents and children: Randomized controlled trial. J Pediatr Gastroenterol Nutr 2024;78(4):800-09.

48	Di Lorenzo C, Nurko S, Hyams JS, et al. Linaclotide Safety and Efficacy in Children Aged 6 to 17 Years With Functional Constipation. The American Journal of GASTROENTEROLOGY; 2019.

49	Di Lorenzo C, Khlevner J, Rodriguez-Araujo G, et al. Efficacy and safety of linaclotide in treating functional constipation in paediatric patients: a randomised, double-blind, placebo-controlled, multicentre, phase 3 trial. Lancet Gastroenterol Hepatol 2024;9(3):238-50.

50	Di Lorenzo C, Robert J, Rodriguez-Araujo G, et al. Safety and efficacy of linaclotide in children aged 2-5 years with functional constipation: Phase 2, randomized study. J Pediatr Gastroenterol Nutr 2024;79(3):510-18.

51	Bongers ME, van den Berg MM, Reitsma JB, et al. A randomized controlled trial of enemas in combination with oral laxative therapy for children with chronic constipation. Clin Gastroenterol Hepatol 2009;7(10):1069-74.

52	Dehghani SM, Askarian M, Kaffashan HA Oral domperidone has no additional effect on chronic functional constipation in children: a randomized clinical trial. Indian J Gastroenterol 2014;33(2):125-30.

53	Iacono G, Cavataio F, Montalto G, et al. Intolerance of cow's milk and chronic constipation in children. N Engl J Med 1998;339(16):1100-4.

54	Fabrizio V, Harris CL, Walsh KR, et al. Softer More Frequent Stools in Infants With Difficult Stooling Fed Hydrolyzed Protein Formula With Added Prebiotics: Randomized Controlled Trial. Front Pediatr 2022;10(894626.

55	Mohammadi Bourkheili A, Mehrabani S, Esmaeili Dooki M, et al. Effect of Cow's-milk-free diet on chronic constipation in children; A randomized clinical trial. Caspian J Intern Med 2021;12(1):91-96.

56	Dehghani SM, Ahmadpour B, Haghighat M, et al. The role of cow's milk allergy in pediatric chronic constipation: A randomized clinical trial. Iranian Journal of Pediatrics 2012;22(4):468-74.

57	Chmielewska A, Horvath A, Dziechciarz P, et al. Glucomannan is not effective for the treatment of functional constipation in children: A double-blind, placebo-controlled, randomized trial. Clinical Nutrition 2011;30(4):462-68.

58	Weber TK, Toporovski MS, Tahan S, et al. Dietary fiber mixture in pediatric patients with controlled chronic constipation. Journal of Pediatric Gastroenterology and Nutrition 2014;58(3):297-302.

59	Loening-Baucke V, Miele E, Staiano A Fiber (glucomannan) is beneficial in the treatment of childhood constipation. Pediatrics 2004;113(3):e259-64.

60	Souza DDS, Tahan S, Weber TK, et al. Randomized, Double-Blind, Placebo-Controlled Parallel Clinical Trial Assessing the Effect of Fructooligosaccharides in Infants with Constipation. Nutrients 2018;10(11).

61	Young RJ, Beerman LE, Vanderhoof JA Increasing oral fluids in chronic constipation in children. Gastroenterology nursing : the official journal of the Society of Gastroenterology Nurses and Associates 1998;21(4):156-61.

62	Coccorullo P, Strisciuglio C, Martinelli M, et al. Lactobacillus reuteri (DSM 17938) in infants with functional chronic constipation: A double-blind, randomized, placebo-controlled study. Journal of Pediatrics 2010;157(4):598-602.

63	Gan D, Chen J, Tang X, et al. Impact of a probiotic chewable tablet on stool habits and microbial profile in children with functional constipation: A randomized controlled clinical trial. Front Microbiol 2022;13(985308.

64	Lojanatorn P, Phrommas J, Tanpowpong P, et al. Efficacy of Bacillus clausii in Pediatric Functional Constipation: A Pilot of a Randomized, Double-Blind, Placebo-Controlled Trial. Indian Pediatr 2023;60(6):453-58.

65	Tabbers MM, Chmielewska A, Roseboom MG, et al. Fermented milk containing Bifidobacterium lactis DN-173 010 in childhood constipation: A randomized, double-blind, controlled trial. Pediatrics 2011;127(6):e1392-e99.

66	Tjokronegoro SDP, Advani N, Firmansyah A Effectiveness of probiotics in the management of functional constipation in children: A randomized, double-blind, placebo-controlled trial. International Journal of Probiotics and Prebiotics 2020;15(1):1-6.

67	Wojtyniak K, Horvath A, Dziechciarz P, et al. Lactobacillus casei rhamnosus Lcr35 in the Management of Functional Constipation in Children: A Randomized Trial. Journal of Pediatrics 2017;184(101-05.e1.

68	Žaja O, Fiolić M, Ćuk MC, et al. "The role of L. reuteri DSM17938 in nutritional recovery and treatment of constipation in children and adolescents with anorexia nervosa - a randomized, double blind, placebo controlled study". Clin Nutr ESPEN 2021;46(47-53.

69	Olgaç MA, Sezer OB, Özçay F Fonksiyonel kabızlığı olan çocuklarda probiyotik ve laktuloz tedavilerinin etkinliğinin karşılaştırılması ve kabızlık tedavisinin yaşam kalitesi üzerine olan etkisinin değerlendirilmesi. Çocuk Sağlığı ve Hastalıkları Dergisi 201356):1-7.

70	Abediny M, Ataiee P, Afkhamzadeh A, et al. The effect of probiotics on the treatment of functional constipation in children of 4-12 years of age. [Persian]. Journal of Isfahan Medical School 2016;33(368):2448-54.

71	Banaszkiewicz A, Szajewska H Ineffectiveness of Lactobacillus GG as an adjunct to lactulose for the treatment of constipation in children: A double-blind, placebo-controlled randomized trial. Journal of Pediatrics 2005;146(3):364-69.

72	Jadresin O, Sila S, Trivic I, et al. Lack of benefit of lactobacillus reuteri DSM 17938 as an addition to the treatment of functional constipation. Journal of Pediatric Gastroenterology and Nutrition 2018;67(6):763-66.

73	Russo M, Giugliano FP, Quitadamo P, et al. Efficacy of a mixture of probiotic agents as complementary therapy for chronic functional constipation in childhood. Italian Journal of Pediatrics 2017;43(24).

74	Wegner A, Banaszkiewicz A, Kierkus J, et al. The effectiveness of Lactobacillus reuteri DSM 17938 as an adjunct to macrogol in the treatment of functional constipation in children. A randomized, double-blind, placebo-controlled, multicentre trial. Clinics and Research in Hepatology and Gastroenterology 2018;42(5):494-500.

75	Sadeghzadeh M, Rabieefar A, Khoshnevisasl P, et al. The effect of probiotics on childhood constipation: a randomized controlled double blind clinical trial. Int J Pediatr 2014;2014(937212.

76	Guerra PVP, Lima LN, Souza TC, et al. Pediatric functional constipation treatment with bifidobacterium-containing yogurt: A crossover, double-blind, controlled trial. World Journal of Gastroenterology 2011;17(34):3916-21.

77	Sevilla DAC, Hofman D, Madero SD, et al. The use of two Comfort Young Child Formulas in the dietary management of toddlers with functional constipation: a randomized controlled trial. BMC Pediatr 2022;22(1):672.

78	Bongers ME, de Lorijn F, Reitsma JB, et al. The clinical effect of a new infant formula in term infants with constipation: a double-blind, randomized cross-over trial. Nutr J 2007;6(8.

79	Savino F, Maccario S, Castagno E, et al. Advances in the management of digestive problems during the first months of life. Acta Paediatr Suppl 2005;94(449):120-4.

80	Baştürk A, Artan R, Atalay A, et al. Investigation of the efficacy of synbiotics in the treatment of functional constipation in children: a randomized double-blind placebo-controlled study. Turkish journal of gastroenterology 2017;28(5):388‐93.

81	Van Dijk M, Bongers MEJ, De Vries GJ, et al. Behavioral therapy for childhood constipation: A randomized, controlled trial. Pediatrics 2008;121(5):e1334-e41.

82	Loening-Baucke V Modulation of abnormal defecation dynamics by biofeedback treatment in chronically constipated children with encopresis. Journal of Pediatrics 1990;116(2):214-22.

83	Sunic-Omejc M, Mihanovic M, Jurcic D, et al. Efficiency of biofeedback therapy for chronic constipation in children. Collegium antropologicum 2002;26(93-101.

84	Van der Plas RN, Benninga MA, Buller HA, et al. Biofeedback training in treatment of childhood constipation: A randomised controlled study. Lancet 1996;348(9030):776-80.

85	Castilla LKS, Serrano CAM, Figueroa AMC. Biofeedback therapy in children with chronic functional constipation and sensitivity disorders according to London criteria. ESPGHAN and NASPGHAN; 2021:603.

86	Croffie JM, Ammar MS, Pfefferkorn MD, et al. Assessment of the effectiveness of biofeedback in children with dyssynergic defecation and recalcitrant constipation/encopresis: Does home biofeedback improve long-term outcomes. Clinical Pediatrics 2005;44(1):63-71.

87	van Engelenburg-van Lonkhuyzen ML, Bols EMJ, Benninga MA, et al. Effectiveness of Pelvic Physiotherapy in Children With Functional Constipation Compared With Standard Medical Care. Gastroenterology 2017;152(1):82-91.

88	Silva CAG, Motta MEFA The use of abdominal muscle training, breathing exercises and abdominal massage to treat paediatric chronic functional constipation. Colorectal Disease 2013;15(5):e250-e55.

89	Blanco Díaz M, Bousoño García C, Segura Ramírez DK, et al. Manual Physical Therapy in the Treatment of Functional Constipation in Children: A Pilot Randomized Controlled Trial. J Altern Complement Med 2020;26(7):620-27.

90	Clarke MC, Chase JW, Gibb S, et al. Improvement of quality of life in children with slow transit constipation after treatment with transcutaneous electrical stimulation. J Pediatr Surg 2009;44(6):1268-72; discussion 72.

91	de Abreu GE, de Souza LA, da Fonseca MLV, et al. Parasacral Transcutaneous Electrical Nerve Stimulation for the Treatment of Children and Adolescents with Bladder and Bowel Dysfunction: A Randomized Clinical Trial. J Urol 2021;205(6):1785-91.

92	Ladi-Seyedian SS, Sharifi-Rad L, Kajbafzadeh AM Management of Bladder Bowel Dysfunction in Children by Pelvic Floor Interferential Electrical Stimulation and Muscle Exercises: A Randomized Clinical Trial. Urology 2020;144(182-87.

93	Sharifi-Rad L, Ladi-Seyedian SS, Manouchehri N, et al. Effects of interferential electrical stimulation plus pelvic floor muscles exercises on functional constipation in children: A randomized clinical trial. American Journal of Gastroenterology 2018;113(2):295-302.

94	Khan MA, Chubarova AI [Modern possibilities of cryotherapy for chronic functional constipation in children]. 2020;97(3):68-75.

95	Yu ZT, Song JM, Qiao L, et al. A Randomized, Double-Blind, Controlled Trial of Percutaneous Tibial Nerve Stimulation With Pelvic Floor Exercises in the Treatment of Childhood Constipation. Am J Gastroenterol 2023;118(3):553-60.

96	Cai QH, Ma R, Hu SY A Randomized Controlled Trial of Chinese Patent Medicine Xiao'er Biantong Granules in the Treatment of Functional Constipation in Children. 2018;2018(4941505.

97	Qiao L, Wang LJ, Wang Y, et al. A Randomized, Double-Blind, and Placebo-Controlled Trial of Chinese Herbal Medicine in the Treatment of Childhood Constipation. Clin Transl Gastroenterol 2021;12(5):e00345.

98	Mao ZL Clinical observation of Xingqi Daozhi Tongfu Fang combined with massage therapy on 47 cases of children with functional constipation (excess syndrome). Journal of pediatrics of traditional chinese medicine [zhong yi ER ke za zhi] 2015;11(6):77‐79.

99	Xu F Clinical analysis of massage combined with oral TCM in the treatment 61 cases of functional constipation in children of deficiency syndromes. Journal of pediarics of traditional chinese medicine [zhong yi ER ke za zhi] 2015;11(2):64‐67.

100	Canbulat Sahiner N, Demirgoz Bal M A Randomized Controlled Trial Examining the Effects of Reflexology on Children With Functional Constipation. Gastroenterology nursing : the official journal of the Society of Gastroenterology Nurses and Associates 2017;40(5):393-400.

101	Shahamat M, Daneshfard B, Najib KS, et al. Dry cupping in children with functional constipation: A randomized open label clinical trial. African Journal of Traditional, Complementary and Alternative Medicines 2016;13(4):22-28.

102	Reeves PT, Jack BO, Rogers PL, et al. The Uniformed Services Constipation Action Plan: An Effective Tool for the Management of Children with Functional Constipation. J Pediatr 2023;253(46-54.e1.
