# Supplementary material for: European Society for Paediatric Gastroenterology, Hepatology and Nutrition/North American Society for Pediatric Gastroenterology, Hepatology and Nutrition guidelines for treatment of functional constipation in children aged 0–18 years
Source: J Pediatr Gastroenterol Nutr. 2026 May 20;83(2):335–66. doi: 10.1002/jpn3.70447 (PMC13446596; doi:10.1002/jpn3.70447)
Supplement: Supplementary file 2 [file JPN3-83-335-s002.docx]

**Supplementary materials file 2 - Meta Analyses and GRADE profiles**

**Information regarding this document:**

Forest plots are displayed for the primary outcomes.

Risk of Bias Legend for Forest Plots:

A: Random sequence generation (selection bias)
B: Allocation concealment (selection bias)
C: Blinding of participants and personnel (performance bias)
D: Blinding of outcome assessment (detection bias)
E: Incomplete outcome data (attrition bias)
F: Selective reporting (reporting bias)
G: Other bias

Pharmacological treatment: disimpaction

1. Rectal enema vs oral medication

**Notes:** rectal enema: dioctylsulfosuccinate sodium, once daily for 6 days (60 ml children < 6 years, and 120 ml for children of 6 years and older). Maintenance therapy was started after 6 days of disimpaction: PEG3350 + electrolytes 0.5 g/kg/day for at least 2 weeks (follow-up period). Oral medication: PEG3350 + electrolytes 1.5 gr/kg per day for 6 days. Maintenance therapy was started after 6 days of disimpaction: PEG3350 + electrolytes 0.5 g/kg/day for at least 2 weeks (follow-up period).

**Question:** Should Rectal medication vs Oral medication be used for fecal impaction in functional constipation?
**Bibliography:** Bekkali 2009

GRADE

| **Quality assessment** | | | | | | | **No of patients** | | **Effect** | | **Certainty** | **Importance** |
| --- | --- | --- | --- | --- | --- | --- | --- | --- | --- | --- | --- | --- |
|  |  |  |  |  |  |  |  |  |  |  |  |  |
| **No of studies** | **Design** | **Risk of bias** | **Inconsistency** | **Indirectness** | **Imprecision** | **Other considerations** | **Rectal medication** | **Oral medication** | **Relative (95% CI)** | **Absolute** |  |  |
| **Treatment success - absence of fecaloma on DRE. If children scared to undergo second DRE, X-ray performed (time of measurement: 6 days)** | | | | | | | | | | | | |
| 1 | randomised trials | Serious^1^ | Not serious | Not serious | Very serious^2^ | Not serious | 37/46 | 30/44 | RR: 1.18 (0.92 to 1.51) | 123 more per 1000 (from 55 less to 348 more per 1000)  Small magnitude in favour (trivial against to large in favour) | Very low |  |
| **Withdrawals due to adverse event (time of measurement: 3 weeks = 2 weeks after disimpaction)** | | | | | | | | | | | | |
| 1 | randomised trials | Serious^1^ | Not serious | Not serious | Very serious^2^ | Not serious | 4/46 | 2/44 | RR: 1.91 (0.37 to 9.92) | 41 more per 1000 (from 29 less to 406 more per 1000)  Large magnitude against (large in favour to large against) | Very low |  |
| **Defecation frequency per week - (time of measurement: 3 weeks = 2 weeks after disimpaction)** | | | | | | | | | | | | |
| 1 | randomised trials | Serious^1^ | Not serious | Not serious | Very serious^2^ | Not serious | N=41 | N=39 |  | MD: 1.00 lower (from 3.58 lower to 1.58 higher)  Trivial magnitude against (moderate against to small in favour) | Very low |  |
| **Stool consistency – number of patients with watery stools (time of measurement: 3 weeks = 2 weeks after disimpaction) – incompatible outcome** | | | | | | | | | | | | |
| 1 | randomised trials | Serious^1^ | Not serious | Not serious | Serious^3^ | Not serious | 4/41 | 13/39 | RR: 0.29 (0.10 to 0.82) | 237 less per 1000 (from 60 less to 300 less per 1000) | Low |  |
| **Fecal incontinence frequency per week - (time of measurement: 3 weeks = 2 weeks after disimpaction)^4^** | | | | | | | | | | | | |
| 1 | randomised trials | Serious^1^ | Not serious | Not serious | Very serious^2^ | Serious | N=41 | N=39 |  | MD: 0.80 lower (from 3.28 lower to 1.68 higher)  Trivial magnitude in favour (moderate in favour to small against) | Very low |  |
| **Abdominal pain - assessed with: Bowel diary (time of measurement: 3 weeks = 2 weeks after disimpaction) – incompatible outcome** | | | | | | | | | | | | |
| 1 | randomised trials | Serious^1^ | Not serious | Not serious | Serious^3^ | Not serious | 23/41 | 17/39 | RR 1.29 (0.82 to 2.01) | 126 more per 1000 (from 78 less to 440 more) | Low |  |

^1^ Downgraded one level because no safety data was reported
^2^ Downgraded two levels due to very serious imprecision

^3^Downgraded one level due to significant imprecision
^4^After 6 days mean (SD) fecal incontinence frequency per week was much higher in the PEG group: enema 3.4 (4.3) N=46, PEG: 13.6 (12.6) N=44 (MD: 10.20 lower (from 6.28 lower to 14.12 lower))

Pharmacological treatment: maintenance

PEG

1. PEG vs placebo (N=3)

**Question:** Should Polyethylene glycol (PEG) vs placebo be used for functional constipation? **Bibliography:** Modin 2018, Nurko 2008^1^, Thomson 2007

GRADE

| **Quality assessment** | | | | | | | **No of patients** | | **Effect** | | **Certainty** | **Importance** |
| --- | --- | --- | --- | --- | --- | --- | --- | --- | --- | --- | --- | --- |
|  |  |  |  |  |  |  |  |  |  |  |  |  |
| **No of studies** | **Design** | **Risk of bias** | **Inconsistency** | **Indirectness** | **Imprecision** | **Other considerations** | **PEG** | **Placebo** | **Relative (95% CI)** | **Absolute** |  |  |
| **Treatment success (time of measurement range: 2 weeks to 24 weeks)** | | | | | | | | | | | | |
| 2 | Randomised trials | Serious^2^ | Not serious | Not serious | Not serious | Not serious | 73/112 | 29/81 | RR: 1.74 (1.25 – 2.41)  NNT 4 (2-10) | 265 more per 1000 (from 90 more to 505 more per 1000)  Large magnitude in favour (small in favour to large in favour) | Moderate |  |
| **Withdrawals due to Adverse Events at study end (range: 2 weeks to 24 weeks)** | | | | | | | | | | | | |
| 2 | Randomised trials | Serious^2^ | Serious^5^ | Not serious | Very serious^6^ | Not serious | 4/112 | 3/81 | RR: 0.92 (0.06 – 14.92) | 3 less per 1000 (from 35 less to 515 more per 1000)  Trivial magnitude in favour (large in favour to large in against) | Very low |  |
| **Defecation frequency per week (time of measurement range: 2 weeks to 24 weeks)** | | | | | | | | | | | | |
| 2 | Randomised trials | Serious^2^ | Serious^5^ | Not serious | Serious^3^ | Not serious | N=76 | N=77 |  | MD: 1.32 more stools per week (from 0.15 less to 2.79 more)  Small magnitude in favour (trivial against to moderate in favour) | Very low |  |
| **Stool consistency: reported on a scale from 0-4 (0 = too loose, watery to 4 = very hard) (time of measurement: 2 weeks)** | | | | | | | | | | | | |
| 1 | Randomised trials | Serious^2^ | Not serious | Not serious | Serious^3^ | Not serious | N = 53 | N = 24 |  | MD: 0.80 lower (from 1.38 lower to 0.22 lower)  Small magnitude in favour (moderate to trivial) | Low |  |
| **Fecal incontinence frequency per week (time of measurement: 2 weeks)** | | | | | | | | | | | | |
| 1 | Randomised trials | Serious^2^ | Not serious | Not serious | Very serious | Not serious | N = 53 | N = 24 |  | MD: 1.23 higher (from 0.52 lower to 2.98 more)  Small magnitude against (trivial in favour to moderate against) | Very low |  |
| **Abdominal pain: cramping on a scale of 0-4 (0 = none to 4 = very painful) (time of measurement: 2 weeks)** | | | | | | | | | | | | |
| 1 | Randomised trials | Serious^2^ | Not serious | Not serious | Serious^3^ | Not serious | N = 53 | N = 24 |  | MD: 0.79 lower (from 1.35 lower to 0.23 lower)  Small magnitude (moderate to trivial) | Low |  |
| **Serious adverse events (time of measurement: 2 to 24 weeks)** | | | | | | | | | | | | |
| 2 | Randomised trials | Serious^2^ | Not serious | Not serious | Very serious^6^ | Not serious | 0/111 | 1/81 | RR: 0.15 (0.01 - 3.66) | 10 less per 1000 (from 12 less to 33 higher per 1000)  Small magnitude (small in favour to large against) | Very low |  |
| **Adverse events (time of measurement: 2 weeks)** | | | | | | | | | | | | |
| 1 | Randomised trials | Serious^2^ | Not serious | Not serious | Very serious^6^ | Not serious | 33/53 | 14/24 | RR: 1.07 (0.72 - 1.59) | 41 more per 1000 (from 163 less to 344 more per 1000)  Small magnitude (large in favour to large against) | Very low |  |

^1^Nurko 2008 studied three groups with different dosages: 0.2, 0.4 and 0.8 g/kg. The group of 0.2 g/kg was left out of the analysis, because this dosage is not used in clinical practice and would affect the results. Groups 0.4 and 0.8 g/kg were combined in analysis.
^2^Downgraded one level due to unclear allocation concealment and reporting ^3^Downgraded one level due to imprecision.
^5^Downgraded one level due to serious inconsistency
^6^Downgraded two levels due to very serious imprecision

*Forest plot PEG vs Placebo Treatment Success*

*Forest plot PEG vs Placebo Withdrawals due to AE*

*Forest plot PEG vs Placebo Defecation Frequency*

2. PEG vs Lactulose (N=8)

**Notes:** Dupont 2005 only included children aged 6 months – 3 years old. Treepongkaruna 2014 only included children aged 12 – 36 months old. **Question:** Should Polyethylene glycol (PEG) vs lactulose be used for functional constipation? **Bibliography:** Dheivamani 2021, Dupont 2005, Jarzebicka 2019, Saneian 2012, Treepongkaruna 2014, Uhm 2007, Voskuijl 2004, Wang 2007

GRADE

| **Quality assessment** | | | | | | | **No of patients** | | **Effect** | | **Certainty** | **Importance** |
| --- | --- | --- | --- | --- | --- | --- | --- | --- | --- | --- | --- | --- |
|  |  |  |  |  |  |  |  |  |  |  |  |  |
| **No of studies** | **Design** | **Risk of bias** | **Inconsistency** | **Indirectness** | **Imprecision** | **Other considerations** | **Polyethylene glycol** | **Lactulose** | **Relative (95% CI)** | **Absolute** |  |  |
| **Treatment success (time of measurement range: 2 weeks to 12 months)** | | | | | | | | | | | | |
| 5 | randomised trials | Serious^1^ | Serious | Not serious | Serious^2^ | Not serious | 207/288 | 151/297 | RR 1.35 (1.11 to 1.64)  NNT: 5 (range 3 – 16) | 201 more per 1000 (from 63 more to 368 more per 1000)  Moderate magnitude in favour (trivial to large) | Low |  |
| **Withdrawals due to Adverse Events at study end (range 2 weeks to 12 months)** | | | | | | | | | | | | |
| 6 | randomised trials | Serious^1^ | Not serious | Not serious | Very serious^7^ | Not serious | 19/272 | 20/272 | RR 0.97 (0.47 to 2.00) | 4 less per 1000 (from 38 less to 74 more)  Trivial magnitude (large in favour to large against) | Very low |  |
| **Defecation frequency per week (time of measurement range: 4 weeks to 3 months)** | | | | | | | | | | | | |
| 5^3^ | randomised trials | Serious^1^ | Not serious^4^ | Not serious | Not serious | Not serious | N = 254 | N = 246 |  | SMD: 0.34 higher (from 0.14 higher to 0.53 higher)  To better interpret what the results above mean, sensitivity analysis leaving the only study out that reported def freq per day was performed. This resulted in the following:  MD: 0.75 higher (0.12 higher to 1.38 higher)  Trivial magnitude in favour (trivial in favour to small in favour) | Moderate |  |
| **Painful defecation: number of patients with painful defecation - incompatible outcome, do not use** | | | | | | | | | | | | |
| 3 | randomised trials | Serious^1^ | Not serious | Not serious | Very serious^7^ | Not serious | 24/151 | 47/151 | RR: 0.54 (0.27 to 1.07) | 143 less per 1000 (from 227 less to 22 more per 1000)  Moderate magnitude in favour (large in favour to trivial against) | Very low |  |
| **Stool consistency: compared to baseline 0 = harder stool, 1 = no change from baseline, 2 = softer stool. Number of patients with improved stool consistency (time of measurement: 4 weeks)** | | | | | | | | | | | | |
| 1 | randomised trials | Not serious | Not serious | Not serious | Very serious^7^ | Not serious | 24/43 | 27/44 | RR: 0.80 (0.34 to 1.87) | 123 less per 1000 (from 405 less to 534 more per 1000)  Small magnitude against (large against to large in favour) | Low |  |
| **Fecal incontinence frequency per week (time of measurement: 8 weeks)** | | | | | | | | | | | | |
| 1 | randomised trials | Serious^1^ | Not serious | Not serious | Very serious^7^ | Not serious | N = 46 | N = 45 |  | MD: 0.27 higher (1.61 lower to 2.15 higher)  Trivial magnitude (small in favour to moderate against) | Very Low |  |
| **Abdominal pain – number of patients with abdominal pain (time of measurement: 8 weeks) - incompatible outcome, do not use** | | | | | | | | | | | | |
| 1 | randomised trials | Serious^1^ | Not serious | Not serious | Very serious^7^ | Not serious | 16/50 | 25/50 | RR: 0.64 (0.39 to 1.04) | 180 less per 1000 (from 305 less to 20 more per 1000)  Moderate magnitude in favour (large in favour to trivial against) | Very low |  |
| **Serious adverse events** | | | | | | | | | | | | |
| 6 | Randomised trials | Serious^1^ | Not serious | Not serious | Very serious^7^ | Not serious | 2/328 | 1/334 | RR: 2.00 (0.19 - 21.26) | 3 more per 1000 (from 2 less to 61 more per 1000)  Trivial magnitude against (trivial in favour to large against) | Very low |  |
| **Adverse events** | | | | | | | | | | | | |
| 5 | Randomised trials | Serious^1^ | Not serious | Not serious | Serious | Not serious | 59/215 | 72/220 | RR: 0.85 (0.69 to 1.06) | 49 less per 1000 (from 101 less to 20 more per 1000)  Small magnitude in favour (moderate in favour to trivial against) | Low |  |

^1^Downgraded one level due to lack of blinding in all studies ^2^Downgraded one level due to significant imprecision. Range in treatment success is very wide and NNT = 7.  ^3^Dupont: defecation frequency reported as median (IQR), converted to mean (SD)
^4^The study of Jarzebicka 2019 caused a high I^2^ of 96%. No clinical explanation was found for the high heterogeneity. Therefore, sensitivity analysis was performed, leaving Jarzebicka 2019 out of the meta-analysis. This resulted in a heterogeneity of I^2^=0% and inconsistency was graded as ‘not serious’.
^5^Downgraded one level due to significant imprecision
^7^Downgraded two levels due to very serious imprecision

*Forest plot PEG vs Lactulose Treatment Success*

*Forest plot PEG vs Lactulose Withdrawals due to AE*

*Forest plot PEG vs Lactulose Defecation Frequency (with Jarzebicka)*

*Forest plot PEG vs Lactulose Defecation Frequency (without Jarzebicka)*

3. PEG vs Magnesium hydroxide (N=4)

**Question:** Should Polyethylene glycol (PEG) vs magnesium hydroxide be used for functional constipation? **Bibliography:** Gomes 2011, Loening-Baucke 2006, Ratanamongkol 2009, Saneian 2012

GRADE

| **Quality assessment** | | | | | | | **No of patients** | | **Effect** | | **Certainty** | **Importance** |
| --- | --- | --- | --- | --- | --- | --- | --- | --- | --- | --- | --- | --- |
|  |  |  |  |  |  |  |  |  |  |  |  |  |
| **No of studies** | **Design** | **Risk of bias** | **Inconsistency** | **Indirectness** | **Imprecision** | **Other considerations** | **Polyethylene glycol** | **MgOH** | **Relative (95% CI)** | **Absolute** |  |  |
| **Treatment success (measured at 4 weeks)** | | | | | | | | | | | | |
| 1 | randomised trials | Very serious^1^ | Not serious | Not serious | Not serious | Not serious | 42/47 | 28/47 | RR: 1.50 (1.16 to 1.94)  NNT: 3 (range 2 – 10) | 290 more per 1000 (from 96 more to 564 more)  Large magnitude in favour (small in favour to large in favour) | Low |  |
| **Withdrawals due to Adverse Events at study end (range: 4 weeks to 12 months)** | | | | | | | | | | | | |
| 3 | randomised trials | Serious^3^ | Not serious | Not serious | Not serious | Not serious | 6/103 | 18/108 | RR: 0.38 (0.16 to 0.92) | 104 less per 1000 (from 140 less to 13 less)  Large magnitude in favour (large in favour to small in favour) | Moderate |  |
| **Defecation frequency per week (time of measurement range: 4 weeks to 3 months)** | | | | | | | | | | | | |
| 4 | randomised trials | Very serious^4^ | Very serious^5^ | Not serious | Serious^2^ | Not serious | N = 127 | N = 115 |  | MD 0.02 less stools per week (from 1.20 less to 1.16 more per week)  Trivial magnitude against (small against to small in favour) | Very low |  |
| **Painful defecation: number of patients with episodes of painful defecations – incompatible outcome, do not use** | | | | | | | | | | | | |
| 1 | randomised trials | Very serious^1^ | Not serious | Not serious | Serious^2^ | Not serious | 2/47 | 11/47 | RR: 0.18 (0.04 to 0.78) | 192 less per 1000 (from 225 less to 51 less per 1000)  Moderate magnitude in favour (large in favour to trivial in favour) | Very low |  |
| **Serious adverse events** | | | | | | | | | | | | |
| 2 | Randomised trials | Very serious^1^ | Not serious | Not serious | Very serious^6^ | Not serious | 0/85 | 0/83 | Not estimable | Not estimable | Very low |  |
| **Adverse events** | | | | | | | | | | | | |
| 1 | Randomised trials | Very serious^1^ | Not serious | Not serious | Very serious | Not serious | 20/46 | 24/43 | RR: 0.78 (0.51 - 1.19) | 123 less per 1000 (from 273 less to 106 more per 1000)  Large magnitude in favour (large in favour to moderate against) | Very low |  |

^1^Downgraded two levels due to lack of blinding and unclear selective reporting bias ^2^Downgraded one level due to significant imprecision from sparse data
^3^Downgraded one level due to lack of blinding
^4^Downgraded two levels due to lack of blinding and selective reporting bias/attrition bias
^5^Downgraded two levels due to substantial heterogeneity
^6^Downgraded two levels due to no events

*Forest plot PEG vs Magnesium hydroxide Treatment Success*

-14%

*Forest plot PEG vs Magnesium hydroxide Withdrawals due to AE*

*Forest plot PEG vs Magnesium hydroxide Defecation Frequency*

4. PEG vs Sodium picosulfate (N=1)

**Question:** Should PEG vs sodium picosulfate be used for treatment of functional constipation in children?
**Bibliography:** Cassetari 2019

GRADE

| **Quality assessment** | | | | | | | **No of patients** | | **Effect** | | **Certainty** | **Importance** |
| --- | --- | --- | --- | --- | --- | --- | --- | --- | --- | --- | --- | --- |
|  |  |  |  |  |  |  |  |  |  |  |  |  |
| **No of studies** | **Design** | **Risk of bias** | **Inconsistency** | **Indirectness** | **Imprecision** | **Other considerations** | **PEG** | **Sodiumpicosulfate** | **Relative (95% CI)** | **Absolute** |  |  |
| **Treatment success – not reported** | | | | | | | | | | | | |
|  |  |  |  |  |  |  |  |  |  |  |  |  |
| **Withdrawals due to Adverse Events – not reported** | | | | | | | | | | | | |
|  |  |  |  |  |  |  |  |  |  |  |  |  |
| **Defecation frequency – Dichotomous: number of patients having more than 3 bowel motions per week (time of measurement: 8 weeks)** | | | | | | | | | | | | |
| 1 | randomised trials | Not serious | Not serious | Not serious | Very serious^1^ |  | 10/16 | 8/17 | RR: 1.33 (0.71 – 2.50) | 155 per 1000 more (from 136 fewer to 709 more)  Moderate magnitude in favour (small against to large in favour) | Low |  |
| **Painful defecation – number of patients with painful defecation (time of measurement: 8 weeks) - incompatible outcome, do not use** | | | | | | | | | | | | |
| 1 | randomised trials | Not serious | Not serious | Not serious | Very serious^1^ |  | 4/16 | 2/17 | RR: 2.13 (0.45 - 10.05) | 133 more per 1000 (from 65 fewer to 1000 more per 1000)  Moderate magnitude against (trivial in favour to large against) | Low |  |
| **Stool consistency - number of patients with BSFS score higher than 2 (time of measurement: 8 weeks)** | | | | | | | | | | | | |
| 1 | randomised trials | Not serious | Not serious | Not serious | Very serious^1^ |  | 11/16 | 13/17 | RR: 0.90 (0.59 - 1.37) | 76 fewer per 1000 (from 336 fewer to 283 more per 1000)  Small magnitude against (large against to large in favour) | Low |  |
| **Fecal incontinence: number of patients with > 1 episode of fecal incontinence per week (time of measurement: 8 weeks) - incompatible outcome, do not use** | | | | | | | | | | | | |
| 1 | randomised trials | Not serious | Not serious | Not serious | Very serious^1^ |  | 4/16 | 5/17 | RR: 0.85 (0.28 - 2.61) | 26 fewer per 1000 (from 127 fewer to 284 more per 1000)  Trivial magnitude in favour (small in favour to large against) | Low |  |
| **Abdominal pain – number of patients with abdominal pain (time of measurement: 8 weeks) - incompatible outcome, do not use** | | | | | | | | | | | | |
| 1 | randomised trials | Not serious | Not serious | Not serious | Very serious^1^ |  | 2/16 | 5/17 | RR: 0.42 (0.10 - 1.89) | 171 fewer per 1000 (from 265 fewer to 262 more per 1000)  Moderate magnitude in favour (large in favour to large against) | Low |  |
| **Adverse events** | | | | | | | | | | | | |
| 1 | randomised trials | Not serious | Not serious | Not serious | Very serious^1^ |  | 0/16 | 0/17 | Not estimable | Not estimable | Low |  |

^1^Downgraded two levels due to very serious imprecision

*Forest plot PEG vs Sodium picosulfate hydroxide Treatment Success*

*Forest plot PEG vs Sodium picosulfate* *Defecation frequency*

5. PEG vs Sodium picosulfate + fiber (N=1)

**Notes**: Cassettari 2019 compared PEG with sodium picosulfate and green banana biomass. Green (unripe) banana contains a high amount of fiber and a high concentration of amylase-resistant starch, which is not digested or absorbed in the intestine. **Question:** Should PEG vs sodium picosulfate in combination with fiber be used for treatment of functional constipation?
**Bibliography:** Cassettari 2019

GRADE

| **Quality assessment** | | | | | | | **No of patients** | | **Effect** | | **Certainty** | **Importance** |
| --- | --- | --- | --- | --- | --- | --- | --- | --- | --- | --- | --- | --- |
|  |  |  |  |  |  |  |  |  |  |  |  |  |
| **No of studies** | **Design** | **Risk of bias** | **Inconsistency** | **Indirectness** | **Imprecision** | **Other considerations** | **PEG** | **Sodium picosulfate + fiber** | **Relative (95% CI)** | **Absolute** |  |  |
| **Treatment success – not reported** | | | | | | | | | | | | |
|  |  |  |  |  |  |  |  |  |  |  |  |  |
| **Withdrawals due to Adverse Events – not reported** | | | | | | | | | | | | |
|  |  |  |  |  |  |  |  |  |  |  |  |  |
| **Defecation frequency – dichotomous: number of patients having more than 3 bowel motions per week** | | | | | | | | | | | | |
| 1 | randomised trials | Not serious | Not serious | Not serious | Very serious^1^ | Not serious | 10/16 | 15/16 | RR: 0.67 (0.45 – 0.99) | 31 fewer per 1000 (9 fewer to 515 fewer)  Trivial magnitude against (trivial against to large against) | Low |  |
| **Painful defecation – number of patients with painful defecation (time of measurement: 8 weeks) - incompatible outcome, do not use** | | | | | | | | | | | | |
| 1 | randomised trials | Not serious | Not serious | Not serious | Very serious^1^ | Not serious | 4/16 | 1/16 | RR: 4.00 (0.50 - 31.98) | 188 more per 1000 (from 31 fewer to 1000 more per 1000)  Moderate magnitude in against (trivial in favour to large against) | Low |  |
| **Stool consistency - Number of patients with Bristol Stool Form Scale higher than 1 or 2 (meaning: children without hard stools) (time of measurement: 8 weeks)** | | | | | | | | | | | | |
| 1 | randomised trials | Not serious | Not serious | Not serious | Very serious^1^ | Not serious | 11/16 | 13/16 | RR: 0.85 (0.56 - 1.27) | 122 fewer per 1000 (from 358 fewer to 219 more per 1000)  Small magnitude against (large against to moderate in favour) | Low |  |
| **Fecal incontinence: number of patients with > 1 episode of fecal incontinence per week (time of measurement: 8 weeks) - incompatible outcome, do not use** | | | | | | | | | | | | |
| 1 | randomised trials | Not serious | Not serious | Not serious | Very serious^1^ | Not serious | 4/16 | 2/16 | RR: 2.00 (0.42 - 9.42) | 125 more per 1000 (from 73 fewer to 1000 more per 1000)  Small magnitude against (trivial in favour to large against) | Low |  |
| **Abdominal pain – number of patients with abdominal pain (time of measurement: 8 weeks) - incompatible outcome, do not use** | | | | | | | | | | | | |
| 1 | randomised trials | Not serious | Not serious | Not serious | Very serious^1^ | Not serious | 2/16 | 4/16 | RR: 0.50 (0.11 - 2.35) | 125 fewer per 1000 (from 223 fewer to 338 more per 1000)  Small magnitude in favour (large in favour to large against) | Low |  |
| **Adverse events (time of measurement: 8 weeks)** | | | | | | | | | | | | |
| 1 | randomised trials | Not serious | Not serious | Not serious | Very serious^1^ |  | 0/16 | 0/17 | Not estimable | Not estimable | Low |  |

^1^Downgraded two levels due to very serious imprecision

6. PEG vs Liquid paraffin (N=2)

**Question:** Should PEG vs liquid paraffin be used for functional constipation? **Bibliography:** Karami 2009, Rafati 2011

GRADE

| **Quality assessment** | | | | | | | **No of patients** | | **Effect** | | **Certainty** | **Importance** |
| --- | --- | --- | --- | --- | --- | --- | --- | --- | --- | --- | --- | --- |
|  |  |  |  |  |  |  |  |  |  |  |  |  |
| **No of studies** | **Design** | **Risk of bias** | **Inconsistency** | **Indirectness** | **Imprecision** | **Other considerations** | **PEG** | **Liquid paraffin** | **Relative (95% CI)** | **Absolute** |  |  |
| **Treatment success – not reported** | | | | | | | | | | | | |
|  |  |  |  |  |  |  |  |  |  |  |  |  |
| **Withdrawals due to Adverse Events at study end (4 months)** | | | | | | | | | | | | |
| 1 | Randomised trials | Very serious^1^ | Not serious | Not serious | Very serious^2^ | Not serious | 0/80 | 2/80 | RR: 0.20 (0.01 – 4.1) | 20 less per 1000 (from 25 less to 78 more)  Moderate magnitude in favour (moderate in favour to large against) | Very low |  |
| **Defecation frequency per week (time of measurement range: 1 to 4 months)** | | | | | | | | | | | | |
| 2 | Randomised trials | Very serious^1^ | Serious^3^ | Not serious | Serious^4^ | Not serious | N = 128 | N = 133 |  | MD: 0.65 higher (from 0.33 lower to 1.62 higher)  Trivial magnitude in favour (trivial against to small in favour) | Very low |  |
| **Fecal incontinence frequency per month (time of measurement: 4 weeks)** | | | | | | | | | | | | |
| 1 | Randomised trails | Very serious^1^ | Not serious | Not serious | Not serious | Not serious | N = 48 | N = 55 |  | MD: 0.00 (from 0.12 lower to 0.12 higher)  Trivial magnitude (trivial in favour to trivial against) | Low |  |
| **Fecal incontinence: number of patients with fecal incontinence (time of measurement: 4 weeks) - incompatible outcome, do not use** | | | | | | | | | | | | |
| 1 | Randomised trails | Very serious^1^ | Not serious | Not serious | Very serious^2^ | Not serious | 12/80 | 10/78 | RR: 1.17 (0.54 - 2.55) | 23 more per 1000 (from 59 less to 199 more per 1000)  Trivial magnitude against (trivial in favour to moderate against) | Very low |  |

^1^Downgraded two levels due to lack of blinding and high risk of attrition bias and selective reporting
^2^Downgraded two levels due to very serious imprecision
^3^Downgraded one level due to considerable heterogeneity
^4^Downgraded one level due to significant imprecision

*Forest plot PEG vs Liquid paraffin Defecation Frequency*

*Forest plot PEG vs Liquid paraffin Withdrawals due to AE*

7. PEG vs enema (N=1)

**Notes:** Included children aged 6 months to 4 years. The study investigated Promelaxin microenema (4 ml/5g). **Question:** Should PEG vs enemas be used for the treatment of functional constipation?
**Bibliography:** Strisciuglio 2021

GRADE

| **Quality assessment** | | | | | | | **No of patients** | | **Effect** | | **Certainty** | **Importance** |
| --- | --- | --- | --- | --- | --- | --- | --- | --- | --- | --- | --- | --- |
|  |  |  |  |  |  |  |  |  |  |  |  |  |
| **No of studies** | **Design** | **Risk of bias** | **Inconsistency** | **Indirectness** | **Imprecision** | **Other considerations** | **PEG** | **Enema** | **Relative (95% CI)** | **Absolute** |  |  |
| **Treatment success (time of measurement: 2 weeks)** | | | | | | | | | | | | |
| 1 | Randomised trials | Very serious^1^ | Not serious | Not serious | Very serious^2^ | Not serious | 43/77 | 55/76 | RR: 0.77 (0.61 – 0.98)  NNT: -6 (-70 to -4) | 167 fewer per 1000 (282 fewer to 15 fewer)  Moderate magnitude against (large magnitude against to trivial against) | Very low |  |
| **Withdrawals due to Adverse Events (time of measurement: 8 weeks)** | | | | | | | | | | | | |
| 1 | Randomised trials | Very serious | Not serious | Not serious | Very serious^2^ | Not serious | 17/77 | 24/76 | RR: 0.70 (0.41 – 1.19) | 95 fewer per 1000 (186 fewer to 60 more)  Large magnitude in favour (large in favour to large against) | Very low |  |
| **Defecation frequency – not reported** | | | | | | | | | | | | |
|  |  |  |  |  |  |  |  |  |  |  |  |  |
| **Stool consistency: number of patients with improved stool consistency (time of measurement: 8 weeks)** | | | | | | | | | | | | |
| 1 | Randomised trials | Very serious | Not serious | Not serious | Very serious^2^ | Not serious | 37/77 | 38/76 | RR: 0.96 (0.70 – 1.33) | 20 less per 1000 (from 150 less to 165 more per 1000)  Trivial magnitude against (moderate against to moderate in favour) | Very low |  |

^1^Downgraded two levels due to lack of blinding and due to the fact that after the initial 14 days of treatment, the participants received self-directed variable amounts of the agent, which could have affected the composition of the treatment groups.
^2^Downgraded two levels due to very serious imprecision

*Forest plot PEG vs* enema *Treatment Success*

*Forest plot PEG vs* enema *Withdrawals due to AE*

8. PEG4000 vs PEG3350 + Electrolytes (N=2)

**Question:** Should PEG4000 vs PEG3350 + electrolytes be used for treatment of functional constipation?
**Bibliography:** Bekkali 2018, Savino 2012

GRADE

| **Quality assessment** | | | | | | | **No of patients** | | **Effect** | | **Certainty** | **Importance** |
| --- | --- | --- | --- | --- | --- | --- | --- | --- | --- | --- | --- | --- |
|  |  |  |  |  |  |  |  |  |  |  |  |  |
| **No of studies** | **Design** | **Risk of bias** | **Inconsistency** | **Indirectness** | **Imprecision** | **Other considerations** | **PEG4000** | **PEG3350 + Electrolytes** | **Relative (95% CI)** | **Fl** |  |  |
| **Treatment success (time of measurement: 52 weeks)** | | | | | | | | | | | | |
| 1 | randomised trials | Serious^1^ | Not serious | Not serious | Very serious^4^ | Not serious | 22/49 | 24/48 | RR: 0.90 (0.59 – 1.37) | 50 less per 1000 (205 less to 185 more)  Trivial magnitude against (moderate against to moderate in favour) | Very low |  |
| **Withdrawals due to Adverse Events (time of measurement range: 4 to 52 weeks)** | | | | | | | | | | | | |
| 2 | randomised trials | Serious^3^ | Not serious | Not serious | Very serious^4^ | Not serious | 4/99 | 10/94 | RR 0.42 (0.15 – 1.19) | 61 less per 1000 (90 less to 20 more)  Large magnitude in favour (large in favour to moderate against) | Very low |  |
| **Defecation frequency per week (time of measurement range: 4 to 52 weeks)** | | | | | | | | | | | | |
| 2 | randomised trials | Serious^3^ | Very serious | Not serious | Very serious^4^ | Not serious | N = 94 | N = 81 |  | MD: 0.15 lower (from 3.37 lower to 3.08 higher)  Trivial magnitude against (moderate against to moderate in favour) | Very low |  |
| **Serious adverse events (time of measurement: 52 weeks)** | | | | | | | | | | | | |
| 1 | Randomised trials | Serious^1^ | Not serious | Not serious | Very serious^4^ | Not serious | 0/49 | 2/48 | RR: 0.20 (0.01 to 3.98) | 33 less per 1000 (from 41 less to 124 more per 1000)  Large magnitude in favour (large in favour to large against) | Very low |  |
| **Adverse events (time of measurement: 52 weeks)** | | | | | | | | | | | | |
| 1 | Randomised trials | Serious^1^ | Not seriosu | Not serious | Very serious | Not serious | 28/49 | 28/48 | RR: 0.98 (0.70 - 1.38) | 12 less per 1000 (from 175 less to 222 more per 100)  Trivial magnitude in favour (large in favour to large against) | Very low |  |

^1^Downgraded one level due to high risk of attrition bias
^2^Downgraded one level due to significant imprecision
^3^Downgraded one level due to lack of blinding and high risk of attrition bias
^4^Downgraded two levels due to very serious imprecision

*Forest plot PEG4000 vs PEG3350 + Electrolytes Treatment success*

*Forest plot PEG4000 vs PEG3350 + Electrolytes Withdrawals due to AE*

*Forest plot PEG4000 vs PEG3350 + Electrolytes Defecation frequency*

9. PEG vs fiber (N=3)

**Notes:**
Cassettari 2019 investigates green banana biomass: Green (unripe) banana contains a high amount of fiber and a high concentration of amylase-resistant starch, which is not digested or absorbed in the intestine.
Quitadamo 2012 investigates the mixture of acacia fiber, psyllium fiber (67.7% and 17.3%, respectively), and fructose (AFPFF): “*a supplement designed to enrich dietary fiber. The characteristics of low interference with the absorption of water and nutrients, together with the acacia and psyllium fiber’s low swelling index, make it a compound fit for use in the treatment of CFC.”*
Foroughi 2022 investigates Pysllium Seed Husk Powder
**Question:** Should Polyethylene glycol (PEG) vs fiber be used for functional constipation?
**Bibliography:** Cassettari 2019, Quitadamo 2012, Foroughi 2022

GRADE

| **Quality assessment** | | | | | | | **No of patients** | | **Effect** | | **Certainty** | **Importance** |
| --- | --- | --- | --- | --- | --- | --- | --- | --- | --- | --- | --- | --- |
|  |  |  |  |  |  |  |  |  |  |  |  |  |
| **No of studies** | **Design** | **Risk of bias** | **Inconsistency** | **Indirectness** | **Imprecision** | **Other considerations** | **Polyethylene glycol** | **Fiber** | **Relative (95% CI)** | **Absolute** |  |  |
| **Treatment success (measured at 8 weeks)** | | | | | | | | | | | | |
| 1 | Randomised trials | Very serious^1^ | Not serious | Not serious | Serious^2^ | Not serious | 39/50 | 28/50 | RR: 1.39 (1.05 – 1.85)  NNT: 6 (2 – 36) | 218 more per 1000 (28 more to 476 more per 1000)  Moderate magnitude in favour (trivial in favour to large in favour) | Very low |  |
| **Withdrawals due to Adverse Events at study end (time of measurement: 8 weeks)** | | | | | | | | | | | | |
| 1 | Randomised trials | Very serious^1^ | Not serious | Not serious | Very serious^3^ | Not serious | 1/50 | 0/50 | RR: 3.00 (0.13 to 71.92) | 40 more per 1000 (from 17 less to 1000 more per 1000)^7^  Large magnitude against (moderate in favour to large against) | Very low |  |
| **Defecation frequency per week (time of measurement: 8 weeks)** | | | | | | | | | | | | |
| 2 | Randomised trials | Very serious^1^ | Very serious^4^ | Not serious | Not serious | Not serious | N=83 | N=72 |  | MD: 0.80 stools per week more (0.30 stools per week less to 2.05 stools per week more)  Trivial magnitude in favour (trivial against to small in favour) | Very low |  |
| **Defecation frequency - number of patients with more than 3 bowel movements per week (time of measurement: 8 weeks)** | | | | | | | | | | | | |
| 1 | Randomised trials | Not serious | Not serious | Not serious | Very serious^3^ | Not serious | 10/16 | 9/15 | RR: 1.04 (0.59 to 1.83) | 24 more per 1000 (from 246 less to 500 more per 1000)  Trivial magnitude in favour (large against to large in favour) | Low |  |
| **Painful defecation – number of patients with painful stools (time of measurement: 8 weeks) - incompatible outcome, do not use** | | | | | | | | | | | | |
| 2 | Randomised trials | Serious | Not serious | Not serious | Serious | Not serious | 8/66 | 11/65 | RR: 0.73 (0.32 to 1.67) | 46 less per 1000 (from 115 less to 113 more per 1000)  Trivial magnitude in favour (small in favour to small against) | Low |  |
| **Number of painless bowel movements per week (time of measurement: 3 weeks) – incompatible outcome, do not use** | | | | | | | | | | | | |
| 1 | randomised trials | Very serious^5^ | Not serious | Not serious | Not serious | Not serious | N = 36 | N = 36 |  | MD: 1.58 more painless bowel movements (0.98 more painless bowel movements to 2.18 higher more painless bowel movements) | Low |  |
| **Serious adverse events (time of measurement: 8 weeks)** | | | | | | | | | | | | |
| 1 | Randomised trials | Very serious^1^ | Not serious | Not serious | Very serious^6^ | Not serious | 0/50 | 0/50 | Not estimable | Not estimable | Very low |  |
| **Adverse events (time of measurement: 8 weeks)** | | | | | | | | | | | | |
| 2 | Randomised trials | serious | Not serious | Not serious | Very serious^6^ | Not serious | 0/66 | 0/65 | Not estimable | Not estimable | Very low |  |

^1^Downgraded two levels due to lack of blinding and unclear allocation concealment and selective reporting
^2^Downgraded two levels due to significant imprecision from sparse data
^3^Downgraded two levels due to significant imprecision from sparse data
^4^Downgraded two levels due to very serious inconsistency
^5^Downgraded two levels due to unclear method of randomization and allocation and unclear attrition and selective reporting
^6^ Downgraded two levels due to significant imprecision from sparse data
^7^Added one fictional case to the control group in RevMan to calculate absolute numbers in order to better interpret results

*Forest plot PEG vs Fiber Treatment success*

*Forest plot PEG vs Fiber Withdrawals due to AE*

*Forest plot PEG vs Fiber Defecation frequency per week*

*Forest plot PEG vs Fiber Defecation frequency- number of patients with more than 3 bowel movements per week*

10. PEG + fiber vs fiber (N=1)

**Notes:** Cassettari 2019 compared PEG with green banana biomass. Green (unripe) banana contains a high amount of fiber and a high concentration of amylase-resistant starch, which is not digested or absorbed in the intestine.
**Question:** Should Polyethylene glycol (PEG) as addition to fibers vs fibers alone be used for functional constipation?
**Bibliography:** Cassettari 2019

GRADE

| **Quality assessment** | | | | | | | **No of patients** | | **Effect** | | **Certainty** | **Importance** |
| --- | --- | --- | --- | --- | --- | --- | --- | --- | --- | --- | --- | --- |
|  |  |  |  |  |  |  |  |  |  |  |  |  |
| **No of studies** | **Design** | **Risk of bias** | **Inconsistency** | **Indirectness** | **Imprecision** | **Other considerations** | **PEG + fiber** | **Fiber** | **Relative (95% CI)** | **Absolute** |  |  |
| **Treatment success – not reported** | | | | | | | | | | | | |
|  |  |  |  |  |  |  |  |  |  |  |  |  |
| **Withdrawals due to Adverse Events – not reported** | | | | | | | | | | | | |
|  |  |  |  |  |  |  |  |  |  |  |  |  |
| **Defecation frequency – Dichotomous: number of patients having more than 3 bowel motions per week (time of measurement: 8 weeks)** | | | | | | | | | | | | |
| 1 | Randomised trials | Not serious | Not serious | Not serious | Very serious^1^ | Not serious | 12/16 | 9/15 | RR: 1.25 (0.76 – 2.06) | 150 more per 1000 (from 144 less to 637 more)  Moderate magnitude in favour (small against to large in favour) | Low |  |
| **Painful defecation – number of patients with painful defecation (time of measurement: 8 weeks) – incompatible outcome, do not use** | | | | | | | | | | | | |
| 1 | Randomised trials | Not serious | Not serious | Not serious | Very serious^1^ | Not serious | 3/16 | 4/15 | RR: 0.70 (0.19 - 2.63) | 80 fewer per 1000 (from 216 less to 435 more per 1000)  trivial magnitude in favour (large in favour to large against) | Low |  |
| **Stool consistency - number of patients with BSFS score higher than 2 (time of measurement: 8 weeks)** | | | | | | | | | | | | |
| 1 | Randomised trials | Not serious | Not serious | Not serious | Very serious^1^ | Not serious | 15/16 | 13/15 | RR: 1.08 (0.85 - 1.37) | 69 more per 1000 (from 130 less to 321 more per 1000)  Trivial magnitude in favour (small against to large in favour) | Low |  |
| **Fecal incontinence: number of patients with > 1 episode of fecal incontinence per week (time of measurement: 8 weeks) – incompatible outcome, do not use** | | | | | | | | | | | | |
| 1 | Randomised trials | Not serious | Not serious | Not serious | Very serious^1^ | Not serious | 2/16 | 5/15 | RR: 0.38 (0.09 - 1.65) | 207 fewer per 1000 (from 303 less to 217 more per 1000)  Moderate in favour (large in favour to large against) | Low |  |
| **Abdominal pain – number of patients with abdominal pain (time of measurement: 8 weeks) – incompatible outcome, do not use** | | | | | | | | | | | | |
| 1 | Randomised trials | Not serious | Not serious | Not serious | Very serious^1^ | Not serious | 2/16 | 2/15 | RR: 0.94 (0.15 - 5.84) | 8 fewer per 1000 (from 113 less to 645 more per 1000)  Trivial in favour (small in favour to large against) | Low |  |
| **Adverse events (time of measurement: 8 weeks)** | | | | | | | | | | | | |
| 1 | randomised trials | Not serious | Not serious | Not serious | Very serious^1^ | Not serious | 0/16 | 0/17 | Not estimable | Not estimable | Very low^2^ |  |

^1^Downgraded two levels due to significant imprecision from extremely sparse data
^2^Graded as very low due to such sparse data and lack of events assessments for serious events are of very low certainty

*Forest plot PEG + Fiber vs Fiber Defecation frequency*

11. PEG vs synbiotics (N=1)

**Notes:** prebiotics included Pysllium Seed Husk Powder. Probiotics consisted of a mixture of Lactobacillus reuteri, Lactobacillus rhamnosus, and Bifidobacterium infantis. **Question:** Should PEG alone vs synbiotics be used for the treatment of functional constipation?
**Bibliography:** Foroughi 2022

GRADE

| **Quality assessment** | | | | | | | **No of patients** | | **Effect** | | **Certainty** | **Importance** |
| --- | --- | --- | --- | --- | --- | --- | --- | --- | --- | --- | --- | --- |
|  |  |  |  |  |  |  |  |  |  |  |  |  |
| **No of studies** | **Design** | **Risk of bias** | **Inconsistency** | **Indirectness** | **Imprecision** | **Other considerations** | **PEG** | **Synbiotics** | **Relative (95% CI)** | **Absolute** |  |  |
| **Treatment success – not reported** | | | | | | | | | | | | |
|  |  |  |  |  |  |  |  |  |  |  |  |  |
| **Withdrawals due to Adverse Events – not reported** | | | | | | | | | | | | |
|  |  |  |  |  |  |  |  |  |  |  |  |  |
| **Defecation frequency per week (time of measurement: 3 weeks)** | | | | | | | | | | | | |
| 1 | randomised trials | Very serious^1^ | Not serious | Not serious | Not serious | Not serious | N = 36 | N = 36 |  | MD: 0.75 higher (0.36 higher to 1.14 higher)  Trivial magnitude in favour (trivial in favour to trivial in favour) | Low |  |
| **Number of painless bowel movements per week (time of measurement: 3 weeks) – incompatible outcome, do not use** | | | | | | | | | | | | |
| 1 | randomised trials | Very serious^1^ | Not serious | Not serious | Serious | Not serious | N = 36 | N = 36 |  | MD: 0.89 higher (0.35 higher to 1.43 higher)  Trivial magnitude in favour (trivial in favour to small in favour) | Very low |  |

^1^Downgraded two levels due to unclear method of randomization and allocation and unclear attrition and selective reporting

*Forest plot PEG vs Synbiotics Defecation frequency*

12. PEG + probiotics vs fiber (N=1)

**Notes:** prebiotics included Pysllium Seed Husk Powder. Probiotics consisted of a mixture of Lactobacillus reuteri, Lactobacillus rhamnosus, and Bifidobacterium infantis. **Question:** Should PEG with the addition of probiotics vs fiber alone be used for the treatment of functional constipation?
**Bibliography:** Foroughi 2022

GRADE

| **Quality assessment** | | | | | | | **No of patients** | | **Effect** | | **Certainty** | **Importance** |
| --- | --- | --- | --- | --- | --- | --- | --- | --- | --- | --- | --- | --- |
|  |  |  |  |  |  |  |  |  |  |  |  |  |
| **No of studies** | **Design** | **Risk of bias** | **Inconsistency** | **Indirectness** | **Imprecision** | **Other considerations** | **PEG + probiotics** | **Fiber** | **Relative (95% CI)** | **Absolute** |  |  |
| **Treatment success – not reported** | | | | | | | | | | | | |
|  |  |  |  |  |  |  |  |  |  |  |  |  |
| **Withdrawals due to Adverse Events – not reported** | | | | | | | | | | | | |
|  |  |  |  |  |  |  |  |  |  |  |  |  |
| **Defecation frequency per week (time of measurement: 3 weeks)** | | | | | | | | | | | | |
| 1 | randomised trials | Very serious^2^ | Not serious | Not serious | Not serious | Not serious | N = 36 | N = 36 |  | MD: 1.55 higher (1.37 higher to 1.73 higher)  Small magnitude in favour (small in favour to small in favour) | Low |  |
| **Number of painless bowel movements per week (time of measurement: 3 weeks) – incompatible outcome, do not use** | | | | | | | | | | | | |
| 1 | randomised trials | Very serious^2^ | Not serious | Not serious | Not serious | Not serious | N = 36 | N = 36 |  | MD: 1.86 higher (1.33 higher to 2.39 higher)  Small magnitude in favour (small in favour to moderate in favour) | Low |  |

^1^Foroughi 2022 compared PEG + probiotics vs Pysllium Seed Husk Powder (fiber). Probiotics consisted of a mixture of Lactobacillus reuteri, Lactobacillus rhamnosus, and Bifidobacterium infantis.
^2^Downgraded two levels due to unclear method of randomization and allocation and unclear attrition and selective reporting

*Forest plot PEG + probiotics vs fiber Defecation frequency*

13. PEG vs herbal medicine (N=7)

**Question:** Should Polyethylene glycol (PEG) vs herbal medicine be used for functional constipation? **Bibliography:** Dehghani 2019, Esmaeilidooki 2016, Imanieh 2022, Nasri 2022, Nimrouzi 2015, Saneian 2021, Tavassoli 2021

GRADE

| **Quality assessment** | | | | | | | **No of patients** | | **Effect** | | **Certainty** | **Importance** |
| --- | --- | --- | --- | --- | --- | --- | --- | --- | --- | --- | --- | --- |
|  |  |  |  |  |  |  |  |  |  |  |  |  |
| **No of studies** | **Design** | **Risk of bias** | **Inconsistency** | **Indirectness** | **Imprecision** | **Other considerations** | **Polyethylene glycol** | **Herbal medicine** | **Relative (95% CI)** | **Absolute** |  |  |
| **Treatment success (time of measurement range: 4 to 8 weeks)** | | | | | | | | | | | | |
| 4 | Randomised trials | Very serious^1^ | Not serious | Not serious | Serious^2^ | Not serious | 115/164 | 116/157 | RR: 0.98 (0.86 to 1.12) | 15 less per 1000 (from 108 less to 93 more per 1000)  Trivial magnitude against (small against to small in favour) | Very low |  |
| **Withdrawals due to Adverse Events (time of measurement range: 4 to 8 weeks)** | | | | | | | | | | | | |
| 7 | Randomised trials | Very serious^1^ | Not serious | Not serious | Very serious^5^ | Not serious | 12/374 | 8/367 | RR: 1.44 (0.60 – 3.45) | 10 more per 1000 (from 9 less to 53 more per 1000)  Small magnitude against (trivial in favour to large against) | Very low |  |
| **Defecation frequency per week (time of measurement range: 3 to 8 weeks)** | | | | | | | | | | | | |
| 4 | Randomised trials | Very serious^1^ | Serious^3^ | Not serious | Very serious^5^ | Not serious | N = 203 | N = 205 |  | MD: 1.22 lower (from 2.79 lower to 0.34 higher)  Small magnitude against (moderate against to trivial in favour) | Very low |  |
| **Painful defecation: number of patients with painful defecations (time of measurement: 8 weeks) – incompatible outcome, do not use** | | | | | | | | | | | | |
| 2 | Randomised trials | Very serious^4^ | Not serious | Not serious | Very serious^5^ | Not serious | 25/90 | 28/90 | RR: 0.90 (0.57 to 1.42) | 31 less per 1000 (from 134 less to 131 more per 1000)  Trivial in favour (moderate in favour to moderate against) | Very low |  |
| **Stool consistency: frequency of hard stools per week (time of measurement: 3 to 4 weeks) – incompatible outcome, do not use** | | | | | | | | | | | | |
| 2 | Randomised trials | Very serious^4^ | Serious | Not serious | Serious | Not serious | N = 119 | N = 123 |  | MD: 0.45 more number of hard stools per week (from 0.49 less to 1.39 more hard stools per week) | Very low |  |
| **Fecal incontinence: frequency of fecal incontinence per week (time of measurement: 3 to 4 weeks)** | | | | | | | | | | | | |
| 3 | Randomised trials | Very serious^4^ | Not serious | Not serious | NOT serious | Not serious | N = 176 | N = 175 |  | MD: 0.21 episode of fecal incontinence per week more (from 0.44 episode less to 0.87 episode of fecal incontinence more per week)  Trivial against (trivial in favour to trivial against) | Low |  |
| **Adverse events (time of measurement: 4 weeks)** | | | | | | | | | | | | |
| 1 | Randomised trials | Not serious | Not serious | Not serious | Very serious^5^ | Not serious | 7/47 | 4/45 | RR: 1.68 (0.53 - 5.34) | 60 more per 1000 (from 42 less to 386 more per 1000)  Small magnitude against (small in favour to large against) | Low |  |

^1^Downgraded two levels for lack of blinding and unclear allocation bias
^2^Downgraded one level for significant imprecision
^3^Downgraded one level due to substantial heterogeneity
^4^Downgraded two levels for lack of blinding, unclear allocation bias and selective reporting
^5^Downgraded two levels due to very serious imprecision

*Forest plot PEG vs Herbal medicine Treatment success*

*Forest plot PEG vs Herbal medicine Withdrawals due to AE*

*Forest plot PEG vs Herbal medicine Defecation frequency*

*Forest plot PEG vs Herbal medicine Stool consistency*

14. PEG vs dry cupping (N=1)

**Question:** Should PEG vs dry cupping be used for treatment of functional constipation?
**Bibliography:** Shahamat 2016

GRADE

| **Quality assessment** | | | | | | | **No of patients** | | **Effect** | | **Certainty** | **Importance** |
| --- | --- | --- | --- | --- | --- | --- | --- | --- | --- | --- | --- | --- |
|  |  |  |  |  |  |  |  |  |  |  |  |  |
| **No of studies** | **Design** | **Risk of bias** | **Inconsistency** | **Indirectness** | **Imprecision** | **Other considerations** | **PEG** | **Dry cupping** | **Relative (95% CI)** | **Absolute** |  |  |
| **Treatment success: not fulfilling the Rome III criteria (time of measurement: 12 weeks)** | | | | | | | | | | | | |
| 1 | Randomised trials | Very serious^1^ | Not serious | Not serious | Very serious^3^ | Not serious | 50/60 | 46/60 | RR: 1.09 (0.91 - 1.30) | 69 more per 1000 (from 69 less to 230 more per 1000)  Small magnitude in favour (small against to moderate in favour) | Very low |  |
| **Withdrawals due to adverse events (time of measurement: 12 weeks)** | | | | | | | | | | | | |
| 1 | Randomised trials | Very serious^1^ | Not serious | Not serious | Very serious^3^ | Not serious | 0/60 | 2/60 | RR: 0.20 (0.01 - 4.08) | 27 less per 1000 (from 33 less per to 103 more per 1000)  Large magnitude in favour (large in favour to large against) | Very low |  |
| **Defecation frequency: number of patients with 2 or more bowel movements per week (time of measurement: 12 weeks) – data not adequately reported** | | | | | | | | | | | | |
|  |  |  |  |  |  |  |  |  |  |  |  |  |
| **Fecal incontinence: number of patients with 1 ≤ episode of fecal incontinence/week (time of measurement: 12 weeks)** | | | | | | | | | | | | |
| 1 | Randomised trials | Very serious^1^ | Not serious | Not serious | Very serious^3^ | Not serious | 50/60 | 55/60 | RR: 0.91 (0.79 - 1.04) | 83 less per 1000 (from 193 less to 37 more per 1000)  Small magnitude in favour (moderate in favour to trivial against) | Very low |  |

^1^Downdgraded two levels due to lack of blinding and unclear allocation concealment and reporting
^2^Downgraded one level due to significant imprecision
^3^Downgraded two levels due to very serious imprecision

*Forest plot PEG vs dry cupping Treatment success*

*Forest plot PEG vs dry cupping Withdrawals due to AE*

*Forest plot PEG vs dry cupping Defecation Frequency*

15. PEG vs Manual therapy (N=1)

**Notes:** Manual physical therapy (MPT) consisted of nine sessions of MPT with a 45-min initial session and 30 min for the rest of sessions distributed weekly during the first and second months and biweekly in the third month. MPT was the same throughout the sessions for all the participants. It was designed and performed by a physiotherapist with 20 years experience in manual physiotherapy, helped by a clinical assistant. The physical treatment was done through direct and indirect articular, vascular, visceral, muscular, and myofascial techniques for the pelvic floor, abdomen (diaphragm, colon, and ileocecal valve, duo- denojejunal flexure), skull, spine (D10–D12), and sacrum, exerting light pressure and vibration, seeking a balance in fascial tensions. it pretended to normalise the neurovegetative, mascular and joint functions.
Control group was kept in the maintenance phase for 2 months until obtaining a regular defecation habit and were treated with PEG followed by a phase of medication withdrawal.
**Question:** Should PEG vs manual therapy be used for functional constipation?
**Bibliography**: Blanco Diaz 2020

GRADE

| **Quality assessment** | | | | | | | **No of patients** | | **Effect** | | **Certainty** | **Importance** |
| --- | --- | --- | --- | --- | --- | --- | --- | --- | --- | --- | --- | --- |
|  |  |  |  |  |  |  |  |  |  |  |  |  |
| **No of studies** | **Design** | **Risk of bias** | **Inconsistency** | **Indirectness** | **Imprecision** | **Other considerations** | **PEG** | **Manual physical therapy** | **Relative (95% CI)** | **Absolute** |  |  |
| **Treatment success (time of measurement range: ) – not reported** | | | | | | | | | | | | |
|  |  |  |  |  |  |  |  |  |  |  |  |  |
| **Withdrawals due to Adverse Events at study end (range: ) – not reported** | | | | | | | | | | | | |
|  |  |  |  |  |  |  |  |  |  |  |  |  |
| **Defecation frequency per week (time of measurement: 3 months)** | | | | | | | | | | | | |
| 1 | Randomised controlled trials | Very serious^1^ | Not serious | Not serious | Serious | Not serious | N=21 | N=23 |  | MD: 1 less per week (from 0.11 less to 1.89 less per week)  Trivial magnitude against (trivial against to small against) | Very low |  |
| **Stool consistency: Modified Bristol Stool Form Scale (scale 1-5) (time of measurement: 1 month)** | | | | | | | | | | | | |
| 1 | Randomised controlled trials | Very serious^1^ | Not serious | Not serious | Not serious | Not serious | N=21 | N=25 |  | MD: 0.00 higher (from 0.43 higher to 0.43 lower on the BSFS)  Trivial magnitude (trivial against to trivial in favour) | Low |  |
| **Quality of life: PedsQL scale of 0-100, higher scores indicate better QoL (time of measurement: 3 months)** | | | | | | | | | | | | |
| 1 | Randomised controlled trials | Very serious^1^ | Not serious | Not serious | Not serious^2^ | Not serious | N=21 | N=26 |  | MD: 30.00 lower (from 24.5 lower to 35.5 lower) | Low |  |

^1^Downgraded due to lack of blinding and high risk of selective reporting
^2^Downgraded one level due to sparse data

16. PEG 0.7 g/kg/day vs 0.3 g/kg/day (N=1)

**Question:** Should 0.7 g/kg/day dose PEG vs 0.3 g/kg/day dose PEG be used for treatment of functional constipation?

**Bibliography:** Dziechciarz 2015

GRADE

| **Quality assessment** | | | | | | | **No of patients** | | **Effect** | | **Certainty** | **Importance** |
| --- | --- | --- | --- | --- | --- | --- | --- | --- | --- | --- | --- | --- |
|  |  |  |  |  |  |  |  |  |  |  |  |  |
| **No of studies** | **Design** | **Risk of bias** | **Inconsistency** | **Indirectness** | **Imprecision** | **Other considerations** | **Polyethylene glycol 0.7 g/kg/day** | **Polyethylene glycol 0.3 g/kg/day** | **Relative (95% CI)** | **Absolute** |  |  |
| **Treatment success (time of measurement at 6 weeks)** | | | | | | | | | | | | |
| 1 | Randomised trials | Serious^2^ | Not serious | Not serious | Very serious^4^ | Not serious | 43/45 | 41/47 | RR: 1.10 (0.97 – 1.24) | 87 more per 1000 (26 fewer to 209 more)  Small magnitude in favour (trivial against to moderate in favour) | Very low |  |
| **Withdrawals due to Adverse Events (time of measurement at 6 weeks)** | | | | | | | | | | | | |
| 1 | Randomised trials | Serious^2^ | Not serious | Not serious | Very serious^4^ | Not serious | 1/45 | 1/47 | RR: 1.04 (0.07 – 16.20) | 1 more per 1000 (20 fewer to 324 more)  Trivial magnitude against (moderate in favour to large against) | Very low |  |
| **Defecation frequency per week (time of measurement at 6 weeks)** | | | | | | | | | | | | |
| 1 | Randomised trials | Serious^2^ | Not serious | Not serious | Serious | Not serious | N = 44 | N = 46 |  | MD: 1.30 higher (0.76 lower to 1.84 higher)  Small magnitude in favour (trivial against to small in favour) | Low |  |
| **Painful defecation: number of patients with painful defecation (time of measurement at 6 weeks) – incompatible outcome, do not use** | | | | | | | | | | | | |
| 1 | Randomised trials | Serious^2^ | Not serious | Not serious | Very serious^4^ | Not serious | 0/44 | 11/46 | RR: 0.05 (0.00 – 0.75)  NNT: 4 (4 – 17) | 227 less per 1000 (from 60 less to 238 less per 1000)  Large magnitude in favour (trivial in favour to large in favour) | Very low |  |
| **Fecal incontinence: number of patients with fecal incontinence (time of measurement at 6 weeks)** | | | | | | | | | | | | |
| 1 | Randomised trials | Serious^2^ | Not serious | Not serious | Serious^3^ | Not serious | 1/44 | 6/46 | RR: 0.17 (0.02 – 1.39) | 108 less per 1000 (from 128 less to 51 more per 1000)  Small magnitude in favour (small in favour to trivial against) | Low |  |
| **Abdominal pain: number of patients with abdominal pain (time of measurement at 6 weeks)** | | | | | | | | | | | | |
| 1 | Randomised trials | Serious^2^ | Not serious | Not serious | Serious^3^ | Not serious | 4/44 | 11/46 | RR: 0.38 (0.13 – 1.11) | 148 less per 1000 (from 208 less to 26 more per 1000)  Moderate magnitude in favour (moderate in favour to trivial against) | Low |  |
| **Adverse events (time of measurement at 6 weeks)** | | | | | | | | | | | | |
| 1 | Randomised trials | Serious^2^ | Not serious | Not serious | Very serious^4^ | Not serious | 3/44 | 3/46 | RR: 1.05 (0.22 - 4.91) | 3 more per 1000 (from 51 less to 255 more per 1000)  Trivial against (small in favour to large against) | Very low |  |

^1^One group received a dose of 0.3 g/kg and was considered as low-dose. The other group received a dose of 0.7 g/kg and was considered as normal dose.
^2^Downgraded two levels due to lack of blinding.
^3^Downgraded one level due to significant imprecision
^4^Downgraded two levels due to very serious imprecision

*Forest plot PEG 0.7 g/kg/day vs 0.3 g/kg/day Treatment success*

*Forest plot PEG 0.7 g/kg/day vs 0.3 g/kg/day Withdrawals due to AE*

*Forest plot PEG 0.7 g/kg/day vs 0.3 g/kg/day Defecation Frequency*

Lactulose

1. Lactulose vs placebo (N=1)

**Question:** Should lactulose vs placebo be used for treatment of functional constipation?
**Bibliography:** Cao 2018

GRADE

| **Quality assessment** | | | | | | | **No of patients** | | **Effect** | | **Quality** | **Importance** |
| --- | --- | --- | --- | --- | --- | --- | --- | --- | --- | --- | --- | --- |
|  |  |  |  |  |  |  |  |  |  |  |  |  |
| **No of studies** | **Design** | **Risk of bias** | **Inconsistency** | **Indirectness** | **Imprecision** | **Other considerations** | **Lactulose** | **placebo** | **Relative (95% CI)** | **Absolute** |  |  |
| **Treatment success – not reported** | | | | | | | | | | | | |
|  |  |  |  |  |  |  |  |  |  |  |  |  |
| **Withdrawals due to Adverse Events at study end (time of measurement: 6 weeks)** | | | | | | | | | | | | |
| 1 | Randomised trials | Not serious | Not serious | Not serious | Very serious^1^ | Not serious | 4/50 | 5/50 | RR: 0.80 (0.23 – 2.81) | 20 fewer per 1000 (77 fewer to 181 more)  Moderate magnitude in favour (large in favour to large against) | Low |  |
| **Defecation frequency – not adequately reported^2^** | | | | | | | | | | | | |
|  |  |  |  |  |  |  |  |  |  |  |  |  |
| **Serious adverse events (time of measurement: 6 weeks)** | | | | | | | | | | | | |
| 1 | Randomised trials | Not serious | Not serious | Not serious | Very serious^1^ | Not serious | 0/50 | 0/50 | Not estimable | Not estimable | Low |  |

^1^Downgraded two levels due to very serious imprecision

*Forest plot Lactulose vs placebo Withdrawals due to AE*

2. Lactulose vs lactitol (N=1)

**Question:** Should lactulose vs lactitol be used as treatment of functional constipation?
**Bibliography:** Pitzalis 1995^1^

GRADE

| **Quality assessment** | | | | | | | **No of patients** | | **Effect** | | **Certainty** | **Importance** |
| --- | --- | --- | --- | --- | --- | --- | --- | --- | --- | --- | --- | --- |
|  |  |  |  |  |  |  |  |  |  |  |  |  |
| **No of studies** | **Design** | **Risk of bias** | **Inconsistency** | **Indirectness** | **Imprecision** | **Other considerations** | **Lactulose** | **lactitol** | **Relative (95% CI)** | **Absolute** |  |  |
| **Treatment success - not reported** | | | | | | | | | | | | |
|  |  |  |  |  |  |  |  |  |  |  |  |  |
| **Withdrawals due to Adverse Events – not reported** | | | | | | | | | | | | |
|  |  |  |  |  |  |  |  |  |  |  |  |  |
| **Defecation frequency per week (time of measurement: 4 weeks)** | | | | | | | | | | | | |
| 1 | Randomised trials | Very serious^2^ | Not serious | Not serious | Serious^3^ | Not serious | N = 19 | N = 23 |  | MD: 0.80 lower (2.55 lower to 0.95 more)  Trivial against (moderate against to trivial in favour) | Very low |  |
| **Painful defecation – number of patients with painful defecation (time of measurement: 4 weeks) – incompatible outcome, do not use** | | | | | | | | | | | | |
| 1 | Randomised trials | Very serious^2^ | Not serious | Not serious | Very serious^4^ | Not serious | 8/24 | 6/27 | RR: 1.50 (0.61 to 3.71) | 111 more per 1000 (from 87 less to 602 more per 1000) | Very low |  |
| **Fecal incontinence – number of patients with fecal incontinence (time of measurement: 4 weeks) – incompatible outcome, do not use** | | | | | | | | | | | | |
| 1 | Randomised trials | Very serious^2^ | Not serious | Not serious | Very serious^4^ | Not serious | 15/24 | 12/27 | RR: 1.41 (0.83, 2.37) | 182 more per 1000 (76 less to 609 more per 1000) | Very low |  |
| **Abdominal pain – number of patients with abdominal pain (time of measurement: 4 weeks) - incompatible outcome, do not use** | | | | | | | | | | | | |
| 1 | Randomised trials | Very serious^2^ | Not serious | Not serious | Very serious^4^ | Not serious | 15/24 | 9/27 | RR: 1.88 (1.01, 3.47) | 293 more per 1000 (from 3 more to 892 more per 1000) | Very low |  |

^1^Study was translated from Italian
^2^Downgraded two levels due to lack of blinding and unclear method of randomization, allocation and incomplete reporting of dropouts
^3^Downgraded one level due to significant imprecision
^4^Downgraded two levels due to very serious imprecision

*Forest plot Lactulose vs lactitol Defecation Frequency*

3. Lactulose vs Magnesium hydroxide (N=1)

**Question:** Should lactulose vs magnesium hydroxide be used as treatment of functional constipation?
**Bibliography:** Saneian 2012

GRADE

| **Quality assessment** | | | | | | | **No of patients** | | **Effect** | | **Certainty** | **Importance** |
| --- | --- | --- | --- | --- | --- | --- | --- | --- | --- | --- | --- | --- |
|  |  |  |  |  |  |  |  |  |  |  |  |  |
| **No of studies** | **Design** | **Risk of bias** | **Inconsistency** | **Indirectness** | **Imprecision** | **Other considerations** | **Lactulose** | **Magnesium hydroxde** | **Relative (95% CI)** | **Absolute** |  |  |
| **Treatment success – not reported** | | | | | | | | | | | | |
|  |  |  |  |  |  |  |  |  |  |  |  |  |
| **Withdrawals due to Adverse Events – not reported** | | | | | | | | | | | | |
|  |  |  |  |  |  |  |  |  |  |  |  |  |
| **Defecation frequency - increase in frequency per week** | | | | | | | | | | | | |
| 1 | Randomised trials | Very serious^1^ | Not serious | Not serious | Serious^2^ | Not serious | N = 30 | N = 30 |  | MD: 1.51 lower (2.53 lower to 0.49 lower)  Small magnitude against (moderate against to trivial against) | Very low |  |

^1^Downgraded two levels due to lack of blinding and high risk of selective reporting
^2^Downgraded one level due to significant imprecision

*Forest plot Lactulose vs Magnesium hydroxide Defecation Frequency*

4. Lactulose vs liquid paraffin (N=2)

**Question:** Should lactulose vs liquid paraffin be used for functional constipation? **Bibliography:** Farahmand 2007, Urganci 2005

GRADE

| **Quality assessment** | | | | | | | **No of patients** | | **Effect** | | **Certainty** | **Importance** |
| --- | --- | --- | --- | --- | --- | --- | --- | --- | --- | --- | --- | --- |
|  |  |  |  |  |  |  |  |  |  |  |  |  |
| **No of studies** | **Design** | **Risk of bias** | **Inconsistency** | **Indirectness** | **Imprecision** | **Other considerations** | **Lactulose** | **Liquid paraffin** | **Relative (95% CI)** | **Absolute** |  |  |
| **Treatment success (time of measurement: week 4-8)** | | | | | | | | | | | | |
| 2 | Randomised trials | Very serious^1^ | Very serious | Not serious | Very serious^3^ | Not serious | 51/140 | 128/147 | RR: 0.53 (0.18 – 1.51) | 410 less per 1000 (714 less to 444 more)  Large magnitude against (large against to large in favour) | Very low |  |
| **Withdrawals due to Adverse Events (time of measurement: 8 weeks)** | | | | | | | | | | | | |
| 2 | Randomised trials | Very serious^1^ | Not serious | Not serious | Very serious^3^ | Not serious | 17/140 | 20/147 | RR: 0.90 (0.50- 1.63) | 14 less per 1000 (68 less to 86 more)  Small magnitude in favour (large in favour to large against) | Very low |  |
| **Defecation frequency per week (time of measurement: week 4-8)** | | | | | | | | | | | | |
| 2 | Randomised trials | Very serious^1^ | Not serious | Not serious | Not serious | Not serious | N = 140 | N = 147 |  | MD: 4.94 lower (5.61 lower to 4.28 lower)^5^  Large magnitude against (large against to large against) | Low |  |
| **Stool consistency: based on a scale of 1-3 (1=hard, 2=firm, 3=loose stools) (time of measurement: week 4-8)** | | | | | | | | | | | | |
| 1 | Randomised trials | Very serious^1^ | Not serious | Not serious | Not serious | Not serious | N=20 | N=20 |  | MD: 0.09 lower (0.29 lower to 0.11 higher)  Trivial magnitude against (trivial against to trivial in favour) | Low |  |
| **Fecal incontinence frequency (time of measurement: week 4-8)** | | | | | | | | | | | | |
| 1 | Randomised trials | Very serious^1^ | Not serious | Not serious | Not serious | Not serious | N=120 | N=127 |  | Not estimable^6^ | Very low |  |
| **Serious adverse events (time of measurement: 8 weeks)** | | | | | | | | | | | | |
| 1 | Randomised trials | Very serious^1^ | Not serious | Not serious | Very serious^3^ | Not serious | 0/120 | 0/127 | Not estimable | Not estimable | Very low |  |

^1^Downgraded two levels due to unclear randomization and allocation and lack of blinding
^3^Downgraded two levels due to serious imprecision
^5^Relatively high numbers: mean age of children was approximately 4 years in both studies. With max age 7 years
^6^Mean (SD): liquid paraffin: 0 (0), Lactulose: 3 (4.1)

*Forest plot Lactulose vs Liquid paraffin Treatment success*

*Forest plot Lactulose vs Liquid paraffin Withdrawals due to AE*

*Forest plot Lactulose vs Liquid paraffin Defecation frequency*

5. Lactulose + PEG vs PEG (N=1)

**Notes:** Initial randomized treatment period was 4 weeks, after these 4 weeks patients who were successfully treated were followed for 12 months in order to investigate recurrence rates.  **Question:** Should lactulose as addition to PEG vs PEG alone be used for the treatment of functional constipation?
**Bibliography:** Ala 2015

GRADE

| **Quality assessment** | | | | | | | **No of patients** | | **Effect** | | **Certainty** | **Importance** |
| --- | --- | --- | --- | --- | --- | --- | --- | --- | --- | --- | --- | --- |
|  |  |  |  |  |  |  |  |  |  |  |  |  |
| **No of studies** | **Design** | **Risk of bias** | **Inconsistency** | **Indirectness** | **Imprecision** | **Other considerations** | **PEG + Lactulose** | **PEG** | **Relative (95% CI)** | **Absolute** |  |  |
| **Treatment success (time of measurement: 4 weeks)** | | | | | | | | | | | | |
| 1 | Randomised trials | Very serious^1^ | Not serious | Not serious | Very serious^2^ | Not serious | 87/100 | 70/100 | RR: 1.24 (1.07 – 1.44)  NNT: 6 (3 to 20) | 168 more per 1000 (50 more to 308 more)  Moderate magnitude in favour (trivial in favour to large in favour) | Very low |  |
| **Withdrawals due to Adverse Events (time of measurement: 4 weeks)** | | | | | | | | | | | | |
| 1 | Randomised trials | Very serious^1^ | Not serious | Not serious | Very serious^2^ | Not serious | 7/100 | 0/100 | RR: 15.00 (0.87 – 259.16) | 140 more per 1000 (from 1 less to 1000 more per 1000)  Moderate magnitude against (trivial in favour to large against) | Very low |  |
| **Defecation frequency – not reported** | | | | | | | | | | | | |
|  |  |  |  |  |  |  |  |  |  |  |  |  |
| **Adverse events (time of measurement: 4 weeks)** | | | | | | | | | | | | |
| 1 | Randomised trials | Very serious^1^ | Not serious | Not serious | Very serious^2^ | Not serious | 15/100 | 0/100 | RR: 31.00 (1.88 - 511.12) | 300 more per 1000 (from 9 more to 1000 more per 1000)  Large magnitude against (trivial against to large against) | Very low |  |

^1^Downgraded two levels due to lack of blinding and unclear allocation concealment, attrition bias and selective reporting
^2^Downgraded two levels due to very serious imprecision

*Forest plot Lactulose + PEG vs PEG Treatment success*

*Forest plot Lactulose + PEG vs PEG Withdrawals due to AE*

6. Lactulose vs fiber (N=2)

**Notes:** In Kokke 2008 both groups received a yoghurt drink containing either a fiber mixture or lactulose. The fiber mixture consisted of transgalacto-oligosaccharides, inulin, soy fiber, and resistant starch. Üstündağ 2010 investigated partially hydrolyzed guar gum (PHGG), which is a fiber source with low viscosity and it is completely fermented in the colon.  **Question:** Should lactulose vs fibers be used for treatment of functional constipation?
**Bibliography:** Kokke 2008, Üstündag 2010

GRADE

| **Quality assessment** | | | | | | | **No of patients** | | **Effect** | | **Certainty** | **Importance** |
| --- | --- | --- | --- | --- | --- | --- | --- | --- | --- | --- | --- | --- |
|  |  |  |  |  |  |  |  |  |  |  |  |  |
| **No of studies** | **Design** | **Risk of bias** | **Inconsistency** | **Indirectness** | **Imprecision** | **Other considerations** | **Lactulose** | **Fiber** | **Relative (95% CI)** | **Absolute** |  |  |
| **Treatment success – not reported** | | | | | | | | | | | | |
|  |  |  |  |  |  |  |  |  |  |  |  |  |
| **Withdrawals due to Adverse Events (time of measurement: 4 weeks to 12 weeks)** | | | | | | | | | | | | |
| 2 | Randomised trials | Very serious^1^ | Not serious | Not serious | Very serious | Not serious | 5/103 | 5/100 | RR: 0.99 (0.29 – 3.36) | 1 less per 1000 (from 36 less to 118 more)  Trivial magnitude in favour (large in favour to large against) | Very low |  |
| **Defecation frequency per week (time of measurement: 4 weeks)** | | | | | | | | | | | | |
| 1 | Randomised trials | Very serious^1^ | Not serious | Not serious | Not serious | Not serious | N = 30 | N = 31 |  | MD: 1.00 higher (from 0.28 higher to 1.72 higher)  Trivial magnitude in favour (trivial in favour to small in favour) | Low |  |
| **Stool consistency: Bristol Stool Form Scale (time of measurement: 4 weeks)** | | | | | | | | | | | | |
| 1 | Randomised trials | Very serious^1^ | Not serious | Not serious | Very serious^3^ | Not serious | N = 30 | N = 31 |  | MD: 0.40 higher (from 1.41 lower to 2.21 higher)  Trivial magnitude in favour (small against to moderate in favour) | Very low |  |
| **Fecal incontinence- number of patients with 1 or more fecal incontinence epidsodes per week. (time of measurement: 4 weeks)** | | | | | | | | | | | | |
| 1 | Randomised trials | very serious | Not serious | Not serious | Serious^2^ | Not serious | 5/70 | 9/65 | RR: 0.52 (0.18 - 1.46) | 66 less per 1000 (from 114 less to 64 more per 1000)  Trivial magnitude in favour (small in favour to trivial against) | Very low |  |
| **Abdominal pain – number of patients with abdominal pain (time of measurement: 4 weeks) – incompatible outcome, do not use** | | | | | | | | | | | | |
| 1 | Randomised trials | Very serious^1^ | Not serious | Not serious | Serious^2^ | Not serious | 3/33 | 5/35 | RR: 0.64 (0.16 - 2.45) | 51 less per 1000 (from 120 less to 207 more per 1000)  Trivial magnitude in favour (small in favour to moderate against) | Very low |  |
| **Serious adverse events (time of measurement: 4 weeks)** | | | | | | | | | | | | |
| 1 | Randomised trials | Very serious^1^ | Not serious | Not serious | Very serious^3^ | Not serious | 0/70 | 0/65 | Not estimable | Not estimable | Very low |  |
| **Adverse events (time of measurement: 4 weeks)** | | | | | | | | | | | | |
| 1 | Randomised trials | Very serious^1^ | Not serious | Not serious | Very serious^3^ | Not serious | 2/70 | 1/65 | RR: 1.86 (0.17 - 20.00) | 13 more per 1000 (from 13 less to 292 more per 1000)  Trivial magnitude against (trivial in favour to large against) | Very low |  |

^1^Downgraded two levels due to lack of blinding, high risk of selective reporting and unclear allocation concealment
^2^Downgraded one level due to significant imprecision
^3^Downgraded two levels due to very sparse data

*Forest plot Lactulose vs Fiber Withdrawals due to AE*

*Forest plot Lactulose vs Fiber Defecation frequency*

7. Lactulose vs probiotic (N=2)

**Notes:** Lee 2022 investigated probiotic S. boulardii and Olgaç 2013 investigated L. reuteri. **Question:** Should lactulose vs probiotics be used for the treatment of functional constipation? **Bibliography:** Lee 2022, Olgaç 2013

GRADE

| **Quality assessment** | | | | | | | **No of patients** | | **Effect** | | **Certainty** | **Importance** |
| --- | --- | --- | --- | --- | --- | --- | --- | --- | --- | --- | --- | --- |
|  |  |  |  |  |  |  |  |  |  |  |  |  |
| **No of studies** | **Design** | **Risk of bias** | **Inconsistency** | **Indirectness** | **Imprecision** | **Other considerations** | **Lactulose** | **Probiotics** | **Relative (95% CI)** | **Absolute** |  |  |
| **Treatment success (time of measurement: at 12 weeks)** | | | | | | | | | | | | |
| 1 | Randomised trials | Very serious^1^ | Not serious | Not serious | Very serious^2^ | Not serious | 18/69 | 4/50 | RR: 3.26 (1.18 – 9.05)  NNT: 6 (2 to 69) | 181 more per 1000 (from 14 more to 644 more per 1000)  Moderate magnitude in favour (trivial in favour to large in favour) | Very low |  |
| **Withdrawals due to Adverse Events (time of measurement: 2-12 weeks)** | | | | | | | | | | | | |
| 2 | Randomised trials | Very serious^1^ | Not serious | Not serious | Very serious^2^ | Not serious | 53/97 | 50/75 | RR: 0.76 (0.64 – 0.92) | 160 less per 1000 (from 53 less to 240 less per 1000)  Large magnitude in favour (large in favour to large against) | Very low |  |
| **Defecation frequency per week (time of measurement: range 2 to 4 weeks)** | | | | | | | | | | | | |
| 2 | Randomised trials | Very serious^1^ | Serious^3^ | Not serious | Not serious | Not serious | N=88 | N=64 |  | MD: 0.20 stools less per week (from 0.86 stools less per week to 0.46 stools more per week)  Trivial magnitude against (trivial against to trivial in favour) | Very low |  |
| **Painful defecation – frequency of painful defecations per week (time of measurement: 2 weeks) – incompatible outcome, do not use** | | | | | | | | | | | | |
| 1 | Randomised trials | Very serious^1^ | Not serious | Not serious | Not serious | Not serious | N=60 | N=37 |  | MD: 0.20 painful stools less per week (from 0.47 painful stools less per week to 0.07 painful stools more per week)  Trivial magnitude in favour (trivial in favour to trivial against) | low |  |
| **Stool consistency Bristol Stool Form Scale (time of measurement: range 2 – 4 weeks)** | | | | | | | | | | | | |
| 1 | Randomised trials | Very serious^1^ | Serious | Not serious | Not serious | Not serious | N=88 | N=62 |  | MD: 0.17 higher score on BSFS (from 0.26 lower score to 0.61 higher score on BSFS scale)  Trivial magnitude in favour (trivial against to trivial in favour) | Very low |  |
| **Fecal incontinence frequency per week (time of measurement: 2 weeks)** | | | | | | | | | | | | |
| 1 | Randomised trials | Very serious^1^ | Not serious | Not serious | Serious^3^ | Not serious | N=60 | N=37 |  | MD: 0.43 number of times less fecal incontinence per week (from 1.68 number of times less incontinence to 0.82 number of times more incontinence per week)  Trivial magnitude in favour (small in favour to trivial against) | Very low |  |

^1^Downgraded two levels due to lack of blinding and incomplete outcome data
^2^Downgraded two levels due to very serious imprecision
^3^Downgraded one level due to significant heterogeneity
^4^Downgraded one level due to significant imprecision

*Forest plot Lactulose vs probiotics Treatment success*

*Forest plot Lactulose vs probiotics Withdrawals due to AE*

*Forest plot Lactulose vs probiotics Defecation frequency*

Magnesium oxide

1. Magnesium oxide vs probiotics (N=2)

**Question:** Should magnesium oxide vs probiotics be used as treatment for functional constipation?
**Bibliography:** Bu 2007, Kubota 2020^4^

GRADE

| **Quality assessment** | | | | | | | **No of patients** | | **Effect** | | **Certainty** | **Importance** |
| --- | --- | --- | --- | --- | --- | --- | --- | --- | --- | --- | --- | --- |
|  |  |  |  |  |  |  |  |  |  |  |  |  |
| **No of studies** | **Design** | **Risk of bias** | **Inconsistency** | **Indirectness** | **Imprecision** | **Other considerations** | **Magnesium oxide** | **Probiotics** | **Relative (95% CI)** | **Absolute** |  |  |
| **Treatment success (time of measurement: 4 weeks)** | | | | | | | | | | | | |
| 1 | Randomised trials | Very serious^1^ | Not serious | Not serious | Very serious^2^ | Not serious | 13/18 | 14/18 | RR: 0.93 (0.64 - 1.36) | 54 less per 1000 (from 280 less to 280 more per 1000)  Trivial magnitude against (large against to large in favour) | Very low |  |
| **Withdrawals due to adverse events (time of measurement: 4 weeks)** | | | | | | | | | | | | |
| 1 | Randomised trials | Very serious^1^ | Not serious | Not serious | Very serious^2^ | Not serious | 2/18 | 1/18 | RR: 2.00 (0.20 - 20.15) | 56 more per 1000 (from 44 less to 1000 more per 1000)  Large magnitude against (large in favour to large against) | Very low |  |
| **Defecation frequency per week (time of measurement: 4 weeks)** | | | | | | | | | | | | |
| 1 | Randomised trials | Very serious^1^ | Not serious | Not serious | Serious^3^ | Not serious | N = 18 | N = 18 |  | MD: 0.28 lower (1.14 lower to 0.58 higher)  Trivial magnitude against (small against to trivial in favour) | Very low |  |
| **Stool consistency: Percentage of hard stools (time of measurement: 4 weeks) – incompatible outcome** | | | | | | | | | | | | |
| 1 | Randomised trials | Very serious^1^ | Not serious | Not serious | Serious^3^ | Not serious | N = 18 | N = 18 |  | MD: 1.10 (from 6.61 lower to 8.81 higher) | Very low |  |
| **Fecal incontinence – unclear if frequency is per week or per month, therefore not reported** | | | | | | | | | | | | |
| 1 |  |  |  |  |  |  |  |  |  |  |  |  |
| **Abdominal pain frequency – unclear if frequency is per week or per month, therefore not reported** | | | | | | | | | | | | |
| 1 |  |  |  |  |  |  |  |  |  |  |  |  |
| **Adverse events** | | | | | | | | | | | | |
| 1 | Randomised trials | Very serious^1^ | Not serious | Not serious | Very serious^2^ | Not serious | 1/18 | 0/18 | RR: 3.17 (0.12 - 83.17) | Not estimable | Very low |  |

^1^Downdgraded two levels due to unclear method of allocation, attrition and reporting
^2^Downgraded two levels due to significant imprecision and sparse data
^3^Downgraded one level due to significant imprecision
^4^Kubota 2020 reports change from baseline as least square means, therefore not added to the meta-analyses.

*Forest plot Magnesium oxide vs probiotics Treatment success*

*Forest plot Magnesium oxide vs probiotics Withdrawals due to AE*

*Forest plot Magnesium oxide vs probiotics Defecation frequency*

2. Magnesium oxide + probiotics vs probiotics (N=1)

**Question:** Should magnesium oxide as addition to probiotics vs probiotics alone be used for treatment of functional constipation?
**Bibliography:** Kubota 2020

GRADE

| **Quality assessment** | | | | | | | **No of patients** | | **Effect** | | **Quality** | **Importance** |
| --- | --- | --- | --- | --- | --- | --- | --- | --- | --- | --- | --- | --- |
|  |  |  |  |  |  |  |  |  |  |  |  |  |
| **No of studies** | **Design** | **Risk of bias** | **Inconsistency** | **Indirectness** | **Imprecision** | **Other considerations** | **Magnesium oxide + probiotics** | **Probiotics** | **Relative (95% CI)** | **Absolute** |  |  |
| **Treatment success – not reported** | | | | | | | | | | | | |
|  |  |  |  |  |  |  |  |  |  |  |  |  |
| **Withdrawals due to adverse events (time of measurement: 4 weeks) – not reported per group** | | | | | | | | | | | | |
| 1 | Randomised controlled trial | Very serious^1^ | Not serious | Not serious | Not estimable |  |  |  |  |  |  |  |
| **Defecation frequency per week (time of measurement: 4 weeks) – reported as change from baseline in least square means** | | | | | | | | | | | | |
| 1 | Randomised controlled trial | Very serious^1^ | Not serious | Not serious | Not estimable |  |  |  |  |  |  |  |
| **Stool consistency (time of measurement: 4 weeks) – Bristol stool form scale, reported as change from baseline in least square means.** | | | | | | | | | | | | |
| 1 | Randomised controlled trial | Very serious^1^ | Not serious | Not serious | Not estimable |  |  |  |  |  |  |  |

^1^Downgraded two levels due to unclear method of allocation concealment, attrition bias and selective reporting

*Forest plot Magnesium oxide + probiotics vs probiotics Defecation frequency*

3. Magnesium oxide vs placebo (N=1)

**Question:** Should magnesium oxide vs placebo be used for treatment of functional constipation?
**Bibliography:** Bu 2007

GRADE

| **Quality assessment** | | | | | | | **No of patients** | | **Effect** | | **Certainty** | **Importance** |
| --- | --- | --- | --- | --- | --- | --- | --- | --- | --- | --- | --- | --- |
|  |  |  |  |  |  |  |  |  |  |  |  |  |
| **No of studies** | **Design** | **Risk of bias** | **Inconsistency** | **Indirectness** | **Imprecision** | **Other considerations** | **Magnesium oxide** | **Placebo** | **Relative (95% CI)** | **Absolute** |  |  |
| **Treatment success (time of measurement: 4 weeks)** | | | | | | | | | | | | |
| 1 | Randomised trials | Very serious^1^ | Not serious | Not serious | Very serious^2^ | Not serious | 13/18 | 1/9 | RR: 6.50 (1.00 - 42.17) | 611 more per 1000 (from 0 more to 1000 more per 1000)  Large magnitude in favour (trivial to large in favour) | Very low |  |
| **Withdrawals due to adverse events (time of measurement: 4 weeks)** | | | | | | | | | | | | |
| 1 | Randomised trials | Very serious^1^ | Not serious | Not serious | Very serious^2^ | Not serious | 2/18 | 1/9 | RR: 1.00 (0.10 - 9.61) | 0 more per 1000 (from 100 less to 957 more per 1000)  Trivial magnitude (large in favour to large against) | Very low |  |
| **Defecation frequency per week (time of measurement: 4 weeks)** | | | | | | | | | | | | |
| 1 | Randomised trials | Very serious^1^ | Not serious | Not serious | Not serious | Not serious | N = 18 | N = 9 |  | MD: 2.15 higher (from 1.46 higher to 2.84 higher)  Small magnitude in favour (small in favour to small in favour) | Low |  |
| **Stool consistency: Percentage of hard stools (time of measurement: 4 weeks) – incompatible outcome** | | | | | | | | | | | | |
| 1 | Randomised trials | Very serious^1^ | Not serious | Not serious | Not serious | Not serious | N = 18 | N = 9 |  | MD: 52.00 lower (57.40 lower to 46.60 lower) | Low |  |
| **Fecal incontinence – unclear if frequency is per week or per month, therefore not reported** | | | | | | | | | | | | |
| 1 |  |  |  |  |  |  |  |  |  |  |  |  |
| **Abdominal pain frequency – unclear if frequency is per week or per month, therefore not reported** | | | | | | | | | | | | |
| 1 |  |  |  |  |  |  |  |  |  |  |  |  |
| **Adverse events** | | | | | | | | | | | | |
| 1 | Randomised trials | Very serious^1^ | Not serious | Not serious | Very serious^2^ | Not serious | 1/18 | 0/9 | RR: 3.17 (0.12 - 83.17) | Not estimable | Very low |  |

^1^Downdgraded one level due to unclear allocation concealment, attrition and selective reporting
^2^Downgraded two levels due to significant imprecision and sparse data

*Forest plot Magnesium oxide vs placebo Treatment success*

*Forest plot Magnesium oxide vs placebo Withdrawals due to AE*

*Forest plot Magnesium oxide vs placebo Defecation frequency*

Sodium picosulfate

1. Sodium picosulfate vs PEG + fibers (N=1)

**Notes**: Cassettari 2019 compared sodium picosulfate with PEG and green banana biomass. Green (unripe) banana contains a high amount of fiber and a high concentration of amylase-resistant starch, which is not digested or absorbed in the intestine. **Question:** Should sodium picosulfate vs PEG in combination with fibers be used for treatment of functional constipation?
**Bibliography:** Cassettari 2019

GRADE

| **Quality assessment** | | | | | | | **No of patients** | | **Effect** | | **Certainty** | **Importance** |
| --- | --- | --- | --- | --- | --- | --- | --- | --- | --- | --- | --- | --- |
|  |  |  |  |  |  |  |  |  |  |  |  |  |
| **No of studies** | **Design** | **Risk of bias** | **Inconsistency** | **Indirectness** | **Imprecision** | **Other considerations** | **Sodium picosulfate** | **PEG + fiber** | **Relative (95% CI)** | **Absolute** |  |  |
| **Treatment success – not reported** | | | | | | | | | | | | |
|  |  |  |  |  |  |  |  |  |  |  |  |  |
| **Withdrawals due to Adverse Events – not reported** | | | | | | | | | | | | |
|  |  |  |  |  |  |  |  |  |  |  |  |  |
| **Defecation frequency – Dichotomous: number of patients having more than 3 bowel motions per week** | | | | | | | | | | | | |
| 1 | randomised trials | Not serious | Not serious | Not serious | Very serious^1^ | Not serious | 8/17 | 12/16 | RR: 0.63 (0.35 – 1.12) | 278 fewer per 1000 (from 488 fewer to 90 more)  Large magnitude against (large against to small in favour) | Low |  |
| **Painful defecation – number of patients (time of measurement: 8 weeks) – incompatible outcome, do not use** | | | | | | | | | | | | |
| 1 | randomised trials | Not serious | Not serious | Not serious | Very serious^1^ | Not serious | 2/17 | 3/16 | RR: 0.63 (0.12 - 3.28) | 69 fewer per 1000 (from 165 fewer to 428 more per 1000)  Small magnitude in favour (moderate in favour to large against) | Low |  |
| **Stool consistency - number of patients with Bristol Stool Form Scale higher than 1 or 2 (1= very hard stools, 7 = very loose stools) (time of measurement: 8 weeks)** | | | | | | | | | | | | |
| 1 | randomised trials | Not serious | Not serious | Not serious | Very serious^1^ | Not serious | 13/17 | 15/16 | RR: 0.82 (0.61 - 1.09) | 169 fewer per 1000 (from 366 fewer to 84 more per 1000)  Moderate magnitude against (large against to small in favour) | Low |  |
| **Fecal incontinence: number of patients with > 1 episode of fecal incontinence per week (time of measurement: 8 weeks)** | | | | | | | | | | | | |
| 1 | randomised trials | Not serious | Not serious | Not serious | Very serious^1^ | Not serious | 5/17 | 2/16 | RR: 2.35 (0.53 - 10.45) | 169 more per 1000 (from 59 fewer to 1000 more per 1000)  Moderate magnitude against (trivial in favour to large against) | Low |  |
| **Abdominal pain – number of patients (time of measurement: 8 weeks) – incompatible outcome, do not use** | | | | | | | | | | | | |
| 1 | randomised trials | Not serious | Not serious | Not serious | Very serious^1^ | Not serious | 5/17 | 2/16 | RR: 2.35 (0.53 - 10.45) | 169 more per 1000 (from 59 fewer to 1000 more per 1000)  Moderate magnitude against (trivial in favour to large against) | Low |  |
| **Adverse events (time of measurement: 8 weeks)** | | | | | | | | | | | | |
| 1 | randomised trials | Not serious | Not serious | Not serious | Very serious^1^ |  | 0/16 | 0/17 | Not estimable | Not estimable | Low |  |

^1^Downgraded two levels due to very serious imprecision

*Forest plot Sodium picosulfate vs PEG + fibers Defecation frequency*

2. Sodium picosulfate vs fiber (N=1)

**Notes:** Cassettari 2019 compared sodium picosulfate with green banana biomass. Green (unripe) banana contains a high amount of fiber and a high concentration of amylase-resistant starch, which is not digested or absorbed in the intestine. **Question:** Should sodium picosulfate vs fiber be used for treatment of functional constipation?
**Bibliography:** Cassettari 2019

GRADE

| **Quality assessment** | | | | | | | **No of patients** | | **Effect** | | **Certainty** | **Importance** |
| --- | --- | --- | --- | --- | --- | --- | --- | --- | --- | --- | --- | --- |
|  |  |  |  |  |  |  |  |  |  |  |  |  |
| **No of studies** | **Design** | **Risk of bias** | **Inconsistency** | **Indirectness** | **Imprecision** | **Other considerations** | **Sodium picosulfate** | **Fiber** | **Relative (95% CI)** | **Absolute** |  |  |
| **Treatment success – not reported** | | | | | | | | | | | | |
|  |  |  |  |  |  |  |  |  |  |  |  |  |
| **Withdrawals due to Adverse Events – not reported** | | | | | | | | | | | | |
|  |  |  |  |  |  |  |  |  |  |  |  |  |
| **Defecation frequency – Dichotomous: number of patients having more than 3 bowel motions per week (time of measurement: 8 weeks)** | | | | | | | | | | | | |
| 1 | randomised trials | Not serious | Not serious | Not serious | Very serious^1^ | Not serious | 8/17 | 9/15 | RR: 0.78 (0.41 – 1.51) | 132 fewer per 1000 (from 355 fewer to 306 more)  Small magnitude against (large against to large in favour) | Low |  |
| **Painful defecation – number of patients (time of measurement: 8 weeks) – incompatible outcome, do not use** | | | | | | | | | | | | |
| 1 | randomised trials | Not serious | Not serious | Not serious | Very serious^1^ | Not serious | 2/17 | 4/15 | RR: 0.44 (0.09 - 2.08) | 149 fewer per 1000 (from 242 fewer to 288 more per 1000)  Moderate magnitude in favour (large in favour to large against) | Low |  |
| **Stool consistency - Number of patients with Bristol Stool Form Scale higher than 1 or 2 (hard stools) (time of measurement: 8 weeks)** | | | | | | | | | | | | |
| 1 | randomised trials | Not serious | Not serious | Not serious | Very serious^1^ | Not serious | 13/17 | 13/15 | RR: 0.88 (0.63 - 1.23) | 104 fewer per 1000 (from 321 fewer to 199 more per 1000)  Small magnitude against (large against to moderate in favour) | Low |  |
| **Fecal incontinence: number of patients with > 1 episode of fecal incontinence per week (time of measurement: 8 weeks) – incompatible outcome, do not use** | | | | | | | | | | | | |
| 1 | randomised trials | Not serious | Not serious | Not serious | Very serious^1^ | Not serious | 5/17 | 5/15 | RR: 0.88 (0.32 - 2.46) | 40 fewer per 1000 (from 227 fewer to 487 more per 1000)  Trivial in favour (large in favour to large against) | Low |  |
| **Abdominal pain – number of patients (time of measurement: 8 weeks) – incompatible outcome, do not use** | | | | | | | | | | | | |
| 1 | randomised trials | Not serious | Not serious | Not serious | Very serious^1^ | Not serious | 5/17 | 2/15 | RR: 2.71 (0.44 - 16.68) | 228 more per 1000 (from 75 fewer to 1000 more per 1000)  Large magnitude against (small in favour to large against) | Low |  |
| **Adverse events (time of measurement: 8 weeks)** | | | | | | | | | | | | |
| 1 | randomised trials | Not serious | Not serious | Not serious | Very serious^1^ |  | 0/16 | 0/17 | Not estimable | Not estimable | Low |  |

^1^Downgraded two levels due to very serious imprecision

*Forest plot Sodium picosulfate vs fibers Defecation frequency*

3. Sodium picosulfate + fiber vs fiber (N=1)

**Notes:** Cassettari 2019 compared sodium picosulfate with green banana biomass. Green (unripe) banana contains a high amount of fiber and a high concentration of amylase-resistant starch, which is not digested or absorbed in the intestine. **Question:** Should sodium picosulfate as addition to fiber vs fiber alone be used for treatment of functional constipation?
**Bibliography:** Cassettari 2019

GRADE

| **Quality assessment** | | | | | | | **No of patients** | | **Effect** | | **Certainty** | **Importance** |
| --- | --- | --- | --- | --- | --- | --- | --- | --- | --- | --- | --- | --- |
|  |  |  |  |  |  |  |  |  |  |  |  |  |
| **No of studies** | **Design** | **Risk of bias** | **Inconsistency** | **Indirectness** | **Imprecision** | **Other considerations** | **Sodium picosulfate + fiber** | **fiber** | **Relative (95% CI)** | **Absolute** |  |  |
| **Treatment success – not reported** | | | | | | | | | | | | |
|  |  |  |  |  |  |  |  |  |  |  |  |  |
| **Withdrawals due to Adverse Events – not reported** | | | | | | | | | | | | |
|  |  |  |  |  |  |  |  |  |  |  |  |  |
| **Defecation frequency - Dichotomous: number of patients having more than 3 bowel motions per week** | | | | | | | | | | | | |
| 1 | randomised trials | Not serious | Not serious | Not serious | Very serious^1^ | Not serious | 15/16 | 9/15 | RR: 1.56 (1.01 – 2.41)  NNT: 3 (1 to 167) | 336 more per 1000 (from 6 more to 847 more)  Large magnitude in favour (trivial in favour to large in favour) | Low |  |
| **Painful defecation – number of patients (time of measurement: 8 weeks) – incompatible outcome, do not use** | | | | | | | | | | | | |
| 1 | randomised trials | Not serious | Not serious | Not serious | Very serious^1^ | Not serious | 1/16 | 4/15 | RR: 0.23 (0.03 - 1.87) | 205 fewer per 1000 (from 259 fewer to 232 more per 1000)  Large magnitude in favour (large in favour to large against) | Low |  |
| **Stool consistency - Number of patients with Bristol Stool Form higher than Scale 1 or 2 (time of measurement: 8 weeks)** | | | | | | | | | | | | |
| 1 | randomised trials | Not serious | Not serious | Not serious | Very serious^1^ | Not serious | 13/16 | 13/15 | RR: 0.94 (0.69 - 1.28) | 52 fewer per 1000 (from 269 fewer 243 more per 1000)  Trivial magnitude against (large against to large in favour) | Low |  |
| **Fecal incontinence: number of patients with > 1 episode of fecal incontinence per week (time of measurement: 8 weeks) – incompatible outcome, do not use** | | | | | | | | | | | | |
| 1 | randomised trials | Not serious | Not serious | Not serious | Very serious^1^ | Not serious | 2/16 | 5/15 | RR: 0.38 (0.09 - 1.65) | 207 fewer per 1000 (from 303 fewer to 217 more per 1000)  Moderate magnitude in favour (large in favour to large against) | Low |  |
| **Abdominal pain – number of patients (time of measurement: 8 weeks) – incompatible outcome, do not use** | | | | | | | | | | | | |
| 1 | randomised trials | Not serious | Not serious | Not serious | Very serious^1^ | Not serious | 4/16 | 2/15 | RR: 1.88 (0.40 - 8.78) | 117 more per 1000 (from 80 fewer to 1000 more per 1000)  Small magnitude against (small against to large in favour) | Low |  |
| **Adverse events (time of measurement: 8 weeks)** | | | | | | | | | | | | |
| 1 | randomised trials | Not serious | Not serious | Not serious | Very serious^1^ | Not serious | 0/16 | 0/17 | Not estimable | Not estimable | Low |  |

^1^Downgraded two levels due to very serious imprecision

*Forest plot Sodium picosulfate + fiber vs fibers Defecation frequency*

Liquid paraffin

1. Liquid paraffin vs herbal medicine (N=1)

**Note:** herbal medicine consisted of cassia fistula emulsion.  **Question:** Should liquid paraffin vs herbal medicine be used as treatment of functional constipation?
**Bibliography:** Mozaffarpur 2012

GRADE

| **Quality assessment** | | | | | | | **No of patients** | | **Effect** | | **Certainty** | **Importance** |
| --- | --- | --- | --- | --- | --- | --- | --- | --- | --- | --- | --- | --- |
|  |  |  |  |  |  |  |  |  |  |  |  |  |
| **No of studies** | **Design** | **Risk of bias** | **Inconsistency** | **Indirectness** | **Imprecision** | **Other considerations** | **Liquid paraffin** | **Herbal medicine** | **Relative (95% CI)** | **Absolute** |  |  |
| **Treatment success (time of measurement: 3 weeks)** | | | | | | | | | | | | |
| 1 | Randomised trials | Very serious^1^ | Not serious | Not serious | Very serious^3^ | Not serious | 17/40 | 31/41 | RR: 0.56 (0.38 to 0.84) | 333 less per 1000 (from 121 less to 469 less per 100)  Large magnitude against (small against to large in favour) | Very low |  |
| **Withdrawals due to Adverse Events (time of measurement: 3 weeks)** | | | | | | | | | | | | |
| 1 | Randomised trials | Very serious^1^ | Not serious | Not serious | Very serious^3^ | Not serious | 1/40 | 0/41 | RR: 3.07 (0.13 to 73.28) | 52 more per 1000 (from 22 less to 1000 more per 1000)^4^  Large magnitude against (moderate in favour to large against) | Very low |  |
| **Defecation frequency per week (time of measurement: 3 weeks)** | | | | | | | | | | | | |
| 1 | Randomised trials | Very serious^1^ | Not serious | Not serious | Very serious^3^ | Not serious | N = 34 | N = 37 |  | MD: 4.50 stools per week less (6.88 stools per week less to 2.12 stools per week more)  Large magnitude against (large against to small in favour) | Very low |  |
| **Painful defecation: severity of pain on VAS 0-100 (time of measurement: 3 weeks)** | | | | | | | | | | | | |
| 1 | Randomised trials | Very serious^1^ | Not serious | Not serious | Very serious^3^ | Not serious | N = 34 | N = 37 |  | MD: 15.30 more pain during defecation on a scale of 0-100 (from 8.07 more pain to 22.53 more pain during defecation on a scale of 0-100)  Small magnitude against (trivial against to moderate in favour) | Very low |  |
| **Stool consistency: VAS score (0-100), 0=soft stools (time of measurement: 3 weeks)** | | | | | | | | | | | | |
| 1 | Randomised trials | Very serious^1^ | Not serious | Not serious | Serious^2^ | Not serious | N = 34 | N = 37 |  | MD: 13.50 harder stools on a scale of 0-100 (from 4.34 harder stools to 22.66 harder stools on a scale of 0-100)  Small magnitude against (trivial against to moderate in against) | Very low |  |
| **Fecal incontinence frequency per week (time of measurement: 3 weeks)** | | | | | | | | | | | | |
| 1 | Randomised trials | Very serious^1^ | Not serious | Not serious | Not serious | Not serious | N = 34 | N = 37 |  | MD: 0.20 more number of times fecal incontinence per week (from 0.45 less number of times fecal incontinence to 0.85 number of times more fecal incontinence per week)  Trivial magnitude against (trivial in favour to trivial against) | Low |  |
| **Serious adverse events (time of measurement: 3 weeks)** | | | | | | | | | | | | |
| 1 | Randomised trials | Very serious^1^ | Not serious | Not serious | Very serious^3^ | Not serious | 0/40 | 0/41 | Not estimable | Not estimable | Very low |  |

^1^Downgraded two levels due to lack of blinding and unclear randomization and allocation concealment ^2^Downgraded one level due to significant imprecision
^3^Downgraded two levels due to very sparse data and significant imprecision
^4^In order to interpret the results and calculate the absolute numbers a hypothetical event was added to the control group (1/41)

*Forest plot Liquid paraffin vs herbal medicine Treatment success*

*Forest plot Liquid paraffin vs herbal medicine Withdrawals due to AE*

*Forest plot Liquid paraffin vs herbal medicine Defecation frequency*

2. Liquid paraffin vs synbiotics (N=1)

**Note:** Synbiotics consisted of 1x10^9 CFU multispecies probiotics (L. casei, L. rhamnosus, S. thermophilus, B. breve, L. acidophilus, B. infantis) and fructo-oligosaccharides. **Question:** Should liquid paraffin vs synbiotics be used as treatment of functional constipation? **Bibliography:** Khodadad 2010

GRADE

| **Quality assessment** | | | | | | | **No of patients** | | **Effect** | | **Certainty** | **Importance** |
| --- | --- | --- | --- | --- | --- | --- | --- | --- | --- | --- | --- | --- |
|  |  |  |  |  |  |  |  |  |  |  |  |  |
| **No of studies** | **Design** | **Risk of bias** | **Inconsistency** | **Indirectness** | **Imprecision** | **Other considerations** | **Liquid paraffin** | **Synbiotics** | **Relative (95% CI)** | **Absolute** |  |  |
| **Treatment success (time of measurement: 4 weeks)** | | | | | | | | | | | | |
| 1 | Randomised trials | Very serious^1^ | Not serious | Not serious | Very serious^3^ | Not serious | 24/29 | 22/31 | RR: 1.17 (0.88 - 1.54) | 121 more per 1000 (from 85 less to 383 more per 1000)  Small magnitude in favour (small against to large in favour) | Very low |  |
| **Withdrawals due to adverse events (time of measurement: 4 weeks)** | | | | | | | | | | | | |
| 1 | Randomised trials | Very serious^1^ | Not serious | Not serious | Serious^2^ | Not serious | 0/29 | 0/31 | Not estimable | Not estimable | Very low |  |
| **Defecation frequency per week (time of measurement: 4 weeks)** | | | | | | | | | | | | |
| 1 | Randomised trials | Very serious^1^ | Not serious | Not serious | Serious^2^ | Not serious | N = 29 | N = 31 |  | MD: 1.53 higher (from 0.06 higher to 3.00 higher)  Small magnitude in favour (trivial in favour to moderate in favour) | Very low |  |
| **Painful defecation – number of patients (time of measurement: 4 weeks) – incompatible outcome** | | | | | | | | | | | | |
| 1 | Randomised trials | Very serious^1^ | Not serious | Not serious | Very serious^3^ | Not serious | 2/29 | 3/31 | RR: 0.71 (0.13 - 3.96) | 28 less per 1000 (from 84 less to 287 more per 1000)  Trivial magnitude in favour (small in favour to large against) | Very low |  |
| **Stool consistency: Number of patients with hard stools (time of measurement: 4 weeks) – incompatible outcome** | | | | | | | | | | | | |
| 1 | Randomised trials | Very serious^1^ | Not serious | Not serious | Serious | Not serious | 2/29 | 7/31 | RR: 0.31 (0.07 - 1.35) | 156 less per 1000 (from 210 less to 79 more per 1000) | Very low |  |
| **Fecal incontinence frequency per week (time of measurement: 4 weeks)** | | | | | | | | | | | | |
| 1 | Randomised trials | Very serious^1^ | Not serious | Not serious | Not serious | Not serious | N = 29 | N = 31 |  | MD: 0.18 higher (from 0.30 less to 0.66 more)  Trivial magnitude against (trivial in favour to trivial against) | Low |  |
| **Abdominal pain- number of patients (time of measurement: 4 weeks) – incompatible outcome** | | | | | | | | | | | | |
| 1 | Randomised trials | Very serious^1^ | Not serious | Not serious | Serious^2^ | Not serious | 4/29 | 2/31 | RR: 2.14 (0.42 - 10.80) | 74 more per 1000 (from 37 less to 632 more per 1000) | Very low |  |

^1^Downdgraded two levels due to unclear method of randomization, allocation and blinding
^2^Downgraded one level due to significant imprecision
^3^Downgraded two levels due to very serious imprecision

*Forest plot Liquid paraffin vs synbiotics Treatment success*

*Forest plot Liquid paraffin vs synbiotics Withdrawals due to AE*

*Forest plot Liquid paraffin vs synbiotics Defecation frequency*

Prucalopride

1. Prucalopride vs placebo (N=1)

**Question:** Should prucalopride vs placebo be used as treatment for functional constipation?
**Bibliography:** Mugie 2014

GRADE

| **Quality assessment** | | | | | | | **No of patients** | | **Effect** | | **Certainty** | **Importance** |
| --- | --- | --- | --- | --- | --- | --- | --- | --- | --- | --- | --- | --- |
|  |  |  |  |  |  |  |  |  |  |  |  |  |
| **No of studies** | **Design** | **Risk of bias** | **Inconsistency** | **Indirectness** | **Imprecision** | **Other considerations** | **Prucalopride** | **Placebo** | **Relative (95% CI)** | **Absolute** |  |  |
| **Treatment success (time of measurement: 8 weeks)** | | | | | | | | | | | | |
| 1 | Randomised trials | Not serious | Not serious | Not serious | Very serious^1^ | Not serious | 15/107 | 9/108 | RR: 1.68 (0.77 – 3.68) | 57 more per 1000 (19 fewer to 223 more)  Trivial magnitude in favour (trivial against to moderate in favour) | Low |  |
| **Withdrawals due to Adverse Events (time of measurement: 8 weeks)** | | | | | | | | | | | | |
| 1 | Randomised trials | Not serious | Not serious | Not serious | Very serious^1^ | Not serious | 8/107 | 5/108 | RR: 1.61 (0.55 – 4.78) | 28 more per 1000 (21 fewer to 175 more)  Large magnitude against (moderate in favour to large against) | Low |  |
| **Defecation frequency per week (time of measurement: 8 weeks)** | | | | | | | | | | | | |
| 1 | Randomised trials | Not serious | Not serious | Not serious | Serious^2^ | Not serious | N = 106 | N = 107 |  | MD: 0.50 more stools per week (from 0.06 less stools per week to 1.06 more stools per week)  Trivial magnitude in favour (trivial against to small in favour) | Moderate |  |
| **Painful defecation, change from baseline (scale 0-5), time of measurement 8 weeks** | | | | | | | | | | | | |
| 1 | Randomised trials | Not serious | Not serious | Not serious | Not serious | Not serious | N = 106 | N = 107 |  | MD: 0.20 point less pain during defecation on a scale of 0-5 (from 0.51 point less pain to 0.11 point more pain during defecation on a scale of 0-5)  Trivial magnitude in favour (trivial in favour to trivial against) | High |  |
| **Stool consistency: Bristol Stool Form Scale (time of measurement: 8 weeks)** | | | | | | | | | | | | |
| 1 | Randomised trials | Not serious | Not serious | Not serious | Serious^2^ | Not serious | N = 106 | N = 107 |  | MD: 0.50 points softer stools on a scale of 0-7 (from 0.15 point softer stools to 0.85 softer stools on a scale of 0-7)  Trivial magnitude in favour (trivial in favour to small in favour) | Moderate |  |
| **Fecal incontinence frequency per two weeks (time of measurement: 8 weeks)** | | | | | | | | | | | | |
| 1 | Randomised trials | Not serious | Not serious | Not serious | Very serious^1^ | Not serious | N = 106 | N = 107 |  | MD: 5.20 less number of times fecal incontinence per 2 weeks (from 19.36 less to 8.96 more number of times fecal incontinence per 2 weeks)  Moderate magnitude in favour (large in favour to large against) | Low |  |
| **Abdominal pain (scale 0-5) (time of measurement: 8 weeks)** | | | | | | | | | | | | |
| 1 | Randomised trials | Not serious | Not serious | Not serious | Not serious | Not serious | N = 106 | N = 107 |  | MD: 0.10 points less abdominal pain on a scale of 0-5 (from 0.33 points less to 0.13 point more abdominal pain on a scale of 0-5)  Trivial magnitude in favour (trivial in favour to trivial against) | High |  |
| **Serious adverse events (time of measurement: 8 weeks)** | | | | | | | | | | | | |
| 1 | Randomised trials | Not serious | Not serious | Not serious | Very serious^1^ | Not serious | 5/106 | 2/107 | RR: 2.52 (0.50 - 12.72) | 28 more per 1000 (from 9 less to 219 more per 1000)  Large magnitude against (small in favour to large against) | Low |  |
| **Adverse events (time of measurement: 8 weeks)** | | | | | | | | | | | | |
| 1 | Randomised trials | Not serious | Not serious | Not serious | Very serious^1^ | Not serious | 101/106 | 72/107 | RR: 1.42 (1.23 - 1.63) | 283 more per 1000 (from 155 more to 424 more per 1000)  Large magnitude against (large against to large in favour) | Low |  |

^1^Downdgraded two levels due to very serious imprecision
^2^Downgraded one level due to significant imprecision

*Forest plot Prucalopride vs placebo Treatment success*

*Forest plot Prucalopride vs placebo Withdrawals due to AE*

*Forest plot Prucalopride vs placebo Defecation frequency*

Lubiprostone

1. Lubiprostone vs placebo (N=1)

**Question:** Should lubiprostone vs placebo be used as treatment of functional constipation in children?
**Bibliography:** Benninga 2022

GRADE

| **Quality assessment** | | | | | | | **No of patients** | | **Effect** | | **Certainty** | **Importance** |
| --- | --- | --- | --- | --- | --- | --- | --- | --- | --- | --- | --- | --- |
|  |  |  |  |  |  |  |  |  |  |  |  |  |
| **No of studies** | **Design** | **Risk of bias** | **Inconsistency** | **Indirectness** | **Imprecision** | **Other considerations** | **Lubiprostone** | **Placebo** | **Relative (95% CI)** | **Absolute** |  |  |
| **Treatment success^1^ (time of measurement: during 12 week treatment)** | | | | | | | | | | | | |
| 1 | Randomised trials | Not serious | Not serious | Not serious | Very serious^2^ | Not serious | 74/404 | 28/202 | RR: 1.32 (0.89 to 1.97) | 44 more per 1000 (from 15 fewer to 134 more per 1000)  Trivial magnitude in favour (trivial against to small in favour) | low |  |
| **Withdrawals due to Adverse Events (measured at 12 weeks)** | | | | | | | | | | | | |
| 1 | Randomised trials | Not serious | Not serious | Not serious | Very serious^2^ | Not serious | 99/404 | 48/202 | RR: 1.03 (0.76 to 1.39) | 7 more per 1000 (from 57 less to 93 more per 1000)  Trivial magnitude against (large in favour to large against) | Low |  |
| **Defecation frequency – reported as treatment success** | | | | | | | | | | | | |
|  |  |  |  |  |  |  |  |  |  |  |  |  |
| **Painful defecation (scale 1-4) (measured at 12 weeks)** | | | | | | | | | | | | |
| 1 | Randomised trials | Not serious | Not serious | Not serious | Serious^3^ | Not serious | N=395 | N=199 |  | MD: 0.21 higher on a scale of 1-4 for pain during defecation (from 0.01 lower to 0.43 higher on a scale of 1-4 for pain during defecation)  Trivial magnitude against (trivial in favour to small against) | Moderate |  |
| **Fecal incontinence frequency per two weeks (measured at 12 weeks)** | | | | | | | | | | | | |
| 1 | Randomised trials | Not serious | Not serious | Not serious | Not serious | Not serious | N=395 | N=199 |  | MD: 0.03 number of times less fecal incontinence per 2 weeks (from 0.11 number of times less to 0.05 number of times more fecal incontinence per 2 weeks)  Trivial magnitude in favour (trivial in favour to trivial against) | High |  |
| **Abdominal pain (scale 1-4) (measured at 12 weeks)** | | | | | | | | | | | | |
| 1 | Randomised trials | Not serious | Not serious | Not serious | Not serious | Not serious | N=395 | N=199 |  | MD: 0.07 points more abdominal pain on a scale of 1-4 (from 0.06 points less to 0.20 points more abdominal pain on a scale of 1-4)  Trivial magnitude against (trivial in favour to trivial against) | High |  |
| **Serious adverse events** | | | | | | | | | | | | |
| 1 | Randomised trials | Not serious | Not serious | Not serious | Very serious^2^ | Not serious | 11/400 | 7/195 | RR: 0.77 (0.30 - 1.95) | 8 less per 1000 (from 25 less to 34 more per 1000)  Small magnitude in favour (large in favour to large against) | Low |  |
| **Adverse events** | | | | | | | | | | | | |
| 1 | Randomised trials | Not serious | Not serious | Not serious | Very serious^2^ | Not serious | 239/400 | 114/195 | RR: 1.02 (0.89 to 1.18) | 12 more per 1000 (from 64 less to 105 more per 1000)  Trivial magnitude against (small in favour to moderate against) | Low |  |

^1^Defined as: overall Spontaneous Bowel Movement (SBM) response, defined as an increase of 1 or more SBM/wk compared with baseline and 3 or more SBMs/wk for at least 9 weeks, including 3 of the final 4 treatment weeks. ^2^Downgraded two levels due to very serious imprecision
^3^Downgraded one level due to serious imprecision

*Forest plot Lubiprostone vs placebo Treatment success*

*Forest plot Lubiprostone vs placebo Withdrawals due to AE*

2. Lubiprostone vs conventional treatment (N=1)

**Notes:** Conventional treatment consisted of treatment with either lactulose, bisacodyl or sodium picosulfate.  **Question:** Should lubiprostone vs conventional treatment be used as treatment of functional constipation in children? **Bibliography:** Elkaragy 2024

GRADE

| **Quality assessment** | | | | | | | **No of patients** | | **Effect** | | **Certainty** | **Importance** |
| --- | --- | --- | --- | --- | --- | --- | --- | --- | --- | --- | --- | --- |
|  |  |  |  |  |  |  |  |  |  |  |  |  |
| **No of studies** | **Design** | **Risk of bias** | **Inconsistency** | **Indirectness** | **Imprecision** | **Other considerations** | **Lubiprostone** | **Conventional treatment** | **Relative (95% CI)** | **Absolute** |  |  |
| **Treatment success^1^ (time of measurement: 16 weeks)** | | | | | | | | | | | | |
| 1 | Randomised trials | Very serious^2^ | Not serious | Not serious | Not serious | Not serious | 128/140 | 40/140 | RR: 3.20 (2.45 to 4.18)  NNT: 2 (1 – 2) | 629 more per 1000 (from 414 more to 909 more per 1000)  Large magnitude in favour (large in favour to large in favour) | Low |  |
| **Withdrawals due to Adverse Events (time of measurement: 16 weeks)** | | | | | | | | | | | | |
| 1 | Randomised trials | Very serious^2^ | Not serious | Not serious | Very serious | Not serious | 5/140 | 4/140 | RR: 1.25 (0.34 to 4.56) | 7 more per 1000 (from 19 less to 102 more per 1000)  Small magnitude of effect against (moderate in favour to large against) | Very low |  |
| **Defecation frequency per week (time of measurement: 16 weeks)** | | | | | | | | | | | | |
| 1 | Randomised trials | Very serious^2^ | Not serious | Not serious | Not serious | Not serious | N = 140 | N = 140 |  | MD: 2.46 more stools per week (from 2.20 more to 2.72 more stools per week)  Moderate magnitude of effect in favour (small magnitude in favour to moderate magnitude in favour) | Low |  |
| **Stool consistency – Bristol Stool Form score of 3 or 4 during the last 4 weeks of therapy and through the 4 weeks of follow-up (time of measurement: 16 weeks)** | | | | | | | | | | | | |
| 1 | Randomised trials | Very serious^2^ | Not serious | Not serious | Not serious | Not serious | 106/140 | 50/140 | RR: 2.12 (1.67 to 2.70) | 400 more per 1000 (from 239 more to 607 more per 1000)  Large magnitude of effect in favour (large in favour to large in favour) | Low |  |
| **Serious adverse events** | | | | | | | | | | | | |
| 1 | Randomised trials | Very serious^2^ | Not serious | Not serious | Very serious | Not serious | 0/135 | 0/136 | Not estimable | Not estimable | Very low |  |
| **Adverse events** | | | | | | | | | | | | |
| 1 | Randomised trials | Very serious^2^ | Not serious | Not serious | Very serious | Not serious | 62/135 | 65/136 | RR: 0.96 (0.75 to 1.24) | 19 less per 1000 (from 119 less to 115 more per 1000)  Trivial magnitude of effect in favour (moderate in favour to moderate against) | Very low |  |

^1^Defined as: Improvement of constipation manifested as improved SBM defined as having ≥1 SBM/week increase in frequency compared with the baseline and maintaining ≥3 SBMs/week for at least 8 weeks, including the last four study weeks and 4 weeks of follow-up
^2^Downgraded two levels due to high risk of blinding, only one study included in the analysis and the study could have been blinded.

*Forest plot Lubiprostone vs conventional treatment Treatment success*

*Forest plot Lubiprostone vs conventional treatment Withdrawals due to adverse events*

*Forest plot Lubiprostone vs conventional treatment defecation frequency*

Linaclotide

1. Linaclotide vs placebo (N=3)

**Notes:** Di Lorenzo 2024b included children aged 2 – 5 years. **Question:** Should linaclotide vs placebo be used for treatment of functional constipation?
**Bibliography:** Lorenzo 2020 (abstract only)^1^, Di Lorenzo 2024, Di Lorenzo 2024b^5^

GRADE

| **Quality assessment** | | | | | | | **No of patients** | | **Effect** | | **Certainty** | **Importance** |
| --- | --- | --- | --- | --- | --- | --- | --- | --- | --- | --- | --- | --- |
|  |  |  |  |  |  |  |  |  |  |  |  |  |
| **No of studies** | **Design** | **Risk of bias** | **Inconsistency** | **Indirectness** | **Imprecision** | **Other considerations** | **Linaclotide** | **placebo** | **Relative (95% CI)** | **Absolute** |  |  |
| **Treatment success (time of measurement: 4 weeks to 12 weeks)** | | | | | | | | | | | | |
| 2 | Randomised trials | Not serious | Not serious | Not serious | Very serious^2^ | Not serious | 27/177 | 21/172 | RR: 1.21 (0.69 to 2.13) | 25 more per 1000 (from 38 less to 138 more per 1000)  Trivial magnitude in favour (trivial against to moderate in favour) | Low |  |
| **Withdrawals due to Adverse Events (time of measurement range: 4 weeks to 12 weeks)** | | | | | | | | | | | | |
| 3 | Randomised trials | Not serious | Not serious | Not serious | Very serious^3^ | Not serious | 14/216 | 18/213 | RR: 0.78 (0.40 to 1.52) | 19 less per 1000 (from 53 less to 46 more per 1000)  Moderate magnitude in favour (large in favour to large against) | Low |  |
| **Defecation frequency per week (time of measurement range: 4 to 12 weeks)** | | | | | | | | | | | | |
| 3 | Randomised trials | Not serious | Not serious | Not serious | Serious^2^ | Not serious | N=216 | N=213 |  | MD: 1.17 higher (from 0.45 higher to 1.89 higher)  Small magnitude in favour (trivial in favour to small in favour) | Moderate |  |
| **Stool consistency on the BSFS (scale 1-7) (time of measurement range: 4 weeks to 12 weeks)** | | | | | | | | | | | | |
| 3 | Randomised trials | Not serious | Serious^4^ | Not serious | Serious^2^ | Not serious | N=198 | N=198 |  | MD: 0.23 higher (0.65 lower to 1.11 higher)  Trivial magnitude against (trivial against to small in favour) | Low |  |
| **Fecal incontinence - continuous: Change from baseline fecal incontinence daytime per day (time of measurement: 4 weeks)** | | | | | | | | | | | | |
| 1 | Randomised trials | Not serious | Not serious | Not serious | Serious^2^ | Not serious | N=10 | N=11 |  | MD: 0.14 lower (from 0.33 lower to 0.05 higher)  Small magnitude in favour (small in favour to trivial against) | Moderate |  |
| **Fecal incontinence – dichotomous: number of patients with fecal incontinence (time of measurement: 12 weeks) – incompatible outcome** | | | | | | | | | | | | |
| 1 | Randomised trials | Not serious | Not serious | Not serious | Serious^2^ | Not serious | 38/164 | 36/164 | RR: 1.06 (from 0.71 to 1.58) | 13 more per 1000 (from 64 less to 127 more per 1000) | Moderate |  |
| Fecal incontinence – continuous: proportion of days with fecal incontinence (time of measurement 4 weeks) – incompatible outcome | | | | | | | | | | | | |
| 1 | Randomised trials | Not serious | Not serious | Not serious | Very serious | Not serious | N=10 | N=6 |  | MD: not estimable | Very low |  |
| **Abdominal pain on** **scale of 0-4 0=none, 4=a lot (time of measurement range: 4 weeks to 12 weeks)** | | | | | | | | | | | | |
| 2 | Randomised trials | Not serious | Serious^4^ | Not serious | Not serious | Not serious | N=203 | N=205 |  | MD: 0.03 higher (from 0.46 lower to 0.52 higher)  Trivial magnitude against (trivial in favour to trivial against) | Low |  |
| **Serious adverse events (time of measurement range: 4 weeks to 12 weeks)** | | | | | | | | | | | | |
| 3 | Randomised trials | Not serious | Not serious | Not serious | Very serious^3^ | Not serious | 2/216 | 2/213 | RR: 1.00 (0.14 to 7.01) | 0 more per 1000 (from 8 less to 59 more per 1000)  Trivial magnitude (small in favour to large against) | Low |  |
| **Adverse events (time of measurement range: 4 weeks to 12 weeks)** | | | | | | | | | | | | |
| 3 | Randomised trials | Not serious | Not serious | Not serious | Very serious^3^ | Not serious | 45/216 | 31/213 | RR: 1.55 (0.84 to 2.84) | 80 more per 1000 (from 23 less to 268 more per 1000)  Moderate magnitude against (trivial in favour to large against) | Low |  |

^1^Study investigated different range of dosages for effectiveness and safety (phase 2 study). We only included data from high dose group (72 microgram), to combine data from Di Lorenzo 2024 paper which also used 72 microgram dosage.
^2^Downgraded one level due to significant imprecision
^3^Downgraded two levels due to serious imprecision
^4^Downgraded one level due to heterogeneity
^5^Study investigated different range of dosages for effectiveness and safety (phase 2 study). We only included data from high dose group (72 microgram), as the phase 3 trial is currently conducted for only 72 microgram

*Linaclotide vs placebo Treatment success*

*Linaclotide vs placebo Defecation frequency*

*Linaclotide vs placebo Withdrawals due to AE*

Enema

1. Enema + PEG vs PEG (N=1)

**Question:** Should enemas as addition to PEG vs PEG alone be used for the treatment of functional constipation?
**Bibliography:** Bongers 2009

GRADE

| **Quality assessment** | | | | | | | **No of patients** | | **Effect** | | **Quality** | **Importance** |
| --- | --- | --- | --- | --- | --- | --- | --- | --- | --- | --- | --- | --- |
|  |  |  |  |  |  |  |  |  |  |  |  |  |
| **No of studies** | **Design** | **Risk of bias** | **Inconsistency** | **Indirectness** | **Imprecision** | **Other considerations** | **Enema + PEG** | **PEG** | **Relative (95% CI)** | **Absolute** |  |  |
| **Treatment success (time of measurement: 52 weeks)** | | | | | | | | | | | | |
| 1 | Randomised trials | Not serious | Not serious | Not serious | Very serious^1^ | Not serious | 24/51 | 18/51 | RR: 1.33 (0.83 – 2.14) | 116 more per 1000 (60 fewer to 402 more)  Small magnitude in favour (trivial against to large in favour) | Low |  |
| **Withdrawals due to Adverse Events (time of measurement: 52 weeks)** | | | | | | | | | | | | |
| 1 | Randomised trials | Not serious | Not serious | Not serious | Very serious^1^ | Not serious | 4/51 | 0/51 | RR: 9.00 (0.50 - 162.97) | 157 more per 1000 (from 10 less to 1000 more per 1000)^2^  Large magnitude against (small in favour to large against) | Low |  |
| **Defecation frequency – no useful data (no SD reported)** | | | | | | | | | | | | |
|  |  |  |  |  |  |  |  |  |  |  |  |  |
| **Painful defecation – number of patients (time of measurement: 52 weeks) – incompatible outcome** | | | | | | | | | | | | |
| 1 | Randomised trials | Not serious | Not serious | Not serious | Serious^3^ | Not serious | 11/51 | 17/51 | RR: 0.65 (0.34 to 1.24) | 117 less per 1000 (from 220 less 80 more per 1000) | Moderate |  |
| **Abdominal pain – number of patients (time of measurement: 52 weeks) – incompatible outcome** | | | | | | | | | | | | |
| 1 | Randomised trials | Not serious | Not serious | Not serious | Serious^3^ | Not serious | 17/51 | 22/51 | RR: 0.77 (0.47 to 1.27) | 99 less per 1000 (229 less to 116 more per 1000) | Moderate |  |

^1^Downgraded two levels due to very serious imprecision ^2^Added one fictional case to the control group in RevMan to calculate absolute numbers in order to better interpret results
^3^Downgraded one level due to serious imprecision

*Forest plot Enema + PEG vs PEG Treatment success*

*Forest plot Enema + PEG vs PEG Withdrawals due to AE*

Other

1. PEG + Domperidone vs PEG + Placebo (N=1)

**Question:** Should domperidone as addition to PEG vs PEG only be used for treatment of functional constipation?
**Bibliography:** Dehghani 2014

*GRADE*

| **Quality assessment** | | | | | | | **No of patients** | | **Effect** | | **Quality** | **Importance** |
| --- | --- | --- | --- | --- | --- | --- | --- | --- | --- | --- | --- | --- |
|  |  |  |  |  |  |  |  |  |  |  |  |  |
| **No of studies** | **Design** | **Risk of bias** | **Inconsistency** | **Indirectness** | **Imprecision** | **Other considerations** | **PEG + Domperidone** | **PEG + placebo** | **Relative (95% CI)** | **Absolute** |  |  |
| **Treatment success (time of measurement: 6 months)** | | | | | | | | | | | | |
| 1 | Randomised trials | Serious^1^ | Not serious | Not serious | Very serious^2^ |  | 38/52 | 45/53 | RR: 0.86 (0.70 – 1.05) | 119 fewer per 1000 (254 fewer to 42 more)  Small magnitude against (large against to trivial in favour) | Very low |  |
| **Withdrawals due to Adverse Events (time of measurement: 6 months)** | | | | | | | | | | | | |
| 1 | Randomised trials | Serious^1^ | Not serious | Not serious | Very serious^2^ |  | 5/52 | 2/53 | RR: 2.55 (0.52 – 12.55) | 58 more per 1000 (18 fewer to 437 more)  Large magnitude against (moderate in favour to large against) | Very low |  |
| **Defecation frequency – dichotomous: number of patients that reported 3 or more episodes of defecation per week^3^** | | | | | | | | | | | | |
| 1 | randomised trials | Serious | Not serious | Not serious | Very serious^2^ |  | 47/52 | 44/53 | RR 1.09 (0.94 – 1.27) | 75 more per 1000 (50 less to 224 more)  Small magnitude in favour (trivial against to moderate in favour) | Very low |  |
| **Fecal incontinence: number of patients with ≥1 dirty underwear per week (time of measurement: 6 months)** | | | | | | | | | | | | |
| 1 | randomised trials | Serious^1^ | Not serious | Not serious | Very serious^2^ |  | 10/52 | 7/53 | RR: 1.46 (0.60 – 3.35) | 61 more per 1000 (from 53 less to 310 more per 1000)  Trivial magnitude against (trivial in favour to large against) | Very low |  |
| **Adverse events (time of measurement: 6 months)** | | | | | | | | | | | | |
| 1 | Randomised trials | Serious^1^ | Not serious | Not serious | Very serious^2^ |  | 0/52 | 0/52 | Not estimable | Not estimable | Very low |  |

^1^Downgraded one level due to high risk of selective reporting
^2^Downgraded two levels due to very serious imprecision
^3^Outcome in study was reported as number of patients that reported ≤2 episodes of defecation per week. To compare with other studies the data is reported in this table as the number of patients that reported 3 or more episodes of defecation per week.

*Forest plot PEG + Domperidone vs PEG + Placebo Treatment success*

*Forest plot PEG + Domperidone vs PEG + Placebo Withdrawals due to AE*

*Forest plot PEG + Domperidone vs PEG + Placebo Defecation frequency*

Non-pharmacological treatment

Probiotics

1. Probiotics vs placebo (N=8)

**Notes**: Zaja 2021 included only patients with anorexia nervosa. Wojtyniak 2017 included only children below 5 years of age. Lojanatorn 2023 included only children aged 1 – 5 years old.
Three studies investigated a mixture of different probiotics (Gan 2022, Tabbers 2011, Tjokronegoro 2020). Other studies investigated B. clausii (Lojanatorn 2023), L. Reuteri (Coccorullo 2010, Zaja 2021), and L. casei rhamnosus (Bu 2007, Wojtyniak 2017).
**Question:** Should probiotics vs placebo be used for functional constipation?
**Bibliography:** Lojanatorn 2023, Tabbers 2011, Tjokronegoro 2020, Zaja 2021, Bu 2007, Wojtyniak 2017, Gan 2022, Coccorullo 2010

GRADE

| **Quality assessment** | | | | | | | **No of patients** | | **Effect** | | **Certainty** | **Importance** |
| --- | --- | --- | --- | --- | --- | --- | --- | --- | --- | --- | --- | --- |
|  |  |  |  |  |  |  |  |  |  |  |  |  |
| **No of studies** | **Design** | **Risk of bias** | **Inconsistency** | **Indirectness** | **Imprecision** | **Other considerations** | **Probiotics** | **Placebo** | **Relative (95% CI)** | **Absolute** |  |  |
| **Treatment success (time of measurement range: 3 to 12 weeks)** | | | | | | | | | | | | |
| 6 | Randomised controlled trials | Serious^1^ | Serious^2^ | Not serious | Very serious^3^ | Not serious | 118/220 | 84/208 | RR: 1.29 (0.89 to 1.85) | 117 more per 1000 (from 44 less to 343 more per 1000)  Small magnitude in favour (trivial against to large in favour) | Very low |  |
| **Withdrawals due to Adverse Events at study end (range: 3 weeks to 6 months)** | | | | | | | | | | | | |
| 8 | Randomised controlled trials | Serious^1^ | Not serious | Not serious | Very serious^3^ | Not serious | 19/292 | 23/280 | RR: 0.79 (0.44 to 1.40) | 17 less per 1000 (from 46 less to 33 more per 1000)  Moderate magnitude in favour (large in favour to large against) | Very low |  |
| **Defecation frequency per week (time of measurement range: 3 weeks to 6 months)** | | | | | | | | | | | | |
| 5 | Randomised controlled trials | Serious^1^ | Serious^2^ | Not serious | Serious^4^ | Not serious | N=192 | N=180 |  | MD: 0.32 stools more per week (from 1.12 stools less per week to 1.76 stools more per week)  Trivial magnitude in favour (trivial against to small in favour) | Very low |  |
| **Painful defecation frequency per week (time of measurement range: 4 weeks)** | | | | | | | | | | | | |
| 2 | Randomised controlled trials | Serious^1^ | Not serious | Not serious | Not serious | Not serious | N=61 | N=60 |  | MD: 0.05 higher frequency per week (from 0.25 lower to 0.35 higher)  Trivial magnitude against (trivial in favour to trivial against) | Moderate |  |
| **Painful defecation – dichotomous: number of patients with painful defecation (time of measurement range: 3 to 4 weeks) – incompatible outcome** | | | | | | | | | | | | |
| 2 | Randomised controlled trials | Not serious | Serious^2^ | Not serious | Very serious^3^ | Not serious | 44/118 | 50/119 | RR: 0.74 (0.27 to 2.02) | 109 less per 1000 (from 307 less to 429 more per 1000) | Very low |  |
| **Stool consistency – mean score on Bristol Stool form Scale (1= very hard, 7=very loose) (time of measurement: 4 weeks)** | | | | | | | | | | | | |
| 2 | Randomised controlled trials | Serious^1^ | Not serious | Not serious | Not serious | Not serious | N=61 | N=58 |  | MD: 0.16 lower (from 0.49 lower to 0.16 higher)  Trivial magnitude against (trivial against to trivial in favour) | Moderate |  |
| **Stool consistency – dichotomous: number of patients with normal stool consistency (time of measurement range: 1 to 6 months)** | | | | | | | | | | | | |
| 2 | Randomised controlled trials | Not serious | Serious^2^ | Not serious | Very serious^3^ | Not serious | 39/54 | 31/55 | RR: 1.19 (0.64 to 2.20) | 107 more per 1000 (from 203 less to 677 more per 1000)  Small magnitude in favour (moderate against to large in favour) | Very low |  |
| **Fecal incontinence – dichotomous: number of patients with fecal incontinence episodes (time of measurement range: 3 to 4 weeks) – incompatible outcome** | | | | | | | | | | | | |
| 2 | Randomised controlled trials | Not serious | Not serious | Not serious | Very serious^3^ | Not serious | 29/118 | 43/119 | RR: 0.61 (0.27 to 1.38) | 141 less per 1000 (from 264 less to 137 more per 1000) | Low |  |
| **Fecal incontinence frequency per week (time of measurement: 4 weeks)** | | | | | | | | | | | | |
| 1 | Randomised controlled trials | Not serious | Not serious | Not serious | Very serious^3^ | Not serious | N=41 | N=40 |  | MD: not estimable | Low |  |
| **Abdominal pain – number of patients with abdominal pain (time of measurements: 3 weeks) – incompatible outcome** | | | | | | | | | | | | |
| 1 | Randomised controlled trials | Not serious | Not serious | Not serious | Very serious^3^ | Not serious | 43/79 | 40/80 | RR: 1.09 (0.81 to 1.47) | 45 more per 1000 (from 95 less to 235 more per 1000) | Low |  |
| **Serious adverse events (time of measurements range: 3 weeks to 6 months)** | | | | | | | | | | | | |
| 4 | Randomised controlled trials | Not serious | Not serious | Not serious | Very serious^3^ | Not serious | 0/123 | 0/119 | Not estimable | Not estimable | Low |  |
| **Adverse events (time of measurements range: 3 weeks to 6 months)** | | | | | | | | | | | | |
| 5 | Randomised controlled trials | Serious^1^ | Not serious | Not serious | Serious^4^ | Not serious | 9/202 | 16/199 | RR: 0.59 (0.24 to 1.44) | 33 less per 1000 (61 less to 35 more per 1000)  Trivial magnitude in favour (small in favour to trivial against) | low |  |

^1^Downgraded one level due to unclear selective reporting and unclear allocation concealment in several studies and high risk of performance and assessment bias in one study.
^2^Downgraded one level due to serious heterogeneity
^3^Downgraded two levels due to very serious imprecision
^4^Downgraded one level due to significant imprecision

*Forest plot probiotics vs placebo treatment success*

*Forest plot probiotics vs placebo withdrawals due to AE*

*Forest plot probiotics vs placebo defecation frequency*

*Forest plot probiotics vs placebo stool consistency – continuous*

 *Forest plot probiotics vs placebo stool consistency – dichotomous*

2. Probiotics vs laxatives (N=3)

**Notes**: Did not use data from Kubota 2020 because def frequency was reported as “Change from baseline in Bowel movements per week, least square mean (95% CI)”, saw that in probiotics SR Kubota was also not included in meta-analysis.
Number of withdrawals was very high in Lee 2022 (IG: 22 lost to follow-up, 23 drug change (could be due to poor treatment outcome, poor compliance, and/or other side effects), CG: 44 lost to follow-up, 3 drug change).
Bu 2007 compared L. casei rhamnosus lcr35 to magnesiumoxide. Lee 2022 compared S. boulardii to lactulose. Olgaç 2013 compared L. reuteri to lactulose.
**Question:** Should probiotics vs laxative be used for functional constipation?
**Bibliography:** Bu 2007, Lee 2022, Olgaç 2013

GRADE

| **Quality assessment** | | | | | | | **No of patients** | | **Effect** | | **Certainty** | **Importance** |
| --- | --- | --- | --- | --- | --- | --- | --- | --- | --- | --- | --- | --- |
|  |  |  |  |  |  |  |  |  |  |  |  |  |
| **No of studies** | **Design** | **Risk of bias** | **Inconsistency** | **Indirectness** | **Imprecision** | **Other considerations** | **Probiotics** | **Laxative** | **Relative (95% CI)** | **Absolute** |  |  |
| **Treatment success (time of measurement range: 4 to 12 weeks)** | | | | | | | | | | | | |
| 2 | Randomised controlled trials | Very serious^1^ | Very serious | Not serious | Very serious^2^ | Not serious | 18/68 | 31/87 | RR: 0.61 (0.12 to 2.96) | 139 less per 1000 (from 314 less to 698 more per 1000)  Moderate magnitude against (large against to large in favour) | Very low |  |
| **Withdrawals due to Adverse Events at study end (range: 4 to 12 weeks)** | | | | | | | | | | | | |
| 3 | Randomised controlled trials | Very serious^1^ | Not serious | Not serious | Very serious^2^ | Not serious | 51/93 | 55/115 | RR: 1.30 (1.08 to 1.56) | 143 more per 1000 (38 more 268 more per 1000)  Large magnitude against (large against to large against) | Very low |  |
| **Defecation frequency per week (time of measurement range: 2 to 4 weeks)** | | | | | | | | | | | | |
| 3 | Randomised controlled trials | Very serious^1^ | Not serious | Not serious | Not serious | Not serious | N=82 | N=106 |  | MD: 0.36 stools more per week (0.15 stools more per week to 0.57 stools more per week)  Trivial magnitude in favour (trivial in favour to trivial in favour) | Low |  |
| **Painful defecation – frequency per week (Lee 2022) (time of measurement: 2 weeks)** | | | | | | | | | | | | |
| 1 | Randomised controlled trials | Very serious^1^ | Not serious | Not serious | Not serious | Not serious | N=39 | N=60 |  | MD: 0.20 higher (from 0.07 lower to 0.47 higher)  Trivial magnitude against (trivial in favour to trivial against) | Low |  |
| **Stool consistency: BSFS (time of measurement range: 2 to 4 weeks)** | | | | | | | | | | | | |
| 2 | Randomised controlled trials | Very serious^1^ | Serious^3^ | Not serious | Not serious | Not serious | N=62 | N=88 |  | MD: 0.17 lower (from 0.61 lower to 0.26 higher)  Trivial magnitude against (trivial against to trivial in favour) | Very low |  |
| **Stool consistency: percentage of hard stools (time of measurement: 4 weeks) – incompatible outcome** | | | | | | | | | | | | |
| 1 | Randomised controlled trials | Very serious | Not serious | Not serious | Not serious | Not serious | N=18 | N=18 |  | MD: 1.10 lower (from 8.81 lower to 6.61 higher) | Low |  |
| **Fecal incontinence frequency per week (time of measurement: 2 weeks)** | | | | | | | | | | | | |
| 1 | Randomised controlled trials | Very serious^1^ | Not serious | Not serious | Serious^4^ | Not serious | N= 37 | N=60 |  | MD: 0.43 higher (from 0.82 lower to 1.68 higher)  Trivial magnitude against (trivial in favour to small against) | Very low |  |
| **Adverse events (Kubota, olgac) (time of measurements range: 4 to 12 weeks)** | | | | | | | | | | | | |
| 2 | Randomised controlled trials | Very serious^1^ | Not serious | Not serious | Very serious^2^ | Not serious | 0/45 | 0/49 | Not estimable | Not estimable | Very low |  |

^1^Downgraded two levels due to lack of blinding, unclear allocation concealment and high risk of attrition bias
^2^Downgraded two levels due to very serious imprecision
^3^Downgraded one level due to significant heterogeneity I^2^=73%
^4^Downgraded one level due to significant imprecision

*Forest plot Probiotics vs Laxatives treatment success*

*Forest plot Probiotics vs laxatives Withdrawals due to AE*

*Forest plot probiotics vs laxatives Defecation frequency*

*Forest plot probiotics vs laxatives Stool consistency*

3. Additional effect: Probiotics + laxative vs laxative (N=9)

**Notes**: included PEG and Lactulose as laxatives. Did not use data from Kubota 2020 (yet) because defecation frequency was reported as “Change from baseline in Bowel movements per week, least square mean (95% CI)” and could not be included in the meta-analysis.
Abediny 2016, Foroughi 2022, Russo 2017, Sadeghzadeh 2014 investigated multispecies probiotics. Banaszkiewicz 2005 investigated Lacticaseibacillus rhamnosus. Jadrešin 2018, Kubota 2020, and Wegner investigated 2018 L. reuteri.
**Question:** Should probiotics in addition to a laxative vs laxative only be used for functional constipation?
**Bibliography:** Abediny 2016, Banaszkiewicz 2005, Foroughi 2022, Jadrešin 2018, Lee 2022, Kubota 2020, Russo 2017, Wegner 2018, Sadeghzadeh 2014

GRADE

| **Quality assessment** | | | | | | | **No of patients** | | **Effect** | | **Certainty** | **Importance** |
| --- | --- | --- | --- | --- | --- | --- | --- | --- | --- | --- | --- | --- |
|  |  |  |  |  |  |  |  |  |  |  |  |  |
| **No of studies** | **Design** | **Risk of bias** | **Inconsistency** | **Indirectness** | **Imprecision** | **Other considerations** | **Probiotics + laxative** | **Laxative** | **Relative (95% CI)** | **Absolute** |  |  |
| **Treatment success (time of measurement range: 8 to 12 weeks)** | | | | | | | | | | | | |
| 4 | Randomised controlled trials | Very serious^1^ | Not serious | Not serious | Serious^5^ | Not serious | 86/156 | 74/153 | RR: 1.08 (0.87 to 1.34) | 25 more per 1000 (from 40 less to 104 more per 1000)  Trivial magnitude in favour (trivial against to small in favour) | Very low |  |
| **Withdrawals due to Adverse Events at study end (time of measurement range: 4 to 12 weeks)** | | | | | | | | | | | | |
| 6 | Randomised controlled trials | Very serious^1^ | Not serious | Not serious | Very serious^2^ | Not serious | 49/247 | 58/242 | RR: 0.71 (0.24 to 2.07) | 70 less per 1000 (from 182 less to 256 more per 1000)  Large magnitude in favour (large in favour to large against) | Very low |  |
| **Defecation frequency per week (time of measurement range: 3 weeks – 3 months)** | | | | | | | | | | | | |
| 5 | Randomised controlled trials | Very serious^1^ | Not serious | Not serious | Not serious | Not serious | N=188 | N=190 |  | MD: 0.12 stools more per week (0.09 lower stools per week to 0.34 higher)  Trivial magnitude in favour (trivial against to trivial in favour) | Low |  |
| **Painful defecation frequency per week (time of measurement: 2 weeks)** | | | | | | | | | | | | |
| 1 | Randomised controlled trials | Very serious^3^ | Not serious | Not serious | Not serious | Not serious | N=61 | N=60 |  | MD: 0.16 higher (from 0.11 lower to 0.43 higher)  Trivial magnitude against (trivial against to trivial in favour) | Low |  |
| **Painful defecation – dichotomous: number of children with painful defecation (time of measurement: 8 weeks) – incompatible outcome** | | | | | | | | | | | | |
| 1 | Randomised controlled trials | Very serious^4^ | Not serious | Not serious | Serious^5^ | Not serious | 13/65 | 8/64 | RR: 1.60 (0.71 to 3.60) | 75 more per 1000 (from 36 lower to 325 more per 1000) | Very Low |  |
| **Stool consistency – continuous: BSFS and scale 1-3 (low is hard stools, high is soft stools) (time of measurement range: 4 to 12 weeks)** | | | | | | | | | | | | |
| 3 | Randomised controlled trials | Very serious^3^ | Not serious | Not serious | Not serious | Not serious | N=110 | N=109 |  | SMD: 0.18 higher (from 0.09 lower to 0.45 higher)  Sensitivity analysis leaving study with scale 1-3 out: MD: 0.09 [-0.21, 0.39]  Estimation: trivial magnitude in favour (trivial against to trivial in favour) | Low |  |
| **Stool consistency – dichotomous: number of patients with hard stools (time of measurement: 4 to 8 weeks) – incompatible outcome** | | | | | | | | | | | | |
| 2 | Randomised controlled trials | Very serious^3^ | Serious^6^ | Not serious | Serious^5^ | Not serious | 12/110 | 11/109 | RR: 1.06 (0.33 to 3.36) | 6 more per 1000 (from 68 less to 238 more per 1000) | Very Low |  |
| **Fecal incontinence frequency per week (time of measurement: 2 to 12 weeks)** | | | | | | | | | | | | |
| 2 | Randomised controlled trials | Very serious^3^ | Not serious | Not serious | Not serious | Not serious | N=104 | N=101 |  | MD: 0.26 higher (from 0.20 lower to 0.72 higher)  Trivial magnitude against (trivial in favour to trivial against) | Very low |  |
| **Fecal incontinence – dichotomous: number of patients with fecal incontinence (time of measurement: 8 weeks) – incompatible outcome** | | | | | | | | | | | | |
| 2 | Randomised controlled trials | Very serious^3^ | Not serious | Not serious | Serious^5^ | Not serious | 19/84 | 12/86 | RR: 1.63 (0.85 to 3.09) | 88 more per 1000 (from 21 less to 292 more per 1000) | Very low |  |
| **Abdominal pain – dichotomous: number of patients with abdominal pain (time of measurement: 4 to 8 weeks) – incompatible outcome** | | | | | | | | | | | | |
| 3 | Randomised controlled trials | Very serious^3^ | Not serious | Not serious | Very serious^2^ | Not serious | 27/135 | 43/134 | RR: 0.64 (0.43 to 0.96) NNT: 9 (5 to 78) | 116 less per 1000 (from 183 less to 13 less per 1000) | Very low |  |
| **Serious adverse events (time of measurement: 8 weeks)** | | | | | | | | | | | | |
| 1 | Randomised controlled trials | Very serious^4^ | Not serious | Not serious | Very serious^2^ | Not serious | 0/65 | 0/64 | Not estimable | Not estimable | Very low |  |
| **Adverse events (time of measurements range: 4 to 12 weeks)** | | | | | | | | | | | | |
| 4 | Randomised controlled trials | Very serious^3^ | Not serious | Not serious | Serious^5^ | Not serious | 6/173 | 8/173 | RR: 0.72 (0.26 to 1.98) | 13 less per 1000 (from 34 less to 45 more per 1000)  Trivial magnitude in favour (trivial in favour to small against) | Very low |  |

^1^Downgraded two levels due to lack of blinding, high risk of attrition bias and selective reporting, and unclear allocation concealment
^2^Downgraded two levels due to very serious imprecision
^3^Downgraded two levels due to lack of blinding, high risk of attrition bias and unclear allocation concealment
^4^Downgraded two levels due to unclear allocation concealment and unclear blinding.
^5^Downgraded one level due to significant imprecision
^6^Downgraded one level due to heterogeneity I^2^=57%

*Forest plot probiotics + laxatives vs laxatives treatment success*

*Forest plot probiotics + laxatives vs laxatives withdrawals due to AE*

*Forest plot probiotics + laxatives vs laxatives defecation frequency*

*Forest plot probiotics + laxatives vs laxatives stool consistency*

4. Additional effect: probiotics + diet with goat yoghurt vs diet with goat yoghurt (N=1)

**Question:** Should probiotics as addition to goat yoghurt vs goat yoghurt be used for functional constipation? **Bibliography:** Guerra 2011

GRADE

| **Quality assessment** | | | | | | | **No of patients** | | **Effect** | | **Certainty** | **Importance** |
| --- | --- | --- | --- | --- | --- | --- | --- | --- | --- | --- | --- | --- |
|  |  |  |  |  |  |  |  |  |  |  |  |  |
| **No of studies** | **Design** | **Risk of bias** | **Inconsistency** | **Indirectness** | **Imprecision** | **Other considerations** | **Probiotics + goat yoghurt** | **Goat yoghurt** | **Relative (95% CI)** | **Absolute** |  |  |
| **Treatment success – not reported** | | | | | | | | | | | | |
|  |  |  |  |  |  |  |  |  |  |  |  |  |
| **Withdrawals due to AE (time of measurement: 5 weeks)** | | | | | | | | | | | | |
| 1 | Randomised controlled trials | Serious | Not serious | Not serious | Very serious | Not serious | 1/30 | 0/30 | RR: 3.00 (0.13 to 70.83) | 67 more per 1000 (from 29 less to 1000 more per 1000)  Large magnitude against (large in favour to large against) | Very low |  |
| **Defecation frequency – no data reported** | | | | | | | | | | | | |
|  |  |  |  |  |  |  |  |  |  |  |  |  |

^1^Downgraded one level due to unclear allocation concealment and selective reporting

5. Formula 1 intact protein + probiotic + PEG vs Formula 2 hydrolyzed whey + PEG (N=1)

**Notes:** included children aged 12 to 32 months. Compared two different formulas. Formula 1 (intervention) contained intact protein and a probiotic. Formula 2 (control) contained hydrolyzed whey protein. Both groups also received PEG. **Question:** Should formula with intact protein and a probiotic vs formula with hydrolyzed whey protein in addition to PEG be used for the treatment of functional constipation  **Bibliography**: Sevilla 2022

GRADE

| **Quality assessment** | | | | | | | **No of patients** | | **Effect** | | **Certainty** | **Importance** |
| --- | --- | --- | --- | --- | --- | --- | --- | --- | --- | --- | --- | --- |
|  |  |  |  |  |  |  |  |  |  |  |  |  |
| **No of studies** | **Design** | **Risk of bias** | **Inconsistency** | **Indirectness** | **Imprecision** | **Other considerations** | **Formula 1** | **Formula 2** | **Relative (95% CI)** | **Absolute** |  |  |
| **Treatment success (time of measurement: 8 weeks)** | | | | | | | | | | | | |
| 1 | Randomised controlled trials | Very serious^1^ | Not serious | Not serious | Very serious^5^ | Not serious | 43/47 | 38/48^3^ | RR: 1.16 (0.98 to 1.37) | 127 more per 1000 (from 16 less to 293 more per 1000)  Small magnitude in favour (trivial against to large in favour) | Very low |  |
| **Withdrawals due to Adverse Events (time of measurement: 8 weeks)** | | | | | | | | | | | | |
| 1 | Randomised controlled trials | Very serious^1^ | Not serious | Not serious | Very serious^5^ | Not serious | 2/47 | 0/48 | RR: 5.10 (0.25 to 103.57) | 86 more per 1000 (from 16 less to 1000 more per 1000)^2^  Large magnitude against (moderate in favour to large against) | Very low |  |
| **Defecation frequency per week (time of measurement: mean week 1-8)** | | | | | | | | | | | | |
| 1 | Randomised controlled trials | Very serious^1^ | Not serious | Not serious | Serious^6^ | Not serious | N=47 | N=48 |  | MD: 0.06 lower (from 1.54 lower to 1.42 higher)  Trivial magnitude against (small against to small in favour) | Very low |  |
| **Painful defecation – dichotomous: number of patients (time of measurement: 8 weeks) – incompatible outcome** | | | | | | | | | | | | |
| 1 | Randomised controlled trials | Very serious^1^ | Not serious | Not serious | Serious | Not serious | 6/47 | 6/48 | RR: 1.02 (0.35 to 2.94) | 3 more per 1000 (from 81 less to 243 more per 1000) | Very low |  |
| **Stool consistency – dichotomous: number of subjects with a hard stool on one or more occasions throughout week 5-8 – incompatible outcome** | | | | | | | | | | | | |
| 1 | Randomised controlled trials | Very serious^1^ | Not serious | Not serious | Very serious | Not serious | 10/47 | 14/48 | RR: 0.73 (0.36 to 1.48) | 79 less per 1000 (from 187 less to 140 more per 1000) | Very low |  |
| **Fecal incontinence – dichotomous: number of subjects with fecal incontinence throughout week 5-8^4^ – incompatible outcome** | | | | | | | | | | | | |
| 1 | Randomised controlled trials | Very serious^1^ | Not serious | Not serious | Very serious | Not serious | 10/47 | 14/48 | RR: 0.73 (0.36 to 1.48) | 79 less per 1000 (from 187 less to 140 more per 1000) | Very low |  |
| **Serious adverse events (time of measurement: 8 weeks)** | | | | | | | | | | | | |
| 1 | Randomised controlled trials | Very serious^1^ | Not serious | Not serious | Very serious | Not serious | 0/47 | 0/48 | Not estimable | Not estimable | Very low |  |
| **Adverse events (time of measurement: 8 weeks)** | | | | | | | | | | | | |
| 1 | Randomised controlled trials | Very serious^1^ | Not serious | Not serious | Very serious | Not serious | 0/47 | 0/48 | Not estimable | Not estimable | Very low |  |

^1^Downgraded two levels due to lack of blinding and unclear allocation concealment
^2^Added 1 fictional event to Formula 2 group to calculate absolute numbers
^3^Please note the very high success numbers in both groups. This could indicate that PEG is effective in children aged 1 to 2,5 years old.
^4^Please note that fecal incontinence can be considered as an invalid outcome in such young children.
^5^Downgraded two levels due to very serious imprecision
^6^Downgraded one level due to serious due to significant imprecision

*Forest plot infant formula with intact protein + probiotic + PEG vs. infant formula with hydrolysed whey protein + PEG: treatment success*

*Forest plot infant formula with intact protein + probiotic + PEG vs. infant formula with hydrolysed whey protein + PEG: defecation frequency per week*

*Forest plot infant formula with intact protein + probiotic + PEG vs. infant formula with hydrolysed whey protein + PEG: withdrawals due to adverse events at study end*

Herbal medicine

1. Herbal medicine vs laxative (N=8)

**Notes:** Herbal medicines consisted of Black Strap Molasses, Cassia fistula, LaxaPlus Barij®, flixweed (D. Sophia seed), Golghand®, and Viola Flower Syrup, R. damascena and brown sugar. Mozaffarpur 2012 compared herbal medicine to liquid paraffin, all other studies compared herbal medicine to PEG. **Question:** Should herbal medicine vs laxative be used for functional constipation? **Bibliography:** Dehghani 2019, Esmaeilidooki 2016, Imanieh 2022, Nasri 2022, Nimrouzi 2015, Saneian 2021, Tavassoli 2021, Mozaffarpur 2012

GRADE

| **Quality assessment** | | | | | | | **No of patients** | | **Effect** | | **Certainty** | **Importance** |
| --- | --- | --- | --- | --- | --- | --- | --- | --- | --- | --- | --- | --- |
|  |  |  |  |  |  |  |  |  |  |  |  |  |
| **No of studies** | **Design** | **Risk of bias** | **Inconsistency** | **Indirectness** | **Imprecision** | **Other considerations** | **Herbal mediicne** | **Laxative** | **Relative (95% CI)** | **Absolute** |  |  |
| **Treatment success (time of measurement range: 3 to 8 weeks)** | | | | | | | | | | | | |
| 5 | Randomised controlled trials | Serious^1^ | Serious^4^ | Not serious | Very serious^3^ | Not serious | 191/248 | 176/254 | RR: 1.11 (0.92 to 1.34) | 76 more per 1000 (from 55 less to 236 more per 1000)  Small magnitude in favour (trivial against to large against) | Very low |  |
| **Withdrawals due to Adverse Events at study end (range: 3 to 8 weeks)** | | | | | | | | | | | | |
| 8 | Randomised controlled trials | Serious^1^ | Not serious | Not serious | Very serious^3^ | Not serious | 7/408 | 12/414 | RR: 0.63 (0.26 to 1.53) | 14 less per 1000 (from 27 less to 20 more per 1000)  Small magnitude in favour (large in favour to moderate against) | Very low |  |
| **Defecation frequency per week (time of measurement range: 3 to 8 weeks)** | | | | | | | | | | | | |
| 5 | Randomised controlled trials | Serious^1^ | Serious^4^ | Not serious | Very serious^3^ | Not serious | N=242 | N=237 |  | MD: 1.72 stools more per week (0.23 stools less per week to 3.67 stools more per week)^7^  Small magnitude in favour (trivial against to large in favour) | Very low |  |
| **Painful defecation – dichotomous: number of patients with painful defecation (time of measurement: 8 weeks) – incompatible outcome** | | | | | | | | | | | | |
| 2 | Randomised controlled trials | Very serious^5^ | Not serious | Not serious | Serious^2^ | Not serious | 28/90 | 25/90 | RR: 1.11 (0.70 to 1.75) | 31 more per 1000 (from 83 less to 208 more per 1000) | Very low |  |
| **Painful defecation – continuous: painful defecations per week (time of measurement: 3 to 4 weeks)** | | | | | | | | | | | | |
| 2 | Randomised controlled trials | Serious^6^ | Serious^4^ | Not serious | Not serious | Not serious | N=123 | N=119 |  | MD: 0.10 higher (from 0.52 lower to 0.71 higher)  Trivial magnitude against (trivial in favour to trivial against) | Very low |  |
| **Painful defecation – pain severity on a VAS-scale (0-100) (time of measurement range: 3 to 4 weeks)** | | | | | | | | | | | | |
| 2 | Randomised controlled trials | Serious^6^ | Serious^4^ | Not serious | Serious^2^ | Not serious | N=89 | N=91 |  | MD: 8.19 lower (from 21.40 lower to 5.02 higher)  Trivial magnitude in favour (small in favour to trivial against) | Very low |  |
| **Stool consistency –** **frequency of hard stools per week (time of measurement range: 3 to 4 weeks) – incompatible outcome** | | | | | | | | | | | | |
| 2 | Randomised controlled trials | Very serious^4^ | Serious^4^ | Not serious | Serious^2^ | Not serious | N = 119 | N = 123 |  | MD: 0.45 lower (from 1.39 lower to 0.49 higher) | Very low |  |
| **Stool consistency - reported on VAS score (0-100), 0=soft (time of measurement range: 3 to 4 weeks)** | | | | | | | | | | | | |
| 2 | Randomised controlled trials | Very serious^4^ | Serious^4^ | Not serious | Serious^2^ | Not serious | N=89 | N=91 |  | MD: 8.44 lower (from 16.77 lower to 0.11 lower)  Trivial magnitude in favour (small in favour to trivial in favour) | Very low |  |
| **Fecal incontinence frequency per week (time of measurement range: 3 to 4 weeks)** | | | | | | | | | | | | |
| 4 | Randomised controlled trials | Very serious^4^ | Not serious | Not serious | Serious^2^ | Not serious | N=212 | N=210 |  | MD: 0.43 lower (from 1.39 lower to 0.52 higher)  Trivial magnitude in favour (small in favour to trivial against) | Very low |  |
| **Adverse events (time of measurement: 4 weeks)** | | | | | | | | | | | | |
| 3 | Randomised controlled trials | Serious^6^ | Not serious | Not serious | Very serious^3^ | Not serious | 20/164 | 39/179 | RR: 0.49 (0.15 to 1.60) | 111 less per 1000 (from 185 less to 131 more per 1000)  Moderate magnitude in favour (large in favour to large against) | Very low |  |

^1^Downgraded one level due to open label studies, high risk selective reporting, high risk for other bias
^2^Downgraded one level due to significant imprecision
^3^Downgraded two levels due to very serious imprecision
^4^Downgraded one level due to significant heterogeneity. Could be explained by the different types of herbal medicine and/or different types of laxatives.
^5^Downgraded two levels due to high risk performance and assessment bias, and high risk selective reporting
^6^Downgraded one level due to open label studies
^7^Sensitivity analysis for only Esmaeilidooki 2016 and Mozaffarpur 2012 (both Cassia fistula as intervention), led to a significant difference favoring Cassia fistula (MD 4.22 higher, 95% CI 2.78 higher to 5.66 higher per week). However evidence would be low/very low (very serious RioB).

*Forest plot Herbal medicine vs laxatives Treatment success*

*Forest plot Herbal medicine vs laxatives Withdrawals due to AE*

*Forest plot Herbal medicine vs laxatives Defecation frequency*

*Forest plot Herbal medicine vs laxatives Stool consistency - frequency of hard stools per week*

*Forest plot Herbal medicine vs laxatives Stool consistency – VAS-score*

2. Herbal medicine vs placebo (N=2)

**Note:** Cai 2018 investigated Xiao’er Biantong granules, Qiao 2021 investigated XiaojiDaozhi Decoction, a mixture of 12 different herbs. **Question:** Should herbal medicine vs placebo be used for functional constipation? **Bibliography:** Cai 2018, Qiao 2021

GRADE

| **Quality assessment** | | | | | | | **No of patients** | | **Effect** | | **Certainty** | **Importance** |
| --- | --- | --- | --- | --- | --- | --- | --- | --- | --- | --- | --- | --- |
|  |  |  |  |  |  |  |  |  |  |  |  |  |
| **No of studies** | **Design** | **Risk of bias** | **Inconsistency** | **Indirectness** | **Imprecision** | **Other considerations** | **Herbal medicine** | **Placebo** | **Relative (95% CI)** | **Absolute** |  |  |
| **Treatment success (time of measurement: 2 weeks to 8 weeks)** | | | | | | | | | | | | |
| 2 | Randomised controlled trials | Very serious^1^ | Very serious^4^ | Not serious | Very serious^2^ | Not serious | 264/460 | 46/220 | RR: 5.04 (0.21 to 121.88) | 845 more per 1000 (from 165 less to 1000 more per 1000)  Large magnitude in favour (moderate against to large in favour) | Very low |  |
| **Withdrawals due to Adverse Events at study end (time of measurement: 2 weeks to 8 weeks)** | | | | | | | | | | | | |
| 2 | Randomised controlled trials | Very serious^1^ | Not serious | Not serious | Very serious^2^ | Not serious | 48/460 | 37/220 | RR: 0.70 (0.46 to 1.05) | 51 less per 1000 (from 91 less to 8 more per 1000)  Large magnitude in favour (large in favour to trivial against) | Very low |  |
| **Defecation frequency – continuous: frequency per week (time of measurement: 8 weeks)** | | | | | | | | | | | | |
| 1 | Randomised controlled trials | Very serious^1^ | Not serious | Not serious | Serious^5^ | Not serious | N=100 | N=100 |  | MD: 1.17 stools per week more (from 0.68 more to 1.66 more)  Small magnitude in favour (trivial in favour to small in favour) | Very low |  |
| **Defecation frequency – dichotomous: number of children with ≥3 bowel movements per week (time of measurement range: 2 weeks)** | | | | | | | | | | | | |
| 1 | Randomised controlled trials | Very serious^1^ | Not serious | Not serious | Not serious | Not serious | 291/360 | 34/120 | RR 2.85 (2.14 to 3.81) | 524 more per 1000 (from 323 more to 796 more per 1000)  Large magnitude in favour (large in favour to large in favour) | Low |  |
| **Stool consistency – Disappearance rate of dry stool (type 1 and 2 Bristol Stool Scale)** | | | | | | | | | | | | |
| 1 | Randomised controlled trials | Very serious^1^ | Not serious | Not serious | Not serious | Not serious | 236/360 | 11/120 | RR 7.15 (4.05 to 12.62) | 564 more per 1000 (from 280 more to 1000 more per 1000)  Large magnitude in favour (large in favour to large in favour) | Low |  |
| **Fecal incontinence – number of children with fecal incontinence (time of measurement range: 8 weeks) – incompatible outcome** | | | | | | | | | | | | |
| 1 | Randomised controlled trials | Very serious^1^ | Not serious | Not serious | Serious^5^ | Not serious | 6/100 | 15/100 | RR: 0.40 (0.16 to 0.99) | 90 less per 1000 (from 126 less to 2 less per 1000) | Very low |  |
| **Adverse events** | | | | | | | | | | | | |
| 2 | Randomised controlled trials | Very serious^1^ | Not serious | Not serious | Very serious^2^ | Not serious | 18/460 | 5/220 | 2.28 (0.75 to 6.91) | 29 more per 1000 (from 6 less to 134 more per 1000)  Trivial magnitude in against (trivial in favour to large against) | Very low |  |

^1^Downgraded two levels due to unclear risk of attrition bias and selecting reporting
^2^Downgraded two levels due to significant imprecision
^3^Inserted a fictional event (1/3) for placebo in Revman to calculate absolute numbers.
^4^Downgraded two levels due to very serious inconsistency (I2=97%)
^5^Downgraded one level due to significant imprecision

*Forest plot Herbal medicine vs placebo Treatment success*

*Forest plot Herbal medicine vs placebo Withdrawals due to AE*

Fibers

1. Fiber vs placebo (N=4)

**Notes:** Outcome data from Loening-Baucke 2004 were not included, because no data pre cross-over was available. The study compared Glucomannan fiber to placebo (4 weeks treatment and then cross-over, without washout period).
Weber 2014 investigated mixture of several fibers and reported in their methods that “It should be emphasized that several of the components (10.5% fructooligosaccharides, 12.5% inulin, 24% gum arabic, 9% resistant starch, 33% soy polysaccharide, and 12% cellulose) are considered prebiotics.”
Da Silva Souze 2018 only included children aged 6 months to 24 months old and investigated Fructo-oligo-saccharide (in the study defined as prebiotics).
**Question:** Should fiber vs placebo be used for functional constipation?
**Bibliography:** Chmielewska 2011, Da Silva Souza 2018, Loening-Baucke 2004, Weber 2014

GRADE

| **Quality assessment** | | | | | | | **No of patients** | | **Effect** | | **Certainty** | **Importance** |
| --- | --- | --- | --- | --- | --- | --- | --- | --- | --- | --- | --- | --- |
|  |  |  |  |  |  |  |  |  |  |  |  |  |
| **No of studies** | **Design** | **Risk of bias** | **Inconsistency** | **Indirectness** | **Imprecision** | **Other considerations** | **Fiber** | **Placebo** | **Relative (95% CI)** | **Absolute** |  |  |
| **Treatment success (time of measurement: 4 weeks)** | | | | | | | | | | | | |
| 3 | Randomised controlled trials | Serious^1^ | Not serious | Not serious | Very serious^2^ | Not serious | 52/86 | 49/89 | RR: 1.12 [0.87, 1.44] | 66 more per 1000 (from 72 less to 242 more per 1000)  Small magnitude in favour (small against to large in favour) | Very low |  |
| **Withdrawals due to Adverse Events at study end (time of measurement: 4 weeks)** | | | | | | | | | | | | |
| 4 | Randomised controlled trials | Serious | Not serious | Not serious | Very serious^2^ | Not serious | 12/113 | 11/108 | RR: 0.84 (0.41 to 1.73) | 16 less per 1000 (from 60 less to 74 more per 1000)  Moderate magnitude in favour (large in favour to large against) | Very low |  |
| **Defecation frequency per week (time of measurement: 4 weeks)** | | | | | | | | | | | | |
| 3 | Randomised controlled trials | Serious | Very serious | Not serious | Very serious^2^ | Not serious | N=80 | N=82 |  | SMD: 0.96 higher (from 0.14 lower to 2.07 higher)  Estimation: moderate in favour (trivial against to large in favour) | Very low |  |
| **Painful defecation frequency per week (time of measurement: 4 weeks)** | | | | | | | | | | | | |
| 1 | Randomised controlled trials | Serious | Not serious | Not serious | Very serious^2^ | Not serious | N=36 | N=36 |  | MD: not estimable | Very low |  |
| **Painful defecation - % of bowel movements (time of measurement: 4 weeks) – incompatible outcome** | | | | | | | | | | | | |
| 1 | Randomised controlled trials | Serious^1^ | Not serious | Not serious | Serious | Not serious | N=18 | N=18 |  | MD: 13.71 lower (from 37.99 lower to 10.57 more) | Low |  |
| **Stool consistency BSFS 4-7 were scored as non hardened stool (time of measurement: 4 weeks)** | | | | | | | | | | | | |
| 1 | Randomised controlled trials | Serious^1^ | Not serious | Not serious | Very serious^2^ | Not serious | 12/27 | 4/30 | RR: 3.33 (1.22 to 9.11) | 311 more per 1000 (from 29 more to 1000 more per 1000)  Large magnitude in favour (trivial against to large in favour) | Very low |  |
| **Stool consistency BSFS (1-7) (time of measurement: 4 weeks)** | | | | | | | | | | | | |
| 1 | Randomised controlled trials | Serious | Not serious | Not serious | Not serious | Not serious | N=36 | N=36 |  | MD: 0.10 lower (from 0.59 lower to 0.39 higher)  Trivial magnitude against (trivial against to trivial in favour) | Moderate |  |
| **Stool consistency - % of BMs with soft stool (time of measurement: 4 weeks) – incompatible outcome** | | | | | | | | | | | | |
| 1 | Randomised controlled trials | Serious^1^ | Not serious | Not serious | Very serious | Not serious | N=18 | N=18 |  | MD: 18.00 higher (from 3.58 lower to 39.58 more) | Very low |  |
| **Abdominal pain frequency episodes per week (time of measurement: 4 weeks)** | | | | | | | | | | | | |
| 1 | Randomised controlled trials | Serious | Not serious | Not serious | Not serious | Not serious | N=36 | N=36 |  | MD: 0.00 (from 0.54 lower to 0.54 higher) | Moderate |  |
| **Serious adverse events (time of measurement: 4 weeks)** | | | | | | | | | | | | |
| 3 | Randomised controlled trials | Serious | Not serious | Not serious | Very serious^2^ | Not serious | 1/94 | 0/89 | RR: 3.00 (from 0.13 to 71.51) | Not estimable | Very low |  |
| **Adverse events (time of measurement: 4 weeks)** | | | | | | | | | | | | |
| 2 | Randomised controlled trials | Very serious^3^ | Not serious | Not serious | Very serious^2^ | Not serious | 4/46 | 1/38 | 4.00 [0.49 to 32.57] | 79 less per 1000 (from 13 less to 831 more per 1000)^4^  Moderate magnitude in favour (trivial in favour to large against) | Very low |  |

^1^Downgraded one level due to unclear allocation concealment and selective reporting in one study
^2^Downgraded two levels due to very serious imprecision
^3^Downgraded two levels due to unclear risk of bias in almost every domain
^4^Added fictional event to control group to obtain absolute numbers, in order to better interpret results.

*Forest plot Fibers vs placebo Treatment success*

*Forest plot Fibers vs placebo withdrawals due to AE*

*Forest plot Fibers vs placebo Defecation frequency*

2. Fiber vs laxative (N=5)

**Notes:** Cassetari 2018 investigated the laxatives PEG and sodium picosulfate^5^. The comparison of Green Banana Biomass vs Sodium Picosulfate was not included, because then the Green Banana Biomass group would be included twice in the overall comparison of fiber vs laxative.
Kokke 2018 compared a fiber mixture to lactulose. Quitadamo 2012 compared a fiber mixture to PEG. Üstündağ 2010 compared partially hydrolysed guargum to lactulose. Foroughi 2022 investigates Pysllium Seed Husk Powder
**Question:** Should fiber vs laxative be used for functional constipation?
**Bibliography:** Cassetari 2018, Foroughi 2022, Kokke 2008, Quitadamo 2012, Üstündağ 2010

GRADE

| **Quality assessment** | | | | | | | **No of patients** | | **Effect** | | **Certainty** | **Importance** |
| --- | --- | --- | --- | --- | --- | --- | --- | --- | --- | --- | --- | --- |
|  |  |  |  |  |  |  |  |  |  |  |  |  |
| **No of studies** | **Design** | **Risk of bias** | **Inconsistency** | **Indirectness** | **Imprecision** | **Other considerations** | **Fiber** | **Laxative** | **Relative (95% CI)** | **Absolute** |  |  |
| **Treatment success (time of measurement: 8 weeks)** | | | | | | | | | | | | |
| 1 | Randomised controlled trials | Very serious^1^ | Not serious | Not serious | Very serious^2^ | Not serious | 28/50 | 38/50 | RR: 0.74 (0.55 to 0.99) | 197 less per 1000 (from 342 less to 8 less per 1000)  Moderate magnitude against (large against to trivial against) | Very low |  |
| **Withdrawals due to Adverse Events at study end (range: 4 - 8 weeks)** | | | | | | | | | | | | |
| 3 | Randomised controlled trials | Very serious^1^ | Not serious | Not serious | Very serious^2^ | Not serious | 6/155 | 5/148 | RR: 1.16 (0.37 to 3.62) | 5 more per 1000 (from 21 less to 89 more per 1000)  Small magnitude against (moderate against to large in favour) | Very low |  |
| **Defecation frequency per week (time of measurement range: 4 - 8 weeks)** | | | | | | | | | | | | |
| 3 | Randomised controlled trials | Very serious^1^ | Serious | Not serious | Serious^4^ | Not serious | N=103 | N=113 |  | MD: 0.96 stools less per week (1.65 stools less per week 0.28 stools less per week)  Trivial magnitude against (small against to trivial against) | Very low |  |
| **Defecation frequency – dichotomous: number of patients having more than 3 bowel motions as week (time of measurement: 8 weeks)** | | | | | | | | | | | | |
| 1 | Randomised controlled trials | Serious^3^ | Not serious | Not serious | Very serious^2^ | Not serious | 9/15 | 8/17 | RR: 1.27 (0.66 to 2.45) | 127 more per 1000 (from 160 less to 682 more per 1000)  Small magnitude in favour (moderate against to large in favour) | Very low |  |
| **Painful defecation – number of patients reporting painful stools (time of measurement; 8 weeks) – incompatible outcome** | | | | | | | | | | | | |
| 1 | Randomised controlled trials | Serious^3^ | Not serious | Not serious | Very serious^2^ | Not serious | 4/15 | 4/16 | RR: 1.07 (0.32 to 3.52) | 17 more per 1000 (from 170 less to 630 more per 1000) | Very low |  |
| **Number of painless bowel movements per week (time of measurement: 3 weeks) – incompatible outcome** | | | | | | | | | | | | |
| 1 | randomised trials | Very serious^5^ | Not serious | Not serious | Not serious | Not serious | N = 36 | N = 36 |  | MD: 1.58 less painless bowel movements (2.18 less painless bowel movements to 0.98 less painless bowel movements) | Low |  |
| **Fecal incontinence - number of patients with 1 or more fecal incontinence episodes per week (time of measurement; 8 weeks) – incompatible outcome** | | | | | | | | | | | | |
| 1 | Randomised controlled trials | Very serious^1^ | Not serious | Not serious | Serious^4^ | Not serious | 9/70 | 5/70 | RR: 1.80 (0.64 to 5.10) | 57 more per 1000 (from 26 less to 293 more per 1000) | Very low |  |
| **Fecal incontinence frequency per week (time of measurement; 8 weeks)** | | | | | | | | | | | | |
| 1 | Randomised controlled trials | Very serious^1^ | Not serious | Not serious | Not serious | Not serious | N=36 | N=47 |  | MD: 0.10 higher (from 0.42 lower to 0.62 higher)  Trivial magnitude against (trivial in favour to trivial against) | Low |  |
| **Abdominal pain – number of patients with abdominal pain (time of measurement; 4 - 8 weeks) – incompatible outcome** | | | | | | | | | | | | |
| 3 | Randomised controlled trials | Very serious^1^ | Not serious | Not serious | Serious^4^ | Not serious | 15/86 | 14/97 | RR: 1.21 (0.63 to 2.33) | 30 more per 1000 (from 53 less to 192 more per 1000) | Very low |  |
| **Serious adverse events (time of measurement; 8 weeks)** | | | | | | | | | | | | |
| 2 | Randomised controlled trials | Very serious^1^ | Not serious | Not serious | Very serious^2^ | Not serious | 0/80 | 0/86 | Not estimable | Not estimable | Very low |  |
| **Adverse events (time of measurement; 8 weeks)** | | | | | | | | | | | | |
| 3 | Randomised controlled trials | Very serious^1^ | Not serious | Not serious | Very serious^2^ | Not serious | 0/116 | 0/133 | Not estimable | Not estimable | Very low |  |

^1^Downgraded two levels due to lack of blinding and unclear allocation concealment and risk of selective reporting
^2^Downgraded two levels due to very serious imprecision
^3^Downgraded one level due to unclear allocation concealment
^4^Downgraded one level due to significant imprecision
^5^Now only included comparison of Green Banana Biomass vs PEG, because there is also another study comparing fiber vs PEG. The comparison of GBB vs sodium picosulfate was also made in Cassetari. However both laxatives were compared to the same GBB group, so including both comparisons would cause that the GBB group would be included twice in the comparison of Fiber vs Laxative. The comparison of GBB vs sodium picosulfate is discussed separately in the pharma part.

*Forest plot Fibers vs laxatives Withdrawals due to AE*

*Forest plot Fibers vs laxatives Defecation frequency*

Infant formula

1. Formula with prebiotics + hydrolyzed whey protein vs formula with intact protein (N=2)

**Notes:** Bongers 2007, children were aged 3 – 20 weeks. Savino 2005, children were aged max 16 weeks old.  **Question:** Should formula with prebiotics and hydrolyzed whey protein vs formula with intact protein be used for functional constipation? **Bibliography**: Bongers 2007, Savino 2005

GRADE

| **Quality assessment** | | | | | | | **No of patients** | | **Effect** | | **Quality** | **Importance** |
| --- | --- | --- | --- | --- | --- | --- | --- | --- | --- | --- | --- | --- |
|  |  |  |  |  |  |  |  |  |  |  |  |  |
| **No of studies** | **Design** | **Risk of bias** | **Inconsistency** | **Indirectness** | **Imprecision** | **Other considerations** | **Formula with prebiotics + hydrolyzed whey protein** | **Formula with intact protein** | **Relative (95% CI)** | **Absolute** |  |  |
| **Treatment success (time of measurement range: ) – not reported** | | | | | | | | | | | | |
|  |  |  |  |  |  |  |  |  |  |  |  |  |
| **Withdrawals due to Adverse Events at study end (range: 2 -3 weeks )** | | | | | | | | | | | | |
| 2 | Randomised controlled trials | Very serious^1^ | Not serious | Not serious | Very serious^2^ | Not serious | 0/89 | 1/69 | RR: 0.25 (0.01 to 5.83) | 11 less per 1000 (from 14 less to 70 more per 1000)  Small magnitude in favour (small in favour to large against) | Very low |  |
| **Defecation frequency (time of measurement range: 2 - 3 weeks)** | | | | | | | | | | | | |
| 2 | Randomised controlled trials | Very serious^2^ | Not serious | Not serious | Serious^4^ | Not serious | N=75 | N=55 |  | SMD: 0.38 higher (0.03 higher to 0.73 higher)  Estimation: trivial in favour (trivial in favour to small in favour) | Very low |  |
| **Painful defecation – number of patients with painful stools (time of measurement: 3 weeks) – incompatible outcome** | | | | | | | | | | | | |
| 1 | Randomised controlled trials | Serious^3^ | Not serious | Not serious | Very serious^2^ | Not serious | 13/20 | 10/15 | RR: 0.97 (0.60 to 1.58) | 20 less per 1000 (from 280 less to 387 more per 1000) | Very low |  |
| **Stool consistency - number of patients with formed stools (on scale of hard/formed/runny) (time of measurement range: 2-3 weeks)** | | | | | | | | | | | | |
| 1 | Randomised controlled trials | Very serious^1^ | Not serious | Not serious | Very serious^2^ | Not serious | 18/69 | 17/54 | RR: 1.29 (0.79 to 2.10) | 91 more per 1000 (from 66 less to 346 more per 1000)  Small magnitude in favour (small against to large in favour) | Very low |  |
| **Serious adverse events (time of measurement range: 2-3 weeks)** | | | | | | | | | | | | |
| 2 | Randomised controlled trials | Very serious^1^ | Not serious | Not serious | Very serious^2^ | Not serious | 0/89 | 0/69 | Not estimable | Not estimable | Very low |  |
| **Adverse events (time of measurement range: 2-3 weeks)** | | | | | | | | | | | | |
| 2 | Randomised controlled trials | Very serious^1^ | Not serious | Not serious | Very serious^2^ | Not serious | 0/89 | 0/69 | Not estimable | Not estimable | Very low |  |

^1^Downgraded two levels due to lack of blinding and unclear risks of selective reporting and attrition bias
^2^Downgraded two levels due to very serious imprecision
^3^Downgraded one level due to unclear allocation concealment and unclear risk of selective reporting
^4^Downgraded one level due to significant imprecision

*Forest plot Formula with prebiotics + hydrolyzed whey protein vs formula with intact protein withdrawals due to AE*

*Forest plot Formula with prebiotics + hydrolyzed whey protein vs formula with intact protein defecation frequency*

Synbiotics

1. Synbiotics vs placebo (N=1)

**Question:** Should synbiotics vs placebo be used for functional constipation? **Bibliography:** Baştürk 2017

GRADE

| **Quality assessment** | | | | | | | **No of patients** | | **Effect** | | **Certainty** | **Importance** |
| --- | --- | --- | --- | --- | --- | --- | --- | --- | --- | --- | --- | --- |
|  |  |  |  |  |  |  |  |  |  |  |  |  |
| **No of studies** | **Design** | **Risk of bias** | **Inconsistency** | **Indirectness** | **Imprecision** | **Other considerations** | **Synbiotics** | **Placebo** | **Relative (95% CI)** | **Absolute** |  |  |
| **Treatment success (time of measurement: 4 weeks)** | | | | | | | | | | | | |
| 1 | Randomised controlled trial | Very serious^1^ | Not serious | Not serious | Not serious | Not serious | 48/77 | 21/78 | RR: 2.32 (1.54 to 3.47)  NNT: 3 (2 to 7) | 355 more per 1000 (from 145 more to 665 more per 1000)  Large magnitude in favour (moderate in favour to large in favour) | Low |  |
| **Withdrawals due to Adverse Events at study end (time of measurement: 4 weeks)** | | | | | | | | | | | | |
| 1 | Randomised controlled trial | Very serious^1^ | Not serious | Not serious | Very serious^3^ | Not serious | 5/77 | 4/78 | RR: 1.27 (0.35 to 4.54) | 14 more per 1000 (from 33 less to 182 more per 1000)  Small magnitude against (large in favour to large against) | Very low |  |
| **Defecation frequency per week (time of measurement range: ) – not adequately reported** | | | | | | | | | | | | |
|  |  |  |  |  |  |  |  |  |  |  |  |  |
| **Painful defecation – number of patients (time of measurement: 4 weeks)** | | | | | | | | | | | | |
| 1 | Randomised controlled trials | Very serious^1^ | Not serious | Not serious | Serious^2^ | Not serious | 16/77 | 27/78 | RR: 0.60 (0.35 to 1.02) | 138 less per 1000 (from 225 less to 7 more per 1000) | Very low |  |
| **Stool consistency – not adequately reported** | | | | | | | | | | | | |
|  |  |  |  |  |  |  |  |  |  |  |  |  |
| **Fecal incontinence – not adequately reported** | | | | | | | | | | | | |
|  |  |  |  |  |  |  |  |  |  |  |  |  |
| **Abdominal pain – number of patients (time of measurement: 4 weeks)** | | | | | | | | | | | | |
| 1 | Randomised controlled trials | Very serious^1^ | Not serious | Not serious | Not serious | Not serious | 4/77 | 41/78 | RR: 0.10 (0.04 to 0.26) | 473 less per 1000 (from 505 less to 389 less per 1000) | Low |  |
| **Adverse events (time of measurement: 4 weeks)** | | | | | | | | | | | | |
| 1 | Randomised controlled trials | Very serious^1^ | Not serious | Not serious | Very serious^3^ | Not serious | 0/77 | 0/78 | Not estimable | Not estimable | Very low |  |

^1^Downgraded two levels due to unclear randomization methods and unclear risk of attrition and selective reporting bias
^2^Downgraded one level due to significant imprecision
^3^Downgraded two levels due to very serious imprecision

*Synbiotics vs placebo treatment success*

*Synbiotics vs placebo withdrawals due to adverse events*

2. Synbiotics vs. laxative (N = 2)

**Notes:** Foroughi 2022 used PEG as laxative. Khodadad 2010 used liquid paraffin as laxative.

**Question:** Should synbiotics compared to laxative be used for the treatment of functional constipation in children? **Bibliography**: Foroughi 2022, Khodadad 2010

GRADE

| **Quality assessment** | | | | | | | **N^o^ of patients** | | **Effect** | | **Certainty** |
| --- | --- | --- | --- | --- | --- | --- | --- | --- | --- | --- | --- |
|  |  |  |  |  |  |  |  |  |  |  |  |
| **N^o^ of studies** | **Design** | **Risk of bias** | **Inconsistency** | **Indirectness** | **Imprecision** | **Publication bias** | **Synbiotics** | **Laxative** | **Relative (95% CI)** | **Absolute** |  |
| **Treatment success (time of measurement: 4 weeks)** | | | | | | | | | | | |
| 1 | Randomised controlled trial | Very serious^1^ | NA | Not serious | Very serious^4^ | NA | 22/31 | 24/29 | RR: 0.86 (0.65 to 1.13) | 116 less per 1000 (from 290 less to 108 more per 1000)  Small magnitude against (large against to small in favour) | Very low |
| **Defecation frequency per week (time of measurement: 3 to 4 weeks)** | | | | | | | | | | | |
| 2 | Randomised controlled trial | Very serious^2^ | Very serious^3^ | Not serious | Serious^5^ | NA | N = 67 | N = 65 | MD: -0.23 (-1.34 to 0.87) | MD: 0.23 lower (from 1.34 lower to 0.87 higher)  Trivial magnitude against (small against to trivial in favour) | Very low |
| **Withdrawals due to adverse events at study end (time of measurement: 4 weeks)** | | | | | | | | | | | |
| 1 | Randomised controlled trial | Very serious^1^ | NA | Not serious | Very serious^4^ | NA | 0/31 | 0/29 | Not estimable | Not estimable | Very low |
| **Stool consistency - number of patients with hard stools (time of measurement: 4 weeks) – incompatible outcome** | | | | | | | | | | | |
| 1 | Randomised controlled trial | Very serious^1^ | NA | Not serious | Very serious^4^ | NA | 7/31 | 2/29 | RR: 3.27 (0.74 to 14.49) | 157 more per 1000 (from 18 less to 930 more per 1000)  Moderate magnitude against (trivial in favour to large against) | Very low |
| **Painful defecation – number of patients with painful defecation (time of measurement: 4 weeks) – incompatible outcome** | | | | | | | | | | | |
| 1 | Randomised controlled trial | Very serious^1^ | NA | Not serious | Very serious^4^ | NA | 3/31 | 2/29 | RR: 1.40 (0.25 to 7.81) | 28 more per 1000 (from 52 less to 470 more per 1000)  Trivial magnitude against (trivial in favour to large against) | Very low |
| **Painful defecation - number of painless bowel movements per week (time of measurement: 3 weeks)** | | | | | | | | | | | |
| 1 | Randomised controlled trial | Very serious^1^ | NA | Not serious | Serious^5^ | NA | N = 36 | N = 36 | MD: -0.89 (-1.43 to -0.35) | 0.89 lower (from 1.43 lower to 0.35 lower)  Trivial magnitude against (small against to trivial against) | Low |
| **Abdominal pain – number of patients with abdominal pain (time of measurement: 4 weeks) – incompatible outcome** | | | | | | | | | | | |
| 1 | Randomised controlled trial | Very serious^1^ | Not serious | Not serious | Very serious^4^ | NA | 2/31 | 4/29 | RR: 0.47 (0.09 to 2.36) | 73 less per 1000 (from 126 less to 188 more per 1000)  Trivial magnitude in favour (small in favour to moderate against) | Very low |
| **Faecal incontinence – number faecal incontinence episodes per week (time of measurement: 4 weeks) – incompatible outcome** | | | | | | | | | | | |
| 1 | Randomised controlled trial | Very serious^1^ | Not serious | Not serious | Not serious | NA | N = 31 | N = 29 | MD: -0.18 (-0.66 to 0.30) | MD: 0.18 less (from 0.66 less to 0.30 more)  Trivial magnitude in favour (trivial in favour to trivial against) | Low |

^1^Downdgraded two levels due to unclear method of randomisation, allocation and blinding

^2^Downdgraded two levels due to unclear method of randomisation, allocation, blinding attrition and selective outcome reporting

^3^Downgraded two levels due to very serious inconsistency, I^2^ = 86%

^4^Downgraded two levels due to very serious imprecision

^5^Downgraded one level due to significant imprecision

*Forest plot synbiotics vs. laxative: treatment success*

*Forest plot synbiotics vs. laxative: defecation frequency per week*

*Forest plot synbiotics vs. laxative: withdrawals due to adverse events at study end*

3. Additional effect: synbiotics + laxative vs laxative (N=1)

**Question:** Should synbiotics as addition to laxatives vs laxatives alone be used for functional constipation? **Bibliography:** Khodadad 2010

GRADE

| **Quality assessment** | | | | | | | **No of patients** | | **Effect** | | **Quality** | **Importance** |
| --- | --- | --- | --- | --- | --- | --- | --- | --- | --- | --- | --- | --- |
|  |  |  |  |  |  |  |  |  |  |  |  |  |
| **No of studies** | **Design** | **Risk of bias** | **Inconsistency** | **Indirectness** | **Imprecision** | **Other considerations** | **Synbiotics + laxative** | **Laxative** | **Relative (95% CI)** | **Absolute** |  |  |
| **Treatment success (time of measurement: 4 weeks)** | | | | | | | | | | | | |
| 1 | Randomised trials | Very serious^1^ | Not serious | Not serious | Very serious^2^ | Not serious | 28/37 | 24/29 | RR: 0.91 (0.71 to 1.17) | 74 less per 1000 (from 240 less to 141 more per 1000)  Small magnitude against (large against to moderate in favour) | Very low |  |
| **Withdrawals due to adverse events (time of measurement: 4 weeks)** | | | | | | | | | | | | |
| 1 | Randomised trials | Very serious^1^ | Not serious | Not serious | Very serious^2^ | Not serious | 0/37 | 0/29 | Not estimable | Not estimable | Very low |  |
| **Defecation frequency per week (time of measurement: 4 weeks)** | | | | | | | | | | | | |
| 1 | Randomised trials | Very serious^1^ | Not serious | Not serious | Not serious | Not serious | N=37 | N=29 |  | MD: 0.02 higher (from 0.56 lower to 0.60 higher)  Trivial magnitude in favour (trivial against to trivial in favour) | Low |  |
| **Painful defecation – number of patients (time of measurement: 4 weeks) – incompatible outcome** | | | | | | | | | | | | |
| 1 | Randomised trials | Very serious^1^ | Not serious | Not serious | Very serious^2^ | Not serious | 4/37 | 2/29 | RR: 1.57 (0.31 to 7.97) | 39 more per 1000 (from 48 less to 481 more per 1000) | Very low |  |
| **Stool consistency: Number of patients with hard stools (time of measurement: 4 weeks) – incompatible outcome** | | | | | | | | | | | | |
| 1 | Randomised trials | Very serious^1^ | Not serious | Not serious | Very serious^2^ | Not serious | 4/37 | 2/29 | RR: 1.57 (0.31 to 7.97) | 39 more per 1000 (from 48 less to 481 more per 1000) | Very low |  |
| **Fecal incontinence – number of patients (time of measurement: 4 weeks) – incompatible outcome** | | | | | | | | | | | | |
| 1 | Randomised trials | Very serious^1^ | Not serious | Not serious | Very serious^2^ | Not serious | N=37 | N=29 |  | MD: not estimable | Very low |  |
| **Abdominal pain – number of patients (time of measurement: 4 weeks) – incompatible outcome** | | | | | | | | | | | | |
| 1 | Randomised trials | Very serious^1^ | Not serious | Not serious | Very serious^2^ | Not serious | 5/37 | 4/29 | RR: 0.98 (0.29 to 3.32) | 3 less per 1000 (from 98 less to 320 more per 1000) | Very low |  |

^1^Downdgraded two levels due to unclear method of randomization, allocation and blinding
^2^Downgraded two levels due to very serious imprecision

*Synbiotics + laxatives vs laxatives defecation frequency*

Biofeedback

1. Additional effect: biofeedback + laxative vs laxative (N=3)

**Question:** Should biofeedback as addition to a laxative vs laxative only be used for functional constipation? **Bibliography:** Loening-Baucke 1990, Sunic-Omejc 2002, Van der Plas 1996

GRADE

| **Quality assessment** | | | | | | | **No of patients** | | **Effect** | | **Certainty** | **Importance** |
| --- | --- | --- | --- | --- | --- | --- | --- | --- | --- | --- | --- | --- |
|  |  |  |  |  |  |  |  |  |  |  |  |  |
| **No of studies** | **Design** | **Risk of bias** | **Inconsistency** | **Indirectness** | **Imprecision** | **Other considerations** | **Biofeedback + laxative** | **Laxative** | **Relative (95% CI)** | **Absolute** |  |  |
| **Treatment success (time of measurement range: 6 weeks to 7 months)** | | | | | | | | | | | | |
| 3 | Randomsied controlled trials | Very serious^1^ | Not serious | Not serious | Very serious^2^ | Not serious | 64/146 | 47/138 | RR: 1.36 (0.75 to 2.47) | 122 more per 1000 (from 85 less to 500 more per 100)  Small magnitude in favour (small in against to large in favour) | Very low |  |
| **Withdrawals due to Adverse Events at study end (time of measurement range: 12 weeks to 18 months)** | | | | | | | | | | | | |
| 3 | Randomsied controlled trials | Very serious^1^ | Not serious | Not serious | Very serious^3^ | Not serious | 6/146 | 2/138 | RR: 2.44 (0.58 to 10.28) | 21 more per 1000 (from 6 less to 134 more per 100)  Moderate magnitude against (small in favour to large against) | Very low |  |
| **Defecation frequency per week: not reported** | | | | | | | | | | | | |
|  |  |  |  |  |  |  |  |  |  |  |  |  |
| **Fecal incontinence frequency per week (time of measurement: 7 months)** | | | | | | | | | | | | |
| 1 | Randomsied controlled trials | Very serious^1^ | Not serious | Not serious | Very serious^3^ | Not serious | N=22 | N=19 |  | 2.00 lower (from 4.73 lower to 0.73 higher)  Small magnitude in favour (large in favour to trivial against) | Very low |  |

^1^Downgraded two levels due to unclear randomization methods, allocation methods and unclear or high risk selective reporting
^2^Downgraded one level due to significant imprecision
^3^Downgraded two levels due to serious imprecision

*Forest plot Biofeedback + laxative vs laxative Treatment Success*

*Forest plot Biofeedback + laxative vs laxative Withdrawals due to Adverse Events*

2. Biofeedback vs no biofeedback (N=1)

**Notes**: no description of the control group, just ‘no biofeedback’
**Question:** Should biofeedback vs no biofeedback be used for functional constipation?
**Bibliography:** Castilla 2021 (abstract only)

GRADE

| **Quality assessment** | | | | | | | **No of patients** | | **Effect** | | **Certainty** | **Importance** |
| --- | --- | --- | --- | --- | --- | --- | --- | --- | --- | --- | --- | --- |
|  |  |  |  |  |  |  |  |  |  |  |  |  |
| **No of studies** | **Design** | **Risk of bias** | **Inconsistency** | **Indirectness** | **Imprecision** | **Other considerations** | **Biofeedback** | **No biofeedback** | **Relative (95% CI)** | **Absolute** |  |  |
| **Treatment success (time of measurement range: unclear)** | | | | | | | | | | | | |
| 1 | Randomised controlled trials | Very serious^1^ | Not serious | Not serious | Very serious^2^ | Not serious | 10/12 | 4/12 | RR: 2.50 (1.08 to 5.79) | 500 more per 1000 (from 27 more to 1000 per 1000 more)  Large magnitude in favour (trivial in favour to large in favour) | Very low |  |
| **Withdrawals due to Adverse Events at study end – not reported** | | | | | | | | | | | | |
|  |  |  |  |  |  |  |  |  |  |  |  |  |
| **Defecation frequency per week – not reported** | | | | | | | | | | | | |
|  |  |  |  |  |  |  |  |  |  |  |  |  |

^1^Downgraded two levels due to unclear randomization methods, allocation concealment, blinding and selective reporting
^2^Downgraded wo levels due to serious imprecision

3. Biofeedback at home + laboratory vs biofeedback in laboratory (N=1)

**Question:** Should biofeedback at home in addition to laboratory feedback vs biofeedback in laboratory only be used for functional constipation? **Bibliography:** Croffie 2005

GRADE

| **Quality assessment** | | | | | | | **No of patients** | | **Effect** | | **Certainty** | **Importance** |
| --- | --- | --- | --- | --- | --- | --- | --- | --- | --- | --- | --- | --- |
|  |  |  |  |  |  |  |  |  |  |  |  |  |
| **No of studies** | **Design** | **Risk of bias** | **Inconsistency** | **Indirectness** | **Imprecision** | **Other considerations** | **Biofeedback at home + laboratory** | **Laboratory** | **Relative (95% CI)** | **Absolute** |  |  |
| **Treatment success (time of measurement: 4 months)** | | | | | | | | | | | | |
| 1 | Randomised controlled trials | Very serious^1^ | Not serious | Not serious | Very serious^2^ | Not serious | 10/12 | 22/24 | RR: 0.91 (0.69 to 1.20) | 83 less per 1000 (284 less to 183 more per 1000)  Small magnitude against (large against to moderate in favour) | Very low |  |
| **Withdrawals due to Adverse Events at study end – not reported** | | | | | | | | | | | | |
| 1 |  |  |  |  |  |  |  |  |  |  |  |  |
| **Defecation frequency per week: not reported – not adequately reported** | | | | | | | | | | | | |
| 1 |  |  |  |  |  |  |  |  |  |  |  |  |
| **Fecal incontinence – not adequately reported** | | | | | | | | | | | | |
| 1 |  |  |  |  |  |  |  |  |  |  |  |  |

^1^Downgraded two levels due to unclear randomization methods, allocation concealment and selective reporting
^2^Downgraded two levels due to serious imprecision

Nerve stimulation

1. Parasacral transcutaneous electrical nerve stimulation vs sham therapy (N=1)

**Notes:** Treatment with parasacral transcutaneous electrical nerve stimulation (PTENS) consisted of 20 minute sessions, 3 times a week for a total of 20 sessions. The control group received sham therapy. Both groups received standard urotherapy, because the included patients were diagnosed with functional constipation associated with lower urinary tract symptoms (LUTS). **Question:** Should parasascral transcutaneous electrical nerve stimulation vs sham therapy be used for functional constipation? **Bibliography**: de Abreu 2021

GRADE

| **Quality assessment** | | | | | | | **No of patients** | | **Effect** | | **Certainty** | **Importance** |
| --- | --- | --- | --- | --- | --- | --- | --- | --- | --- | --- | --- | --- |
|  |  |  |  |  |  |  |  |  |  |  |  |  |
| **No of studies** | **Design** | **Risk of bias** | **Inconsistency** | **Indirectness** | **Imprecision** | **Other considerations** | **PTENS** | **Sham** | **Relative (95% CI)** | **Absolute** |  |  |
| **Treatment success (time of measurement: 7-9 weeks)** | | | | | | | | | | | | |
| 1 | Randomised controlled trial | Very serious^2^ | Not serious | Not serious | Not serious | Not serious | 16/20 | 6/20 | RR: 2.67 (1.32 to 5.39)  NNT: 2 (1 to 10) | 501 more per 1000 (from 96 more to 1000 more per 1000)  Large magnitude in favour (small in favour to large in favour) | Low |  |
| **Withdrawals due to Adverse Events at study end (time of measurement: 7-9 weeks)** | | | | | | | | | | | | |
| 1 | Randomised controlled trial | Very serious^2^ | Not serious | Not serious | Very serious^2^ | Not serious | 0/20 | 0/20 | Not estimable | Not estimable | Very low |  |
| **Defecation frequency - dichotomous: two or more bowel movements per week (time of measurement: 7-9 weeks)** | | | | | | | | | | | | |
| 1 | Randomised controlled trial | Very serious^2^ | Not serious | Not serious | Very serious^2^ | Not serious | 19/20 | 13/20 | RR: 1.46 (1.04 to 2.05)  NNT: 3 (1 to 38) | 299 more per 1000 (from 26 more to 683 more per 1000)  Large magnitude in favour (trivial in favour to large in favour) | Very low |  |
| **Painful defecation - Number of patients with pain/straining during defecation after treatment (time of measurement: 7-9 weeks) – incompatible outcome** | | | | | | | | | | | | |
| 1 | Randomised controlled trial | Very serious^2^ | Not serious | Not serious | Very serious^2^ | Not serious | 6/20 | 10/20 | RR: 0.60 (0.27 to 1.34) | 200 less per 1000 (from 365 less to 170 more per 1000) | Very low |  |
| **Stool consistency - number of patients with stool type 1 or 2 on Bristol Stool Scale (hard) after treatment (time of measurement: 7-9 weeks) – incompatible outcome** | | | | | | | | | | | | |
| 1 | Randomised controlled trial | Very serious^2^ | Not serious | Not serious | Very serious^2^ | Not serious | 5/20 | 10/20 | RR: 0.50 (0.21 to 1.20) | 250 less per 1000 (from 395 less to 100 more per 1000) | Very low |  |
| **Fecal incontinence - Episode of fecal incontinence after treatment (time of measurement: 7-9 weeks) – incompatible outcome** | | | | | | | | | | | | |
| 1 | Randomised controlled trial | Very serious^2^ | Not serious | Not serious | Very serious^2^ | Not serious | 2/20 | 4/20 | RR: 0.50 (0.10 to 2.43) | 100 less per 1000 (from 180 less to 286 more per 1000) | Very low |  |

^1^Downgraded one level due to selective reporting, because no safety outcome was reported
^2^Downgraded two levels due to very serious imprecision

*Parasacral nerve stimulation vs sham treatment success*

*Parasacral nerve stimulation vs sham defecation frequency (dichotomous)*

2. Abdominal transcutaneous electrical stimulation vs sham therapy (N=1)

**Notes:** Children were diagnosed with FC based on the Rome II criteria and diagnosed with slow transit constipation. Treatment consisted of 12x20 minutes session of interferential electrical stimulation. **Question:** Should abdominal transcutaneous electrical stimulation vs sham therapy be used for functional constipation? **Bibliography**: Clarke 2009

GRADE

| **Quality assessment** | | | | | | | **No of patients** | | **Effect** | | **Certainty** | **Importance** |
| --- | --- | --- | --- | --- | --- | --- | --- | --- | --- | --- | --- | --- |
|  |  |  |  |  |  |  |  |  |  |  |  |  |
| **No of studies** | **Design** | **Risk of bias** | **Inconsistency** | **Indirectness** | **Imprecision** | **Other considerations** | **Abdominal transcutaneous electrical stimulation** | **Sham therapy** | **Relative (95% CI)** | **Absolute** |  |  |
| **Treatment success - not reported** | | | | | | | | | | | | |
|  |  |  |  |  |  |  |  |  |  |  |  |  |
| **Withdrawals due to Adverse Events at study end – not reported** | | | | | | | | | | | | |
|  |  |  |  |  |  |  |  |  |  |  |  |  |
| **Defecation frequency per week – not reported** | | | | | | | | | | | | |
|  |  |  |  |  |  |  |  |  |  |  |  |  |
| **Quality of life measured with PedsQL – not adequately reported** | | | | | | | | | | | | |
| 1 |  |  |  |  |  |  |  |  |  |  |  |  |

3. Additional effect: abdominal transcutaneous electrical stimulation + pelvic floor muscle exercises (PFME) vs PFME (N=2)

**Notes:** In both studies all patients were asked to repeat the PFME each day at home under parental supervision. Sharifi-Rad used sham therapy + PFME as control.
**Question:** Should abdominal transcutaneous electrical stimulation as addition to pelvic floor muscle exercises vs pelvic floor muscle exercises only be used for functional constipation?
**Bibliography**: Ladi-Seyedian 2020, Sharifi-Rad 2018

GRADE

| **Quality assessment** | | | | | | | **No of patients** | | **Effect** | | **Certainty** | **Importance** |
| --- | --- | --- | --- | --- | --- | --- | --- | --- | --- | --- | --- | --- |
|  |  |  |  |  |  |  |  |  |  |  |  |  |
| **No of studies** | **Design** | **Risk of bias** | **Inconsistency** | **Indirectness** | **Imprecision** | **Other considerations** | **Abdominal transcutaneous electrical stimulation + PFME** | **PFME** | **Relative (95% CI)** | **Absolute** |  |  |
| **Treatment success (time of measurement: 6 months)** | | | | | | | | | | | | |
| 2 | Randomised controlled trials | Serious^1^ | Not serious | Not serious | Not serious | Not serious | 45/62 | 25/62 | RR: 1.75 (1.25 to 2.44)  NNT: 3 (2-10) | 302 more per 1000 (from 101 more to 581 more per 1000)  Large magnitude in favour (small in favour to large in favour) | Moderate |  |
| **Withdrawals due to Adverse Events at study end (time of measurement: 6 months)** | | | | | | | | | | | | |
| 2 | Randomised controlled trials | Serious^1^ | Not serious | Not serious | Very serious^3^ | Not serious | 0/62 | 1/62 | RR: 0.33 (0.01 to 7.97) | 11 less per 1000 (from 16 less to 112 more per 1000)  Small magnitude in favour (moderate in favour to large against) | Very low |  |
| **Defecation frequency per week (time of measurement: 6 months)** | | | | | | | | | | | | |
| 2 | Randomised controlled trials | Serious | Not serious | Not serious | Not serious | Not serious | N=62 | N=61 |  | MD: 1.85 stools more per week (1.28 stools more per week to 2.43 stools more per week)  Small magnitude in favour (small in favour to moderate in favour) | Moderate |  |
| **Painful defecation – number of patients with painful defecation (time of measurement: 6 months) – incompatible outcome** | | | | | | | | | | | | |
| 1 | Randomised controlled trials | Very serious^1^ | Not serious | Not serious | Very serious^3^ | Not serious | 2/17 | 6/17 | RR: 0.33 (0.08 to 1.42) | 236 less per 1000 (from 311 less to 64 more per 1000) | Very low |  |
| **Stool consistency – number of patients with abnormal stool form (time of measurement: 6 months) – incompatible outcome** | | | | | | | | | | | | |
| 1 | Randomised controlled trials | Very serious^1^ | Not serious | Not serious | Very serious^3^ | Not serious | 3/17 | 8/17 | RR: 0.38 (0.12 to 1.18) | 292 less per 1000 (from 414 less to 85 more per 1000) | Very low |  |
| **Fecal incontinence – number of patients with fecal soiling (time of measurement: 6 months) – incompatible outcome** | | | | | | | | | | | | |
| 2 | Randomised controlled trials | Serious^1^ | Not serious | Not serious | Very serious^3^ | Not serious | 12/62 | 28/61 | RR: 0.43 (0.25 to 0.73) | 262 less per 1000 (from 124 less to 344 less per 1000) | Very low |  |
| **Adverse events (time of measurement: 6 months)** | | | | | | | | | | | | |
| 2 | Randomised controlled trials | Serious^1^ | Not serious | Not serious | Very serious^3^ | Not serious | 0/62 | 0/62 | RR: not estimable | Not estimable | Very low |  |

^1^Downgraded one level due to unclear allocation concealment and unclear risk of selective reporting
^2^Downgraded one level due to significant imprecision
^3^Downgraded two levels due to serious imprecision

*Forest plot abdominal transcutaneous electrical stimulation + PFME vs PFME treatment success*

*Forest plot abdominal transcutaneous electrical stimulation + PFME vs PFME withdrawals due to AE*

*Forest plot abdominal transcutaneous electrical stimulation + PFME vs PFME defecation frequency*

4. Additional effect: abdominal transcutaneous electrical stimulation + standard therapy vs standard therapy (N=1)

**Notes:** treatment duration of 10 days, follow-up of 3, 6 and 12 months after treatment. Unclear at which time point the reported results are measured. Standard therapy consisted of: laxative diet, probiotics, choleretic drugs, enzymes **Question:** Should abdominal transcutaneous electrical stimulation as addition to standard therapy vs standard therapy only be used for functional constipation? **Bibliography**: Khan 2020

GRADE

| **Quality assessment** | | | | | | | **No of patients** | | **Effect** | | **Certainty** | **Importance** |
| --- | --- | --- | --- | --- | --- | --- | --- | --- | --- | --- | --- | --- |
|  |  |  |  |  |  |  |  |  |  |  |  |  |
| **No of studies** | **Design** | **Risk of bias** | **Inconsistency** | **Indirectness** | **Imprecision** | **Other considerations** | **Abdominal transcutaneous electrical stimulation + standard therapy** | **Standard therapy** | **Relative (95% CI)** | **Absolute** |  |  |
| **Treatment success (time of measurement: unclear)** | | | | | | | | | | | | |
| 1 | Randomised controlled trials | Very serious^1^ | Not serious | Not serious | Very serious^2^ | Not serious | 13/20 | 10/20 | RR: 1.30 (0.75 to 2.24) | 150 more per 1000 (from 125 less to 620 more per 1000)  Moderate magnitude in favour (small against to large in favour) | Very low |  |
| **Withdrawals due to Adverse Events at study end – not reported** | | | | | | | | | | | | |
|  |  |  |  |  |  |  |  |  |  |  |  |  |
| **Defecation frequency per week – not reported** | | | | | | | | | | | | |
|  |  |  |  |  |  |  |  |  |  |  |  |  |
| **Painful defecation – number of patients (time of measurement: unclear) – incompatible outcome** | | | | | | | | | | | | |
| 1 | Randomised controlled trials | Very serious^1^ | Not serious | Not serious | Very serious^2^ | Not serious | 0/20 | 0/20 | Not estimable | Not estimable | Very low |  |
| **Fecal incontinence – number of patients (time of measurement: unclear) – incompatible outcome** | | | | | | | | | | | | |
| 1 | Randomised controlled trials | Very serious^1^ | Not serious | Not serious | Very serious^2^ | Not serious | 4/20 | 6/20 | RR: 0.67 (0.22 to 2.01) | 99 less per 1000 (from 234 less to 303 more per 1000) | Very low |  |
| **Abdominal pain – number of patients (time of measurement: unclear) – incompatible outcome** | | | | | | | | | | | | |
| 1 | Randomised controlled trials | Very serious^1^ | Not serious | Not serious | Very serious^2^ | Not serious | 3/20 | 5/20 | RR: 0.60 (0.17 to 2.18) | 100 less per 1000 (from 208 less to 295 more per 1000) | Very low |  |

^1^Downgraded two levels due to unclear method of allocation concealment, and unclear risk of attrition and selective reporting bias
^2^Downgraded two levels due to very serious imprecision

*Abdominal transcutaneous electrical stimulation + standard therapy vs standard therapy treatment success*

5. Additional effect: percutaneous tibial nerve stimulation + pelvic floor exercises (PFE) vs sham + PFE (N=1)

**Notes:** intervention group received percutaneous Tibial Nerve Stimulation (PTNS) with PFE twice daily for 4 weeks. Control group received sham PTNS + PFE twice daily for 4 weeks. PFE was performed using an electromyography biofeedback method, in which an electrode is inserted through the anus.^1^ 20-40 hours of progressive resistance training. These hours would be best spread over 4 weeks, with 15 minutes of exercises twice per day.
**Question:** Should tibial nerve stimulation as an addition to pelvic floor exercises vs pelvic floor exercises be used for functional constipation?
**Bibliography**: Yu 2023

GRADE

| **Quality assessment** | | | | | | | **No of patients** | | **Effect** | | **Certainty** | **Importance** |
| --- | --- | --- | --- | --- | --- | --- | --- | --- | --- | --- | --- | --- |
|  |  |  |  |  |  |  |  |  |  |  |  |  |
| **No of studies** | **Design** | **Risk of bias** | **Inconsistency** | **Indirectness** | **Imprecision** | **Other considerations** | **Tibial nerve stimulation + PFE** | **PFE** | **Relative (95% CI)** | **Absolute** |  |  |
| **Treatment success (time of measurement: 16 weeks)** | | | | | | | | | | | | |
| 1 | Randomised controlled trial | Not serious | Not serious | Not serious | Very serious^2^ | Not serious | 26/42 | 15/42 | RR: 1.73 (1.08 to 2.77)  NNT: 4 (2 to 35) | 261 more per 1000 (from 29 more to 632 more per 1000)  Large magnitude in favour (trivial in favour to large in favour) | Low |  |
| **Withdrawals due to Adverse Events at study end (time of measurement: 16 weeks)** | | | | | | | | | | | | |
| 1 | Randomised controlled trial | Not serious | Not serious | Not serious | Very serious^2^ | Not serious | 3/42 | 3/42 | RR: 1.00 (0.21 to 4.67) | 0 more per 1000 (from 56 less to 262 more per 1000)  No effect (large in favour to large against) | Low |  |
| **Defecation frequency - changes in SBM* per week from baseline (time of measurement: 16 weeks)** | | | | | | | | | | | | |
| 1 | Randomised controlled trial | Not serious | Not serious | Not serious | Serious^3^ | Not serious | N=42 | N=42 |  | MD: 1.82 higher (from 0.82 higher to 2.82 higher)  Small magnitude in favour (trivial in favour to moderate in favour) | Moderate |  |
| **Painful or hard defecation – dichotomous: number of children with painful or hard defecation (time of measurement: 16 weeks) – incompatible outcome** | | | | | | | | | | | | |
| 1 | Randomised controlled trial | Not serious | Not serious | Not serious | Very serious^2^ | Not serious | 9/42 | 18/42 | RR: 0.50 (0.25 to 0.98) | 214 less per 1000 (from 9 less to 321 less per 1000) | low |  |
| **Fecal incontinence - dichotomous: number of children with encopresis (time of measurement: 16 weeks) – incompatible outcome** | | | | | | | | | | | | |
| 1 | Randomised controlled trial | Not serious | Not serious | Not serious | Serious^3^ | Not serious | 6/42 | 19/42 | 0.32 (0.14 to 0.71) | 308 less per 1000 (from 131 less to 389 less per 1000) | Moderate |  |
| **Serious adverse events (time of measurement: 16 weeks)** | | | | | | | | | | | | |
| 1 | Randomised controlled trial | Not serious | Not serious | Not serious | Very serious^2^ | Not serious | 0/42 | 0/42 | Not estimable | Not estimable | Low |  |
| **Adverse events (time of measurement: 16 weeks)** | | | | | | | | | | | | |
| 1 | Randomised controlled trial | Not serious | Not serious | Not serious | Very serious^2^ | Not serious | 3/42 | 4/42 | RR: 0.75 (0.18 to 3.15) | 24 less per 1000 (from 78 less to 205 more per 1000)  Trivial magnitude in favour (moderate in favour to large against) | Low |  |

^1^Very different way of pelvic floor muscle exercises from how pelvic floor muscle exercises as described in the rest of the literature.
^2^Downgraded two levels due to very serious imprecision
^3^Downgraded one level due to significant imprecision
*SBM: spontaneous bowel movements

*PTNS + PFME vs PFME Treatment success*

*PTNS + PFME vs PFME Defecation frequency*

*PTNS + PFME vs PFME Withdrawals due to adverse events*

Cow’s milk free diet

1. Cow’s milk free diet vs cow’s milk diet (N=1)

**Question:** Should cow’s milk free diet vs cow’s milk diet be used for treatment of functional constipation? **Bibliography**: Iacono 1998

GRADE

| **Quality assessment** | | | | | | | **No of patients** | | **Effect** | | **Certainty** | **Importance** |
| --- | --- | --- | --- | --- | --- | --- | --- | --- | --- | --- | --- | --- |
|  |  |  |  |  |  |  |  |  |  |  |  |  |
| **No of studies** | **Design** | **Risk of bias** | **Inconsistency** | **Indirectness** | **Imprecision** | **Other considerations** | **Cow’s milk free diet** | **Cow’s milk diet** | **Relative (95% CI)** | **Absolute** |  |  |
| **Treatment success – not adequately reported** | | | | | | | | | | | | |
|  |  |  |  |  |  |  |  |  |  |  |  |  |
| **Withdrawals due to Adverse Events at study end (time of measurements range: 2 to 4 weeks)** | | | | | | | | | | | | |
| 1 | Randomised controlled trials | Very serious^1^ | Not serious | Not serious | Very serious^2^ | Not serious | 0/32 | 0/32 | Not estimable | Not estimable | Very low |  |
| **Defecation frequency – not adequately reported** | | | | | | | | | | | | |
|  |  |  |  |  |  |  |  |  |  |  |  |  |

^1^Downgraded two levels due to lack of blinding, unclear allocation concealment and unclear selective reporting
^2^Downgraded two levels due to significant imprecision

2. Additional effect: Cow’s milk free diet + laxative vs cow’s milk diet + laxative (N=2)

**Question:** Should Cow’s milk free diet as addition to laxative vs cow’s milk diet and laxative be used for treatment of functional constipation?
**Notes**: Children were treated with the osmotic laxative PEG in both groups. 1 g/kg/day in Bourkheili 2021 and 0.5 g/kg/day in Dehghani 2012. **Bibliography**: Bourkheili 2021, Dehghani 2012

GRADE

| **Quality assessment** | | | | | | | **No of patients** | | **Effect** | | **Certainty** | **Importance** |
| --- | --- | --- | --- | --- | --- | --- | --- | --- | --- | --- | --- | --- |
|  |  |  |  |  |  |  |  |  |  |  |  |  |
| **No of studies** | **Design** | **Risk of bias** | **Inconsistency** | **Indirectness** | **Imprecision** | **Other considerations** | **Cow’s milk free diet + PEG** | **Cow’s milk diet + PEG** | **Relative (95% CI)** | **Absolute** |  |  |
| **Treatment success (time of measurement: 4 weeks)** | | | | | | | | | | | | |
| 2 | Randomised controlled trials | Very serious^1^ | Very serious^2^ | Not serious | Very serious^3^ | Not serious | 81/105 | 37/106 | RR: 3.09 (0.75 to 12.78) | 730 more per 1000 (from 87 less to 1000 more per 1000)  Large magnitude in favour (small against to large in favour) | Very low |  |
| **Withdrawals due to Adverse Events (time of measurement: 4 weeks)** | | | | | | | | | | | | |
| 1 | Randomised controlled trials | Very serious^1^ | Not serious | Not serious | Very serious^3^ | Not serious | 0/35 | 1/36 | RR: 0.34 (0.01 to 8.14) | 18 less per 1000 (from 28 less to 198 more per 1000)  Moderate magnitude in favour (large in favour to large against) | Very low |  |
| **Defecation frequency per week – dichotomous: Number of patients with 3 or more defecations per week (time of measurement: 4 weeks)** | | | | | | | | | | | | |
| 1 | Randomised controlled trials | Very serious^1^ | Not serious | Not serious | Not serious | Not serious | 67/70 | 50/70 | RR: 1.34 (1.15 to 1.57)  NNT: 4 (2 to 9) | 243 more per 1000 (from 107 more to 407 more per 1000)  Moderate magnitude in favour (small in favour to large in favour) | Low |  |
| **Stool consistency/painful defecation: number of patients with painful or hard bowel movements (time of measurement: 4 weeks) – incompatible outcome** | | | | | | | | | | | | |
| 1 | Randomised controlled trials | Very serious^1^ | Not serious | Not serious | Not serious | Not serious | 9/70 | 32/70 | RR: 0.28 (0.15 to 0.54)  NNT: 3 (3 to 5) | 329 less per 1000 (from 210 less to 389 less per 1000) | Low |  |
| **Fecal incontinence – number of patients (time of measurement: 4 weeks) – incompatible outcome** | | | | | | | | | | | | |
| 1 | Randomised controlled trials | Very serious^1^ | Not serious | Not serious | Serious^4^ | Not serious | 2/70 | 15/70 | RR: 0.13 (0.03 to 0.56)  NNT: 5 (5 to 11) | 186 less per 1000 (from 94 less to 208 less per 1000) | Very low |  |

^1^Downgraded two levels due to unclear allocation concealment and selective outcome reporting, and high-risk due to unblinding and selective outcome reporting
^2^Downgraded two levels due to very serious inconsistency
^3^Downgraded two levels due to very serious imprecision
^4^Downgraded one level due to significant imprecision

*Cow’s milk free diet + laxative vs cow’s milk diet + laxative Treatment success*

*Cow’s milk free diet + laxative vs cow’s milk diet + laxative Defecation frequency*

*Cow’s milk free diet + laxative vs cow’s milk diet + laxative withdrawals due to adverse events*

3. Formula with hydrolyzed protein + prebiotics vs formula with cow milk + prebiotics (N=1)

**Notes:** included children aged 28– 300 days old (4 weeks – 43 weeks) **Question:** Should formula with hydrolyzed protein vs formula with cow milk in addition to prebiotics be used for the treatment of functional constipation  **Bibliography**: Fabrizio 2022

GRADE

| **Quality assessment** | | | | | | | **No of patients** | | **Effect** | | **Certainty** | **Importance** |
| --- | --- | --- | --- | --- | --- | --- | --- | --- | --- | --- | --- | --- |
|  |  |  |  |  |  |  |  |  |  |  |  |  |
| **No of studies** | **Design** | **Risk of bias** | **Inconsistency** | **Indirectness** | **Imprecision** | **Other considerations** | **Hydrolyzed protein + prebiotics** | **Cow milk + prebiotics** | **Relative (95% CI)** | **Absolute** |  |  |
| **Treatment success – not reported** | | | | | | | | | | | | |
|  |  |  |  |  |  |  |  |  |  |  |  |  |
| **Withdrawals due to Adverse Events (time of measurement: 2 weeks)** | | | | | | | | | | | | |
| 1 | Randomised controlled trials | Very serious^1^ | Not serious | Not serious | Very serious^2^ | Serious^3^ | 7/42 | 4/47 | RR: 1.96 (0.62 to 6.22) | 82 more per 1000 (from 32 less to 444 more per 1000)  Large magnitude against (large in favour to large against) | Very low |  |
| **Defecation frequency per day (time of measurement: 2 weeks)** | | | | | | | | | | | | |
| 1 | Randomised controlled trials | Very serious^1^ | Not serious | Not serious | Not serious | Serious^3^ | N=42 | N=47 |  | MD: 0.50 higher (from 0.22 higher to 0.78 higher)  Moderate magnitude in favour (small in favour to large in favour) | Very low |  |
| **Stool consistency – continuous: scaled as: hard, 1; formed, 2; mushy, 3; unformed or seedy, 4; watery.** | | | | | | | | | | | | |
| 1 | Randomised controlled trials | Very serious^1^ | Not serious | Not serious | Serious^4^ | Serious^3^ | N=42 | N=47 |  | MD: 0.40 higher (from 0.12 higher to 0.68)  Trivial magnitude in favour (trivial in favour to small in favour) | Very low |  |
| **Adverse events (time of measurement: 2 weeks)** | | | | | | | | | | | | |
| 1 | Randomised controlled trials | Very serious^1^ | Not serious | Not serious | Very serious^2^ | Serious^3^ | 14/42 | 8/47 | RR: 1.96 (0.91 to 4.20) | 163 more per 1000 (from 15 less to 545 more per 1000)  Large magnitude against (trivial in favour to large against) | Very low |  |

^1^Downgraded two levels due to unclear selective reporting. No protocol could be found. Requested twice from the corresponding author. No response. Pharma sponsored trial, therefore the study should have been registered and a protocol should be available. Therefore, downgraded twice.
^2^Downgraded twice due to very serious imprecision
^3^Protocol or trial registration number not available. Requested twice from the corresponding author. No response. Pharma sponsored trial, therefore study needs to be registered and a protocol available.
^4^Downgraded one level due to serious imprecision

*Defecation frequency per day*

*Withdrawals due to adverse events*

Behavioral therapy

1. Additional effect: behavioral therapy (BT) + PEG vs PEG (N=1)

**Notes:** Behavioral therapy hypothesis: phobic reactions related to defecation can be reduced and that adequate toileting behavior and appropriate defecation straining can be (re)acquired by teaching parents behavioral procedures and by behavioral play therapy with the child in presence of his or her parents. The intervention period for both conventional therapy (laxatives) and BT consisted of 12 visits during 22 weeks with similar intervals between treatment sessions. Conventional therapy consisted of disimpaction with enemas at start, maintenance PEG and if necessary enema or bisacodyl suppositories.
**Question:** Should behavioral therapy as addition to PEG vs PEG be used for the treatment of functional constipation
**Bibliography**: van Dijk 2008

GRADE

| **Quality assessment** | | | | | | | **No of patients** | | **Effect** | | **Certainty** | **Importance** |
| --- | --- | --- | --- | --- | --- | --- | --- | --- | --- | --- | --- | --- |
|  |  |  |  |  |  |  |  |  |  |  |  |  |
| **No of studies** | **Design** | **Risk of bias** | **Inconsistency** | **Indirectness** | **Imprecision** | **Other considerations** | **Behavioral therapy + PEG** | **PEG** | **Relative (95% CI)** | **Absolute** |  |  |
| **Treatment success (time of measurement: 22 weeks)** | | | | | | | | | | | | |
| 1 | Randomised controlled trials | Not serious | Not serious | Not serious | Very serious^1^ | Not serious | 35/67 | 42/67 | RR: 0.83 (0.62 to 1.12) | 106 less per 1000 (from 238 less to 75 more per 1000)  Small magnitude against (large against to small in favour) | Low |  |
| **Withdrawals due to Adverse Events (time of measurement: 22 weeks)** | | | | | | | | | | | | |
| 1 | Randomised controlled trials | Not serious | Not serious | Not serious | Very serious^1^ | Not serious | 0/67 | 1/67 | RR: 0.33 (0.01 to 8.21) | 10 less per 1000 (from 15 less to 108 more per 1000)  Small magnitude in favour (moderate in favour to large against) | Low |  |
| **Defecation frequency per week (time of measurement: 22 weeks)** | | | | | | | | | | | | |
| 1 | Randomised controlled trials | Not serious | Not serious | Not serious | Serious^2^ | Not serious | N=67 | N=67 |  | MD: 1.80 lower (from 0.72 lower to 2.88 lower)  Small magnitude against (trivial against to moderate against) | Moderate |  |
| **Fecal incontinence frequency per week (time of measurement: 22 weeks)** | | | | | | | | | | | | |
| 1 | Randomised controlled trials | Not serious | Not serious | Not serious | Serious | Not serious | N=67 | N=67 |  | MD: 2.90 higher (from 0.97 higher to 4.83 higher)  Moderate magnitude against (small against to large against) | Moderate |  |

^1^Downgraded two levels due to very serious imprecision
^2^Downgraded one level due to significant imprecision

*Treatment success*

*Defecation frequency*

*Withdrawals due to adverse events*

Cryotherapy

1. Cryotherapy + standard therapy vs abdominal electrical stimulation + standard therapy (N=1)

**Notes:** treatment duration of 10 days, follow-up of 3, 6 and 12 months after treatment. Unclear at which time point the reported results are measured. Standard therapy consisted of: laxative diet, probiotics, choleretic drugs, enzymes **Question:** Should cryotherapy vs abdominal electrical stimulation be used as addition to standard therapy for the treatment of functional constipation  **Bibliography**: Khan 2020

GRADE

| **Quality assessment** | | | | | | | **No of patients** | | **Effect** | | **Certainty** | **Importance** |
| --- | --- | --- | --- | --- | --- | --- | --- | --- | --- | --- | --- | --- |
|  |  |  |  |  |  |  |  |  |  |  |  |  |
| **No of studies** | **Design** | **Risk of bias** | **Inconsistency** | **Indirectness** | **Imprecision** | **Other considerations** | **Cryotherpy + standard therapy** | **Abdominal electrical stimulation + Standard therapy** | **Relative (95% CI)** | **Absolute** |  |  |
| **Treatment success (time of measurement: unclear)** | | | | | | | | | | | | |
| 1 | Randomised controlled trials | Very serious^1^ | Not serious | Not serious | Very serious^2^ | Not serious | 15/20 | 13/20 | RR: 1.15 (0.77 to 1.74) | 98 more per 1000 (from 150 less to 481 more per 1000)  Small magnitude in favour (moderate against to large in favour) | Very low |  |
| **Withdrawals due to Adverse Events at study end – not reported** | | | | | | | | | | | | |
|  |  |  |  |  |  |  |  |  |  |  |  |  |
| **Defecation frequency per week – not reported** | | | | | | | | | | | | |
|  |  |  |  |  |  |  |  |  |  |  |  |  |
| **Painful defecation: number of patients (time of measurement: unclear) – incompatible outcome** | | | | | | | | | | | | |
| 1 | Randomised controlled trials | Very serious^1^ | Not serious | Not serious | Very serious^2^ | Not serious | 0/20 | 0/20 | Not estimable | Not estimable | Very low |  |
| **Fecal incontinence – number of patients (time of measurement: unclear) – incompatible outcome** | | | | | | | | | | | | |
| 1 | Randomised controlled trials | Very serious^1^ | Not serious | Not serious | Very serious^2^ | Not serious | 2/20 | 4/20 | RR: 0.50 (0.10 to 2.43) | 100 less per 1000 (from 180 less to 286 more per 1000) | Very low |  |
| **Abdominal pain: number of patients (time of measurement: unclear) – incompatible outcome** | | | | | | | | | | | | |
| 1 | Randomised controlled trials | Very serious^1^ | Not serious | Not serious | Very serious^2^ | Not serious | 2/20 | 3/20 | RR: 0.67 (0.12 to 3.57) | 50 less per 1000 (from 132 less to 386 more per 1000) | Very low |  |

^1^Downgraded two levels due to unclear method of allocation concealment, and unclear risk of attrition and selective reporting bias
^2^Downgraded two levels due to very serious imprecision

2. Additional effect: cryotherapy + standard therapy vs standard therapy (N=1)

**Notes:** treatment duration of 10 days, follow-up of 3, 6 and 12 months after treatment. Unclear at which time point the reported results are measured. Standard therapy consisted of: laxative diet, probiotics, choleretic drugs, enzymes **Question:** Should cryotherapy ad addition to standard therapy vs standard therapy alone be used for the treatment of functional constipation  **Bibliography**: Khan 2020

GRADE

| **Quality assessment** | | | | | | | **No of patients** | | **Effect** | | **Quality** | **Importance** |
| --- | --- | --- | --- | --- | --- | --- | --- | --- | --- | --- | --- | --- |
|  |  |  |  |  |  |  |  |  |  |  |  |  |
| **No of studies** | **Design** | **Risk of bias** | **Inconsistency** | **Indirectness** | **Imprecision** | **Other considerations** | **Cryotherpy + standard therapy** | **Standard therapy** | **Relative (95% CI)** | **Absolute** |  |  |
| **Treatment success (time of measurement: unclear)** | | | | | | | | | | | | |
| 1 | Randomised controlled trials | Very serious^1^ | Not serious | Not serious | Very serious^2^ | Not serious | 15/20 | 10/20 | RR: 1.50 (0.90 to 2.49) | 250 more per 1000 (from 50 less to 745 more per 100)  Large magnitude in favour (trivial against to large in favour) | Very low |  |
| **Withdrawals due to Adverse Events at study end – not reported** | | | | | | | | | | | | |
|  |  |  |  |  |  |  |  |  |  |  |  |  |
| **Defecation frequency per week – not reported** | | | | | | | | | | | | |
|  |  |  |  |  |  |  |  |  |  |  |  |  |
| **Painful defecation: number of patients (time of measurement: unclear) – incompatible outcome** | | | | | | | | | | | | |
| 1 | Randomised controlled trials | Very serious^1^ | Not serious | Not serious | Very serious^2^ | Not serious | 0/20 | 0/20 | Not estimable | Not estimable | Very low |  |
| **Fecal incontinence: number of patients (time of measurement: unclear) – incompatible outcome** | | | | | | | | | | | | |
| 1 | Randomised controlled trials | Very serious^1^ | Not serious | Not serious | Very serious^2^ | Not serious | 2/20 | 6/20 | RR: 0.33 (0.08 to 1.46) | 201 less per 1000 (from 276 less to 138 more per 1000) | Very low |  |
| **Abdominal pain: number of patients (time of measurement: unclear) – incompatible outcome** | | | | | | | | | | | | |
| 1 | Randomised controlled trials | Very serious^1^ | Not serious | Not serious | Serious^3^ | Not serious | 2/20 | 5/20 | RR: 0.40 (0.09 to 1.83) | 150 less per 1000 (from 228 less to 208 more per 1000) | Very low |  |

^1^Downgraded two levels due to unclear method of allocation concealment, and unclear risk of attrition and selective reporting bias
^2^Downgraded two levels due to very serious imprecision
^2^Downgraded one level due to significant imprecision

Massage

1. Additional effect: abdominal and acupressure point massage + traditional Chinese medicine vs traditional Chinese medicine (N=2)

**Question:** Should abdominal and acupressure point massage as addition to traditional Chinese medicine vs traditional Chinese medicine alone be used for the treatment of functional constipation  **Bibliography**: Mao 2015, Xu 2015

GRADE

| **Quality assessment** | | | | | | | **No of patients** | | **Effect** | | **Certainty** | **Importance** |
| --- | --- | --- | --- | --- | --- | --- | --- | --- | --- | --- | --- | --- |
|  |  |  |  |  |  |  |  |  |  |  |  |  |
| **No of studies** | **Design** | **Risk of bias** | **Inconsistency** | **Indirectness** | **Imprecision** | **Other considerations** | **Abdominal and acupressure point massage + traditional Chinese medicine** | **Chinese medicine** | **Relative (95% CI)** | **Absolute** |  |  |
| **Treatment success (time of measurement: at 2 weeks)** | | | | | | | | | | | | |
| 2 | Randomised controlled trials | Very serious^1^ | Not serious | Not serious | Serious^2^ | Not serious | 20/108 | 13/108 | RR: 1.53 (0.81 to 2.91) | 64 more per 1000 (from 23 less to 230 more per 1000)  Trivial magnitude in favour (trivial against to moderate in favour) | Very low |  |
| **Withdrawals due to Adverse Events at study end – not reported** | | | | | | | | | | | | |
|  |  |  |  |  |  |  |  |  |  |  |  |  |
| **Defecation frequency per week – not reported** | | | | | | | | | | | | |
|  |  |  |  |  |  |  |  |  |  |  |  |  |

^1^Downgraded two levels due to unclear method of randomization and allocation concealment, lack of blinding and unclear risk of all other aspects (studies were translated from Chinese)
^2^Downgraded one level due to significant imprecision

*Forest plot for Treatment success*

2. Additional effect: foot reflexology massage + toilet/diet/motivation training vs toilet/diet/motivation training (N=1)

**Question:** Should foot reflexology massage as addition to toilet/diet/motivation training vs toilet/diet/motivation training alone be used for the treatment of functional constipation  **Bibliography**: Canbulat Sahiner 2017

GRADE

| **Quality assessment** | | | | | | | **No of patients** | | **Effect** | | **Quality** | **Importance** |
| --- | --- | --- | --- | --- | --- | --- | --- | --- | --- | --- | --- | --- |
|  |  |  |  |  |  |  |  |  |  |  |  |  |
| **No of studies** | **Design** | **Risk of bias** | **Inconsistency** | **Indirectness** | **Imprecision** | **Other considerations** | **foot reflexology massage + toilet/diet/motivation training** | **Toilet/diet/motivation training** | **Relative (95% CI)** | **Absolute** |  |  |
| **Treatment success – not reported** | | | | | | | | | | | | |
|  |  |  |  |  |  |  |  |  |  |  |  |  |
| **Withdrawals due to Adverse Events at study end – (time of measurement: 4 weeks)** | | | | | | | | | | | | |
| 1 | Randomised controlled trials | Very serious^1^ | Not serious | Not serious | Very serious^2^ | Not serious | 2/20 | 0/20 | RR: 5.00 (0.26 to 98.00) | 200 more per 1000 (from 37 less to 1000 more per 1000)^3^  Large magnitude against (large in favour to large against) | Very low |  |
| **Defecation frequency per week – Number of patients with more than 2 bowel movements per week (time of measurement: 4 weeks)** | | | | | | | | | | | | |
| 1 | Randomised controlled trials | Very serious^1^ | Not serious | Not serious | Serious^4^ | Not serious | 16/20 | 19/20 | RR: 0.84 (0.66 to 1.07) | 152 less per 1000 (from 324 less to 66 more per 1000)  Moderate magnitude in against (large against to small in favour) | Very low |  |
| **Stool consistency - number of patients with normal or soft stools (time of measurement: 4 weeks)** | | | | | | | | | | | | |
| 1 | Randomised controlled trials | Very serious^1^ | Not serious | Not serious | Serious^4^ | Not serious | 15/20 | 18/20 | RR: 0.83 (0.62 to 1.12) | 153 less per 1000 (from 342 less to 108 more per 1000)  Moderate magnitude in favour (large against small in favour) | Very low |  |

^1^Downgraded two levels due to unclear method of randomization and allocation concealment, lack of blinding and unclear risk of selective reporting
^2^Downgraded two levels due to very serious imprecision
^3^Added a fictional event to the control group in order to calculate absolute number to better interpret the result.
^4^ Downgraded one level due to significant imprecision

Physiotherapy

1. Additional effect: pelvic physiotherapy + standard medical care vs standard medical care (N=1)

**Notes:** Pelvic floor physiotherapy consisted of max 6 sessions in 6 months. Standard medical care consisted of education, demystification, dietary advice, toilet training, keeping track of bladder and bowel diaries, and when needed prescription of PEG. Children from both groups were disimpacted with high dose PEG (1–1.5 g/kg for a maximum of 7 days) if a large fecal mass was present at intake (rectal examination was performed to confirm or exclude FC when only 1 Rome III criterion was met) and the dose of maintenance oral PEG was tailored to the individual patient’s needs (0.3 – 0.8 g/kg per day). PEG was prescribed to 52 of 53 children (98.1%)
**Question:** Should pelvic floor physiotherapy as addition to standard medical care vs standard medical care alone be used for the treatment of functional constipation?
**Bibliography**: Van Engelenburg 2017

GRADE

| **Quality assessment** | | | | | | | **No of patients** | | **Effect** | | **Certainty** | **Importance** |
| --- | --- | --- | --- | --- | --- | --- | --- | --- | --- | --- | --- | --- |
|  |  |  |  |  |  |  |  |  |  |  |  |  |
| **No of studies** | **Design** | **Risk of bias** | **Inconsistency** | **Indirectness** | **Imprecision** | **Other considerations** | **Pelvic physiotherapy + standard medical care** | **Standard medical care** | **Relative (95% CI)** | **Absolute** |  |  |
| **Treatment success (time of measurement: 6 months)** | | | | | | | | | | | | |
| 1 | Randomised controlled trials | Serious^1^ | Not serious | Not serious | Serious^2^ | Not serious | 24/26 | 12/27 | RR: 2.08 (1.34 to 3.21)  NNT 2 (1 – 6) | 480 more per 1000 (from 151 more to 990 more per 1000)  Large magnitude in favour (moderate in favour to large in favour) | Low |  |
| **Withdrawals due to Adverse Events at study end (time of measurement: 6 months)** | | | | | | | | | | | | |
| 1 | Randomised controlled trials | Serious^1^ | Not serious | Not serious | Very serious^3^ | Not serious | 0/26 | 0/27 | Not estimable | Not estimable | Very low |  |
| **Defecation frequency per week – not adequately reported** | | | | | | | | | | | | |
|  |  |  |  |  |  |  |  |  |  |  |  |  |
| **Painful and hard stools – not adequately reported** | | | | | | | | | | | | |
|  |  |  |  |  |  |  |  |  |  |  |  |  |
| **Fecal incontinence – not adequately reported** | | | | | | | | | | | | |
|  |  |  |  |  |  |  |  |  |  |  |  |  |

^1^Downgraded one level due to unclear method of allocation concealment and open label trial
^2^Downgraded one level due to imprecise date, with a range of 84% in treatment success and the lowest threshold very close to the threshold of small effect (14%)
^2^Downgraded two levels due to significant imprecision

*Treatment success*

2. Manual therapy vs laxative (N=1)

**Notes:** Manual physical therapy (MPT) consisted of nine sessions of MPT with a 45-min initial session and 30 min for the rest of sessions distributed weekly during the first and second months and biweekly in the third month. MPT was the same throughout the sessions for all the participants. It was designed and performed by a physiotherapist with 20 years experience in manual physiotherapy, helped by a clinical assistant. The physical treatment was done through direct and indirect articular, vascular, visceral, muscular, and myofascial techniques for the pelvic floor, abdomen (diaphragm, colon, and ileocecal valve, duo- denojejunal flexure), skull, spine (D10–D12), and sacrum, exerting light pressure and vibration, seeking a balance in fascial tensions. It pretended to normalise the neurovegetative, mascular and joint functions.
Control group was kept in the maintenance phase for 2 months until obtaining a regular defecation habit and were treated with PEG followed by a phase of medication withdrawal.
**Question:** Should manual therapy vs laxative be used for functional constipation?
**Bibliography**: Blanco Diaz 2020

GRADE

| **Quality assessment** | | | | | | | **No of patients** | | **Effect** | | **Certainty** | **Importance** |
| --- | --- | --- | --- | --- | --- | --- | --- | --- | --- | --- | --- | --- |
|  |  |  |  |  |  |  |  |  |  |  |  |  |
| **No of studies** | **Design** | **Risk of bias** | **Inconsistency** | **Indirectness** | **Imprecision** | **Other considerations** | **Manual physical therapy** | **Laxative (PEG)** | **Relative (95% CI)** | **Absolute** |  |  |
| **Treatment success (time of measurement range: ) – not reported** | | | | | | | | | | | | |
|  |  |  |  |  |  |  |  |  |  |  |  |  |
| **Withdrawals due to Adverse Events at study end (range: ) – not reported** | | | | | | | | | | | | |
|  |  |  |  |  |  |  |  |  |  |  |  |  |
| **Defecation frequency per week (time of measurement: 3 months)** | | | | | | | | | | | | |
| 1 | Randomised controlled trials | Very serious^1^ | Not serious | Not serious | Serious^2^ | Not serious | N=23 | N=21 |  | MD: 1 more per week (from 0.11 more per week to 1.89 more per week)  Trivial magnitude in favour (trivial in favour to small in favour) | Very low |  |
| **Stool consistency: Modified Bristol Stool Form Scale (scale 1-5) (time of measurement: 1 month)** | | | | | | | | | | | | |
| 1 | Randomised controlled trials | Very serious^1^ | Not serious | Not serious | Not serious | Not serious | N=25 | N=21 |  | MD: 0.00 higher (from 0.43 lower to 0.43 higher on the BSFS)  Trivial magnitude (trivial against to trivial in favour) | Low |  |
| **Quality of life: PedsQL scale of 0-100, higher scores indicate better QoL (time of measurement: 3 months)** | | | | | | | | | | | | |
| 1 | Randomised controlled trials | Very serious^1^ | Not serious | Not serious | Not serious | Not serious | N=26 | N=21 |  | MD: 30.00 higher (from 24.5 higher to 35.5 higher)  Moderate magnitude in favour (moderate in favour to moderate in favour) | Low |  |

^1^Downgraded due to lack of blinding and high risk of selective reporting
^2^Downgraded one level due significant imprecision

3. Additional effect: abdominal muscle training/breathing exercises/abdominal massage + laxative vs laxative (N=1)

**Notes:** Physiotherapy sessions consisted of isometric training of the abdominal muscles, diaphragmatic breathing exercises and abdominal massage (twice weekly sessions). Laxative treatment in both groups consisted of disimpaction with an enema 1-5 days and maintenance treatment with magnesiumhydroxide.
**Question:** Should abdominal muscle training/breathing exercises/abdominal massage as addition to laxatives vs laxatives alone be used for the treatment of functional constipation
**Bibliography**: Silva 2013

GRADE

| **Quality assessment** | | | | | | | **No of patients** | | **Effect** | | **Quality** | **Importance** |
| --- | --- | --- | --- | --- | --- | --- | --- | --- | --- | --- | --- | --- |
|  |  |  |  |  |  |  |  |  |  |  |  |  |
| **No of studies** | **Design** | **Risk of bias** | **Inconsistency** | **Indirectness** | **Imprecision** | **Other considerations** | **abdominal muscle training/breathing exercises/abdominal massage + laxative** | **Laxative** | **Relative (95% CI)** | **Absolute** |  |  |
| **Treatment success – not reported** | | | | | | | | | | | | |
|  |  |  |  |  |  |  |  |  |  |  |  |  |
| **Withdrawals due to Adverse Events at study end (time of measurement: 6 weeks)** | | | | | | | | | | | | |
| 1 | Randomised controlled trials | Very serious^1^ | Not serious | Not serious | Very serious^2^ | Not serious | 2/36 | 8/36 | RR: 0.25 (0.06 to 1.10) | 167 less per 1000 (from 209 less to 22 more per 1000)  Large magnitude in favour (large in favour to moderate against) | Very low |  |
| **Defecation frequency – days per week with defecation (time of measurement: 6 weeks)** | | | | | | | | | | | | |
| 1 | Randomised controlled trials | Very serious^1^ | Not serious | Not serious | Serious^3^ | Not serious | N=36 | N=36 |  | MD: 1.20 higher (from 0.25 lower to 2.15 higher)  Small magnitude in favour (trivial against to small in favour) | Very low |  |

^1^Downgraded two levels due to unclear method of randomization and allocation concealment, and unclear risk of selective reporting
^2^Downgraded two levels due to very serious imprecision
^3^Downgraded one level due to significant imprecision

Dry cupping

1. Dry cupping vs laxative (N=1)

**Notes:** laxative used was PEG
**Question:** Should dry cupping vs laxatives be used for the treatment of functional constipation
**Bibliography**: Shahamat 2016

GRADE

| **Quality assessment** | | | | | | | **No of patients** | | **Effect** | | **Quality** | **Importance** |
| --- | --- | --- | --- | --- | --- | --- | --- | --- | --- | --- | --- | --- |
|  |  |  |  |  |  |  |  |  |  |  |  |  |
| **No of studies** | **Design** | **Risk of bias** | **Inconsistency** | **Indirectness** | **Imprecision** | **Other considerations** | **Dry cupping** | **Laxative** | **Relative (95% CI)** | **Absolute** |  |  |
| **Treatment success (time of measurement: 12 weeks)** | | | | | | | | | | | | |
| 1 | Randomised controlled trials | Very serious^1^ | Not serious | Not serious | Serious^2^ | Not serious | 46/60 | 50/60 | RR: 0.92 (0.77 to 1.10) | 67 less per 1000 (from 192 less to 83 more per 100)  Small magnitude against (moderate against to small in favour) | Very low |  |
| **Withdrawals due to Adverse Events at study end (time of measurement: 12 weeks)** | | | | | | | | | | | | |
| 1 | Randomised controlled trials | Very serious^1^ | Not serious | Not serious | Very serious^3^ | Not serious | 2/60 | 0/60 | RR: 5.00 (0.25 to 102.00) | 67 more per 1000 (from 13 less to 1000 more per 1000)^4^  Large magnitude against (small in favour to large against) | Very low |  |
| **Defecation frequency – not adequately reported** | | | | | | | | | | | | |
|  |  |  |  |  |  |  |  |  |  |  |  |  |
| **Painful defecation and hard stools - number of patients with painful or hard bowel movements (time of measurement: 12 weeks) – incompatible outcome** | | | | | | | | | | | | |
| 1 | Randomised controlled trials | Very serious^1^ | Not serious | Not serious | Serious^2^ | Not serious | 7/60 | 10/60 | RR: 0.70 (0.29 to 1.72) | 50 less per 1000 (from 118 less to 120 more per 1000) | Very low |  |
| **Fecal incontinence – not adequately reported** | | | | | | | | | | | | |
|  |  |  |  |  |  |  |  |  |  |  |  |  |

^1^Downgraded two levels due tounclear method of allocation concealment, and unclear risk of selective reporting and lack of blinding
^2^Downgraded one level due to significant imprecision ^3^Downgraded two levels due to very serious imprecision
^4^Added one fictional event to the control group in Revman in order to calculate absolute numbers to better visualize results

The Uniformed Services Constipation Action Plan (USCAP)

1. Uniformed Services constipation action plan vs standard care (N=1)

**Notes:** pharmacotherapy was not standardized for all patients. But analyses showed no statistically significant differences in number of patients receiving a clean out, or treatment with osmotic laxative only or in combination with a stimulant laxative, and no differences in dosage were found. Only reported for patients reaching end of study. Contacted authors, but no data were available for the dropouts. Therefore we decided we could not perform any reliable analyses and no GRADE assessment was performed.
**Question:** Should The Uniformed Services Constipation Action Plan (USCAP) vs standard care be used for the treatment of functional constipation?
**Bibliography**: Reeves 2022

No complete GRADE assessment could be performed.

| **Quality assessment** | | | | | | | **No of patients** | | **Effect** | | **Certainty** | **Importance** |
| --- | --- | --- | --- | --- | --- | --- | --- | --- | --- | --- | --- | --- |
|  |  |  |  |  |  |  |  |  |  |  |  |  |
| **No of studies** | **Design** | **Risk of bias** | **Inconsistency** | **Indirectness** | **Imprecision** | **Other considerations** | **USCAP** | **Standard care** | **Relative (95% CI)** | **Absolute** |  |  |
| **Treatment success (time of measurement: 4 months)** | | | | | | | | | | | | |
| 1 | Randomised controlled trials | Very serious^1^ | Not serious | Not serious |  | Not serious | Randomized number unclear | Randomized number unclear |  |  |  |  |
| **Withdrawals due to Adverse Events at study end (time of measurement: 4 months)** | | | | | | | | | | | | |
| 1 | Randomised controlled trials | Very serious^1^ | Not serious | Not serious |  | Not serious | Unclear | Unclear |  |  |  |  |
| **Defecation frequency – not reported** | | | | | | | | | | | | |
|  |  |  |  |  |  |  |  |  |  |  |  |  |
| **Stool consistency: number of patients who achieved a PBSFS 3 or 4 achieved at study end (Pediatric Bristol Stool Form Scale) scale 1-5 (time of measurement: 4 months)** | | | | | | | | | | | | |
| 1 | Randomised controlled trials | Very serious^1^ | Not serious | Not serious |  | Not serious | Unclear | Unclear |  |  |  |  |
| **Fecal incontinence: number of patients with fecal incontinence reduction at study end (time of measurement: 4 months)** | | | | | | | | | | | | |
| 1 | Randomised controlled trials | Very serious^1^ | Not serious | Not serious |  | Not serious | Unclear | Unclear |  |  |  |  |

^1^Downgraded two levels due to high risk of allocation bias, unclear attrition bias and unclear other bias

Fluid

1. Increased water intake vs control (N=1)

**Notes:** measurement of spread was not reported, therefore data could not be analyzed.
**Question:** Should increased water intake vs standard care be used for the treatment of functional constipation?
**Bibliography**: Young 1998

Data were not adequately reported. No complete GRADE analyses could be performed.

| **Quality assessment** | | | | | | | **No of patients** | | **Effect** | | **Certainty** | **Importance** |
| --- | --- | --- | --- | --- | --- | --- | --- | --- | --- | --- | --- | --- |
|  |  |  |  |  |  |  |  |  |  |  |  |  |
| **No of studies** | **Design** | **Risk of bias** | **Inconsistency** | **Indirectness** | **Imprecision** | **Other considerations** | **Increased water** | **Control** | **Relative (95% CI)** | **Absolute** |  |  |
| **Treatment success – not reported** | | | | | | | | | | | | |
|  |  |  |  |  |  |  |  |  |  |  |  |  |
| **Withdrawals due to Adverse Events at study end – not reported** | | | | | | | | | | | | |
|  |  |  |  |  |  |  |  |  |  |  |  |  |
| **Defecation frequency per week (time of measurement: 3 weeks)** | | | | | | | | | | | | |
| 1 | Randomised controlled trials | Very serious^1^ | Not serious | Not serious |  | Not serious | N=36 | N=36 |  | Not estimable |  |  |
| **Stool consistency: consistency on Stool Consistency Continuum (1=watery, 7/8 = hard)** | | | | | | | | | | | | |
| 1 | Randomised controlled trials | Very serious^1^ | Not serious | Not serious |  | Not serious | N=36 | N=36 |  | Not estimable |  |  |

^1^Downgraded two levels due to unclear risk of bias in all domains

2. Increased water intake vs hyperosmolar liquid (N=1)

**Notes:** measurement of spread was not reported, therefore data could not be analyzed.
**Question:** Should increased water intake vs hyperosmolar liquid be used for the treatment of functional constipation?
**Bibliography**: Young 1998

Data were not adequately reported. No complete GRADE analyses could be performed.

| **Quality assessment** | | | | | | | **No of patients** | | **Effect** | | **Certainty** | **Importance** |
| --- | --- | --- | --- | --- | --- | --- | --- | --- | --- | --- | --- | --- |
|  |  |  |  |  |  |  |  |  |  |  |  |  |
| **No of studies** | **Design** | **Risk of bias** | **Inconsistency** | **Indirectness** | **Imprecision** | **Other considerations** | **Increased water intake** | **Hyperosmolar liquid** | **Relative (95% CI)** | **Absolute** |  |  |
| **Treatment success – not reported** | | | | | | | | | | | | |
|  |  |  |  |  |  |  |  |  |  |  |  |  |
| **Withdrawals due to Adverse Events at study end – not reported** | | | | | | | | | | | | |
|  |  |  |  |  |  |  |  |  |  |  |  |  |
| **Defecation frequency per week (time of measurement: 3 weeks)** | | | | | | | | | | | | |
| 1 | Randomised controlled trials | Very serious^1^ | Not serious | Not serious |  | Not serious | N=36 | N=36 |  | Not estimable |  |  |
| **Stool consistency: consistency on Stool Consistency Continuum (1=watery, 7/8 = hard)** | | | | | | | | | | | | |
| 1 | Randomised controlled trials | Very serious^1^ | Not serious | Not serious |  | Not serious | N=36 | N=36 |  | Not estimable |  |  |
